# (Apo)Lipoprotein Profiling with Multi‐Omics Analysis Identified Medium‐HDL‐Targeting *PSRC1* with Therapeutic Potential for Coronary Artery Disease

**DOI:** 10.1002/advs.202413491

**Published:** 2025-02-22

**Authors:** Yingmei Li, Sihan Wang, Ling Liu, Hao Cai, Yacan Huang, Mingjing Gao, Xiaogang Zhang, Qingqing Wu, Gaokun Qiu

**Affiliations:** ^1^ Ministry of Education and State Key Laboratory of Environmental Health (Incubating) School of Public Health Tongji Medical College Huazhong University of Science and Technology Wuhan 430030 China; ^2^ SCIEX Application Support Center Shanghai 200050 China; ^3^ Department of Cardiology Zhongnan Hospital of Wuhan University Wuhan 430062 China; ^4^ Institute of Myocardial Injury and Repair Wuhan University Wuhan 430062 China

**Keywords:** (apo)lipoprotein profiling, atherosclerotic cardiovascular disease, carotid intima‐media thickness, colocalization, mendelian randomization, multi‐omics

## Abstract

Identification of (apo)lipoprotein subclasses causally underpinning atherosclerosis may lead to identification of novel drug targets for treatment of atherosclerotic cardiovascular disease (ASCVD). In this study, observational and genetic associations between (apo)lipoprotein profile and carotid intima‐media thickness‐assessed atherosclerosis, and risks of coronary artery disease (CAD) and ischemic stroke (IS) are assessed, using data from the UK Biobank study, with further exploration of potential drug target for these two ASCVD subtypes through multi‐omics analysis integrating genetic, transcriptomic, and proteomic data. Cholesteryl ester content in medium high‐density lipoprotein causally protective of atherosclerosis is identified, plus a target gene, *PSRC1*, with therapeutic potential for CAD, but not IS, supported by consistent evidence from multi‐omics layers of data, which also reveals that such therapeutic potential may be through downregulation of circulating proteins including *TRP1*, *GRNs*, and *Pla2g12b*, and upregulation of *Neo1*. The results provide strong evidence as well as mechanistic clues of *PSRC1*’s therapeutic potential for CAD.

## Graphical abstract

Lipid content in medium high‐density lipoprotein (HDL) particles, particularly cholesteryl esters, is identified as causally protective of atherosclerosis, and further ASCVD. Evidence from multi‐omics layers of data consistently supports *PSRC1* with therapeutic potential for coronary artery disease (CAD), but not ischemic stroke. This study also provides mechanistic clues of *PSRC1*’s therapeutic potential through its downstream proteins.

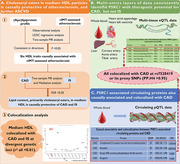

## 1 Introduction

Atherosclerotic cardiovascular disease (ASCVD) remains the leading cause of death worldwide, accounting for nearly half of the deaths caused by CVD.^[^ ^1^ ^]^ The deposition of atherogenic lipoproteins in vessel walls, mainly low‐density lipoprotein cholesterol (LDL‐c),^[^ ^2^ ^]^ constitutes the major driver of atherosclerosis and underpins the development of ASCVD,^[^ ^3^ ^]^ while high‐density lipoprotein cholesterol (HDL‐c) is considered to be atheroprotective according to observational studies.^[^ ^4^ ^]^ Currently, LDL‐c‐targeting therapies, such as statins, stand as the bulwark in the prevention and treatment of ASCVD, with a remarkable reduction of event rates.^[^ ^5^ ^]^ However, significant residual cardiovascular risk remains for recurrent events even with aggressive LDL‐c reduction,^[^ ^6^ ^]^ and in the context of low LDL‐c level, there is a phenomenon of “lipid paradox” from observational studies that low LDL‐c was associated with increased mortality risk.^[^ ^7^ ^]^ Attempts have been made to tackle the residual risk issue in LDL‐c‐lowering therapies, with modulation of triglycerides (TGs) or remnant cholesterol, while the results of risk reduction remain inconsistent and uncertain.^[^ ^8^ ^]^ In fact, drug development for CVD has long been stagnating, with less than half the probability of launch for entering each phase of clinical trials than for anticancer therapies.^[^ ^9^ ^]^ The still‐being tremendous disease burden of ASCVD^[^ ^10^ ^]^ highlighted the urgent need to identify novel therapeutic targets beyond the focus on traditional clinical lipids.

Lipoproteins are macromolecular complexes that vary in density and lipid composition between and within classes,^[^ ^11^ ^]^ which cannot be captured by traditional clinical lipid measures, while with the introduction of nuclear magnetic resonance (NMR) spectroscopy, lipoprotein characteristics can be resolved in thorough detail.^[^ ^12^ ^]^ A number of observational studies using NMR‐measured (apo)lipoprotein profiles revealed association patterns in relation to the particle size of very‐low‐density lipoproteins (VLDL) with CHD risk,^[^ ^13^ ^]^ and that of HDLs with CVD and mortality risk,^[^ ^14^, ^15^, ^16^, ^17^ ^]^ and with risk of heart failure,^[^ ^18^ ^]^ although controversial results were yielded.^[^ ^19^, ^20^, ^21^ ^]^ Another Mendelian randomization (MR) study reported an inverse causal association with CAD risk of medium HDL particles.^[^ ^22^ ^]^ These studies highlighted that the atherogenic or anti‐atherosclerosis properties of lipoproteins might be closely related to their characteristics. Apart from MR analysis, there are other analytic approaches such as colocalization analysis, which together enable the integrative use of multi‐omics genetic association data, from which critical causal inferences can be derived and possibly further lead to the identification of novel drug targets,^[^ ^23^ ^]^ as accumulating evidence suggests that the existence of genetic support of causation increases several‐fold the possibility for a potential drug target to succeed in clinical trials.^[^ ^24^, ^25^ ^]^ By means of multi‐omics integrative analysis and utilization of NMR‐resolved (apo)lipoprotein profile data, it can be promisingly anticipated to isolate the causally atherosclerosis‐associated lipoprotein subclasses and prioritize causal genetic targets with therapeutic potential for ASCVD.

In this study, we aimed to identify the causally atherogenic or anti‐atherosclerosis (apo)lipoprotein subclasses and further search for potential drug targets with therapeutic potential for ASCVD. To this end, we first performed observational association analyses of NMR‐based measurements of (apo)lipoprotein characteristics with carotid intima‐media thickness (cIMT), a validated assessment of early‐stage atherosclerosis,^[^ ^26^ ^]^ and with ASCVD risk,^[^ ^27^ ^]^ using data from the UK Biobank (UKB) study. From this start, we employed various analytical approaches, including linkage disequilibrium score regression (LDSC), MR analysis, and colocalization analysis, which respectively assessed existence of shared overall genetic architecture,^[^ ^28^ ^]^ causality of association,^[^ ^29^ ^]^ and shared causal genetic variant,^[^ ^30^ ^]^ plus integration with multi‐tissue expression quantitative trait loci (eQTL) data and circulating protein QTL (pQTL) data, in quest for identification of novel therapeutic targets for ASCVD.

## 2 Results

### 2.1 Association of Plasma (apo)Lipoprotein Profile with cIMT‐Assessed Atherosclerosis

The baseline characteristics of UKB participants included in this study are described in **Table** **1**. There were a total of 26 091 participants with both cMIT measurements and NMR‐derived (apo)lipoprotein data, the latter of which were released in two phases. There was no significant difference in demographic characteristics between participants within Phase I (N = 10 375) and Phase II (N = 15 716) data releases (all *p* >0.05). Among the total 26 091 participants, the average age was 69.78 (±7.56) years, 49.5% of them were men, and 92.5% of them were of European ancestry. A total of 6.4% of the participants were currently smoking, and 95.3% were currently drinking at the time of baseline interview, with a vast majority (70.2%) of them engaging in moderate to high‐intensity physical activities. The participants had an average body mass index (BMI) level of 26.64 (±4.18) kg m^−2^, and the systolic and diastolic blood pressures (SBP and DBP) were 135.4 (±17.25) mmHg and 81.48 (±9.65) mmHg, respectively. The participants had a baseline diabetes prevalence of 2.6%, and 38.0% of them had a family history of CVD. Of note, 12.0% of the participants were using cholesterol‐lowering medications.

**Table 1:** Baseline characteristics of participants from the UKB study with both (apo)lipoprotein profile and cIMT data.

Variables	Phase I [N = 10 375]	Phase II [N = 15 716]	Overall [N = 26 091]
Age, years	69.44 ± 7.61	70 ± 7.53	69.78 ± 7.56
Men, (%)	5091 (49.6%)	7812 (49.7%)	12 903 (49.5%)
Ethnicity, (%)			
White	9583 (92.4%)	14 561 (92.7%)	24 144 (92.5%)
Mixed	304 (2.9%)	462 (2.9%)	766 (2.9%)
Indian	342 (3.3%)	472 (3.0%)	814 (3.1%)
Black	32 (0.3%)	51 (0.3%)	83 (0.3%)
Chinese	31 (0.3%)	58 (0.4%)	89 (0.3%)
Other	60 (0.6%)	67 (0.4%)	127 (0.5%)
BMI, kg m^−2^	26.64 ± 4.21	26.64 ± 4.15	26.64 ± 4.18
Smoking status (%)			
Never smoker	6267 (60.4%)	9502 (60.5%)	15 769 (60.4%)
Previous smoker	3410 (32.9%)	5192 (33.0%)	8602 (33.0%)
Current smoker	666 (6.4%)	991 (6.3%)	1657 (6.4%)
Drinking status (%)			
Never drinker	249 (2.4%)	420 (2.7%)	669 (2.6%)
Previous drinker	225 (2.2%)	331 (2.1%)	556 (2.1%)
Current drinker	9893 (95.4%)	14 959 (95.2%)	24 852 (95.3%)
Physical activity (%)			
Moderate	3785 (36.5%)	5682 (36.2%)	9467 (36.3%)
High	3493 (33.7%)	5355 (34.1%)	8848 (33.9%)
Systolic blood pressure, mmHg	135.19 ± 16.75	135.54 ± 17.57	135.4 ± 17.25
Diastolic blood pressure, mmHg	81.39 ± 9.48	81.54 ± 9.76	81.48 ± 9.65
Cholesterol‐lowering medication use, (%)	1232 (11.9%)	1892 (12.0%)	3124 (12.0%)
Diabetes, (%)	278 (2.7%)	392 (2.5%)	670 (2.6%)
Family history of CVD, (%)	3254 (31.4%)	6667 (42.4%)	9921 (38.0%)

BMI, body mass index; cIMT, carotid intima‐media thickness; CVD, cardiovascular diseases.

Phase I and phase II indicated the two phases of releases of (apo)lipoprotein profile data.

**Figure 1:**
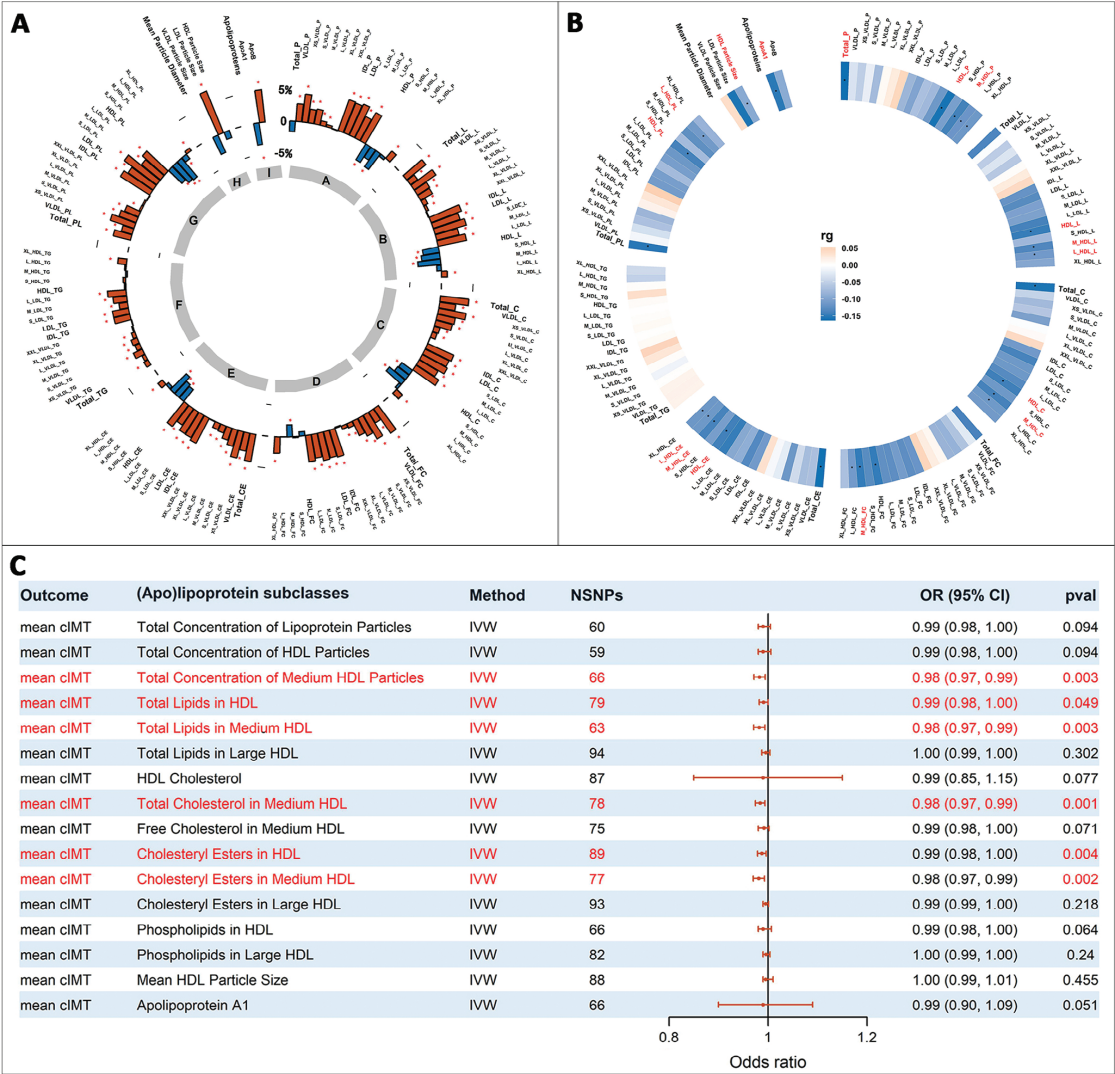
Observational and genetic associations between NMR‐based (apo)lipoprotein profile and mean cIMT. A) Observational association between (apo)lipoprotein profile and mean cIMT. (Apo)lipoprotein measurements included VLDL, LDL, HDL, and 14 subclasses of these lipoproteins discriminated by particle size and lipid composition (including total lipids, total cholesterol, cholesteryl esters, free cholesterol, triglycerides, and phospholipids), as well as concentrations of lipoprotein particles, totaling 131 (apo)lipoprotein measurements, from cluster A to I in Table S23, Supporting Information. Estimates are percent changes in mean cIMT per fold increase in each (apo)lipoprotein measurement from meta‐analysis of the two phases of data. Multivariate linear regression was separately conducted within participants from the two phases of NMR data adjusting for sex, age, ethnicity, BMI, SBP, DBP, smoking status, drinking status, physical activity level, diet, prevalent diabetes, use of cholesterol‐lowering medications, family history of cardiovascular disease, and time‐window between lipoprotein and cIMT measurements, and then meta‐analyzed. Data on (apo)lipoprotein and mean cIMT were all logarithmically transformed prior to analysis. Blue bars indicated negative associations; orange bars indicated positive associations, and red star (*) above the bar indicated nominal significance. The abbreviations of (apo)lipoprotein measurements were shown in the outer circle of the graph. *p* < 0.05 was considered as nominally significant, and a looser criterion of nominal significance was employed at first in this stage of analyses to include as many as possible (apo)lipoproteins into the following evaluation of association with and therapeutic potential for ASCVD. B) Genetic correlation between (apo)lipoprotein profile and mean cIMT from LDSC regression analysis. The rg estimates are shown in the figure. Positive correlations were shown in orange, and negative correlations were shown in blue, while the black star on the circle bar indicated *p* < 0.05. The abbreviations of (apo)lipoprotein measurements were shown in the outer circle of the graph, and the red font indicated that these (apo)lipoprotein measurements showed nominally significant and consistent genetic correlation with mean cIMT as in the observational association analysis. *p* < 0.05 was considered as nominally significant. C) Forest plot showing causal association between (apo)lipoprotein profile and mean cIMT from two‐sample MR analysis. The red font indicated nominally significant association. IVW denotes inverse variance weighted method. *p* < 0.05 was considered as nominally significant. Total_P, total concentration of lipoprotein particles (_P denotes total concentration of each lipoprotein particles); Total_L, total lipids in lipoprotein particles (_L denotes total lipids); Total_C, total cholesterol (_C denotes cholesterol); Total_FC, total free cholesterol (_FC denotes free cholesterol); Total_CE, total cholesteryl esters (_CE denotes cholesteryl esters); Total_TG, total triglycerides (_TG denotes triglycerides); Total_PL, total phospholipids in lipoprotein particles (_PL denotes phospholipids); IDL, intermediate density lipoproteins; VLDL_size, mean VLDL particle size; LDL_size, mean LDL particle size; HDL_size; mean HDL particle size; ApoA1, apolipoprotein A1; ApoB; apolipoprotein B. cIMT, carotid intima‐media thickness; NMR, nuclear magnetic resonance; VLDL, very‐low‐density lipoprotein; LDL, low‐density lipoprotein; HDL, high‐density lipoprotein; BMI, body mass index; SBP, systolic blood pressure; DBP, diastolic blood pressure; ASCVD, atherosclerotic cardiovascular disease; LDSC, linkage disequilibrium score regression; MR, mendelian randomization.

NMR‐based (apo)lipoprotein profiling yielded 131 lipoprotein measurements, including VLDL, LDL, HDL, and 14 subclasses of these lipoproteins discriminated by particle size and lipid composition (including total lipids, total cholesterol, cholesteryl esters, free cholesterol, triglycerides, and phospholipids), as well as concentrations of lipoprotein particles. Between participants from the two data phases, 126 of the 131 lipoprotein measurements showed consistent directions of association with mean cIMT (Table S1, Supporting Information). In meta‐analysis, a total of 113 lipoprotein measurements achieved nominal significance (all *p* < 0.05; **Figure** **1**A) with no heterogeneity observed (all *p* for heterogeneity >0.05; *I* ^2^ = 0.00% to 55.16%; Table S1, Supporting Information). The association profile showed a pattern that apolipoprotein B‐containing lipoprotein subclasses (VLDL, intermediate‐density lipoprotein [IDL], and LDL) were generally positively associated with mean cIMT, while by contrast, HDL subclasses except very large HDL showed negative associations.

In the following LDSC regression analysis, 16 lipoprotein measurements showed genetic correlations with cIMT in consistent directions with observational associations (rg = −0.1637 to −0.0932, *p* < 0.05), including 14 HDL measurements, as well as total concentration of lipoprotein particles (rg = −0.1637) and apolipoprotein A1 (rg = −0.1508). The 14 HDL measurements included mean HDL particle size, concentration of HDL particles, and lipid contents in HDL (total lipids, total cholesterol, free cholesterol, cholesteryl esters, and phospholipids), mainly in medium and large HDL subclasses, with rg estimates ranging from −0.0932 (*p* = 0.020) for mean HDL particle size to −0.1576 (*p* = 0.004) for total concentration of HDL particles (Figure 1B; Table S2, Supporting Information). Noteworthy, we did not observe any significant genetic correlation between LDL measurements and cIMT‐assessed atherosclerosis (rg ranging from −0.1466 to −0.0673 and 0.0015 to 0.0091, *p* > 0.05), suggesting that their shared genetic architecture might be limited.

In subsequent MR analyses of the 16 lipoprotein measurements with cIMT (F statistics ranging from 23.82 to 3116.47, indicating no weak instrument bias [F statistic ≥10];^[^ ^31^ ^]^ Table S3, Supporting Information), only six HDL measurements showed causal associations according to the inverse variance weighted (IVW) method, all being negative, which were total concentration of medium HDL particles, total lipids in HDL and medium HDL, total cholesterol in medium HDL, and cholesteryl esters in HDL and medium HDL (*p* = 0.001 to 0.049; Figure 1C), highlighting medium HDL as the causal HDL subclass and cholesteryl esters as the causal lipid content. In sensitivity analyses (Table S4 and S5, Supporting Information), methods including radial IVW, MR Egger, and weighted median all showed consistent results with the IVW method, at least maintained consistent directionality of effect estimates, and the intercept statistic in MR Egger analysis indicated no pleiotropy (all *p >* 0.05). Although Cochran Q test indicated the existence of heterogeneity (*p* < 0.05) for these lipoprotein measurements except the total concentration of lipoprotein particles and HDL particles (*p* >0.05), results from the leave‐one‐out method suggested that the removal of any of the instrumental single nucleotide polymorphism (SNP) did not alter the direction of the effect estimates. Biases attributed to sample overlap (<10%) were also estimated to be negligible, all with absolute biases <0.001 (type 1 error rate = 0.05 for all outcomes; Figures S1–S16, Supporting Information).

### 2.2 Medium HDLs Stood Out as Causally Linked to CAD and IS Risk but Colocalized with these Two ASCVD Subtypes at Divergent Genetic Loci

**Figure 2:**
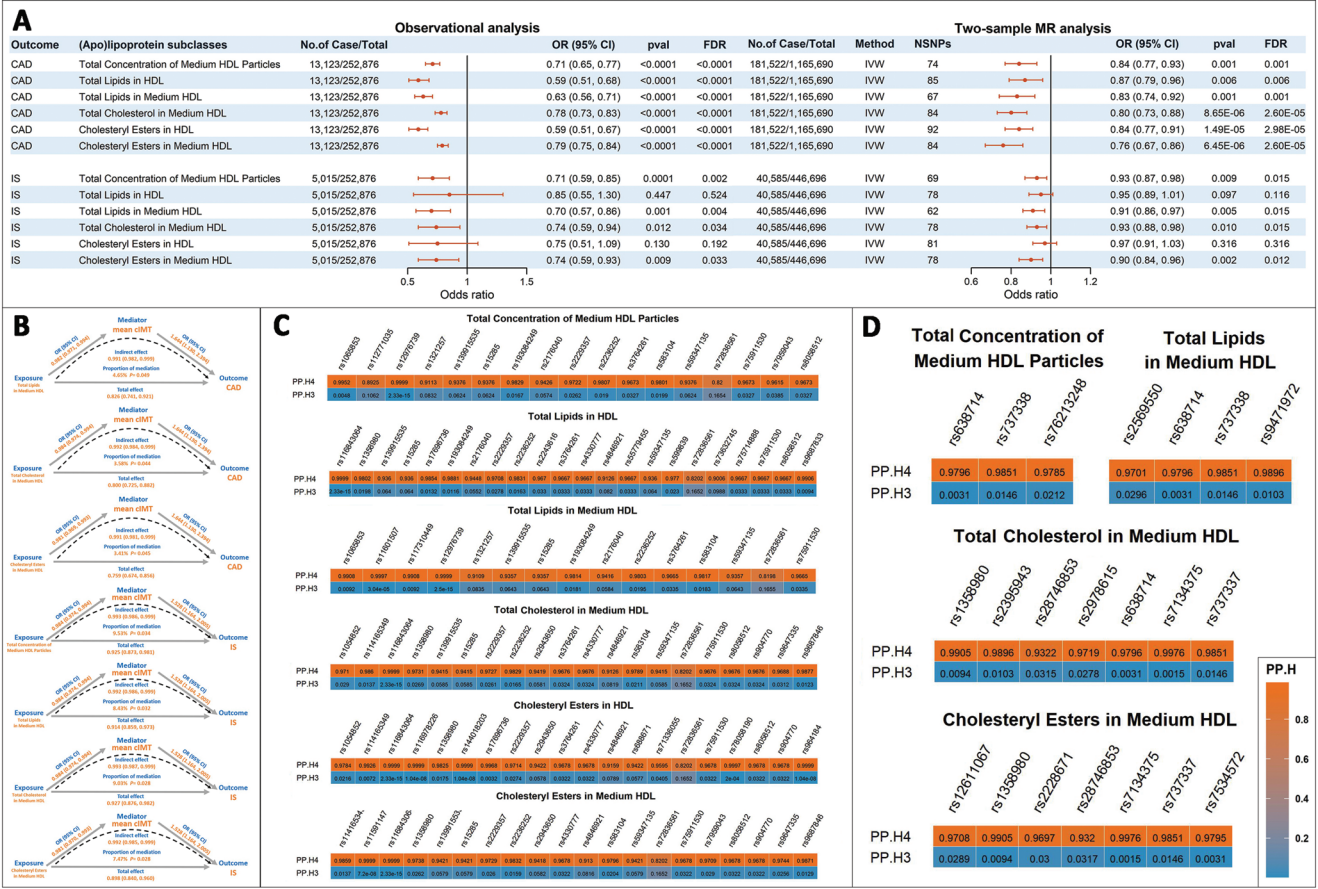
Observational and causal association, and colocalization of (apo)lipoproteins with CAD and IS. A) Forest plot for significant observational and causal associations of HDL subclasses with CAD and IS. The six HDL subclasses casually associated with mean cIMT were shown in red font in Figure 1C. *FDR* <0.05 was considered statistically significant. B) Significant mediation through cIMT‐assessed atherosclerosis in the causal association of HDL subclasses with CAD and IS by two‐step MR analysis. The upper three panels showed the mediation pathway of HDL subclasses → cIMT‐assessed atherosclerosis → CAD, in which total lipids, total cholesterol, and cholesteryl esters in medium HDL demonstrated marginally significant mediation. The lower four panels showed the mediation pathway of HDL subclasses → cIMT‐assessed atherosclerosis → IS, in which concentration of medium HDL particles, total lipids, total cholesterol, and cholesteryl esters in medium HDL demonstrated significant mediation. Total effect indicates the effect of exposure (HDL subclasses) on outcome (CAD and IS). Indirect effect indicates the effect of exposure on outcome through the mediator (cIMT‐assessed atherosclerosis). C) Significant colocalization between HDL subclasses and CAD. PP.H3 denotes association with CAD risk as well as HDL subclasses, but at distinct causal variants; PP.H4 denotes association with both traits, with a shared causal variant. A PP.H4 greater than 0.8 indicates significant colocalization. D) Significant colocalization between HDL subclasses and IS. PP.H3 denotes association with IS risk as well as HDL subclasses, but at distinct causal variants; PP.H4 denotes association with both traits, with a shared causal variant. A PP.H4 greater than 0.8 indicates significant colocalization. CAD, coronary artery disease; IS, ischemic stroke; cIMT, carotid intima‐media thickness; *FDR*, *false discovery rate*; MR, Mendelian randomization; HDL, high‐density lipoprotein; IVW, inverse variance weighted method.

In subsequent observational association analysis and two‐sample MR analysis with risks of CAD and IS (F statistics ranging from 20.92 to 2825.38; Table S6, Supporting Information), we found that all the six atherosclerosis‐associated HDL measurements were causally associated with reduced CAD risk (odds ratios [OR] ranging from 0.88 to 0.91 [all *p*‐value and *False Discovery Rates* (*FDRs*) <0.0001] per standard deviation (SD) increase in each log‐transformed HDL measurement, and ranging from 0.76 to 0.87 [*p* = 6.45E‐06 to 0.006; *FDR* = 2.60E‐05 to 0.006] per SD increase in each genetically‐determined HDL measurement; **Figure** **2**A), and also with IS risk except total lipids and cholesteryl esters in HDL (ORs ranging from 0.92 to 0.94 [*p* = 0.001 to 0.012; *FDR* = 0.006 to 0.018] for observational association and 0.90 to 0.93 [*p* = 0.002 to 0.010; *FDR* = 0.012 to 0.015] for MR analysis; Figure 2A). Sensitivity analysis showed consistent results with no pleiotropy (Tables S7 and S8, Supporting Information). In multivariate MR (MVMR) analyses (Table S9, Supporting Information), the associations of all six atherosclerosis‐associated HDL measurements with CAD risk persisted after adjustment for smoking and alcohol drinking, the two most heritable ASCVD risk factors^[^ ^32^, ^33^ ^]^ (ORs ranging from 0.77 to 0.87 [*p* = 1.34E‐06 to 0.007; *FDR* = 8.05E‐06 to 0.007]), and adjustment for LDL‐c, the currently recommended primary target for lipid management^[^ ^3^ ^]^ (ORs = 0.82 to 0.90 [*p* = 6.21E‐05 to 0.027; *FDR* = 3.73E‐04 to 0.032]) except total lipids in medium HDL with marginal significance (*FDR* = 0.055). For IS risk, only total cholesterol and cholesteryl esters in medium HDL maintained their associations after adjustment for smoking and alcohol drinking (both OR = 0.91 [*p* = 0.002; *FDR* = 0.007]), which also retained marginal significance upon LDL‐c adjustment (*p* = 0.019 and 0.075; *FDR* = 0.062 and 0.075), highlighting the robustness of causal association between cholesteryl esters in medium HDL and ASCVD risk.

Through mediation analysis with MR, we found that the associations of total lipids, total cholesterol, and cholesteryl esters in medium HDL with CAD risk were mediated through cMIT‐assessed atherosclerosis by 4.65%, 3.58%, and 3.41% with marginal significance (*p* all <0.05, *FDRs* <0.1; Figure 2B). Similarly, the associations of concentration of medium HDL particles, total lipids, total cholesterol, and cholesteryl esters in medium HDL with IS risk were significantly mediated through atherosclerosis by 9.53%, 8.43%, 9.03%, and 7.47% (*p* all <0.05, *FDRs* <0.05; Figure 2B). The mediation proportions also supported that cholesteryl esters seemed to be the driving lipid content within medium HDL underlying CAD and IS risk.

These HDL measurements also showed strong colocalization with both CAD and IS (Figure 2C,D; Table S10, Supporting Information). Specifically, total concentration of medium HDL particles, total lipids in HDL and in medium HDL, total cholesterol in medium HDL, and cholesteryl esters in HDL and in medium HDL colocalized with CAD at 17, 22, 15, 20, 20, and 20 genetic variants (PP.H4 = 0.8202 to 0.9999), respectively, with shared causal variants including rs75911530 (chr16q13, PP.H4 = 0.9673, 0.9667, 0.9665, 0.9676, 0.9678, and 0.9678) and rs72836561 (chr17q21.31, PP.H4 = 0.8200, 0.8202, 0.8198, 0.8202, 0.8202, and 0.8202). As for IS, total concentration of medium HDL particles, total lipids, total cholesterol, and cholesteryl esters in medium HDL showed colocalization at 3, 4, 7, and 7 genetic variants (PP.H4 = 0.9320 to 0.9976), respectively, with no shared causal variants. Notably, colocalized causal variants with CAD and IS were totally divergent, with between‐SNP r^2^ all less than 0.01.

### 2.3 Integration with Multi‐Tissue eQTL Data Revealed Circulating Proline/Serine‐Rich Coiled‐Coil 1 (*PSRC1*) Gene Causally Associated with CAD Risk and Colocalized at the Shared Causal Variant rs7528419

**Figure 3:**
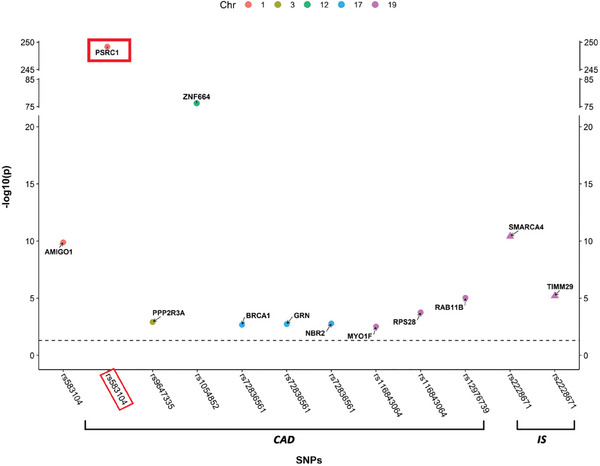
Genes *cis*‐associated with shared causal variants between HDL subclasses and CAD or IS in whole blood retrieved from the eQTLGen Consortium database. This figure was generated using data from eQTLGen consortium database (https://eqtlgen.org/), and the 12 genes casually associated with CAD (shown as filled dot) and IS (shown as filled triangle) were shown in this figure. SNPs were ordered on the x‐axis according to their genomic position. The *y*‐axis represented the strength of the association measured as −log10 transformed p values. The black dotted line marked the significance threshold of *p* <0.05. The five different colors represented different chromosomes, as shown in the legend at the top of the figure. HDL, high‐density lipoprotein; CAD, coronary artery disease; IS, ischemic stroke.

**Figure 4:**
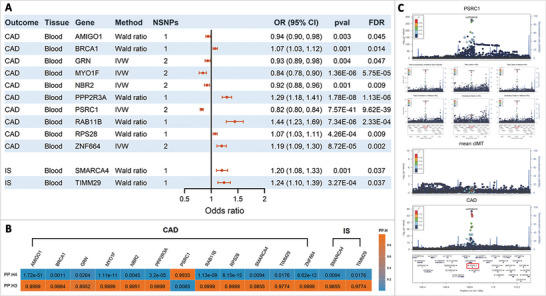
Causal association and colocalization of circulating gene expression level with CAD and IS. A) Forest plot showing causal associations of circulating genes with CAD/IS. IVW denotes inverse variance weighted method. *FDR* < 0.05 was considered statistically significant. B) Colocalization between circulating genes and CAD/IS. PP.H3 denotes association with CAD or IS risk as well as circulating gene expression, but at distinct causal variants; PP.H4 denotes association with both traits, with a shared causal variant. A PP.H4 greater than 0.8 indicates significant colocalization. C) Regional plot for the identified shared causal variants, rs7528419, in association with *PSRC1* expression in whole blood, the six atherosclerosis‐associated HDL subclasses, mean cIMT, and CAD. This figure was generated using data from eQTLGen consortium database (https://eqtlgen.org/), IEU OPEN GWAS (https://gwas.mrcieu.ac.uk/datasets/), Aragam et al. (Reference #34), and Strawbridge et al. (Reference #69), respectively. CAD, coronary artery disease; IS, ischemic stroke; *FDR*, *false discovery rate*; HDL, high‐density lipoprotein; cIMT, carotid intima‐media thickness; *PSRC1*, proline/serine‐rich coiled‐coil 1.

We then performed multi‐tissue transcriptomic search of genes *cis*‐associated with the aforementioned colocalized causal SNPs. From genome‐wide association (GWA) data of whole blood gene expression within the eQTLGen Consortium database, we retrieved 127 genes associated with the shared causal variants between the six HDL measurements and CAD (*P* = 7.02E‐250 to 0.047) and 81 genes (*P* = 2.00E‐248 to 0.049) for IS, with no overlap (Table S11, Supporting Information). Similarly, another 94 genes across seven tissues (including whole blood, artery coronary, artery aorta, artery tibial, heart atrial appendage, heart left ventricle, and liver tissue) were retrieved from the Genotype‐Tissue Expression (GTEx) database, 55 for CAD (*P* = 5.17E‐161 to 7.45E‐06) and 40 for IS (*P* = 5.17E‐161 to 9.84E‐06) with one shared gene (*PEX6*). Among them, a total of 12 genes expressed in whole blood within the eQTLGen Consortium database were casually associated with CAD or IS (**Figure** **3**; F statistics ranging from 30.04 to 1789.75, Table S12, Supporting Information). Specifically, ten genes showed causal association with CAD risk (ORs = 0.82 to 0.94 and 1.07 to 1.44 [*P* = 7.57E‐41 to 0.004; *FDR* = 9.62E‐39 to 0.047]), and two with IS risk (ORs = 1.20 and 1.24 [*P* = 3.27E‐04 and 0.001; both *FDR* = 0.037]; **Figure** **4**A; Table S13, Supporting Information). Sensitivity analysis showed neither heterogeneity between SNP instruments nor pleiotropy (Table S14, Supporting Information). Only the gene *PSRC1* expressed in whole blood showed colocalization with CAD (PP.H4 = 0.9935), which indicated therapeutic potential of *PSRC1* overexpression for CAD together with its negative causal association, and the shared causal variant was identified as rs7528419, while the other 11 genes all obtained PP.H3 greater than 0.8952, indicating distinct causal variants at these loci (Figure 4B; Table S15, Supporting Information). None of the genes showed colocalization with IS (PP.H4 <0.02; Figure 4B; Table S15, Supporting Information). For *PSRC1*, per SD increase in its genetically‐determined expression level in whole blood was associated with 18% lower odds of developing CAD (95% CI: 0.80‐0.84, [*P* = 7.57E‐41, *FDR* = 9.62E‐39]; Figure 4A), and its expression level in heart left ventricle and liver also showed causal association with CAD risk [OR (95% CI): 0.71 (0.68, 0.74,[*P* = 8.11E‐57, *FDR* = 3.97E‐55]) and 0.93 (0.92, 0.94, [*P* = 8.11E‐57, *FDR* = 1.32E‐55]), respectively; Table S16, Supporting Information], although colocalization was not observed of this gene expressed in these two tissues (PP.H4 = 1.11E‐26 and 1.83E‐05, respectively; Table S17, Supporting Information). We observed no other genes expressed in tissues other than whole blood showing colocalization evidence with either CAD or IS (PP.H4 <0.009, Table S17, Supporting Information), although the limited sample size (208 to 584) of the GTEx database might have restricted the power for identification of shared causal variants.

The colocalized causal SNP, rs7528419, is located at chr1p13.3 and 4.964 kb upstream *PSRC1*, and in strong linkage disequilibrium with rs583104 (r^2^ = 0.932), the shared causal variant between the four medium HDL traits (total concentration of medium HDL particles, total lipids, total cholesterol and cholesteryl esters in medium HDL) and CAD, with a distance of 546.7 kb. The rs7528419 was also among the top SNPs at the *PSRC1* locus in association with the four medium HDL traits (data from IEU OPEN GWAS; total concentration of medium HDL particles [effect allele: G; β = 0.05, *P* = 3.40E‐23], total lipids [effect allele: G; β = 0.05, *P* = 9.30E‐30], total cholesterol [effect allele: G; β = 0.04, *P* = 1.80E‐18], and cholesteryl esters [effect allele: G; β = 0.04, *P* = 2.90E‐20] in medium HDL), also associated with *PSRC1* expression in whole blood (data from eQTLGen; effect allele: G; β = 0.49, *P* < 7.02E‐250), and CAD risk (data from Aragam et al.;^[^ ^34^ ^]^ effect allele: G; β = −0.095, *P* = 8.11E‐57; Figure 4C).

As a preliminary exploration on the possibility of adverse metabolic or inflammatory effects from *PSRC1* overexpression, we performed additional MR analysis on the association between *PSRC1* with clinical lipids and the inflammatory marker, C‐reactive protein (CRP), which, to our assurance, revealed negative associations of circulating *PSRC1* with LDL‐c, total cholesterol (TC), and TG levels (ORs = 0.74 [95% CI: 0.69 to 0.79; *P* = 8.43E‐22], 0.78 [95% CI: 0.73 to 0.82; *P* = 1.53E‐19], 0.98 [95% CI: 0.97 to 0.99; *P* = 1.91E‐09], respectively), and null association with CRP (*P* = 0.075) (Table S18, Supporting Information).

### 2.4 *PSRC1*‐Associated Circulating Proteins were also Causally Associated with CAD risk with Strong Colocalization at rs7528419 or its Proxy SNPs

We further explored the associations of *PSRC1*‐associated proteins with CAD risk. From the study of sun et al.,^[^ ^35^ ^]^ we retrieved nine circulating proteins significantly associated with rs7528419 (Table S19, Supporting Information), including Apolipoprotein B (*ApoB*; effect allele: G; β = −0.14, *P* = 5.13E‐06), Carbonic anhydrase‐related protein 10 (*CARP10*; β = −0.30, *P* = 3.02E‐23), Complement C1q tumor necrosis factor‐related protein 1 (*CTRP1*; β = −0.64, *P* = 1.07E‐112), Four‐jointed box protein 1 (*FJX1*; β = −0.26, *P* = 1.82E‐18), Granulins (*GRNs*; β = −0.83, *P* = 1.23E‐208), Group XIIB secretory phospholipase A2‐like protein (*Pla2g12b*; β = −0.43, *P* = 4.17E‐47), Hemojuvelin (*HJV*; β = −0.23, *P* = 2.40E‐14), Sodium‐coupled monocarboxylate transporter 1 (*SLC5A8*; β = −0.16, *P* = 9.33E‐08), and Neogenin (*Neo1*; β = 0.23, *P* = 2.75E‐14).

**Figure 5:**
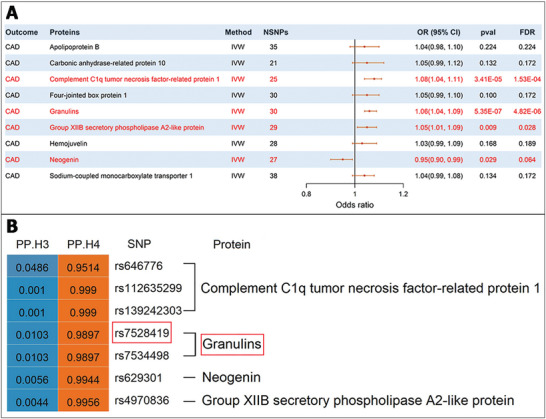
Causal association and colocalization between *PSRC1*‐associated circulating proteins and CAD. A) Forest plot showing causal associations between *PSRC1*‐associated circulating proteins and CAD. *FDR* < 0.05 was considered statistically significant. B) Colocalization between *PSRC1*‐associated circulating proteins and CAD. PP.H3 denotes association with CAD risk as well as circulating proteins, but at distinct causal variants; PP.H4 denotes association with both traits, with a shared causal variant. A PP.H4 greater than 0.8 indicates significant colocalization. *PSRC1*, proline/serine‐rich coiled‐coil 1; CAD, coronary artery disease; *FDR*, *false discovery rate*.

We then performed MR analysis between the nine proteins and CAD (F statistics ranging from 19.20 to 1012.85; Table S20, Supporting Information), and found four of the nine proteins showing casual associations with CAD (**Figure** **5**B), including three proteins showing positive associations with ORs per SD increase in their genetically‐determined levels of 1.08 (95% CI:1.04, 1.11; [*P* = 3.41E‐05, *FDR* = 1.53E‐04]) for *CTRP1*, 1.06 (95% CI:1.04, 1.09; [*P* = 5.35E‐07, *FDR* = 4.82E‐06]) for *GRNs*, and 1.05 (95% CI:1.01, 1.09; [*P* = 0.009, *FDR* = 0.028]) for *Pla2g12b*, while the remaining *Neo1* showed negative casual association with an OR of 0.95 with marginal significance (95% CI: 0.90, 0.99; [*P* = 0.029, *FDR* = 0.064]; Figure 5A; Table S21, Supporting Information). These four proteins also showed strong colocalization with CAD (PP.H4 all > 0.95, Figure 5B; Table S22, Supporting Information), and among them, *CTRP1* and *GRNs* colocalized with CAD at three and two genetic variants (PP.H4 = 0.9514 to 0.9990), respectively. Notably, *GRNs* colocalized with CAD at rs7528419 (PP.H4 = 0.9900), exactly the shared causal variant between *PSRC1* gene and CAD, and the other three proteins each colocalized with CAD at one variant in high linkage disequilibrium with rs7528419, including rs646776 (chr1p13.3, PP.H4 = 0.9514; *r* ^2^ = 1.00 with rs7528419) for *CTRP1*, rs629301 (chr1p13.3, PP.H4 = 0.9944; *r* ^2^ = 0.99 with rs7528419) for *Neo1*, and rs4970836 (chr1p13.3, PP.H4 = 0.9956; r^2^ = 0.93 with rs7528419). In addition, *CTRP1* also colocalized with CAD at rs112635299 (chr14q32.12) and rs139242303 (chr14q32.11), and *GRNs* also colocalized with CAD at rs7534498 (chr1p13.3), which all showed no linkage disequilibrium with rs7528419 (*r* ^2^ <0.001).

## 3 Discussion

In this study, we examined observational and genetic association of NMR‐based (apo)lipoprotein profile with cIMT‐assessed atherosclerosis and ASCVD risk, which consistently identified concentration and lipid content in HDL causally protective of atherosclerosis and further ASCVD, with medium HDL seemingly being the driving HDL subclass underlying such protective effect, and cholesteryl esters being the driving lipid content. These HDL measurements all showed strong colocalization with CAD and IS, the two major ASCVD subtypes, but at totally divergent genetic loci, and subsequent integration with multi‐tissue eQTL data revealed causal association as well as colocalization with CAD only of *PSRC1* expressed in whole blood at the shared causal variant rs7528419, indicating therapeutic potential for CAD. We also found that four out of nine *PSRC1*‐associated circulating proteins, including *CTRP1*, *GRNs*, *Pla2g12b*, *and Neo1*, were also causally associated with CAD risk, all showing strong colocalization at rs7528419 or its proxy SNPs. Taken together, this study provided consistent evidence from multi‐omics layers of data that strongly support *PSRC1* gene as a potential drug target at medium HDL and with therapeutic potential for CAD, as well as mechanistic clues in terms of its downstream proteins underlying such therapeutic potential. However, similar therapeutic potential was not observed for IS.

The athero‐protective role of HDL‐c has been established by ample evidence from observational studies.^[^ ^36^ ^]^ However, MR studies did not support the negative association between HDL‐c and ASCVD risk to be causal,^[^ ^37^ ^]^ and drugs targeted at raising HDL‐c levels have failed to mitigate atherosclerotic risk.^[^ ^38^ ^]^ HDL particles vary in characteristics such as size and lipid content, with different functions,^[^ ^39^ ^]^ and the assumption arose that it might be the functionality of HDL particles with certain characteristics rather than total HDL‐c level responsible for the anti‐atherosclerosis property.^[^ ^40^ ^]^ In line with such assumption, observational studies did reveal associations of HDL particles with CVD event risk varying with particle size, but with controversies that some studies found that larger HDL showed positive associations with CVD mortality and heart failure, and smaller HDL usually showed negative associations,^[^ ^17^, ^18^ ^]^ whereas others showed opposite results,^[^ ^19^, ^20^, ^21^ ^]^ but medium HDL was consistently reported to be negatively associated with cIMT‐assessed atherosclerosis,^[^ ^41^ ^]^ CVD risk,^[^ ^14^ ^]^ and cardiac vascular events.^[^ ^15^, ^16^ ^]^ In addition, a recent MR study by Prats‐Uribe A et al.^[^ ^22^ ^]^ reported that cholesterol and triglyceride content in very large HDL were positively associated with CAD risk, whereas the cholesterol content in medium HDL showed an inverse casual association. In our study, we obtained evidence from observational and genetic associations that consistently identified medium HDL, particularly the cholesteryl ester content within cholesterols, causally protective of atherosclerosis and further ASCVD, in line with Prats‐Uribe A et al.’s study. It has been acknowledged that reverse cholesterol transport constitutes the major mechanism underlying HDL's anti‐atherosclerosis property,^[^ ^4^ ^]^ with Preβ HDL being the major receptor,^[^ ^42^ ^]^ which is primarily remodeled into medium HDL as evidenced by both in vivo and in vitro studies,^[^ ^43^, ^44^ ^]^ supporting our finding of medium HDL causally protective of atherosclerosis. As for cholesteryl esters, the lipid class accounting for 30%–40% of lipids within HDL,^[^ ^45^ ^]^ studies of human subjects with heterozygous cholesteryl ester transfer protein (CETP) deficiency^[^ ^46^ ^]^ and those treated with CETP inhibitor^[^ ^47^ ^]^ both showed reduced atherosclerotic risk, while CETP was exactly the protein that transported cholesteryl esters away from HDL particles. Nevertheless, the mechanisms by which medium HDL and its lipid content exert anti‐atherosclerosis effects were not yet fully understood and warranted further investigations.

The most vital finding of this study is the identification of the *PSRC1* gene targeting medium HDL also having therapeutic potential for CAD, supported by consistent evidence from multi‐omics layers of data. *PSRC1* encodes the proline/serine‐rich coiled‐coil protein 1, which recruits microtubule depolymerases that destabilize microtubules.^[^ ^48^ ^]^ In fact, *PSRC1* has been identified as one of the top‐tier causal genes prioritized as potential therapeutic targets for dyslipidemia through multi‐omics and multi‐trait analyses.^[^ ^23^ ^]^ Our study extended their finding in that we provided strong evidence from transcriptional and proteomic levels supporting the therapeutic potential of *PSRC1* gene for CAD and identified its acting target as medium HDL. Several experimental studies with apoE‐/‐ mice found that *PSRC1* overexpression could inhibit the accumulation of cholesteryl ester in foam cells and alleviate the development of atherosclerosis by raising plasma HDL‐c levels and enhancing HDL function,^[^ ^49^ ^]^ while *PSRC1* deficiency could increase trimethylamine N‐oxide (TMAO) production through manipulating gut microbiota^[^ ^50^ ^]^ and impair cholesterol transport by activation of sulfotransferase 2B1b,^[^ ^51^ ^]^ thereby accelerating atherosclerosis, which supported our findings that *PSRC1* overexpression had therapeutic potential for CAD. Of note, no adverse effects were reported in the study of animal models with *PSRC1* overexpression, consistent with our observation of favorable causal association of *PSRC1* expression with clinical lipids, and null association with CRP, suggesting minimal possibility of adverse side effects. Considering the substantial residual risk for recurrent events in LDL‐c‐lowering therapy, which remains the cornerstone for prevention and treatment of ASCVD currently,^[^ ^5^ ^]^ our identification of *PSRC1* targeting medium HDL with therapeutic potential for CAD might bring new opportunity to tackle such residual risk issue and optimize long‐term lipid‐modifying therapies a step further. Further studies were needed to scrutinize whether such therapeutic potential can be translated into clinical use.

In this study, we had another important finding that four out of nine *PSRC1*‐associated circulating proteins were also causally associated with CAD risk, and all showed strong colocalization at rs7528419 or its proxy SNPs, exactly the shared causal variant between *PSRC1* gene and CAD. This finding supported the therapeutic potential of *PSRC1* for CAD from an independent perspective and also suggested mechanisms through which the *PSRC1* gene exerted its therapeutic potential. Among these four proteins, the one showing negative marginal associations with CAD risk, *Neo1*, was in the same direction as *PSRC1* expression level in whole blood in terms of association with rs7528419, while the remaining three ones with positive associations, including *CTRP1*, *GRNs*, and *Pla2g12b*, were in opposite direction. Therefore, we suspected that the therapeutic potential on CAD of *PSRC1* gene overexpression might be through upregulation of *Neo1* and downregulation of the others. These proteins all have atherosclerosis‐related relevance. *Neo1* has anti‐inflammatory properties, and its deficiency increased the extent of myocardial infarction, elevated inflammation, and aggravated left ventricular dysfunction through regulating macrophage phenotypes and functions via the JAK1‐STAT1 signaling pathway.^[^ ^52^ ^]^ *CTRP1* has been reported to be involved in atherogenesis through induction of adhesion molecules.^[^ ^53^, ^54^, ^55^ ^]^ *GRNs* were pro‐atherogenic due to their pro‐inflammatory characteristics; Kojima Y et al.^[^ ^56^ ^]^ has observed that *GRNs* increased interleukin‐8 production, a crucial cytokine involved in the development of atherosclerosis from human aortic smooth muscle cells,^[^ ^57^ ^]^ while inhibition of the conversion of progranulin to proinflammatory *GRNs* contributed to the anti‐inflammatory effects of HDL.^[^ ^58^ ^]^ *Pla2g12b* could facilitate the transfer of triglycerides to nascent ApoB‐containing lipoproteins to generate triglyceride‐rich lipoproteins, which promoted the formation of atherosclerotic plaques.^[^ ^59^, ^60^ ^]^ Taken together, *PSRC1* expression may affect the development and progression of CAD at least partly through regulation of the aforementioned proteins, and further investigations into the specific mechanisms would provide a broader perspective on the possibility of realization of *PSRC1*’s therapeutic potential in clinical settings.

There were several strengths of the present study. First, we obtained evidence of observational and genetic associations which consistently identified lipid content in HDL subclasses, particularly medium HDL and cholesteryl ester content, casually protective of atherosclerosis. Second, we performed multi‐omics integrative analyses which yielded consistent evidence that strongly supported *PSRC1* gene with therapeutic potential for CAD. Third, integration of pQTL data provided mechanistic clues for the therapeutic potential of *PSRC1*.

However, we also acknowledge several limitations in this study. First, the number of available IVs in MR analyses of gene expression levels was limited, all less than 10, which might have compromised the confidence of MR results. Nevertheless, strong F statistics of these IVs, ranging from 30.17 to 35 167.34, indicated that the possibility of weak instrument bias was negligible, and the identification of *PSRC1* gene with therapeutic potential for CAD was further supported by evidence from its downstream proteins. Second, we did not observe similar therapeutic potential of *PSRC1* on IS, and could not rule out the possibility that the absence of such causal association might be owing to the lack of transcriptome data from more relevant tissues such as cerebral arteries, and the limited sample size of gene expression data. However, previous GWA studies have reported risk loci of CAD at 1p13.3 near *PSRC1*, but no significant association was observed at this locus with IS risk,^[^ ^61^, ^62^, ^63^, ^64^ ^]^ suggesting that the absence of similar therapeutic potential of *PSRC1* for IS as for CAD might be more likely owing to their difference in pathological mechanisms. Third, although preliminary mechanistic clues of *PSRC1*’s therapeutic potential for CAD were yielded from proteomic analysis, the exact mechanisms underlying such potential were yet to be elucidated and needed further investigations using in vivo and in vitro experimental models. Fourth, the results of this study were derived from study samples predominantly comprised of Europeans, and further replication was needed to determine whether our findings could be extrapolated to non‐European populations.

## 4 Conclusion

In the present study, we identified lipid content in HDL particles, particularly medium HDL and cholesteryl ester content, causally protective of atherosclerosis through consistent evidence from observational and genetic associations. We also obtained evidence from multi‐omics layers of data which strongly supported *PSRC1* with therapeutic potential for CAD, and also provided mechanistic clues of such therapeutic potential through its downstream proteins. Further studies are warranted to examine whether the therapeutic potential of *PSRC1* can be translated into clinical use.

## 5 Experimental Section

### NMR‐Based (Apo)Lipoprotein Profiling

The UKB study was launched from 2006 to 2010, and enrolled over 500 000 participants aged 40–69 across the United Kingdom, among whom ≈95% were of European ancestry, and the study design has been previously reported in detail.^[^ ^65^ ^]^ All participants have given their informed consent, and the study was approved by the North West National Health Service Research Ethics Committee ^(Ref 21/NW/0157)^. The present study was conducted under the UKB application project 88159.

(Apo)lipoprotein profiling of baseline plasma samples was performed with the Nightingale NMR spectroscopy,^[^ ^66^ ^]^ producing absolute quantification measurements of 131 (apo)lipoprotein measurements, among a random sample of 118 019 participants from the initial cohort in phase I data release, and an additional 156 336 participants in phase II. Details of the NMR platform, laboratory measurement, and quality control procedures have been described in detail previously.^[^ ^67^ ^]^ In brief, two blind duplicate samples provided by UKB, and two internal control samples provided by Nightingale Health were included in each 96‐well plate for tracking the consistency across multiple spectrometers during the project. Data on levels of cholesteryl esters in medium HDL was used as an example to show quality control data in Figure S17 (Supporting Information) (data from https://biobank.ndph.ox.ac.uk/showcase/label.cgi?id=220), which demonstrated good agreement of the measurement distribution from fourteen consecutively measured sample batches (Figure S17a, Supporting Information) and between spectrometers (Figure S17b, Supporting Information). Consistency of the blind duplicate measures (*r* ^2^ = 0.89; Figure S17c, Supporting Information) and biological stability of the biomarker measures over time between the samples from baseline and repeated assessments (*r* = 0.69; Figure S17d, Supporting Information) were also presented. Considering the high repeatability of data over time, no adjustment for batch effect was performed. For missing values (degree of missingness <1%), samples marked as zero concentration value were replaced with the values just below the lowest observed value to avoid artificial drops in the distributions, as was recommended by Julkunen et al.^[^ ^67^ ^]^ for handling NMR‐derived data in UKB study. Details of all (apo)lipoproteins are shown in Table S23 (Supporting Information).

### Carotid Ultrasound Assessment

The assessment for cIMT was performed with carotid ultrasound at the UKB Imaging Assessment Center in 2014 according to a standard protocol (https://biobank.ndph.ox.ac.uk/showcase/refer.cgi?id=511) and was available for 50 115 participants to date. Mean cIMT was used in this study as an assessment of whole‐body atherosclerosis burden.^[^ ^26^ ^]^ The time window between baseline blood sampling and cIMT measurement was 9.09 ± 2.02 years.

### Statistical Analysis

The observational and genetic association of plasma (apo)lipoproteins was examined with cIMT‐assessed atherosclerosis to identify plasma (apo)lipoproteins causally associated with cIMT‐assessed atherosclerosis among 26 091 participants who had both NMR‐measured (apo)lipoprotein profile data and cIMT assessment, and were without CVD at baseline, including 10 375 participants and 15 716 participants within phases I and II releases of NMR data, respectively. The baseline characteristics for participants from the two phases of data releases were described as mean ± SD for continuous variables and as prevalence (%) for categorical variables. Multivariate linear regression was first conducted separately within participants from the two phases of data adjusting for sex, age, ethnicity, BMI, SBP, DBP, smoking status, drinking status, physical activity level, diet, prevalent diabetes, use of cholesterol‐lowering medications, family history of CVD, and time‐window between lipoprotein and cIMT measurements. For adjustment of diet, principal component analysis of dietary data collected from a 29‐item food frequency questionnaire was performed according to the study of Schweren et al.,^[^ ^68^ ^]^ and we extracted the first four principal components with eigenvalues ≥1 as covariates representing overall dietary intake, which explained 51% of total variance in dietary data (Table S24, Supporting Information). The results from two phases of data were examined for consistency and then meta‐analyzed. Considering the time window between baseline blood sampling and measurement of cIMT, which might introduce unanticipated confounding, LDSC analysis was then performed,^[^ ^28^ ^]^ which assesses the degree of shared genetic architecture between two phenotypes independent of environmental confounders, with estimates ranging from −1 for a negative correlation to 1 for a positive correlation. MR analysis was further performed between lipoprotein measurements and cIMT to examine whether the associations were causal. GWAS summary statistics for (apo)lipoprotein measurements were available via the IEU OPEN GWAS project website (https://gwas.mrcieu.ac.uk/datasets/), comprising 115 078 individuals from UKB. Summary statistics for mean cIMT were derived from Strawbridge et al.^[^ ^69^ ^]^ (https://researchdata.gla.ac.uk/1170/), comprising 22 179 UKB participants. Considering an overlap rate no more than 10%, the two‐sample MR approach was used in this analysis, and then calculated the genetic effect bias resulting from sample overlap with a web application tool.^[^ ^70^ ^]^ SNPs that were not in LD (clumping *r* ^2^ threshold = 0.001 and window size = 10 000 kb) and were associated with lipoprotein measurements at genome‐wide significance (*P* < 5 ×10^−8^) were used as genetic instrument variables (IVs). The strength of IVs was assessed with F statistics [F  =  R^2^ ×(N−2)/(1−R^2^), R^2^ = 2 × MAF × (1−MAF) × beta^2^],^[^ ^71^ ^]^ and IVs with F‐statistic <10 were removed.^[^ ^31^ ^]^ In this study, fixed effects or multiplicative random effects mode of IVW was used as the main method, according to the existence of heterogeneity between IVs,^[^ ^29^ ^]^ with a series of additional sensitivity analyses. Specifically, MR‐Egger analysis was performed to calculate the intercept statistic to assess pleiotropy, Cochran's Q test to assess heterogeneity between IVs, and radial IVW to identify potential outlier IVs.^[^ ^72^ ^]^ In addition, weighted median method was conducted to control horizontal pleiotropy, and leave‐one‐out method to evaluate the impact of potentially pleiotropic SNPs on causal estimates. If there was significant heterogeneity (*p* <0.05), the random effect mode of IVW was reported; otherwise, the fixed effect mode of IVW was reported.^[^ ^73^ ^]^ The analysis was first conducted with no adjustment (Model 1) and followed with sensitivity MVMR analyses by additional adjustment for smoking and alcohol drinking (Model 2), the two most heritable behavior risk factors of ASCVD,^[^ ^32^, ^33^ ^]^ and LDL‐c, the currently recommended primary target for lipid management in prevention and treatment of ASCVD.^[^ ^3^ ^]^ MVMR analysis was enabled by incorporating genetic instruments for each risk factor into the same model,^[^ ^74^ ^]^ and genetic instruments for smoking, alcohol drinking, and LDL‐c were derived from the studies by Liu et al.^[^ ^75^ ^]^ and Graham et al.,^[^ ^76^ ^]^ respectively. All MR analysis in this study was conducted following the STROBE‐MR Guideline (Table S25, Supporting Information).^[^ ^77^ ^]^

To examine the causality of association and shared causal genetic variants of (apo)lipoproteins with the two ASCVD subtypes, observational association analysis, two‐sample MR analysis, and colocalization analysis of atherosclerosis‐associated (apo)lipoproteins with CAD and IS were further performed. Specifically, for (apo)lipoproteins causally associated with mean cIMT, observational association analysis was first performed among a total of 252 876 participants with (apo)lipoprotein profile data and without CVD at baseline in the UKB study, including 108 899 participants and 143 980 participants in phases I and II releases of data, respectively, among whom 13 123 developed CAD during a follow‐up period of 15.10 ± 2.65 years, and 5 015 developed IS during a follow‐up period of 15.55 ± 1.63 years. The study sample covered that in a previous study on (apo)lipoprotein profile and CHD risk based on the phase I release of data (N  =  89 422).^[^ ^13^ ^]^ Cox proportional hazards regression was conducted separately within the two phases of data adjusting for sex, age, ethnicity, BMI, SBP, DBP, smoking status, drinking status, physical activity level, diet, prevalent diabetes, use of cholesterol‐lowering medications, family history of CVD, LDL‐c, HDL‐c, and TG, and then meta‐analyzed. Further, two‐sample MR analysis was conducted to examine causal associations of (apo)lipoproteins with CAD and IS risk. GWAS summary statistics for (apo)lipoprotein measurements were from the UKB study as aforementioned, which had a sample size (N = 115 078) ≈5 times as large as the previous MR study on lipoprotein characteristics and CAD risk based on data from Kettunen et al. (N  =  24 925).^[^ ^22^ ^]^ GWAS summary statistics for CAD were derived from Aragam et al.^[^ ^34^ ^]^ (https://www.ebi.ac.uk/gwas/studies/GCST90132314), comprising 181 522 cases and 984 168 controls, >95% of them being of European ancestry. GWAS summary statistics for IS were derived from Malik et al.^[^ ^64^ ^]^ (https://gwas.mrcieu.ac.uk/datasets/ebi‐a‐GCST005838/), comprising 446 696 European individuals of 40 585 cases and 406 111 controls. MR analysis and MVMR analysis were conducted in the same way as described above. Moreover, an additional two‐step mediation analysis with MR^[^ ^78^ ^]^ was performed to examine to which extent the effect of (apo)lipoproteins on CAD/IS went through atherosclerosis. First, causal effect of each (apo)lipoprotein on mean cIMT was assessed (β_1_), and then causal effects of mean cIMT on CAD and IS, respectively, were also assessed (β_2_CAD_ and β_2_IS_). The indirect effect was derived from multiplying β_1_ and β_2_, and the proportion of mediation was equal to the indirect effect divided by the total effect (β of the lipoprotein‐CAD and lipoprotein‐IS associations, respectively). The *delta* method was utilized to estimate the confidence intervals, and the *pnorm* method was used to estimate the *p‐*value.^[^ ^79^ ^]^ Further, colocalization analysis^[^ ^30^ ^]^ was further conducted to identify the causal SNP shared by (apo)lipoprotein measurements and each of the two ASCVD subtypes. IVs for lipoproteins were designated as lead SNPs, and all SNPs within a 1000 kb window from GWAS of (apo)lipoproteins and the disease outcome (CAD or IS) were included in the colocalization analysis, with posterior probabilities (PPs) of five hypotheses tested: PP.H0, no association with either trait; PP.H1, association with (apo)lipoprotein measurements but not with CAD or IS risk; PP.H2, association with CAD or IS risk but not with (apo)lipoprotein measurements; PP.H3, association with CAD or IS risk as well as (apo)lipoprotein measurements, but at distinct causal variants; and PP.H4, association with both traits, with a shared causal variant. A PP.H4 >0.8 indicates significant colocalization.

In search of potential drug targets for CAD and IS, MR and colocalization analyses were further performed through integration of eQTL and pQTL data. Specifically, transcriptome‐wide search was performed for genes with expression levels cis‐associated with colocalized SNPs between (apo)lipoproteins and CAD or IS in whole blood against the eQTLGen database (N = 31 684; https://eqtlgen.org/) and in artery tissue (coronary [N = 213], aorta [N = 387], and tibial [N = 584] artery), heart tissue (atrial appendage [N = 372] and heart left ventricle [N = 386]), and liver tissue (N = 208) against the GTEx Project Analysis V8 database (https://www.gtexportal.org/). Further MR analysis and colocalization analysis were performed of these genes against CAD and IS as aforementioned, except that the MR PRESSO method was used instead of the radial IVW method to identify and remove potential outliers due to the limited number of available IVs for gene expression levels, and under the circumstance of only one IV being available, the Wald ratio method^[^ ^80^ ^]^ was used. Based on the assumption that the pleiotropic variants/genes that control both lipoproteins and disease traits are more likely to serve therapeutic purposes,^[^ ^23^ ^]^ genes that showed causal association with CAD or IS and also colocalized with the corresponding disease trait with a shared causal genetic variant were considered as potential drug targets for disease treatment. After the identification of the gene target, a search of circulating proteins *cis*‐associated with the shared causal variant against data from a recently published GWAS of human plasma proteome was then performed,^[^ ^35^ ^]^ and further examined the causal association and colocalization of these proteins with the corresponding disease trait to scrutinize the therapeutic potential of the gene target from the perspective of its downstream proteins, which might reveal mechanistic clues of such therapeutic potential.

Analyses were performed with R (version 4.3.0) using packages “stats” for linear regression analysis, “survival” for COX regression analysis, “TwoSampleMR” for two‐sample MR analysis, and “coloc” for localization analysis, while the “LDSC” command line tool supported by Ubuntu Linux 22.04 was used for LDSC analysis. *FDR* <0.05 was considered as significant and calculated by the Benjamini‐Hochberg method, and a *p*‐value <0.05 was regarded as nominally significant. Of note, a looser criterion of nominal significance was employed at first in observational and genetic association analyses between (apo)lipoprotein profile and cIMT‐assessed atherosclerosis, in order to include as many as possible atherosclerosis‐associated (apo)lipoproteins into the following evaluation of association with and therapeutic potential for ASCVD.

## Conflict of Interest

The authors declare no conflict of interest.

## Supporting information



Supporting Information

## Data Availability

The data that support the findings of this study are available from the corresponding author upon reasonable request.
