# Supplementary material for: (Apo)Lipoprotein Profiling with Multi‐Omics Analysis Identified Medium‐HDL‐Targeting PSRC1 with Therapeutic Potential for Coronary Artery Disease
Source: Adv Sci (Weinh). 2025 Feb 22;12(15):2413491. doi: 10.1002/advs.202413491 (PMC12005818; doi:10.1002/advs.202413491)
Supplement: Supplementary file 1 — Supporting Information [file ADVS-12-2413491-s001.pdf]

ADVANCED SCIENCE
Open Access

Supporting Information

for *Adv. Sci.*, DOI 10.1002/advs.202413491

(Apo)Lipoprotein Profiling with Multi-Omics Analysis Identified Medium-HDL-Targeting *PSRC1* with Therapeutic Potential for Coronary Artery Disease

Yingmei Li, Sihan Wang, Ling Liu, Hao Cai, Yacan Huang, Mingjing Gao, Xiaogang Zhang, Qingqing Wu and Gaokun Qiu**

Supporting Information

(Apo)lipoprotein profiling with multi-omics analysis identified medium-HDL-targeting *PSRC1* with therapeutic potential for coronary artery disease

Yingmei Li,[1] Sihan Wang,[1] Ling Liu,[1] Hao Cai,[1] Yacan Huang,[1] Mingjing Gao,[1] Xiaogang Zhang,[4]

Qingqing Wu,[2,3] Gaokun Qiu[1*]*

[1]Ministry of Education and State Key Laboratory of Environmental Health (Incubating), School of Public Health, Tongji Medical College, Huazhong University of Science and Technology, Wuhan 430030, China.

[2]Department of Cardiology, Zhongnan Hospital of Wuhan University, Wuhan 430062, China.

[3]Institute of Myocardial Injury and Repair, Wuhan University, Wuhan 430062, China.

[4]SCIEX Application Support Center, Shanghai 200050, China.

Table S1. Observational association between (apo)lipoprotein profile and mean carotid intima-media thickness (cIMT).

Table S2. Genetic correlation between (apo)lipoprotein profile and mean cIMT from LDSC regression analysis.

Table S3. Genetic instruments for (apo)lipoprotein profile in two-sample Mendelian randomization (MR) analysis.

Table S4. Causal associations between (apo)lipoprotein profile and mean cIMT from two-sample MR analysis.

Table S5. Cochran Q and MR Egger pleiotropy tests of MR analysis between (apo)lipoprotein profile and mean cIMT.

Table S6. Genetic instruments for HDL subclasses in two-sample MR analysis.

Table S7. Causal associations between HDL subclasses and CAD and IS.

Table S8. Cochran Q and MR Egger pleiotropy tests of MR analysis of HDL subclasses with CAD and IS.

Table S9. Univariate and multivariate Causal associations of HDL subclasses with CAD and IS.

Table S10. Colocalization of HDL subclasses with CAD and IS.

Table S11. Circulating genes associated with shared genetic variant between HDL subclasses and CAD/IS retrieved from eQTLGen database.

Table S12. Genetic instruments for circulating gene expression levels in two-sample MR analysis.

Table S13. Causal associations of circulating genes with CAD and IS.

Table S14. Cochran Q and MR Egger pleiotropy tests of MR analysis between circulating genes and CAD/IS.

Table S15. Colocalization between circulating genes and CAD/IS.

Table S16. Causal associations of multi-tissue gene expression levels with CAD and IS using data from GTEx project.

Table S17. Colocalization between multi-tissue gene expression levels and CAD/IS.

Table S18. Causal associations of circulating *PSRC1* expression with traditional circulating lipids and C-reactive protein (CRP).

Table S19. The nine *PSRC1*-associated circulating proteins.

Table S20. Genetic instruments for *PSRC1*-associated circulating proteins in two-sample MR analysis.

Table S21. Causal associations of *PSRC1*-associated circulating proteins with CAD.

Table S22. Colocalization between *PSRC1*-associated circulating proteins and CAD.

Table S23. The 131 (apo)lipoprotein measurements examined in this study.

Table S24. Food loadings (≤ -0.3 or ≥ 0.3) for principal components 1 to 4 (PC1 to PC4).

Table S25. STROBE-MR checklist table.

Figure S1. Bias and type 1 error rate for MR analysis of Total_P with mean cIMT

Figure S2. Bias and type 1 error rate for MR analysis of HDL_P with mean cIMT.

Figure S3. Bias and type 1 error rate for MR analysis of M_HDL_P with mean cIMT.

Figure S4. Bias and type 1 error rate for MR analysis of HDL_L with mean cIMT.

Figure S5. Bias and type 1 error rate for MR analysis of M_HDL_L with mean cIMT.

Figure S6. Bias and type 1 error rate for MR analysis of L_HDL_L with mean cIMT.

Figure S7. Bias and type 1 error rate for MR analysis of HDL_C with mean cIMT.

Figure S8. Bias and type 1 error rate for MR analysis of M_HDL_C with mean cIMT.

Figure S9. Bias and type 1 error rate for MR analysis of M_HDL_FC with mean cIMT.

Figure S10. Bias and type 1 error rate for MR analysis of HDL_CE with mean cIMT.

Figure S11. Bias and type 1 error rate for MR analysis of M_HDL_CE with mean cIMT.

Figure S12. Bias and type 1 error rate for MR analysis of L_HDL_CE with mean cIMT.

Figure S13. Bias and type 1 error rate for MR analysis of HDL_PL with mean cIMT.

Figure S14. Bias and type 1 error rate for MR analysis of L_HDL_PL with mean cIMT.

Figure S15. Bias and type 1 error rate for MR analysis of HDL_size with mean cIMT.

Figure S16. Bias and type 1 error rate for MR analysis of ApoA1 with mean cIMT.

Figure S17. Technical and biological repeatability for measurement of cholesteryl esters in medium HDL.

Table S1. Observational association between (apo)lipoprotein profile and mean carotid intima-media thickness (cIMT).

(apo)lipoprotein profile	Phase I		Phase II		Meta-analysis				
	β（95%CI)	*pval*	β（95%CI)	*pval*	β（95%CI)	*pval*	FDR	$P_{heterogeneity}$	I^2
Total Concentration of Lipoprotein Particles	-2.41 (-4.75, -0.08)	4.29E-02	-0.99 (-2.82, 0.84)	2.91E-01	-1.53 (-2.97, -0.09)	0.0374	0.0497	0.3471	0.00%
Total Concentration of VLDL Particles	3.18 (2.1, 4.27)	9.67E-09	2.52 (1.66, 3.39)	9.78E-09	2.78 (2.10, 3.45)	<.0001	0.0002	0.3526	0.00%
Total Concentration of Very Small VLDL Particles	4.98 (3.62, 6.35)	9.02E-13	4.08 (3, 5.16)	1.26E-13	4.43 (3.57, 5.29)	<.0001	0.0002	0.3084	3.62%
Total Concentration of Small VLDL Particles	2.45 (1.45, 3.46)	1.88E-06	2.01 (1.21, 2.8)	7.30E-07	2.18 (1.56, 2.80)	<.0001	0.0002	0.4974	0.00%
Total Concentration of Medium VLDL Particles	2.71 (1.79, 3.63)	8.92E-09	2.19 (1.45, 2.93)	6.31E-09	2.39 (1.82, 2.97)	<.0001	0.0002	0.3924	0.00%
Total Concentration of Large VLDL Particles	0.88 (0.29, 1.47)	3.44E-03	0.62 (0.14, 1.09)	1.13E-02	0.72 (0.35, 1.09)	0.0001	0.0002	0.4925	0.00%
Total Concentration of Very Large VLDL Particles	0.45 (0.02, 0.89)	4.13E-02	0.3 (-0.05, 0.64)	8.94E-02	0.36 (0.09, 0.63)	0.0093	0.0144	0.5901	0.00%
Total Concentration of Chylomicrons and Extremely Large VLDL Particles	0.01 (-0.26, 0.28)	9.21E-01	-0.13 (-0.34, 0.07)	1.97E-01	-0.08 (-0.24, 0.08)	0.3323	0.3796	0.3911	0.00%
Total Concentration of IDL Particles	5.87 (4.46, 7.28)	3.75E-16	4.79 (3.65, 5.92)	2.22E-16	5.24 (4.19, 6.29)	<.0001	0.0002	0.2407	27.36%
Total Concentration of LDL Particles	5.37 (3.92, 6.81)	3.73E-13	4.92 (3.78, 6.06)	2.22E-16	5.09 (4.20, 5.99)	<.0001	0.0002	0.6339	0.00%
Total Concentration of Small LDL Particles	5.91 (4.37, 7.46)	6.03E-14	5.14 (3.92, 6.36)	2.22E-16	5.44 (4.48, 6.39)	<.0001	0.0002	0.4388	0.00%
Total Concentration of Medium LDL Particles	4.04 (2.75, 5.33)	9.09E-10	3.69 (2.68, 4.71)	9.34E-13	3.82 (3.03, 4.62)	<.0001	0.0002	0.682	0.00%
Total Concentration of Large LDL Particles	5.34 (3.92, 6.76)	1.95E-13	4.98 (3.85, 6.11)	2.22E-16	5.12 (4.23, 6.00)	<.0001	0.0002	0.6937	0.00%
Total Concentration of HDL Particles	-3.84 (-6.04, -1.63)	6.68E-04	-2.19 (-3.92, -0.46)	1.32E-02	-2.86 (-4.45, -1.28)	0.0004	0.0008	0.2499	24.46%
Total Concentration of Small HDL Particles	-2.66 (-5.06, -0.25)	3.02E-02	-1.12 (-3.01, 0.77)	2.44E-01	-1.71 (-3.20, -0.22)	0.024	0.0343	0.3257	0.00%
Total Concentration of Medium HDL Particles	-3.73 (-5.21, -2.24)	8.32E-07	-2.56 (-3.73, -1.39)	1.76E-05	-3.05 (-4.18, -1.92)	<.0001	0.0002	0.2264	31.67%
Total Concentration of Large HDL Particles	-0.7 (-1.37, -0.04)	3.81E-02	-0.18 (-0.72, 0.37)	5.21E-01	-0.41 (-0.92, 0.11)	0.1196	0.1527	0.231	30.29%
Total Concentration of Very Large HDL Particles	1.26 (0.29, 2.23)	1.08E-02	1.58 (0.81, 2.36)	6.51E-05	1.46 (0.85, 2.06)	<.0001	0.0002	0.6133	0.00%
Total Lipids in Lipoprotein Particles	4.15 (2.34, 5.96)	6.88E-06	3.82 (2.38, 5.26)	2.14E-07	3.95 (2.82, 5.08)	<.0001	0.0002	0.7791	0.00%
Total Lipids in VLDL	1.69 (0.86, 2.52)	6.78E-05	1.21 (0.54, 1.88)	3.81E-04	1.40 (0.88, 1.92)	<.0001	0.0002	0.3822	0.00%
Total Lipids in Very Small VLDL	4.71 (3.33, 6.09)	2.63E-11	3.81 (2.72, 4.89)	5.20E-12	4.15 (3.29, 5.01)	<.0001	0.0002	0.3122	2.07%
Total Lipids in Small VLDL	2.51 (1.48, 3.55)	2.05E-06	2.13 (1.3, 2.95)	4.22E-07	2.28 (1.63, 2.92)	<.0001	0.0002	0.5678	0.00%
Total Lipids in Medium VLDL	2.43 (1.52, 3.34)	1.69E-07	1.98 (1.24, 2.71)	1.24E-07	2.16 (1.58, 2.73)	<.0001	0.0002	0.4454	0.00%
Total Lipids in Large VLDL	0.91 (0.31, 1.51)	3.07E-03	0.63 (0.14, 1.11)	1.14E-02	0.74 (0.36, 1.11)	0.0001	0.0002	0.4711	0.00%
Total Lipids in Very Large VLDL	0.52 (0.06, 0.97)	2.66E-02	0.31 (-0.06, 0.68)	9.75E-02	0.39 (0.11, 0.68)	0.0073	0.0115	0.494	0.00%

Total Lipids in Chylomicrons and Extremely Large VLDL	0.05 (-0.27, 0.38)	7.41E-01	-0.24 (-0.49, 0)	5.19E-02	-0.11 (-0.40, 0.18)	0.4436	0.4866	0.1479	52.25%
Total Lipids in IDL	5.42 (3.92, 6.92)	1.60E-12	5.02 (3.83, 6.22)	2.22E-16	5.18 (4.24, 6.11)	<.0001	0.0002	0.687	0.00%
Total Lipids in LDL	4.61 (3.23, 5.99)	6.05E-11	4.54 (3.44, 5.63)	5.48E-16	4.56 (3.71, 5.42)	<.0001	0.0002	0.9375	0.00%
Total Lipids in Small LDL	5.4 (3.94, 6.86)	4.56E-13	5.09 (3.93, 6.25)	2.22E-16	5.21 (4.30, 6.12)	<.0001	0.0002	0.7443	0.00%
Total Lipids in Medium LDL	3.71 (2.49, 4.93)	2.36E-09	3.7 (2.73, 4.67)	7.89E-14	3.70 (2.94, 4.46)	<.0001	0.0002	0.9897	0.00%
Total Lipids in Large LDL	4.68 (3.28, 6.09)	6.91E-11	4.63 (3.51, 5.74)	4.94E-16	4.65 (3.77, 5.52)	<.0001	0.0002	0.9511	0.00%
Total Lipids in HDL	-3.28 (-5.05, -1.51)	2.84E-04	-2.05 (-3.46, -0.64)	4.36E-03	-2.54 (-3.72, -1.36)	<.0001	0.0002	0.2869	11.83%
Total Lipids in Small HDL	-3.72 (-6.14, -1.3)	2.61E-03	-2.22 (-4.13, -0.32)	2.23E-02	-2.80 (-4.30, -1.30)	0.0003	0.0006	0.3413	0.00%
Total Lipids in Medium HDL	-4.34 (-5.97, -2.7)	1.95E-07	-3.01 (-4.29, -1.72)	4.75E-06	-3.57 (-4.86, -2.28)	<.0001	0.0002	0.2094	36.52%
Total Lipids in Large HDL	-1.04 (-1.8, -0.28)	7.53E-03	-0.44 (-1.06, 0.18)	1.64E-01	-0.70 (-1.28, -0.12)	0.0185	0.0269	0.2329	29.73%
Total Lipids in Very Large HDL	0.69 (-0.13, 1.51)	9.72E-02	1.01 (0.37, 1.65)	1.87E-03	0.89 (0.39, 1.39)	0.0005	0.001	0.5455	0.00%
Total Cholesterol	4.69 (2.98, 6.39)	7.46E-08	4.55 (3.19, 5.91)	5.67E-11	4.60 (3.54, 5.67)	<.0001	0.0002	0.9007	0.00%
VLDL Cholesterol	2.94 (1.98, 3.9)	2.25E-09	2.33 (1.57, 3.1)	2.70E-09	2.57 (1.97, 3.17)	<.0001	0.0002	0.3351	0.00%
Total Cholesterol in Very Small VLDL	4.97 (3.65, 6.28)	1.34E-13	4.06 (3.03, 5.09)	1.05E-14	4.42 (3.55, 5.28)	<.0001	0.0002	0.2884	11.26%
Total Cholesterol in Small VLDL	3.21 (2.21, 4.2)	2.51E-10	2.72 (1.93, 3.51)	1.56E-11	2.91 (2.29, 3.53)	<.0001	0.0002	0.4512	0.00%
Total Cholesterol in Medium VLDL	2.97 (2.14, 3.8)	2.56E-12	2.51 (1.84, 3.18)	2.11E-13	2.69 (2.17, 3.21)	<.0001	0.0002	0.3998	0.00%
Total Cholesterol in Large VLDL	1.14 (0.53, 1.75)	2.65E-04	0.84 (0.36, 1.33)	7.13E-04	0.96 (0.58, 1.34)	<.0001	0.0002	0.4586	0.00%
Total Cholesterol in Very Large VLDL	0.95 (0.4, 1.49)	6.61E-04	0.7 (0.26, 1.14)	1.80E-03	0.80 (0.46, 1.14)	<.0001	0.0002	0.4902	0.00%
Total Cholesterol in Chylomicrons and Extremely Large VLDL	0.09 (-0.28, 0.46)	6.34E-01	-0.09 (-0.39, 0.2)	5.33E-01	-0.02 (-0.25, 0.21)	0.8462	0.8707	0.4468	0.00%
Total Cholesterol in IDL	4.83 (3.46, 6.21)	5.57E-12	4.44 (3.34, 5.54)	2.39E-15	4.59 (3.74, 5.45)	<.0001	0.0002	0.66	0.00%
LDL Cholesterol	4.33 (3.01, 5.64)	1.07E-10	4.27 (3.22, 5.31)	1.15E-15	4.29 (3.47, 5.11)	<.0001	0.0002	0.944	0.00%
Total Cholesterol in Small LDL	4.73 (3.37, 6.09)	9.32E-12	4.47 (3.39, 5.54)	5.13E-16	4.57 (3.72, 5.41)	<.0001	0.0002	0.7636	0.00%
Total Cholesterol in Medium LDL	3.51 (2.34, 4.68)	4.19E-09	3.51 (2.58, 4.44)	1.50E-13	3.51 (2.78, 4.24)	<.0001	0.0002	0.9991	0.00%
Total Cholesterol in Large LDL	4.41 (3.08, 5.75)	9.19E-11	4.35 (3.3, 5.41)	7.66E-16	4.38 (3.55, 5.21)	<.0001	0.0002	0.9472	0.00%
HDL Cholesterol	-2.75 (-4.32, -1.19)	5.73E-04	-1.53 (-2.79, -0.28)	1.66E-02	-2.05 (-3.23, -0.87)	0.0007	0.0013	0.2338	29.47%
Total Cholesterol in Small HDL	-3.12 (-5.49, -0.76)	9.67E-03	-1.31 (-3.15, 0.54)	1.66E-01	-2.06 (-3.81, -0.30)	0.0214	0.0308	0.2355	28.96%
Total Cholesterol in Medium HDL	-3.94 (-5.39, -2.48)	1.14E-07	-2.56 (-3.71, -1.41)	1.33E-05	-3.17 (-4.52, -1.83)	<.0001	0.0002	0.1446	53.01%
Total Cholesterol in Large HDL	-0.71 (-1.34, -0.07)	2.85E-02	-0.19 (-0.7, 0.32)	4.71E-01	-0.41 (-0.92, 0.09)	0.1087	0.1417	0.2108	36.14%

Total Cholesterol in Very Large HDL	1.01 (0.1, 1.92)	3.00E-02	1.41 (0.68, 2.14)	1.58E-04	1.25 (0.68, 1.82)	<.0001	0.0002	0.5047	0.00%
Total Free Cholesterol	4.97 (3.34, 6.59)	2.03E-09	4.65 (3.36, 5.94)	1.84E-12	4.77 (3.76, 5.78)	<.0001	0.0002	0.7643	0.00%
Free Cholesterol in VLDL	2.37 (1.47, 3.26)	2.20E-07	1.81 (1.09, 2.52)	7.40E-07	2.03 (1.47, 2.59)	<.0001	0.0002	0.3393	0.00%
Free Cholesterol in Very Small VLDL	4.91 (3.57, 6.26)	8.89E-13	3.89 (2.85, 4.94)	3.12E-13	4.31 (3.33, 5.29)	<.0001	0.0002	0.2416	27.08%
Free Cholesterol in Small VLDL	3.48 (2.46, 4.51)	2.88E-11	2.98 (2.16, 3.8)	9.41E-13	3.18 (2.54, 3.81)	<.0001	0.0002	0.4492	0.00%
Free Cholesterol in Medium VLDL	2.83 (1.97, 3.69)	1.15E-10	2.34 (1.65, 3.04)	3.44E-11	2.53 (2.00, 3.07)	<.0001	0.0002	0.3873	0.00%
Free Cholesterol in Large VLDL	0.85 (0.29, 1.41)	3.07E-03	0.63 (0.18, 1.08)	6.01E-03	0.72 (0.37, 1.07)	<.0001	0.0002	0.5529	0.00%
Free Cholesterol in Very Large VLDL	0.69 (0.21, 1.18)	5.06E-03	0.41 (0.04, 0.79)	3.20E-02	0.52 (0.22, 0.82)	0.0006	0.0012	0.3751	0.00%
Free Cholesterol in Chylomicrons and Extremely Large VLDL	-0.02 (-0.39, 0.35)	9.34E-01	-0.25 (-0.53, 0.03)	8.13E-02	-0.16 (-0.39, 0.06)	0.151	0.1889	0.3212	0.00%
Free Cholesterol in IDL	4.74 (3.37, 6.11)	1.20E-11	4.33 (3.24, 5.41)	5.82E-15	4.49 (3.64, 5.34)	<.0001	0.0002	0.6388	0.00%
Free Cholesterol in LDL	4.39 (3.11, 5.67)	1.71E-11	4.26 (3.25, 5.27)	2.22E-16	4.31 (3.52, 5.10)	<.0001	0.0002	0.877	0.00%
Free Cholesterol in Small LDL	4.46 (3.23, 5.7)	1.71E-12	4.47 (3.49, 5.44)	2.22E-16	4.47 (3.70, 5.23)	<.0001	0.0002	0.9949	0.00%
Free Cholesterol in Medium LDL	3.84 (2.65, 5.02)	2.31E-10	3.85 (2.92, 4.79)	8.05E-16	3.85 (3.11, 4.58)	<.0001	0.0002	0.9821	0.00%
Free Cholesterol in Large LDL	4.4 (3.11, 5.69)	2.46E-11	4.2 (3.17, 5.22)	8.58E-16	4.27 (3.47, 5.08)	<.0001	0.0002	0.8071	0.00%
Free Cholesterol in HDL	-1.28 (-2.83, 0.27)	1.05E-01	-0.23 (-1.48, 1.01)	7.14E-01	-0.65 (-1.66, 0.36)	0.2046	0.2426	0.3011	6.48%
Free Cholesterol in Small HDL	-0.6 (-2.96, 1.76)	6.19E-01	0.85 (-0.99, 2.7)	3.65E-01	0.30 (-1.15, 1.75)	0.6835	0.7151	0.342	0.00%
Free Cholesterol in Medium HDL	-2.46 (-3.72, -1.2)	1.26E-04	-1.42 (-2.42, -0.41)	5.76E-03	-1.87 (-2.88, -0.85)	0.0003	0.0006	0.2032	38.24%
Free Cholesterol in Large HDL	-0.3 (-0.89, 0.3)	3.25E-01	0.19 (-0.31, 0.69)	4.51E-01	-0.03 (-0.50, 0.45)	0.917	0.932	0.2154	34.85%
Free Cholesterol in Very Large HDL	2.67 (1.46, 3.88)	1.49E-05	2.47 (1.53, 3.41)	2.65E-07	2.54 (1.80, 3.29)	<.0001	0.0002	0.7954	0.00%
Total Esterified Cholesterol	4.49 (2.76, 6.21)	3.52E-07	4.42 (3.05, 5.8)	2.90E-10	4.45 (3.37, 5.52)	<.0001	0.0002	0.9546	0.00%
Cholesteryl Esters in VLDL	3.29 (2.29, 4.28)	9.70E-11	2.66 (1.87, 3.46)	4.55E-11	2.91 (2.29, 3.52)	<.0001	0.0002	0.3375	0.00%
Cholesteryl Esters in Very Small VLDL	4.81 (3.53, 6.08)	1.52E-13	3.98 (2.98, 4.98)	6.97E-15	4.30 (3.50, 5.09)	<.0001	0.0002	0.3148	1.04%
Cholesteryl Esters in Small VLDL	2.95 (1.99, 3.91)	1.51E-09	2.5 (1.74, 3.26)	1.25E-10	2.67 (2.08, 3.27)	<.0001	0.0002	0.4654	0.00%
Cholesteryl Esters in Medium VLDL	2.17 (1.51, 2.82)	8.12E-11	1.89 (1.36, 2.42)	3.47E-12	2.00 (1.59, 2.41)	<.0001	0.0002	0.5128	0.00%
Cholesteryl Esters in Large VLDL	1.4 (0.75, 2.04)	2.31E-05	1.03 (0.52, 1.54)	8.68E-05	1.17 (0.77, 1.57)	<.0001	0.0002	0.3838	0.00%
Cholesteryl Esters in Very Large VLDL	1.14 (0.56, 1.72)	1.08E-04	0.94 (0.47, 1.41)	9.46E-05	1.02 (0.66, 1.39)	<.0001	0.0002	0.6013	0.00%
Cholesteryl Esters in Chylomicrons and Extremely Large VLDL	0.15 (-0.2, 0.51)	3.91E-01	0.04 (-0.24, 0.32)	8.01E-01	0.08 (-0.14, 0.30)	0.4649	0.5055	0.6064	0.00%
Cholesteryl Esters in IDL	4.77 (3.41, 6.13)	6.82E-12	4.38 (3.29, 5.47)	3.60E-15	4.53 (3.68, 5.38)	<.0001	0.0002	0.6615	0.00%

Cholesteryl Esters in LDL	4.16 (2.85, 5.46)	4.17E-10	4.13 (3.09, 5.16)	6.52E-15	4.14 (3.33, 4.95)	<.0001	0.0002	0.973	0.00%
Cholesteryl Esters in Small LDL	4.3 (2.97, 5.62)	2.08E-10	3.99 (2.93, 5.04)	1.47E-13	4.11 (3.28, 4.93)	<.0001	0.0002	0.7193	0.00%
Cholesteryl Esters in Medium LDL	3.19 (2.06, 4.32)	3.17E-08	3.19 (2.29, 4.09)	3.82E-12	3.19 (2.49, 3.90)	<.0001	0.0002	0.9966	0.00%
Cholesteryl Esters in Large LDL	4.31 (2.98, 5.64)	2.44E-10	4.31 (3.25, 5.37)	1.54E-15	4.31 (3.48, 5.14)	<.0001	0.0002	0.9998	0.00%
Cholesteryl Esters in HDL	-3.09 (-4.63, -1.54)	9.14E-05	-1.85 (-3.08, -0.61)	3.39E-03	-2.38 (-3.58, -1.17)	0.0001	0.0002	0.2194	33.70%
Cholesteryl Esters in Small HDL	-3.52 (-5.75, -1.29)	1.97E-03	-1.84 (-3.59, -0.09)	3.94E-02	-2.53 (-4.15, -0.91)	0.0022	0.0038	0.2455	25.85%
Cholesteryl Esters in Medium HDL	-4.27 (-5.76, -2.78)	1.92E-08	-2.83 (-4, -1.65)	2.43E-06	-3.47 (-4.88, -2.07)	<.0001	0.0002	0.1353	55.16%
Cholesteryl Esters in Large HDL	-0.74 (-1.35, -0.13)	1.71E-02	-0.28 (-0.79, 0.22)	2.72E-01	-0.48 (-0.92, -0.03)	0.0352	0.0492	0.255	22.82%
Cholesteryl Esters in Very Large HDL	0.51 (-0.22, 1.25)	1.71E-01	1.09 (0.49, 1.68)	3.88E-04	0.84 (0.29, 1.40)	0.0029	0.0049	0.2382	28.12%
Total Triglycerides	0.97 (0.17, 1.77)	1.73E-02	0.62 (-0.02, 1.27)	5.86E-02	0.76 (0.26, 1.26)	0.003	0.005	0.5046	0.00%
Triglycerides in VLDL	0.72 (0.06, 1.38)	3.31E-02	0.39 (-0.14, 0.93)	1.48E-01	0.52 (0.11, 0.94)	0.0137	0.0204	0.4559	0.00%
Triglycerides in Very Small VLDL	1.64 (0.55, 2.74)	3.32E-03	1.43 (0.57, 2.29)	1.13E-03	1.51 (0.83, 2.19)	<.0001	0.0002	0.7647	0.00%
Triglycerides in Small VLDL	0.85 (-0.02, 1.72)	5.56E-02	0.74 (0.05, 1.43)	3.50E-02	0.78 (0.24, 1.32)	0.0045	0.0074	0.8487	0.00%
Triglycerides in Medium VLDL	1.21 (0.41, 2.01)	3.01E-03	0.93 (0.29, 1.57)	4.37E-03	1.04 (0.54, 1.54)	<.0001	0.0002	0.5928	0.00%
Triglycerides in Large VLDL	0.75 (0.16, 1.34)	1.33E-02	0.48 (-0.01, 0.97)	5.29E-02	0.59 (0.21, 0.97)	0.0021	0.0036	0.5001	0.00%
Triglycerides in Very Large VLDL	0.31 (-0.09, 0.72)	1.26E-01	0.13 (-0.2, 0.46)	4.47E-01	0.20 (-0.05, 0.46)	0.1189	0.1526	0.4835	0.00%
Triglycerides in Chylomicrons and Extremely Large VLDL	0.04 (-0.16, 0.25)	6.78E-01	-0.16 (-0.3, -0.03)	1.56E-02	-0.08 (-0.28, 0.12)	0.4604	0.5028	0.0975	63.58%
Triglycerides in IDL	2.64 (1.34, 3.95)	6.80E-05	2.53 (1.5, 3.56)	1.59E-06	2.57 (1.76, 3.38)	<.0001	0.0002	0.8897	0.00%
Triglycerides in LDL	2.59 (1.36, 3.82)	3.62E-05	2.6 (1.61, 3.59)	2.82E-07	2.59 (1.82, 3.37)	<.0001	0.0002	0.996	0.00%
Triglycerides in Small LDL	1.5 (0.52, 2.49)	2.67E-03	1.24 (0.44, 2.04)	2.30E-03	1.35 (0.73, 1.97)	<.0001	0.0002	0.6836	0.00%
Triglycerides in Medium LDL	2.18 (1.06, 3.29)	1.31E-04	2.2 (1.3, 3.11)	1.90E-06	2.19 (1.49, 2.90)	<.0001	0.0002	0.9755	0.00%
Triglycerides in Large LDL	2.92 (1.62, 4.22)	1.06E-05	2.99 (1.94, 4.03)	2.05E-08	2.96 (2.15, 3.77)	<.0001	0.0002	0.9382	0.00%
Triglycerides in HDL	0.4 (-0.59, 1.4)	4.26E-01	0.1 (-0.68, 0.87)	8.05E-01	0.21 (-0.40, 0.82)	0.4947	0.5287	0.6338	0.00%
Triglycerides in Small HDL	0.45 (-0.53, 1.43)	3.70E-01	0.38 (-0.39, 1.16)	3.33E-01	0.41 (-0.20, 1.02)	0.1881	0.2265	0.919	0.00%
Triglycerides in Medium HDL	-0.11 (-1.01, 0.79)	8.10E-01	-0.32 (-1.01, 0.38)	3.75E-01	-0.24 (-0.79, 0.31)	0.3957	0.4438	0.7244	0.00%
Triglycerides in Large HDL	0.41 (-0.38, 1.21)	3.07E-01	0.06 (-0.55, 0.68)	8.41E-01	0.19 (-0.29, 0.68)	0.4333	0.4795	0.4923	0.00%
Triglycerides in Very Large HDL	1.61 (0.75, 2.47)	2.48E-04	1.2 (0.52, 1.89)	5.68E-04	1.36 (0.83, 1.90)	<.0001	0.0002	0.4675	0.00%
Total Phospholipids in Lipoprotein Particles	2.87 (0.72, 5.02)	8.85E-03	2.96 (1.26, 4.67)	6.70E-04	2.93 (1.59, 4.27)	<.0001	0.0002	0.9477	0.00%

Phospholipids in VLDL	2.1 (1.24, 2.97)	1.76E-06	1.58 (0.88, 2.27)	8.21E-06	1.78 (1.24, 2.32)	<.0001	0.0002	0.35	0.00%
Phospholipids in Very Small VLDL	4.58 (3.23, 5.94)	3.57E-11	3.55 (2.51, 4.6)	2.99E-11	3.97 (2.98, 4.97)	<.0001	0.0002	0.2373	28.40%
Phospholipids in Small VLDL	3.19 (2.13, 4.25)	3.63E-09	2.73 (1.89, 3.58)	2.26E-10	2.91 (2.25, 3.57)	<.0001	0.0002	0.5076	0.00%
Phospholipids in Medium VLDL	2.62 (1.77, 3.48)	1.96E-09	2.2 (1.5, 2.89)	6.88E-10	2.37 (1.83, 2.91)	<.0001	0.0002	0.4459	0.00%
Phospholipids in Large VLDL	0.37 (0, 0.74)	5.29E-02	0.27 (-0.02, 0.57)	7.22E-02	0.31 (0.08, 0.54)	0.009	0.014	0.6961	0.00%
Phospholipids in Very Large VLDL	0.3 (-0.04, 0.63)	8.29E-02	0.2 (-0.06, 0.46)	1.39E-01	0.23 (0.03, 0.44)	0.0255	0.0363	0.6477	0.00%
Phospholipids in Chylomicrons and Extremely Large VLDL	-0.06 (-0.27, 0.15)	5.84E-01	-0.12 (-0.27, 0.04)	1.40E-01	-0.10 (-0.22, 0.03)	0.1305	0.1649	0.6651	0.00%
Phospholipids in IDL	5.75 (4.17, 7.32)	8.75E-13	5.42 (4.18, 6.67)	2.22E-16	5.55 (4.57, 6.52)	<.0001	0.0002	0.752	0.00%
Phospholipids in LDL	4.97 (3.53, 6.41)	1.31E-11	4.82 (3.68, 5.96)	2.22E-16	4.88 (3.98, 5.77)	<.0001	0.0002	0.8702	0.00%
Phospholipids in Small LDL	6.65 (5.07, 8.23)	2.22E-16	6.3 (5.05, 7.55)	2.22E-16	6.44 (5.46, 7.41)	<.0001	0.0002	0.7334	0.00%
Phospholipids in Medium LDL	3.97 (2.73, 5.22)	4.17E-10	3.9 (2.91, 4.89)	1.56E-14	3.93 (3.15, 4.71)	<.0001	0.0002	0.9302	0.00%
Phospholipids in Large LDL	4.81 (3.35, 6.26)	1.06E-10	4.65 (3.49, 5.8)	3.20E-15	4.71 (3.80, 5.61)	<.0001	0.0002	0.8669	0.00%
Phospholipids in HDL	-3.65 (-5.45, -1.86)	6.73E-05	-2.4 (-3.83, -0.98)	9.53E-04	-2.90 (-4.11, -1.70)	<.0001	0.0002	0.2842	12.82%
Phospholipids in Small HDL	-4.39 (-6.76, -2.01)	2.94E-04	-2.99 (-4.86, -1.12)	1.73E-03	-3.52 (-4.99, -2.05)	<.0001	0.0002	0.3638	0.00%
Phospholipids in Medium HDL	-4.58 (-6.28, -2.88)	1.30E-07	-3.24 (-4.58, -1.9)	2.09E-06	-3.80 (-5.11, -2.50)	<.0001	0.0002	0.2232	32.61%
Phospholipids in Large HDL	-1.26 (-2.05, -0.47)	1.70E-03	-0.6 (-1.25, 0.04)	6.63E-02	-0.89 (-1.53, -0.25)	0.0063	0.0101	0.2052	37.71%
Phospholipids in Very Large HDL	0.29 (-0.21, 0.8)	2.56E-01	0.55 (0.19, 0.92)	2.73E-03	0.47 (0.17, 0.76)	0.002	0.0035	0.4103	0.00%
Mean VLDL Particle Size	0.37 (-10.69, 11.43)	9.48E-01	-5.01 (-14.17, 4.14)	2.83E-01	-2.82 (-9.87, 4.23)	0.4325	0.4795	0.4622	0.00%
Mean LDL Particle Size	5.25 (-3.46, 13.96)	2.37E-01	8.47 (1.43, 15.52)	1.84E-02	7.20 (1.72, 12.67)	0.01	0.0152	0.5732	0.00%
Mean HDL Particle Size	-1.45 (-3.20, 0.31)	1.06E-01	-1.08 (-2.52, 0.37)	1.45E-01	-1.23 (-2.34, -0.11)	0.0314	0.0442	0.7502	0.00%
Apolipoprotein A1	-4.32 (-6.52, -2.12)	1.17E-04	-2.82 (-4.55, -1.09)	1.41E-03	-3.41 (-4.85, -1.97)	<.0001	0.0002	0.2926	9.73%
Apolipoprotein B	5.56 (4.11, 7)	6.08E-14	4.92 (3.77, 6.07)	2.22E-16	5.16 (4.26, 6.06)	<.0001	0.0002	0.4995	0.00%

Estimates are percent changes in mean cIMT per fold increase in each (apo)lipoprotein measurement. Multivariate linear regression were separately conducted within participants from the two phases of NMR data releases adjusting for sex, age, ethnicity, BMI, SBP, DBP, smoking status, drinking status, physical activity level, diet, prevalent diabetes, use of cholesterol-lowering medications, family history of cardiovascular disease, and time-window between lipoprotein and cIMT measurements, and then meta-analyzed. Data on (apo)lipoprotein and mean cIMT were all logarithmically transformed prior to analysis. $P < 0.05$ was considered as nominally significant, and a looser criterion of nominal significance was employed at first in this stage of analyses to include as many as possible (apo)lipoproteins into the following evaluation of association with and therapeutic potential for ASCVD.

Table S2. Genetic correlation between (apo)lipoprotein profile and mean cIMT from LDSC regression analysis.

**(apo)lipoprotein profile**	**mean cIMT**			
	r_g	**SE**	***pval***	***FDR***
Total Concentration of Lipoprotein Particles	-0.1637	0.0509	0.0022	0.048
Total Concentration of VLDL Particles	-0.0309	0.0593	0.8115	0.844
Total Concentration of Very Small VLDL Particles	-0.0522	0.0696	0.3845	0.552
Total Concentration of Small VLDL Particles	-0.0200	0.0571	0.9632	0.969
Total Concentration of Medium VLDL Particles	-0.0731	0.0748	0.4310	0.582
Total Concentration of Large VLDL Particles	0.0091	0.0626	0.4767	0.606
Total Concentration of Very Large VLDL Particles	0.0230	0.0621	0.3231	0.504
Total Concentration of Chylomicrons and Extremely Large VLDL Particles	0.0440	0.0712	0.2104	0.474
Total Concentration of IDL Particles	-0.0794	0.1126	0.3442	0.524
Total Concentration of LDL Particles	-0.0999	0.0860	0.2053	0.474
Total Concentration of Small LDL Particles	-0.0673	0.0784	0.4055	0.553
Total Concentration of Medium LDL Particles	-0.0754	0.0782	0.3986	0.552
Total Concentration of Large LDL Particles	-0.1123	0.0913	0.1535	0.400
Total Concentration of HDL Particles	-0.1576	0.0527	0.0038	0.052
Total Concentration of Small HDL Particles	-0.1042	0.0660	0.3086	0.504
Total Concentration of Medium HDL Particles	-0.1407	0.0566	0.0103	0.079
Total Concentration of Large HDL Particles	-0.1145	0.0481	0.0045	0.052
Total Concentration of Very Large HDL Particles	-0.1130	0.0682	0.0326	0.181
Total Lipids in Lipoprotein Particles	-0.1357	0.0725	0.0861	0.342
Total Lipids in VLDL	-0.0035	0.0604	0.6513	0.701
Total Lipids in Very Small VLDL	-0.0539	0.0615	0.3292	0.507
Total Lipids in Small VLDL	-0.0286	0.0582	0.9110	0.932

Total Lipids in Medium VLDL	-0.0586	0.0691	0.5833	0.676
Total Lipids in Large VLDL	0.0049	0.0639	0.5190	0.635
Total Lipids in Very Large VLDL	0.0229	0.0630	0.3219	0.504
Total Lipids in Chylomicrons and Extremely Large VLDL	0.0408	0.0732	0.2135	0.474
Total Lipids in IDL	-0.1201	0.0972	0.1320	0.398
Total Lipids in LDL	-0.1285	0.0970	0.1549	0.400
Total Lipids in Small LDL	-0.1025	0.0974	0.2847	0.491
Total Lipids in Medium LDL	-0.1018	0.0853	0.2683	0.491
Total Lipids in Large LDL	-0.1400	0.0996	0.1184	0.398
Total Lipids in HDL	-0.1459	0.0474	0.0011	0.036
Total Lipids in Small HDL	-0.0765	0.0695	0.6112	0.684
Total Lipids in Medium HDL	-0.1340	0.0608	0.0293	0.174
Total Lipids in Large HDL	-0.1110	0.0486	0.0061	0.061
Total Lipids in Very Large HDL	-0.0958	0.0679	0.0519	0.252
Total Cholesterol	-0.1558	0.0763	0.0331	0.181
VLDL Cholesterol	-0.0477	0.0647	0.5742	0.675
Total Cholesterol in Very Small VLDL	-0.0742	0.0823	0.2237	0.481
Total Cholesterol in Small VLDL	-0.0605	0.0695	0.4464	0.591
Total Cholesterol in Medium VLDL	-0.1029	0.0806	0.1559	0.400
Total Cholesterol in Large VLDL	-0.0035	0.0608	0.6637	0.707
Total Cholesterol in Very Large VLDL	0.0076	0.0601	0.5133	0.634
Total Cholesterol in Chylomicrons and Extremely Large VLDL	0.0408	0.0718	0.2437	0.488
Total Cholesterol in IDL	-0.1108	0.0887	0.1132	0.394
LDL Cholesterol	-0.1394	0.1007	0.1322	0.398
Total Cholesterol in Small LDL	-0.1130	0.0987	0.2458	0.488
Total Cholesterol in Medium LDL	-0.1130	0.0887	0.2275	0.481

Total Cholesterol in Large LDL	-0.1289	0.0874	0.0904	0.348
HDL Cholesterol	-0.1437	0.0441	0.0006	0.031
Total Cholesterol in Small HDL	-0.1131	0.0641	0.2275	0.481
Total Cholesterol in Medium HDL	-0.1400	0.0501	0.0040	0.052
Total Cholesterol in Large HDL	-0.1116	0.0505	0.0079	0.065
Total Cholesterol in Very Large HDL	-0.1096	0.0745	0.0520	0.252
Total Free Cholesterol	-0.1386	0.0742	0.0546	0.253
Free Cholesterol in VLDL	-0.0240	0.0609	0.9692	0.969
Free Cholesterol in Very Small VLDL	-0.0637	0.0717	0.2908	0.492
Free Cholesterol in Small VLDL	-0.0840	0.0734	0.2649	0.491
Free Cholesterol in Medium VLDL	-0.0827	0.0723	0.2786	0.491
Free Cholesterol in Large VLDL	0.0102	0.0611	0.4654	0.604
Free Cholesterol in Very Large VLDL	0.0214	0.0611	0.3506	0.525
Free Cholesterol in Chylomicrons and Extremely Large VLDL	0.0461	0.0765	0.2325	0.483
Free Cholesterol in IDL	-0.1171	0.1017	0.1414	0.400
Free Cholesterol in LDL	-0.1253	0.1008	0.1294	0.398
Free Cholesterol in Small LDL	-0.1079	0.1267	0.2604	0.491
Free Cholesterol in Medium LDL	-0.1250	0.1008	0.1528	0.400
Free Cholesterol in Large LDL	-0.1226	0.0949	0.1102	0.394
Free Cholesterol in HDL	-0.1477	0.0456	0.0007	0.031
Free Cholesterol in Small HDL	-0.1271	0.0589	0.0735	0.311
Free Cholesterol in Medium HDL	-0.1472	0.0479	0.0015	0.039
Free Cholesterol in Large HDL	-0.1099	0.0536	0.0127	0.088
Free Cholesterol in Very Large HDL	-0.0670	0.0827	0.1685	0.416
Total Esterified Cholesterol	-0.1601	0.0765	0.0285	0.174
Cholesteryl Esters in VLDL	-0.0663	0.0703	0.3788	0.551

Cholesteryl Esters in Very Small VLDL	-0.0708	0.0807	0.2120	0.474
Cholesteryl Esters in Small VLDL	-0.0392	0.0621	0.6396	0.698
Cholesteryl Esters in Medium VLDL	-0.1132	0.0942	0.1445	0.400
Cholesteryl Esters in Large VLDL	-0.0209	0.0610	0.9455	0.960
Cholesteryl Esters in Very Large VLDL	-0.0092	0.0605	0.7633	0.800
Cholesteryl Esters in Chylomicrons and Extremely Large VLDL	0.0331	0.0684	0.2631	0.491
Cholesteryl Esters in IDL	-0.1099	0.0879	0.1142	0.394
Cholesteryl Esters in LDL	-0.1312	0.0919	0.1337	0.398
Cholesteryl Esters in Small LDL	-0.0885	0.0809	0.3193	0.504
Cholesteryl Esters in Medium LDL	-0.0943	0.0790	0.3139	0.504
Cholesteryl Esters in Large LDL	-0.1466	0.0988	0.1015	0.380
Cholesteryl Esters in HDL	-0.1430	0.0441	0.0006	0.031
Cholesteryl Esters in Small HDL	-0.1054	0.0657	0.3053	0.504
Cholesteryl Esters in Medium HDL	-0.1387	0.0508	0.0048	0.052
Cholesteryl Esters in Large HDL	-0.1128	0.0498	0.0067	0.063
Cholesteryl Esters in Very Large HDL	-0.1136	0.0699	0.0363	0.190
Total Triglycerides	0.0161	0.0638	0.4003	0.552
Triglycerides in VLDL	0.0155	0.0658	0.4001	0.552
Triglycerides in Very Small VLDL	0.0153	0.0564	0.4740	0.606
Triglycerides in Small VLDL	0.0019	0.0663	0.5930	0.681
Triglycerides in Medium VLDL	-0.0138	0.0655	0.7579	0.800
Triglycerides in Large VLDL	0.0041	0.0676	0.5032	0.628
Triglycerides in Very Large VLDL	0.0269	0.0663	0.2823	0.491
Triglycerides in Chylomicrons and Extremely Large VLDL	0.0464	0.0738	0.1650	0.416
Triglycerides in IDL	0.0082	0.0468	0.5767	0.675
Triglycerides in LDL	0.0026	0.0484	0.5627	0.670

Triglycerides in Small LDL	0.0091	0.0589	0.4600	0.603
Triglycerides in Medium LDL	0.0032	0.0508	0.5453	0.655
Triglycerides in Large LDL	0.0015	0.0463	0.6164	0.684
Triglycerides in HDL	0.0069	0.0797	0.6128	0.684
Triglycerides in Small HDL	0.0359	0.0665	0.2743	0.491
Triglycerides in Medium HDL	0.0034	0.0773	0.6114	0.684
Triglycerides in Large HDL	-0.0498	0.0777	0.5432	0.655
Triglycerides in Very Large HDL	-0.0402	0.0593	0.6361	0.698
Total Phospholipids in Lipoprotein Particles	-0.1519	0.0567	0.0118	0.086
Phospholipids in VLDL	-0.0114	0.0585	0.8248	0.851
Phospholipids in Very Small VLDL	-0.0359	0.0565	0.4817	0.607
Phospholipids in Small VLDL	-0.0649	0.0688	0.4462	0.591
Phospholipids in Medium VLDL	-0.0789	0.0756	0.3661	0.539
Phospholipids in Large VLDL	0.0154	0.0609	0.3981	0.552
Phospholipids in Very Large VLDL	0.0270	0.0616	0.2932	0.492
Phospholipids in Chylomicrons and Extremely Large VLDL	0.0529	0.0740	0.1803	0.437
Phospholipids in IDL	-0.1086	0.0925	0.1366	0.398
Phospholipids in LDL	-0.1060	0.0892	0.1862	0.443
Phospholipids in Small LDL	-0.0728	0.0981	0.3527	0.525
Phospholipids in Medium LDL	-0.1054	0.0937	0.2707	0.491
Phospholipids in Large LDL	-0.1138	0.0870	0.1339	0.398
Phospholipids in HDL	-0.1397	0.0506	0.0036	0.052
Phospholipids in Small HDL	-0.0735	0.0728	0.6526	0.701
Phospholipids in Medium HDL	-0.1242	0.0659	0.0768	0.314
Phospholipids in Large HDL	-0.1091	0.0484	0.0074	0.065
Phospholipids in Very Large HDL	-0.0836	0.0644	0.0624	0.272

Mean VLDL Particle Size	0.0373	0.0739	0.2714	0.491
Mean LDL Particle Size	-0.1561	0.1144	0.0561	0.253
Mean HDL Particle Size	-0.0932	0.0517	0.0200	0.131
Apolipoprotein A1	-0.1508	0.0523	0.0029	0.052
Apolipoprotein B	-0.0917	0.0840	0.2359	0.483

$P < 0.05$ was considered as nominally significant.

Table S3. Genetic instruments for (apo)lipoprotein profile to mean cIMT in two-sample Mendelian randomization (MR) analysis.

Exposure	SNP	Chr	Effect Allele	Alternate Allele	EAF	Beta	SE	*pval*	r^2	F statistic	Outcome
Total Concentration of Lipoprotein Particles	rs1002687	1	A	G	0.6448	0.0492	0.0041	5.80E-35	0.0013	146.8897	mean cIMT
Total Concentration of Lipoprotein Particles	rs11208717	1	C	T	0.3689	0.0251	0.0040	3.50E-10	0.0003	38.4707	mean cIMT
Total Concentration of Lipoprotein Particles	rs112495680	6	G	A	0.1334	-0.0324	0.0057	2.00E-08	0.0003	31.9564	mean cIMT
Total Concentration of Lipoprotein Particles	rs112875651	8	A	G	0.3924	-0.0236	0.0040	1.10E-08	0.0003	34.1162	mean cIMT
Total Concentration of Lipoprotein Particles	rs117687565	18	T	C	0.0120	0.1124	0.0185	2.20E-09	0.0003	36.9908	mean cIMT
Total Concentration of Lipoprotein Particles	rs11789603	9	T	C	0.1088	0.0587	0.0063	1.40E-20	0.0008	88.1494	mean cIMT
Total Concentration of Lipoprotein Particles	rs1260326	2	C	T	0.6040	-0.0567	0.0040	7.80E-46	0.0018	204.0334	mean cIMT
Total Concentration of Lipoprotein Particles	rs13107325	4	T	C	0.0743	-0.0628	0.0074	1.90E-18	0.0006	71.8958	mean cIMT
Total Concentration of Lipoprotein Particles	rs139915535	8	G	A	0.0180	-0.0951	0.0147	9.80E-12	0.0004	42.0979	mean cIMT
Total Concentration of Lipoprotein Particles	rs140168704	10	T	C	0.1535	-0.0299	0.0054	1.90E-08	0.0003	30.4740	mean cIMT
Total Concentration of Lipoprotein Particles	rs140584594	1	G	A	0.7301	0.0306	0.0044	5.50E-12	0.0004	49.2949	mean cIMT
Total Concentration of Lipoprotein Particles	rs148063610	10	C	CAAATAAAT	0.7634	-0.0320	0.0047	3.50E-12	0.0004	47.1166	mean cIMT
Total Concentration of Lipoprotein Particles	rs15285	8	T	C	0.2865	0.0731	0.0043	5.10E-65	0.0025	288.7404	mean cIMT
Total Concentration of Lipoprotein Particles	rs1560390	15	C	T	0.2197	-0.0269	0.0047	8.60E-10	0.0003	32.3253	mean cIMT
Total Concentration of Lipoprotein Particles	rs17696736	12	G	A	0.4302	-0.0237	0.0039	7.20E-11	0.0003	36.2526	mean cIMT
Total Concentration of Lipoprotein Particles	rs17699030	19	G	A	0.0337	-0.1130	0.0108	4.90E-27	0.0010	110.2917	mean cIMT
Total Concentration of Lipoprotein Particles	rs1800961	20	T	C	0.0302	-0.1291	0.0114	5.70E-30	0.0011	129.3641	mean cIMT
Total Concentration of Lipoprotein Particles	rs193084249	1	G	A	0.0234	-0.0841	0.0131	1.40E-10	0.0004	40.9877	mean cIMT
Total Concentration of Lipoprotein Particles	rs2066714	9	C	T	0.1289	0.0345	0.0058	6.40E-10	0.0003	35.5614	mean cIMT
Total Concentration of Lipoprotein Particles	rs2071379	17	G	A	0.5962	-0.0231	0.0040	1.20E-08	0.0003	34.0404	mean cIMT
Total Concentration of Lipoprotein Particles	rs2298428	22	T	C	0.1826	-0.0411	0.0050	4.50E-17	0.0006	66.7358	mean cIMT
Total Concentration of Lipoprotein Particles	rs2385020	19	G	A	0.3084	0.0292	0.0043	7.70E-13	0.0004	46.5830	mean cIMT
Total Concentration of Lipoprotein Particles	rs2494748	14	T	C	0.6162	-0.0219	0.0040	8.80E-09	0.0003	29.9882	mean cIMT
Total Concentration of Lipoprotein Particles	rs261290	15	C	T	0.6546	-0.0677	0.0041	9.30E-63	0.0024	273.0737	mean cIMT
Total Concentration of Lipoprotein Particles	rs2642438	1	G	A	0.7038	0.0357	0.0043	7.60E-18	0.0006	70.1278	mean cIMT
Total Concentration of Lipoprotein Particles	rs267738	1	G	T	0.2196	0.0348	0.0047	2.90E-14	0.0005	55.0226	mean cIMT

Total Concentration of Lipoprotein Particles	rs2740488	9	C	A	0.2653	-0.0508	0.0044	7.70E-32	0.0012	132.6148	mean cIMT
Total Concentration of Lipoprotein Particles	rs2792735	10	A	G	0.7203	-0.0373	0.0043	6.70E-19	0.0006	74.1905	mean cIMT
Total Concentration of Lipoprotein Particles	rs2925979	16	C	T	0.6989	0.0259	0.0042	6.10E-11	0.0003	37.5116	mean cIMT
Total Concentration of Lipoprotein Particles	rs2943650	2	T	C	0.6448	-0.0272	0.0041	1.00E-12	0.0004	45.1509	mean cIMT
Total Concentration of Lipoprotein Particles	rs35135293	2	T	C	0.5167	-0.0285	0.0039	2.00E-13	0.0005	53.1737	mean cIMT
Total Concentration of Lipoprotein Particles	rs3741521	12	A	G	0.6697	-0.0306	0.0042	2.90E-14	0.0005	52.7359	mean cIMT
Total Concentration of Lipoprotein Particles	rs3764261	16	A	C	0.3244	0.1398	0.0041	1.00E-200	0.0098	1135.7568	mean cIMT
Total Concentration of Lipoprotein Particles	rs3768321	1	T	G	0.1965	-0.0416	0.0049	1.30E-18	0.0006	72.2263	mean cIMT
Total Concentration of Lipoprotein Particles	rs4239651	20	C	T	0.7942	0.0294	0.0048	2.90E-10	0.0003	37.4300	mean cIMT
Total Concentration of Lipoprotein Particles	rs4240624	8	A	G	0.9092	0.1152	0.0068	2.10E-65	0.0025	289.6498	mean cIMT
Total Concentration of Lipoprotein Particles	rs4656292	1	G	A	0.6196	0.0224	0.0040	4.40E-09	0.0003	31.2279	mean cIMT
Total Concentration of Lipoprotein Particles	rs4721672	7	C	T	0.4919	0.0212	0.0039	3.70E-08	0.0003	29.4833	mean cIMT
Total Concentration of Lipoprotein Particles	rs473224	15	G	T	0.8539	-0.0599	0.0056	4.40E-27	0.0010	115.3299	mean cIMT
Total Concentration of Lipoprotein Particles	rs4846921	1	A	G	0.6132	0.0363	0.0040	1.60E-20	0.0007	82.6667	mean cIMT
Total Concentration of Lipoprotein Particles	rs4969141	17	T	C	0.4895	0.0249	0.0039	1.00E-10	0.0004	40.8993	mean cIMT
Total Concentration of Lipoprotein Particles	rs507666	9	A	G	0.1839	0.0369	0.0050	4.70E-14	0.0005	53.9337	mean cIMT
Total Concentration of Lipoprotein Particles	rs5167	19	G	T	0.3523	0.0427	0.0041	2.50E-26	0.0010	110.5481	mean cIMT
Total Concentration of Lipoprotein Particles	rs56030824	11	A	G	0.3231	-0.0369	0.0042	3.00E-19	0.0007	78.7808	mean cIMT
Total Concentration of Lipoprotein Particles	rs56912706	5	G	A	0.3788	0.0240	0.0040	1.60E-09	0.0003	35.3692	mean cIMT
Total Concentration of Lipoprotein Particles	rs58542926	19	T	C	0.0744	-0.0600	0.0074	2.00E-17	0.0006	65.4890	mean cIMT
Total Concentration of Lipoprotein Particles	rs60847460	10	T	C	0.1433	-0.0325	0.0056	1.10E-08	0.0003	34.1274	mean cIMT
Total Concentration of Lipoprotein Particles	rs62101705	18	T	C	0.0136	-0.0928	0.0171	4.50E-08	0.0003	29.5837	mean cIMT
Total Concentration of Lipoprotein Particles	rs6606717	12	C	A	0.5232	0.0260	0.0039	8.20E-12	0.0004	44.7016	mean cIMT
Total Concentration of Lipoprotein Particles	rs673335	11	C	T	0.1598	-0.0588	0.0053	3.30E-28	0.0011	122.1940	mean cIMT
Total Concentration of Lipoprotein Particles	rs673548	2	A	G	0.2061	0.0375	0.0048	4.40E-14	0.0005	61.1331	mean cIMT
Total Concentration of Lipoprotein Particles	rs688456	11	T	G	0.1923	0.0746	0.0049	1.60E-54	0.0020	228.7174	mean cIMT
Total Concentration of Lipoprotein Particles	rs7138492	12	A	G	0.8620	0.0346	0.0060	4.30E-09	0.0003	33.4268	mean cIMT
Total Concentration of Lipoprotein Particles	rs72836561	17	T	C	0.0315	-0.1200	0.0111	3.00E-28	0.0010	116.1722	mean cIMT

Total Concentration of Lipoprotein Particles	rs75627662	19	T	C	0.2061	-0.0265	0.0048	2.10E-08	0.0003	30.4827	mean cIMT
Total Concentration of Lipoprotein Particles	rs75911530	16	A	G	0.0329	-0.1250	0.0115	5.70E-29	0.0010	119.1294	mean cIMT
Total Concentration of Lipoprotein Particles	rs77960347	18	G	A	0.0132	0.3018	0.0170	1.90E-71	0.0027	316.2387	mean cIMT
Total Concentration of Lipoprotein Particles	rs79366941	19	G	A	0.0909	0.0419	0.0070	5.50E-10	0.0003	36.0410	mean cIMT
Total Concentration of Lipoprotein Particles	rs79834165	6	C	T	0.0340	-0.0633	0.0107	3.70E-09	0.0003	34.6497	mean cIMT
Total Concentration of Lipoprotein Particles	rs9304381	18	T	C	0.8184	0.0881	0.0050	1.50E-68	0.0026	305.1891	mean cIMT
Total Concentration of HDL Particles	rs1002687	1	A	G	0.6448	0.0446	0.0041	2.10E-29	0.0011	121.4300	mean cIMT
Total Concentration of HDL Particles	rs1047891	2	A	C	0.3150	-0.0251	0.0042	4.70E-09	0.0003	36.2300	mean cIMT
Total Concentration of HDL Particles	rs1077835	15	G	A	0.2197	0.0600	0.0047	3.30E-38	0.0014	163.6057	mean cIMT
Total Concentration of HDL Particles	rs11039238	11	C	T	0.3222	-0.0370	0.0041	2.20E-19	0.0007	79.8864	mean cIMT
Total Concentration of HDL Particles	rs112001035	17	A	G	0.0597	-0.0533	0.0083	3.30E-10	0.0004	40.8771	mean cIMT
Total Concentration of HDL Particles	rs11208717	1	C	T	0.3689	0.0247	0.0040	5.40E-10	0.0003	37.4993	mean cIMT
Total Concentration of HDL Particles	rs112495680	6	G	A	0.1334	-0.0330	0.0057	8.00E-09	0.0003	33.2565	mean cIMT
Total Concentration of HDL Particles	rs116843064	19	A	G	0.0199	0.1577	0.0139	4.60E-31	0.0011	128.5038	mean cIMT
Total Concentration of HDL Particles	rs117687565	18	T	C	0.0120	0.1163	0.0184	5.50E-10	0.0003	39.7463	mean cIMT
Total Concentration of HDL Particles	rs11789603	9	T	C	0.1088	0.0588	0.0062	1.40E-20	0.0008	88.5921	mean cIMT
Total Concentration of HDL Particles	rs1260326	2	C	T	0.6040	-0.0529	0.0040	7.40E-40	0.0015	178.1303	mean cIMT
Total Concentration of HDL Particles	rs12721046	19	A	G	0.1577	-0.0311	0.0053	4.50E-09	0.0003	33.9990	mean cIMT
Total Concentration of HDL Particles	rs13107325	4	T	C	0.0743	-0.0657	0.0074	5.20E-20	0.0007	79.0143	mean cIMT
Total Concentration of HDL Particles	rs1321257	1	A	G	0.6132	0.0411	0.0040	6.40E-26	0.0009	106.9342	mean cIMT
Total Concentration of HDL Particles	rs139915535	8	G	A	0.0180	-0.1105	0.0146	4.20E-15	0.0005	57.0450	mean cIMT
Total Concentration of HDL Particles	rs140168704	10	T	C	0.1535	-0.0288	0.0054	4.60E-08	0.0002	28.4838	mean cIMT
Total Concentration of HDL Particles	rs140584594	1	G	A	0.7301	0.0351	0.0043	9.40E-16	0.0006	65.3375	mean cIMT
Total Concentration of HDL Particles	rs148063610	10	C	CAAATAAAT	0.7634	-0.0324	0.0047	2.10E-12	0.0004	48.4917	mean cIMT
Total Concentration of HDL Particles	rs15285	8	T	C	0.2865	0.0816	0.0043	2.60E-81	0.0031	361.9876	mean cIMT
Total Concentration of HDL Particles	rs1601935	15	T	G	0.6547	-0.0629	0.0041	9.50E-54	0.0020	233.9689	mean cIMT
Total Concentration of HDL Particles	rs1761457	19	G	A	0.3267	0.0297	0.0041	7.10E-14	0.0004	51.3685	mean cIMT
Total Concentration of HDL Particles	rs17696736	12	G	A	0.4302	-0.0215	0.0039	1.50E-09	0.0003	30.0358	mean cIMT

Total Concentration of HDL Particles	rs1800961	20	T	C	0.0302	-0.1316	0.0113	5.00E-31	0.0012	135.0185	mean cIMT
Total Concentration of HDL Particles	rs193084249	1	G	A	0.0234	-0.0952	0.0131	3.90E-13	0.0005	52.8077	mean cIMT
Total Concentration of HDL Particles	rs2066714	9	C	T	0.1289	0.0340	0.0058	8.90E-10	0.0003	34.6987	mean cIMT
Total Concentration of HDL Particles	rs2236252	20	T	C	0.1670	-0.0285	0.0052	1.40E-08	0.0003	30.0920	mean cIMT
Total Concentration of HDL Particles	rs2298428	22	T	C	0.1826	-0.0421	0.0050	6.30E-18	0.0006	70.2971	mean cIMT
Total Concentration of HDL Particles	rs2494748	14	T	C	0.6162	-0.0236	0.0040	8.00E-10	0.0003	34.8822	mean cIMT
Total Concentration of HDL Particles	rs2519093	9	T	C	0.1837	0.0288	0.0050	3.00E-09	0.0003	32.9931	mean cIMT
Total Concentration of HDL Particles	rs2642438	1	G	A	0.7038	0.0351	0.0042	2.60E-17	0.0006	68.1037	mean cIMT
Total Concentration of HDL Particles	rs267738	1	G	T	0.2196	0.0383	0.0047	3.10E-17	0.0006	66.7929	mean cIMT
Total Concentration of HDL Particles	rs2740488	9	C	A	0.2653	-0.0494	0.0044	2.70E-30	0.0011	125.7884	mean cIMT
Total Concentration of HDL Particles	rs2792735	10	A	G	0.7203	-0.0371	0.0043	7.30E-19	0.0006	73.6370	mean cIMT
Total Concentration of HDL Particles	rs2925979	16	C	T	0.6989	0.0277	0.0042	3.10E-12	0.0004	43.1397	mean cIMT
Total Concentration of HDL Particles	rs2943650	2	T	C	0.6448	-0.0280	0.0040	2.00E-13	0.0004	47.8295	mean cIMT
Total Concentration of HDL Particles	rs35135293	2	T	C	0.5167	-0.0272	0.0039	1.50E-12	0.0004	48.8796	mean cIMT
Total Concentration of HDL Particles	rs3764261	16	A	C	0.3244	0.1551	0.0041	1.00E-200	0.0121	1406.7559	mean cIMT
Total Concentration of HDL Particles	rs3768321	1	T	G	0.1965	-0.0433	0.0049	5.10E-20	0.0007	78.4996	mean cIMT
Total Concentration of HDL Particles	rs4239651	20	C	T	0.7942	0.0315	0.0048	1.00E-11	0.0004	43.0352	mean cIMT
Total Concentration of HDL Particles	rs4240624	8	A	G	0.9092	0.1151	0.0068	9.10E-66	0.0025	290.5133	mean cIMT
Total Concentration of HDL Particles	rs4656292	1	G	A	0.6196	0.0238	0.0040	3.70E-10	0.0003	35.4730	mean cIMT
Total Concentration of HDL Particles	rs4721672	7	C	T	0.4919	0.0222	0.0039	6.60E-09	0.0003	32.5527	mean cIMT
Total Concentration of HDL Particles	rs4969141	17	T	C	0.4895	0.0250	0.0039	7.90E-11	0.0004	41.2236	mean cIMT
Total Concentration of HDL Particles	rs5167	19	G	T	0.3523	0.0449	0.0041	9.50E-29	0.0011	122.4601	mean cIMT
Total Concentration of HDL Particles	rs60847460	10	T	C	0.1433	-0.0346	0.0056	1.10E-09	0.0003	38.8177	mean cIMT
Total Concentration of HDL Particles	rs61941676	12	A	C	0.1272	-0.0439	0.0060	1.30E-13	0.0005	54.1581	mean cIMT
Total Concentration of HDL Particles	rs62101704	18	A	G	0.0137	-0.0927	0.0170	2.80E-08	0.0003	29.7281	mean cIMT
Total Concentration of HDL Particles	rs6606717	12	C	A	0.5232	0.0266	0.0039	2.70E-12	0.0004	47.2545	mean cIMT
Total Concentration of HDL Particles	rs673548	2	A	G	0.2061	0.0503	0.0048	8.90E-25	0.0010	110.9408	mean cIMT
Total Concentration of HDL Particles	rs686030	9	A	C	0.8595	0.0385	0.0056	5.00E-12	0.0004	47.5087	mean cIMT

Total Concentration of HDL Particles	rs72836561	17	T	C	0.0315	-0.1258	0.0111	6.80E-31	0.0011	128.2317	mean cIMT
Total Concentration of HDL Particles	rs737337	19	C	T	0.0768	-0.0655	0.0073	1.40E-19	0.0007	81.0423	mean cIMT
Total Concentration of HDL Particles	rs75911530	16	A	G	0.0329	-0.1371	0.0114	1.40E-34	0.0013	144.0517	mean cIMT
Total Concentration of HDL Particles	rs77960347	18	G	A	0.0132	0.3037	0.0169	3.60E-73	0.0028	321.7131	mean cIMT
Total Concentration of HDL Particles	rs8100204	19	A	G	0.1453	-0.0340	0.0056	9.10E-11	0.0003	36.3657	mean cIMT
Total Concentration of HDL Particles	rs838876	12	G	A	0.6814	-0.0325	0.0042	4.80E-16	0.0005	59.3920	mean cIMT
Total Concentration of HDL Particles	rs9304381	18	T	C	0.8184	0.0903	0.0050	1.30E-72	0.0028	321.4963	mean cIMT
Total Concentration of HDL Particles	rs9471972	6	A	G	0.5362	0.0216	0.0039	3.80E-08	0.0003	30.8601	mean cIMT
Total Concentration of HDL Particles	rs967645	17	T	C	0.5165	-0.0253	0.0039	3.80E-10	0.0004	42.5562	mean cIMT
Total Concentration of Medium HDL Particles	rs10184004	2	T	C	0.4061	0.0214	0.0039	1.00E-08	0.0003	30.5696	mean cIMT
Total Concentration of Medium HDL Particles	rs1047891	2	A	C	0.3150	-0.0258	0.0041	5.20E-10	0.0003	39.7339	mean cIMT
Total Concentration of Medium HDL Particles	rs1065853	19	T	G	0.0806	0.1388	0.0070	8.90E-92	0.0034	391.1111	mean cIMT
Total Concentration of Medium HDL Particles	rs1077835	15	G	A	0.2197	0.1028	0.0046	2.10E-115	0.0043	499.7994	mean cIMT
Total Concentration of Medium HDL Particles	rs112001035	17	A	G	0.0597	-0.0540	0.0082	1.20E-10	0.0004	43.5202	mean cIMT
Total Concentration of Medium HDL Particles	rs112495680	6	G	A	0.1334	-0.0352	0.0056	2.10E-10	0.0003	39.2809	mean cIMT
Total Concentration of Medium HDL Particles	rs117687565	18	T	C	0.0120	0.1331	0.0181	7.70E-13	0.0005	54.1278	mean cIMT
Total Concentration of Medium HDL Particles	rs11789603	9	T	C	0.1088	0.0542	0.0061	6.00E-18	0.0007	78.1449	mean cIMT
Total Concentration of Medium HDL Particles	rs1260326	2	C	T	0.6040	-0.0385	0.0039	4.30E-22	0.0009	98.2344	mean cIMT
Total Concentration of Medium HDL Particles	rs12904367	15	A	G	0.1413	0.0306	0.0056	3.70E-08	0.0003	30.1188	mean cIMT
Total Concentration of Medium HDL Particles	rs12976739	19	A	G	0.3962	0.0258	0.0039	5.80E-12	0.0004	43.5992	mean cIMT
Total Concentration of Medium HDL Particles	rs13107325	4	T	C	0.0743	-0.0681	0.0073	7.00E-22	0.0008	88.0388	mean cIMT
Total Concentration of Medium HDL Particles	rs1321257	1	A	G	0.6132	0.0507	0.0039	5.60E-40	0.0015	168.8640	mean cIMT
Total Concentration of Medium HDL Particles	rs139915535	8	G	A	0.0180	-0.1280	0.0144	1.60E-20	0.0007	79.5477	mean cIMT
Total Concentration of Medium HDL Particles	rs140584594	1	G	A	0.7301	0.0377	0.0043	2.60E-19	0.0007	78.3489	mean cIMT
Total Concentration of Medium HDL Particles	rs148063610	10	C	CAAATAAAT	0.7634	-0.0336	0.0046	1.50E-13	0.0005	53.9355	mean cIMT
Total Concentration of Medium HDL Particles	rs15285	8	T	C	0.2865	0.0864	0.0042	8.00E-96	0.0036	421.0412	mean cIMT
Total Concentration of Medium HDL Particles	rs1800961	20	T	C	0.0302	-0.1189	0.0111	3.20E-26	0.0010	114.3704	mean cIMT
Total Concentration of Medium HDL Particles	rs193084249	1	G	A	0.0234	-0.1051	0.0129	1.90E-16	0.0006	66.8280	mean cIMT

Total Concentration of Medium HDL Particles	rs2043085	15	C	T	0.6127	-0.0974	0.0039	5.00E-141	0.0054	623.1655	mean cIMT
Total Concentration of Medium HDL Particles	rs2066714	9	C	T	0.1289	0.0319	0.0057	4.30E-09	0.0003	31.7719	mean cIMT
Total Concentration of Medium HDL Particles	rs2176040	2	G	A	0.6467	-0.0287	0.0040	1.20E-14	0.0005	52.1312	mean cIMT
Total Concentration of Medium HDL Particles	rs2229357	12	A	G	0.2409	0.0237	0.0044	4.50E-08	0.0002	28.4813	mean cIMT
Total Concentration of Medium HDL Particles	rs2236252	20	T	C	0.1670	-0.0316	0.0051	1.20E-10	0.0003	38.4088	mean cIMT
Total Concentration of Medium HDL Particles	rs2245221	8	A	G	0.5590	0.0340	0.0039	7.90E-18	0.0007	77.7580	mean cIMT
Total Concentration of Medium HDL Particles	rs2298428	22	T	C	0.1826	-0.0414	0.0049	8.50E-18	0.0006	70.7345	mean cIMT
Total Concentration of Medium HDL Particles	rs235314	21	T	C	0.5327	-0.0213	0.0038	3.50E-08	0.0003	31.1703	mean cIMT
Total Concentration of Medium HDL Particles	rs2494748	14	T	C	0.6162	-0.0220	0.0039	4.20E-09	0.0003	31.3733	mean cIMT
Total Concentration of Medium HDL Particles	rs2642438	1	G	A	0.7038	0.0353	0.0042	3.40E-18	0.0006	71.9138	mean cIMT
Total Concentration of Medium HDL Particles	rs267738	1	G	T	0.2196	0.0369	0.0046	8.10E-17	0.0006	64.4236	mean cIMT
Total Concentration of Medium HDL Particles	rs2740488	9	C	A	0.2653	-0.0432	0.0043	1.00E-24	0.0009	99.7647	mean cIMT
Total Concentration of Medium HDL Particles	rs2781752	19	A	G	0.3182	0.0259	0.0041	2.50E-11	0.0003	39.6298	mean cIMT
Total Concentration of Medium HDL Particles	rs2792735	10	A	G	0.7203	-0.0396	0.0042	5.80E-22	0.0008	87.3812	mean cIMT
Total Concentration of Medium HDL Particles	rs28818616	3	C	T	0.3498	-0.0218	0.0040	3.00E-08	0.0003	29.5504	mean cIMT
Total Concentration of Medium HDL Particles	rs2925979	16	C	T	0.6989	0.0300	0.0041	1.80E-14	0.0005	52.7171	mean cIMT
Total Concentration of Medium HDL Particles	rs35135293	2	T	C	0.5167	-0.0275	0.0038	1.30E-13	0.0005	51.8373	mean cIMT
Total Concentration of Medium HDL Particles	rs35184771	11	T	G	0.3528	-0.0358	0.0040	4.70E-19	0.0007	81.0313	mean cIMT
Total Concentration of Medium HDL Particles	rs3764261	16	A	C	0.3244	0.1700	0.0041	1.00E-200	0.0150	1755.7485	mean cIMT
Total Concentration of Medium HDL Particles	rs3768321	1	T	G	0.1965	-0.0403	0.0048	3.70E-18	0.0006	70.5655	mean cIMT
Total Concentration of Medium HDL Particles	rs3795269	1	A	C	0.4386	0.0208	0.0038	3.20E-08	0.0003	29.7507	mean cIMT
Total Concentration of Medium HDL Particles	rs4239651	20	C	T	0.7942	0.0367	0.0047	5.50E-16	0.0005	60.7350	mean cIMT
Total Concentration of Medium HDL Particles	rs4240624	8	A	G	0.9092	0.1005	0.0066	1.00E-52	0.0020	230.2255	mean cIMT
Total Concentration of Medium HDL Particles	rs4969141	17	T	C	0.4895	0.0216	0.0038	8.00E-09	0.0003	31.8789	mean cIMT
Total Concentration of Medium HDL Particles	rs583104	1	T	G	0.7734	-0.0445	0.0045	6.30E-24	0.0008	96.2719	mean cIMT
Total Concentration of Medium HDL Particles	rs60847460	10	T	C	0.1433	-0.0380	0.0055	6.30E-12	0.0004	48.5849	mean cIMT
Total Concentration of Medium HDL Particles	rs61805076	1	C	T	0.3341	-0.0266	0.0040	1.90E-11	0.0004	43.4645	mean cIMT
Total Concentration of Medium HDL Particles	rs61941676	12	A	C	0.1272	-0.0547	0.0059	6.70E-21	0.0008	87.3789	mean cIMT

Total Concentration of Medium HDL Particles	rs62101704	18	A	G	0.0137	-0.0904	0.0167	1.80E-08	0.0003	29.4299	mean cIMT
Total Concentration of Medium HDL Particles	rs638714	1	T	G	0.3459	-0.0459	0.0040	3.40E-31	0.0011	130.9553	mean cIMT
Total Concentration of Medium HDL Particles	rs6606717	12	C	A	0.5232	0.0299	0.0038	2.00E-15	0.0005	61.9292	mean cIMT
Total Concentration of Medium HDL Particles	rs676210	2	A	G	0.2058	0.0618	0.0047	8.50E-39	0.0015	173.7534	mean cIMT
Total Concentration of Medium HDL Particles	rs686030	9	A	C	0.8595	0.0382	0.0055	1.20E-12	0.0004	48.5126	mean cIMT
Total Concentration of Medium HDL Particles	rs72836561	17	T	C	0.0315	-0.1307	0.0109	1.50E-34	0.0012	143.8236	mean cIMT
Total Concentration of Medium HDL Particles	rs737338	19	T	C	0.0352	-0.1204	0.0103	5.90E-33	0.0012	135.8746	mean cIMT
Total Concentration of Medium HDL Particles	rs75911530	16	A	G	0.0329	-0.1400	0.0112	2.00E-37	0.0014	156.2858	mean cIMT
Total Concentration of Medium HDL Particles	rs76213248	19	T	C	0.4097	0.0306	0.0039	6.80E-15	0.0005	61.7556	mean cIMT
Total Concentration of Medium HDL Particles	rs77960347	18	G	A	0.0132	0.3216	0.0166	4.90E-86	0.0032	374.8592	mean cIMT
Total Concentration of Medium HDL Particles	rs7956099	12	C	T	0.4750	-0.0232	0.0038	6.60E-10	0.0003	36.6691	mean cIMT
Total Concentration of Medium HDL Particles	rs7959043	12	G	A	0.4084	0.0210	0.0039	2.30E-08	0.0003	29.2603	mean cIMT
Total Concentration of Medium HDL Particles	rs8058512	16	T	C	0.7240	-0.0237	0.0043	3.30E-08	0.0003	30.8860	mean cIMT
Total Concentration of Medium HDL Particles	rs838876	12	G	A	0.6814	-0.0376	0.0041	1.90E-21	0.0007	82.4937	mean cIMT
Total Concentration of Medium HDL Particles	rs9274346	6	T	C	0.3919	0.0227	0.0040	6.30E-09	0.0003	31.8943	mean cIMT
Total Concentration of Medium HDL Particles	rs9304381	18	T	C	0.8184	0.0955	0.0049	5.00E-85	0.0032	374.0542	mean cIMT
Total Concentration of Medium HDL Particles	rs9471972	6	A	G	0.5362	0.0262	0.0038	1.00E-11	0.0004	47.0202	mean cIMT
Total Concentration of Medium HDL Particles	rs9491697	6	G	A	0.4636	-0.0221	0.0038	1.00E-08	0.0003	33.1445	mean cIMT
Total Concentration of Medium HDL Particles	rs967645	17	T	C	0.5165	-0.0295	0.0038	8.00E-14	0.0005	60.2157	mean cIMT
Total Lipids in HDL	rs10184004	2	T	C	0.4061	0.0254	0.0038	1.80E-12	0.0004	45.1840	mean cIMT
Total Lipids in HDL	rs11045171	12	G	A	0.1974	0.0258	0.0047	9.40E-09	0.0003	30.2862	mean cIMT
Total Lipids in HDL	rs11057692	12	G	A	0.2363	-0.0232	0.0044	1.80E-08	0.0002	27.6614	mean cIMT
Total Lipids in HDL	rs112001035	17	A	G	0.0597	-0.0509	0.0080	5.70E-10	0.0004	40.7383	mean cIMT
Total Lipids in HDL	rs116843064	19	A	G	0.0199	0.1673	0.0133	7.50E-38	0.0014	158.0945	mean cIMT
Total Lipids in HDL	rs11751347	6	T	C	0.1023	-0.0375	0.0062	4.30E-10	0.0003	36.9974	mean cIMT
Total Lipids in HDL	rs11789603	9	T	C	0.1088	0.0551	0.0060	3.50E-19	0.0007	84.9977	mean cIMT
Total Lipids in HDL	rs12295878	11	T	C	0.1403	0.0310	0.0053	2.00E-08	0.0003	33.6244	mean cIMT
Total Lipids in HDL	rs12608026	18	G	T	0.0424	0.0874	0.0093	8.50E-22	0.0008	88.4975	mean cIMT

Total Lipids in HDL	rs12721046	19	A	G	0.1577	-0.0499	0.0051	4.20E-23	0.0008	95.8331	mean cIMT
Total Lipids in HDL	rs13107325	4	T	C	0.0743	-0.0713	0.0071	1.60E-25	0.0009	101.7111	mean cIMT
Total Lipids in HDL	rs1358980	6	T	C	0.4826	-0.0262	0.0037	5.70E-12	0.0004	49.0266	mean cIMT
Total Lipids in HDL	rs139915535	8	G	A	0.0180	-0.1269	0.0140	2.10E-21	0.0007	82.3115	mean cIMT
Total Lipids in HDL	rs140584594	1	G	A	0.7301	0.0290	0.0042	1.00E-12	0.0004	48.9278	mean cIMT
Total Lipids in HDL	rs141368429	11	T	C	0.0562	-0.0504	0.0087	6.40E-09	0.0003	33.8680	mean cIMT
Total Lipids in HDL	rs147627829	6	A	G	0.0441	-0.0581	0.0091	2.30E-10	0.0004	40.8095	mean cIMT
Total Lipids in HDL	rs148063610	10	C	CAAATAAAT	0.7634	-0.0350	0.0045	1.70E-15	0.0005	61.7159	mean cIMT
Total Lipids in HDL	rs150224153	20	T	C	0.0292	-0.0828	0.0113	5.30E-13	0.0005	53.7652	mean cIMT
Total Lipids in HDL	rs15285	8	T	C	0.2865	0.0861	0.0041	7.60E-100	0.0038	440.3419	mean cIMT
Total Lipids in HDL	rs174574	11	C	A	0.6494	0.0538	0.0039	4.20E-47	0.0017	192.5510	mean cIMT
Total Lipids in HDL	rs17696736	12	G	A	0.4302	-0.0193	0.0037	9.10E-09	0.0002	26.3698	mean cIMT
Total Lipids in HDL	rs193084249	1	G	A	0.0234	-0.0961	0.0125	2.10E-14	0.0005	58.7447	mean cIMT
Total Lipids in HDL	rs2043085	15	C	T	0.6127	-0.1249	0.0038	1.00E-200	0.0093	1079.1776	mean cIMT
Total Lipids in HDL	rs2066714	9	C	T	0.1289	0.0340	0.0055	1.80E-10	0.0003	38.0088	mean cIMT
Total Lipids in HDL	rs2070895	15	A	G	0.2187	0.1388	0.0045	1.00E-200	0.0083	958.7607	mean cIMT
Total Lipids in HDL	rs2176040	2	G	A	0.6467	-0.0326	0.0039	4.70E-19	0.0006	70.7694	mean cIMT
Total Lipids in HDL	rs2229357	12	A	G	0.2409	0.0240	0.0043	9.80E-09	0.0003	30.7046	mean cIMT
Total Lipids in HDL	rs2236252	20	T	C	0.1670	-0.0281	0.0050	5.90E-09	0.0003	32.0104	mean cIMT
Total Lipids in HDL	rs2243616	12	T	G	0.6611	-0.0223	0.0039	2.30E-08	0.0003	32.4358	mean cIMT
Total Lipids in HDL	rs2245221	8	A	G	0.5590	0.0338	0.0038	1.50E-18	0.0007	80.6905	mean cIMT
Total Lipids in HDL	rs2298428	22	T	C	0.1826	-0.0390	0.0048	9.00E-17	0.0006	65.6979	mean cIMT
Total Lipids in HDL	rs2642438	1	G	A	0.7038	0.0359	0.0041	4.20E-20	0.0007	78.1736	mean cIMT
Total Lipids in HDL	rs267738	1	G	T	0.2196	0.0293	0.0045	9.00E-12	0.0004	42.9155	mean cIMT
Total Lipids in HDL	rs2740488	9	C	A	0.2653	-0.0457	0.0042	1.20E-28	0.0010	117.5010	mean cIMT
Total Lipids in HDL	rs2792735	10	A	G	0.7203	-0.0413	0.0041	9.30E-25	0.0009	100.0133	mean cIMT
Total Lipids in HDL	rs28818616	3	C	T	0.3498	-0.0212	0.0039	3.00E-08	0.0003	29.3643	mean cIMT
Total Lipids in HDL	rs2925979	16	C	T	0.6989	0.0324	0.0040	3.20E-17	0.0006	64.7919	mean cIMT

Total Lipids in HDL	rs2978615	19	T	C	0.4902	-0.0225	0.0037	9.80E-10	0.0003	36.6070	mean cIMT
Total Lipids in HDL	rs34663616	15	A	C	0.1377	0.0416	0.0055	7.60E-15	0.0005	57.2338	mean cIMT
Total Lipids in HDL	rs34707604	4	C	T	0.2585	0.0265	0.0045	2.30E-10	0.0003	34.9814	mean cIMT
Total Lipids in HDL	rs34955778	16	C	T	0.4200	-0.0216	0.0038	4.60E-09	0.0003	33.2876	mean cIMT
Total Lipids in HDL	rs35184771	11	T	G	0.3528	-0.0372	0.0039	9.40E-22	0.0008	92.1690	mean cIMT
Total Lipids in HDL	rs3735687	7	G	A	0.4228	-0.0265	0.0038	6.30E-12	0.0004	49.4728	mean cIMT
Total Lipids in HDL	rs3764261	16	A	C	0.3244	0.1899	0.0039	1.00E-200	0.0197	2312.3396	mean cIMT
Total Lipids in HDL	rs3768321	1	T	G	0.1965	-0.0386	0.0047	1.10E-17	0.0006	68.0304	mean cIMT
Total Lipids in HDL	rs41272663	2	A	C	0.2640	0.0244	0.0042	6.50E-09	0.0003	33.7946	mean cIMT
Total Lipids in HDL	rs4240624	8	A	G	0.9092	0.0923	0.0065	1.10E-46	0.0018	204.3352	mean cIMT
Total Lipids in HDL	rs4330777	16	A	G	0.4759	-0.0192	0.0037	4.60E-08	0.0002	26.7603	mean cIMT
Total Lipids in HDL	rs4759375	12	T	C	0.0849	0.0386	0.0068	4.70E-08	0.0003	31.8877	mean cIMT
Total Lipids in HDL	rs4846921	1	A	G	0.6132	0.0494	0.0038	2.50E-40	0.0015	168.4424	mean cIMT
Total Lipids in HDL	rs5167	19	G	T	0.3523	0.0497	0.0039	2.20E-38	0.0014	164.0338	mean cIMT
Total Lipids in HDL	rs599839	1	A	G	0.7721	-0.0270	0.0044	3.60E-10	0.0003	37.4171	mean cIMT
Total Lipids in HDL	rs6018652	20	A	G	0.7925	0.0373	0.0046	3.60E-17	0.0006	65.8524	mean cIMT
Total Lipids in HDL	rs6073958	20	C	T	0.1987	-0.0595	0.0047	1.10E-36	0.0014	163.2217	mean cIMT
Total Lipids in HDL	rs60847460	10	T	C	0.1433	-0.0377	0.0053	1.50E-12	0.0004	50.2639	mean cIMT
Total Lipids in HDL	rs61805076	1	C	T	0.3341	-0.0289	0.0039	4.90E-14	0.0005	54.3242	mean cIMT
Total Lipids in HDL	rs61941676	12	A	C	0.1272	-0.0601	0.0057	1.20E-26	0.0010	110.8668	mean cIMT
Total Lipids in HDL	rs62101704	18	A	G	0.0137	-0.0889	0.0163	7.00E-09	0.0003	29.9371	mean cIMT
Total Lipids in HDL	rs638714	1	T	G	0.3459	-0.0382	0.0039	4.00E-23	0.0008	95.3289	mean cIMT
Total Lipids in HDL	rs6509173	19	G	A	0.7937	-0.0300	0.0046	2.00E-10	0.0004	42.2062	mean cIMT
Total Lipids in HDL	rs6589565	11	G	A	0.9322	0.0506	0.0074	4.20E-13	0.0004	46.9046	mean cIMT
Total Lipids in HDL	rs676210	2	A	G	0.2058	0.0592	0.0046	5.50E-38	0.0015	167.6093	mean cIMT
Total Lipids in HDL	rs686030	9	A	C	0.8595	0.0458	0.0053	1.80E-18	0.0006	73.5569	mean cIMT
Total Lipids in HDL	rs7241918	18	T	G	0.8229	0.0902	0.0049	6.10E-79	0.0030	342.6918	mean cIMT
Total Lipids in HDL	rs72836561	17	T	C	0.0315	-0.1351	0.0106	5.50E-39	0.0014	161.7294	mean cIMT

Total Lipids in HDL	rs7308864	12	G	A	0.5233	0.0311	0.0037	1.30E-17	0.0006	70.3728	mean cIMT
Total Lipids in HDL	rs73632745	11	T	C	0.0737	-0.0447	0.0071	1.10E-09	0.0003	39.5869	mean cIMT
Total Lipids in HDL	rs737337	19	C	T	0.0768	-0.0685	0.0070	5.60E-23	0.0008	96.7460	mean cIMT
Total Lipids in HDL	rs75714888	16	A	C	0.0216	-0.0707	0.0128	3.70E-08	0.0003	30.5131	mean cIMT
Total Lipids in HDL	rs75911530	16	A	G	0.0329	-0.1442	0.0109	2.10E-42	0.0015	174.8114	mean cIMT
Total Lipids in HDL	rs77960347	18	G	A	0.0132	0.3034	0.0162	4.00E-81	0.0030	350.7393	mean cIMT
Total Lipids in HDL	rs7810507	7	A	G	0.2811	-0.0254	0.0041	5.00E-10	0.0003	38.0946	mean cIMT
Total Lipids in HDL	rs7956099	12	C	T	0.4750	-0.0214	0.0037	4.80E-09	0.0003	32.8098	mean cIMT
Total Lipids in HDL	rs8058512	16	T	C	0.7240	-0.0252	0.0042	1.20E-09	0.0003	36.7613	mean cIMT
Total Lipids in HDL	rs838876	12	G	A	0.6814	-0.0410	0.0040	3.50E-27	0.0009	103.0819	mean cIMT
Total Lipids in HDL	rs907866	2	A	G	0.4432	-0.0295	0.0037	2.50E-15	0.0005	61.8200	mean cIMT
Total Lipids in HDL	rs9491697	6	G	A	0.4636	-0.0237	0.0037	1.60E-10	0.0004	40.2941	mean cIMT
Total Lipids in HDL	rs967645	17	T	C	0.5165	-0.0258	0.0037	1.70E-11	0.0004	48.3023	mean cIMT
Total Lipids in HDL	rs9687833	5	A	G	0.2070	-0.0296	0.0046	4.50E-11	0.0004	41.7951	mean cIMT
Total Lipids in Medium HDL	rs1047891	2	A	C	0.3150	-0.0249	0.0041	2.60E-09	0.0003	36.2238	mean cIMT
Total Lipids in Medium HDL	rs1065853	19	T	G	0.0806	0.1619	0.0071	2.30E-121	0.0045	524.3185	mean cIMT
Total Lipids in Medium HDL	rs1077835	15	G	A	0.2197	0.1016	0.0046	9.71E-111	0.0042	480.1325	mean cIMT
Total Lipids in Medium HDL	rs11039238	11	C	T	0.3222	-0.0355	0.0041	3.90E-18	0.0007	74.8717	mean cIMT
Total Lipids in Medium HDL	rs112001035	17	A	G	0.0597	-0.0509	0.0082	1.80E-09	0.0003	38.0946	mean cIMT
Total Lipids in Medium HDL	rs112495680	6	G	A	0.1334	-0.0345	0.0057	5.20E-10	0.0003	37.0955	mean cIMT
Total Lipids in Medium HDL	rs11601507	11	A	C	0.0692	-0.0390	0.0075	3.60E-08	0.0002	27.3264	mean cIMT
Total Lipids in Medium HDL	rs117310449	19	T	C	0.0120	-0.0982	0.0177	1.90E-08	0.0003	30.6435	mean cIMT
Total Lipids in Medium HDL	rs117687565	18	T	C	0.0120	0.1382	0.0182	1.10E-13	0.0005	57.5058	mean cIMT
Total Lipids in Medium HDL	rs11789603	9	T	C	0.1088	0.0504	0.0062	1.40E-15	0.0006	66.6595	mean cIMT
Total Lipids in Medium HDL	rs1260326	2	C	T	0.6040	-0.0457	0.0039	3.30E-30	0.0012	135.8893	mean cIMT
Total Lipids in Medium HDL	rs12904367	15	A	G	0.1413	0.0305	0.0056	4.80E-08	0.0003	29.5983	mean cIMT
Total Lipids in Medium HDL	rs12976739	19	A	G	0.3962	0.0246	0.0039	8.90E-11	0.0003	38.9533	mean cIMT
Total Lipids in Medium HDL	rs13107325	4	T	C	0.0743	-0.0651	0.0073	1.10E-19	0.0007	79.2408	mean cIMT

Total Lipids in Medium HDL	rs1321257	1	A	G	0.6132	0.0506	0.0039	5.10E-39	0.0014	165.2642	mean cIMT
Total Lipids in Medium HDL	rs139915535	8	G	A	0.0180	-0.1229	0.0145	9.50E-19	0.0006	72.1764	mean cIMT
Total Lipids in Medium HDL	rs140168704	10	T	C	0.1535	-0.0286	0.0053	1.80E-08	0.0002	28.7364	mean cIMT
Total Lipids in Medium HDL	rs140584594	1	G	A	0.7301	0.0392	0.0043	2.00E-20	0.0007	83.3199	mean cIMT
Total Lipids in Medium HDL	rs148063610	10	C	CAAATAAAT	0.7634	-0.0326	0.0046	1.10E-12	0.0004	50.1339	mean cIMT
Total Lipids in Medium HDL	rs15285	8	T	C	0.2865	0.0818	0.0042	8.50E-85	0.0032	371.5352	mean cIMT
Total Lipids in Medium HDL	rs1800961	20	T	C	0.0302	-0.1128	0.0112	2.10E-23	0.0009	101.5093	mean cIMT
Total Lipids in Medium HDL	rs193084249	1	G	A	0.0234	-0.1021	0.0130	2.10E-15	0.0005	62.1249	mean cIMT
Total Lipids in Medium HDL	rs2043085	15	C	T	0.6127	-0.0977	0.0039	1.70E-139	0.0053	617.6311	mean cIMT
Total Lipids in Medium HDL	rs2072113	11	T	C	0.1157	-0.0450	0.0060	7.60E-15	0.0005	56.5464	mean cIMT
Total Lipids in Medium HDL	rs2176040	2	G	A	0.6467	-0.0252	0.0040	1.30E-11	0.0003	39.7909	mean cIMT
Total Lipids in Medium HDL	rs2236252	20	T	C	0.1670	-0.0322	0.0051	6.50E-11	0.0003	39.2638	mean cIMT
Total Lipids in Medium HDL	rs2298428	22	T	C	0.1826	-0.0422	0.0050	4.10E-18	0.0006	72.3908	mean cIMT
Total Lipids in Medium HDL	rs235314	21	T	C	0.5327	-0.0214	0.0038	4.50E-08	0.0003	31.1386	mean cIMT
Total Lipids in Medium HDL	rs2569550	19	C	T	0.5933	-0.0270	0.0039	5.40E-12	0.0004	47.4643	mean cIMT
Total Lipids in Medium HDL	rs2642438	1	G	A	0.7038	0.0348	0.0042	1.90E-17	0.0006	68.7232	mean cIMT
Total Lipids in Medium HDL	rs267738	1	G	T	0.2196	0.0386	0.0046	5.50E-18	0.0006	69.6858	mean cIMT
Total Lipids in Medium HDL	rs2740488	9	C	A	0.2653	-0.0378	0.0044	3.40E-19	0.0007	75.3683	mean cIMT
Total Lipids in Medium HDL	rs2781752	19	A	G	0.3182	0.0230	0.0041	4.30E-09	0.0003	30.6207	mean cIMT
Total Lipids in Medium HDL	rs2792735	10	A	G	0.7203	-0.0386	0.0043	1.20E-20	0.0007	81.8818	mean cIMT
Total Lipids in Medium HDL	rs28818616	3	C	T	0.3498	-0.0216	0.0040	4.80E-08	0.0002	28.6445	mean cIMT
Total Lipids in Medium HDL	rs2925979	16	C	T	0.6989	0.0276	0.0042	2.90E-12	0.0004	43.7755	mean cIMT
Total Lipids in Medium HDL	rs35135293	2	T	C	0.5167	-0.0278	0.0039	9.30E-14	0.0005	51.8979	mean cIMT
Total Lipids in Medium HDL	rs3764261	16	A	C	0.3244	0.1599	0.0041	1.00E-200	0.0131	1528.8982	mean cIMT
Total Lipids in Medium HDL	rs3768321	1	T	G	0.1965	-0.0377	0.0048	9.00E-16	0.0005	60.9568	mean cIMT
Total Lipids in Medium HDL	rs3795269	1	A	C	0.4386	0.0219	0.0039	7.00E-09	0.0003	32.3440	mean cIMT
Total Lipids in Medium HDL	rs4239651	20	C	T	0.7942	0.0361	0.0047	3.10E-15	0.0005	57.9372	mean cIMT
Total Lipids in Medium HDL	rs4240624	8	A	G	0.9092	0.1005	0.0067	7.30E-52	0.0020	226.5395	mean cIMT

Total Lipids in Medium HDL	rs4656292	1	G	A	0.6196	0.0239	0.0040	7.40E-11	0.0003	36.5453	mean cIMT
Total Lipids in Medium HDL	rs4969141	17	T	C	0.4895	0.0207	0.0038	3.20E-08	0.0003	28.9608	mean cIMT
Total Lipids in Medium HDL	rs583104	1	T	G	0.7734	-0.0510	0.0046	1.90E-30	0.0011	124.5911	mean cIMT
Total Lipids in Medium HDL	rs60847460	10	T	C	0.1433	-0.0376	0.0055	1.50E-11	0.0004	46.9188	mean cIMT
Total Lipids in Medium HDL	rs61805076	1	C	T	0.3341	-0.0260	0.0041	6.60E-11	0.0004	41.1269	mean cIMT
Total Lipids in Medium HDL	rs61941676	12	A	C	0.1272	-0.0548	0.0059	1.40E-20	0.0007	86.3320	mean cIMT
Total Lipids in Medium HDL	rs62101704	18	A	G	0.0137	-0.0893	0.0168	4.50E-08	0.0002	28.2418	mean cIMT
Total Lipids in Medium HDL	rs638714	1	T	G	0.3459	-0.0507	0.0040	6.30E-37	0.0014	157.2493	mean cIMT
Total Lipids in Medium HDL	rs6511720	19	T	G	0.1198	0.0493	0.0059	7.70E-18	0.0006	69.9979	mean cIMT
Total Lipids in Medium HDL	rs6606717	12	C	A	0.5232	0.0309	0.0038	4.30E-16	0.0006	64.9373	mean cIMT
Total Lipids in Medium HDL	rs676210	2	A	G	0.2058	0.0611	0.0047	2.10E-37	0.0015	167.1657	mean cIMT
Total Lipids in Medium HDL	rs686030	9	A	C	0.8595	0.0346	0.0055	1.70E-10	0.0003	39.3165	mean cIMT
Total Lipids in Medium HDL	rs72836561	17	T	C	0.0315	-0.1254	0.0110	2.90E-31	0.0011	130.4524	mean cIMT
Total Lipids in Medium HDL	rs737338	19	T	C	0.0352	-0.1235	0.0104	7.20E-34	0.0012	141.0048	mean cIMT
Total Lipids in Medium HDL	rs75911530	16	A	G	0.0329	-0.1346	0.0113	3.30E-34	0.0012	142.2489	mean cIMT
Total Lipids in Medium HDL	rs77960347	18	G	A	0.0132	0.3272	0.0167	4.10E-87	0.0033	381.9758	mean cIMT
Total Lipids in Medium HDL	rs7956099	12	C	T	0.4750	-0.0244	0.0039	1.20E-10	0.0003	39.9241	mean cIMT
Total Lipids in Medium HDL	rs838876	12	G	A	0.6814	-0.0366	0.0042	6.00E-20	0.0007	76.9986	mean cIMT
Total Lipids in Medium HDL	rs9304381	18	T	C	0.8184	0.0967	0.0050	1.40E-85	0.0033	377.6504	mean cIMT
Total Lipids in Medium HDL	rs9471972	6	A	G	0.5362	0.0274	0.0038	1.20E-12	0.0004	50.7023	mean cIMT
Total Lipids in Medium HDL	rs967645	17	T	C	0.5165	-0.0312	0.0038	5.80E-15	0.0006	66.1093	mean cIMT
Total Lipids in Large HDL	rs1006656	17	A	G	0.8810	0.0304	0.0056	1.70E-08	0.0003	29.4149	mean cIMT
Total Lipids in Large HDL	rs1024137	2	T	G	0.6465	-0.0391	0.0038	7.00E-28	0.0009	107.1666	mean cIMT
Total Lipids in Large HDL	rs10468017	15	T	C	0.2959	0.1623	0.0040	1.00E-200	0.0143	1673.1451	mean cIMT
Total Lipids in Large HDL	rs1054852	12	G	A	0.3775	0.0312	0.0039	1.80E-16	0.0005	63.2730	mean cIMT
Total Lipids in Large HDL	rs1057208	20	T	C	0.1863	-0.1599	0.0047	1.00E-200	0.0101	1176.4336	mean cIMT
Total Lipids in Large HDL	rs11045171	12	G	A	0.1974	0.0278	0.0046	7.00E-10	0.0003	36.7877	mean cIMT
Total Lipids in Large HDL	rs11057469	12	A	G	0.4239	0.0234	0.0037	3.00E-09	0.0003	39.6561	mean cIMT

Total Lipids in Large HDL	rs11057692	12	G	A	0.2363	-0.0230	0.0043	1.90E-08	0.0002	28.4820	mean cIMT
Total Lipids in Large HDL	rs11065991	12	T	C	0.4159	-0.0189	0.0037	7.40E-09	0.0002	26.4497	mean cIMT
Total Lipids in Large HDL	rs112001035	17	A	G	0.0597	-0.0459	0.0078	6.80E-09	0.0003	34.5829	mean cIMT
Total Lipids in Large HDL	rs112875651	8	A	G	0.3924	0.0303	0.0038	3.90E-18	0.0006	64.8194	mean cIMT
Total Lipids in Large HDL	rs1139490	4	T	C	0.6970	-0.0239	0.0039	8.10E-10	0.0003	36.6315	mean cIMT
Total Lipids in Large HDL	rs11429307	5	G	GT	0.8085	0.0447	0.0046	1.10E-23	0.0008	93.4382	mean cIMT
Total Lipids in Large HDL	rs11434143	8	G	GT	0.8108	-0.0280	0.0047	4.70E-10	0.0003	35.4961	mean cIMT
Total Lipids in Large HDL	rs116843064	19	A	G	0.0199	0.1868	0.0130	4.10E-48	0.0018	206.0099	mean cIMT
Total Lipids in Large HDL	rs11789603	9	T	C	0.1088	0.0421	0.0058	2.70E-12	0.0005	52.0745	mean cIMT
Total Lipids in Large HDL	rs12510382	4	G	A	0.4599	0.0213	0.0037	2.40E-09	0.0003	33.5953	mean cIMT
Total Lipids in Large HDL	rs1260326	2	C	T	0.6040	0.0260	0.0037	4.30E-13	0.0004	49.1605	mean cIMT
Total Lipids in Large HDL	rs12786130	11	T	C	0.7618	0.0228	0.0043	4.80E-08	0.0002	28.5296	mean cIMT
Total Lipids in Large HDL	rs13107325	4	T	C	0.0743	-0.0692	0.0069	4.70E-25	0.0009	100.2262	mean cIMT
Total Lipids in Large HDL	rs13118477	4	A	G	0.3904	-0.0210	0.0037	4.80E-08	0.0003	31.7969	mean cIMT
Total Lipids in Large HDL	rs1319424	9	A	G	0.7431	0.0218	0.0042	4.90E-08	0.0002	27.4692	mean cIMT
Total Lipids in Large HDL	rs13389219	2	T	C	0.3925	0.0360	0.0037	1.00E-23	0.0008	94.1753	mean cIMT
Total Lipids in Large HDL	rs142265900	12	A	AGAT	0.9424	-0.0508	0.0078	4.30E-11	0.0004	42.0475	mean cIMT
Total Lipids in Large HDL	rs145391587	8	C	A	0.1003	0.1350	0.0060	5.70E-118	0.0044	503.3005	mean cIMT
Total Lipids in Large HDL	rs1457489	18	A	G	0.2646	-0.0235	0.0041	6.20E-09	0.0003	32.5329	mean cIMT
Total Lipids in Large HDL	rs147627829	6	A	G	0.0441	-0.0616	0.0089	6.40E-12	0.0004	48.1603	mean cIMT
Total Lipids in Large HDL	rs148063610	10	C	CAAATAAAT	0.7634	-0.0323	0.0044	3.80E-14	0.0005	54.9642	mean cIMT
Total Lipids in Large HDL	rs150224153	20	T	C	0.0292	-0.0710	0.0110	5.40E-11	0.0004	41.3231	mean cIMT
Total Lipids in Large HDL	rs1546954	1	G	T	0.6127	0.0450	0.0037	2.50E-36	0.0013	146.7251	mean cIMT
Total Lipids in Large HDL	rs1560390	15	C	T	0.2197	-0.0669	0.0044	1.80E-58	0.0020	231.9292	mean cIMT
Total Lipids in Large HDL	rs174576	11	A	C	0.3500	-0.0774	0.0038	4.20E-101	0.0036	416.2459	mean cIMT
Total Lipids in Large HDL	rs187459727	20	T	C	0.0369	0.0526	0.0099	2.90E-08	0.0002	28.3404	mean cIMT
Total Lipids in Large HDL	rs200644264	11	CG	C	0.7885	-0.0417	0.0044	1.90E-21	0.0008	88.4626	mean cIMT
Total Lipids in Large HDL	rs2066714	9	C	T	0.1289	0.0312	0.0054	2.10E-09	0.0003	33.4209	mean cIMT

Total Lipids in Large HDL	rs2229357	12	A	G	0.2409	0.0279	0.0042	5.20E-12	0.0004	43.4726	mean cIMT
Total Lipids in Large HDL	rs2307111	5	C	T	0.3966	0.0244	0.0037	5.50E-11	0.0004	43.4258	mean cIMT
Total Lipids in Large HDL	rs2642438	1	G	A	0.7038	0.0327	0.0040	1.10E-17	0.0006	67.7212	mean cIMT
Total Lipids in Large HDL	rs2737205	8	C	T	0.5614	0.0284	0.0037	2.70E-14	0.0005	60.0777	mean cIMT
Total Lipids in Large HDL	rs2740488	9	C	A	0.2653	-0.0378	0.0041	2.40E-21	0.0007	84.4456	mean cIMT
Total Lipids in Large HDL	rs2925979	16	C	T	0.6989	0.0360	0.0039	3.10E-21	0.0007	83.8820	mean cIMT
Total Lipids in Large HDL	rs3092498	20	C	T	0.5174	0.0210	0.0037	3.90E-08	0.0003	33.1361	mean cIMT
Total Lipids in Large HDL	rs333947	1	A	G	0.1501	-0.0298	0.0051	8.80E-10	0.0003	34.7114	mean cIMT
Total Lipids in Large HDL	rs34060476	7	G	A	0.1348	0.0600	0.0053	9.80E-31	0.0011	127.3650	mean cIMT
Total Lipids in Large HDL	rs34695955	17	G	T	0.0795	-0.0389	0.0067	1.40E-08	0.0003	33.5911	mean cIMT
Total Lipids in Large HDL	rs35135293	2	T	C	0.5167	-0.0214	0.0036	5.50E-10	0.0003	34.6433	mean cIMT
Total Lipids in Large HDL	rs368166328	4	C	CT	0.7599	-0.0276	0.0050	7.90E-09	0.0003	30.3646	mean cIMT
Total Lipids in Large HDL	rs3735687	7	G	A	0.4228	-0.0335	0.0037	9.30E-20	0.0007	82.4226	mean cIMT
Total Lipids in Large HDL	rs3764261	16	A	C	0.3244	0.2104	0.0039	1.00E-200	0.0252	2979.3349	mean cIMT
Total Lipids in Large HDL	rs390387	22	G	A	0.8127	0.0280	0.0048	1.80E-09	0.0003	34.5048	mean cIMT
Total Lipids in Large HDL	rs4078216	12	A	G	0.2409	0.0258	0.0042	1.80E-08	0.0003	36.9749	mean cIMT
Total Lipids in Large HDL	rs4418728	10	T	G	0.4507	0.0251	0.0036	5.00E-12	0.0004	47.6132	mean cIMT
Total Lipids in Large HDL	rs4795386	17	G	A	0.7230	0.0217	0.0041	8.60E-09	0.0002	28.1942	mean cIMT
Total Lipids in Large HDL	rs488490	15	A	C	0.8526	-0.1558	0.0052	1.00E-200	0.0078	909.3506	mean cIMT
Total Lipids in Large HDL	rs5030789	15	G	A	0.5673	0.0209	0.0037	3.30E-09	0.0003	32.5071	mean cIMT
Total Lipids in Large HDL	rs5082	1	A	G	0.6207	-0.0216	0.0037	3.30E-08	0.0003	33.1844	mean cIMT
Total Lipids in Large HDL	rs5167	19	G	T	0.3523	0.0419	0.0038	1.50E-29	0.0011	122.0764	mean cIMT
Total Lipids in Large HDL	rs55707100	15	T	C	0.0259	-0.0684	0.0114	6.20E-10	0.0003	36.0807	mean cIMT
Total Lipids in Large HDL	rs55714927	17	T	C	0.1910	0.0251	0.0046	1.00E-08	0.0003	29.7173	mean cIMT
Total Lipids in Large HDL	rs57912727	3	C	A	0.1367	-0.0304	0.0053	1.30E-09	0.0003	32.8149	mean cIMT
Total Lipids in Large HDL	rs58388121	1	T	TAA	0.6288	-0.0232	0.0038	2.70E-10	0.0003	37.1266	mean cIMT
Total Lipids in Large HDL	rs59104589	2	T	C	0.3588	0.0245	0.0038	9.60E-13	0.0004	41.9371	mean cIMT
Total Lipids in Large HDL	rs60847460	10	T	C	0.1433	-0.0343	0.0052	2.30E-11	0.0004	43.5244	mean cIMT

Total Lipids in Large HDL	rs61781392	1	A	C	0.2167	-0.0310	0.0044	8.60E-14	0.0004	49.4874	mean cIMT
Total Lipids in Large HDL	rs6498540	16	G	A	0.3021	-0.0296	0.0039	7.10E-15	0.0005	56.7316	mean cIMT
Total Lipids in Large HDL	rs654689	6	A	G	0.5906	0.0193	0.0037	3.70E-08	0.0002	27.3379	mean cIMT
Total Lipids in Large HDL	rs6694102	1	A	G	0.3209	-0.0335	0.0039	3.10E-18	0.0006	74.6491	mean cIMT
Total Lipids in Large HDL	rs676210	2	A	G	0.2058	0.0598	0.0045	2.40E-40	0.0016	178.8272	mean cIMT
Total Lipids in Large HDL	rs686030	9	A	C	0.8595	0.0485	0.0052	1.90E-21	0.0007	86.3381	mean cIMT
Total Lipids in Large HDL	rs7012814	8	A	G	0.4743	0.0340	0.0037	3.90E-20	0.0008	86.5309	mean cIMT
Total Lipids in Large HDL	rs705379	7	A	G	0.4775	-0.0251	0.0037	1.20E-12	0.0004	46.9354	mean cIMT
Total Lipids in Large HDL	rs7136506	12	C	T	0.2158	-0.0445	0.0045	8.10E-24	0.0009	98.2708	mean cIMT
Total Lipids in Large HDL	rs7241918	18	T	G	0.8229	0.0621	0.0048	7.70E-40	0.0015	169.7199	mean cIMT
Total Lipids in Large HDL	rs72555385	7	G	A	0.0493	-0.0531	0.0084	4.40E-10	0.0003	39.8059	mean cIMT
Total Lipids in Large HDL	rs72836561	17	T	C	0.0315	-0.1380	0.0104	2.20E-43	0.0015	176.6151	mean cIMT
Total Lipids in Large HDL	rs72934503	6	G	A	0.4514	0.0246	0.0037	3.70E-11	0.0004	43.7366	mean cIMT
Total Lipids in Large HDL	rs7308864	12	G	A	0.5233	0.0264	0.0036	4.70E-14	0.0005	53.0632	mean cIMT
Total Lipids in Large HDL	rs737337	19	C	T	0.0768	-0.0529	0.0068	9.40E-15	0.0005	60.2857	mean cIMT
Total Lipids in Large HDL	rs7475340	10	G	A	0.5156	-0.0184	0.0036	4.20E-08	0.0002	25.4755	mean cIMT
Total Lipids in Large HDL	rs75460349	1	C	A	0.0233	-0.0856	0.0122	7.30E-12	0.0004	49.4196	mean cIMT
Total Lipids in Large HDL	rs75627662	19	T	C	0.2061	-0.0325	0.0045	1.10E-13	0.0005	52.6259	mean cIMT
Total Lipids in Large HDL	rs75714888	16	A	C	0.0216	-0.0806	0.0125	2.70E-10	0.0004	41.7117	mean cIMT
Total Lipids in Large HDL	rs75911530	16	A	G	0.0329	-0.1427	0.0106	3.40E-43	0.0016	179.7497	mean cIMT
Total Lipids in Large HDL	rs77516617	15	A	G	0.0528	-0.0503	0.0082	4.80E-10	0.0003	37.9375	mean cIMT
Total Lipids in Large HDL	rs77960347	18	G	A	0.0132	0.2147	0.0158	3.00E-43	0.0016	183.7369	mean cIMT
Total Lipids in Large HDL	rs78058190	2	A	G	0.0501	-0.0569	0.0094	6.30E-09	0.0003	36.7890	mean cIMT
Total Lipids in Large HDL	rs7810507	7	A	G	0.2811	-0.0313	0.0040	2.20E-14	0.0005	60.4711	mean cIMT
Total Lipids in Large HDL	rs7924036	10	T	G	0.5042	0.0201	0.0036	4.20E-09	0.0003	30.7798	mean cIMT
Total Lipids in Large HDL	rs8058512	16	T	C	0.7240	-0.0247	0.0041	1.60E-09	0.0003	37.1435	mean cIMT
Total Lipids in Large HDL	rs921919	12	A	G	0.6695	-0.0429	0.0039	9.50E-31	0.0010	118.6573	mean cIMT
Total Lipids in Large HDL	rs9265113	6	T	C	0.4076	-0.0284	0.0040	3.40E-13	0.0005	51.8103	mean cIMT

Total Lipids in Large HDL	rs9491697	6	G	A	0.4636	-0.0290	0.0037	1.80E-16	0.0005	63.0083	mean cIMT
Total Lipids in Large HDL	rs9955201	18	A	G	0.0338	0.0714	0.0101	1.00E-12	0.0004	49.4892	mean cIMT
Total Lipids in Large HDL	rs998584	6	A	C	0.4823	-0.0344	0.0036	5.90E-22	0.0008	89.9556	mean cIMT
HDL Cholesterol	rs10268632	7	C	A	0.5118	0.0217	0.0037	2.00E-09	0.0003	34.3767	mean cIMT
HDL Cholesterol	rs10468017	15	T	C	0.2959	0.1015	0.0040	3.00E-145	0.0054	629.6227	mean cIMT
HDL Cholesterol	rs1047891	2	A	C	0.3150	-0.0233	0.0040	1.30E-08	0.0003	34.3197	mean cIMT
HDL Cholesterol	rs1054852	12	G	A	0.3775	0.0325	0.0040	1.90E-17	0.0006	66.0723	mean cIMT
HDL Cholesterol	rs11057692	12	G	A	0.2363	-0.0255	0.0044	5.50E-10	0.0003	33.8524	mean cIMT
HDL Cholesterol	rs112001035	17	A	G	0.0597	-0.0574	0.0079	5.80E-13	0.0005	52.3538	mean cIMT
HDL Cholesterol	rs112945592	6	A	G	0.1596	-0.0276	0.0051	3.90E-08	0.0003	29.5359	mean cIMT
HDL Cholesterol	rs11429307	5	G	GT	0.8085	0.0403	0.0047	9.00E-18	0.0006	73.5833	mean cIMT
HDL Cholesterol	rs11434143	8	G	GT	0.8108	-0.0261	0.0048	6.00E-09	0.0003	29.6609	mean cIMT
HDL Cholesterol	rs116843064	19	A	G	0.0199	0.2140	0.0132	9.70E-62	0.0023	261.3838	mean cIMT
HDL Cholesterol	rs116978226	11	A	C	0.0332	0.0647	0.0104	9.80E-11	0.0003	38.7555	mean cIMT
HDL Cholesterol	rs11751347	6	T	C	0.1023	-0.0547	0.0061	2.60E-20	0.0007	79.8307	mean cIMT
HDL Cholesterol	rs11789603	9	T	C	0.1088	0.0525	0.0059	1.10E-17	0.0007	78.1990	mean cIMT
HDL Cholesterol	rs12295878	11	T	C	0.1403	0.0333	0.0053	1.50E-09	0.0003	39.3290	mean cIMT
HDL Cholesterol	rs12453682	17	T	C	0.6936	0.0212	0.0040	1.80E-08	0.0002	27.7073	mean cIMT
HDL Cholesterol	rs12608026	18	G	T	0.0424	0.0724	0.0092	2.40E-15	0.0005	61.3690	mean cIMT
HDL Cholesterol	rs12786130	11	T	C	0.7618	0.0264	0.0044	4.60E-10	0.0003	36.7469	mean cIMT
HDL Cholesterol	rs13107325	4	T	C	0.0743	-0.0745	0.0070	1.00E-27	0.0010	112.3611	mean cIMT
HDL Cholesterol	rs13389219	2	T	C	0.3925	0.0342	0.0038	1.20E-20	0.0007	81.8329	mean cIMT
HDL Cholesterol	rs1358980	6	T	C	0.4826	-0.0324	0.0037	3.80E-18	0.0007	76.0690	mean cIMT
HDL Cholesterol	rs140584594	1	G	A	0.7301	0.0245	0.0041	2.70E-09	0.0003	35.0744	mean cIMT
HDL Cholesterol	rs142265900	12	A	AGAT	0.9424	-0.0531	0.0080	4.90E-11	0.0004	44.2635	mean cIMT
HDL Cholesterol	rs145391587	8	C	A	0.1003	0.1572	0.0061	5.20E-151	0.0057	659.6240	mean cIMT
HDL Cholesterol	rs147233090	15	T	C	0.0246	-0.0768	0.0120	5.60E-11	0.0004	41.0052	mean cIMT
HDL Cholesterol	rs148063610	10	C	CAAATAAAT	0.7634	-0.0335	0.0044	1.50E-14	0.0005	57.1872	mean cIMT

HDL Cholesterol	rs150224153	20	T	C	0.0292	-0.0903	0.0112	1.20E-15	0.0006	64.6245	mean cIMT
HDL Cholesterol	rs1560390	15	C	T	0.2197	-0.0399	0.0045	3.80E-22	0.0007	79.0830	mean cIMT
HDL Cholesterol	rs1761457	19	G	A	0.3267	0.0279	0.0039	6.90E-14	0.0004	50.1051	mean cIMT
HDL Cholesterol	rs17696736	12	G	A	0.4302	-0.0218	0.0037	7.20E-11	0.0003	34.3264	mean cIMT
HDL Cholesterol	rs2066714	9	C	T	0.1289	0.0335	0.0055	1.40E-10	0.0003	37.2032	mean cIMT
HDL Cholesterol	rs2229357	12	A	G	0.2409	0.0291	0.0043	2.90E-12	0.0004	45.7414	mean cIMT
HDL Cholesterol	rs2245221	8	A	G	0.5590	0.0309	0.0037	1.50E-15	0.0006	68.3631	mean cIMT
HDL Cholesterol	rs2298428	22	T	C	0.1826	-0.0360	0.0048	5.20E-15	0.0005	56.5508	mean cIMT
HDL Cholesterol	rs2302263	11	T	C	0.0881	-0.0360	0.0065	2.90E-08	0.0003	30.4078	mean cIMT
HDL Cholesterol	rs2307111	5	C	T	0.3966	0.0246	0.0038	1.30E-10	0.0004	42.4983	mean cIMT
HDL Cholesterol	rs2494748	14	T	C	0.6162	-0.0201	0.0038	1.50E-08	0.0002	27.9697	mean cIMT
HDL Cholesterol	rs2642438	1	G	A	0.7038	0.0359	0.0040	7.70E-20	0.0007	79.1405	mean cIMT
HDL Cholesterol	rs267738	1	G	T	0.2196	0.0272	0.0045	1.70E-10	0.0003	37.2057	mean cIMT
HDL Cholesterol	rs2740488	9	C	A	0.2653	-0.0440	0.0042	5.50E-27	0.0010	110.2983	mean cIMT
HDL Cholesterol	rs2792735	10	A	G	0.7203	-0.0404	0.0041	2.80E-24	0.0008	96.4080	mean cIMT
HDL Cholesterol	rs28818616	3	C	T	0.3498	-0.0203	0.0039	3.50E-08	0.0002	27.2744	mean cIMT
HDL Cholesterol	rs2925979	16	C	T	0.6989	0.0382	0.0040	2.60E-23	0.0008	91.1318	mean cIMT
HDL Cholesterol	rs2943650	2	T	C	0.6448	-0.0405	0.0038	2.20E-28	0.0010	110.8371	mean cIMT
HDL Cholesterol	rs2978615	19	T	C	0.4902	-0.0199	0.0037	4.90E-08	0.0003	28.8336	mean cIMT
HDL Cholesterol	rs34265539	6	A	ATT	0.3561	-0.0267	0.0039	1.70E-13	0.0004	47.1391	mean cIMT
HDL Cholesterol	rs35184771	11	T	G	0.3528	-0.0386	0.0039	1.00E-23	0.0009	100.6991	mean cIMT
HDL Cholesterol	rs35633876	2	T	G	0.4818	-0.0239	0.0037	4.20E-11	0.0004	41.5066	mean cIMT
HDL Cholesterol	rs3735687	7	G	A	0.4228	-0.0246	0.0038	1.90E-10	0.0004	43.1574	mean cIMT
HDL Cholesterol	rs3764261	16	A	C	0.3244	0.2082	0.0039	1.00E-200	0.0239	2814.4768	mean cIMT
HDL Cholesterol	rs3768321	1	T	G	0.1965	-0.0426	0.0046	7.90E-22	0.0007	84.1370	mean cIMT
HDL Cholesterol	rs4078216	12	A	G	0.2409	0.0259	0.0043	2.20E-08	0.0003	36.0844	mean cIMT
HDL Cholesterol	rs4240624	8	A	G	0.9092	0.0930	0.0064	2.70E-48	0.0018	209.7285	mean cIMT
HDL Cholesterol	rs4330777	16	A	G	0.4759	-0.0200	0.0037	1.20E-08	0.0003	29.2575	mean cIMT

HDL Cholesterol	rs4846921	1	A	G	0.6132	0.0487	0.0038	3.10E-40	0.0014	165.9587	mean cIMT
HDL Cholesterol	rs488490	15	A	C	0.8526	-0.0976	0.0053	7.80E-80	0.0030	343.0872	mean cIMT
HDL Cholesterol	rs4969141	17	T	C	0.4895	0.0228	0.0037	7.10E-10	0.0003	38.0103	mean cIMT
HDL Cholesterol	rs4985155	16	G	A	0.3349	-0.0239	0.0039	2.00E-10	0.0003	37.7906	mean cIMT
HDL Cholesterol	rs5030789	15	G	A	0.5673	0.0202	0.0037	4.00E-08	0.0003	29.3731	mean cIMT
HDL Cholesterol	rs5167	19	G	T	0.3523	0.0441	0.0039	3.00E-31	0.0011	130.5128	mean cIMT
HDL Cholesterol	rs57912727	3	C	A	0.1367	-0.0286	0.0054	3.60E-08	0.0002	28.1029	mean cIMT
HDL Cholesterol	rs59104589	2	T	C	0.3588	0.0203	0.0038	9.30E-09	0.0002	27.9310	mean cIMT
HDL Cholesterol	rs6018652	20	A	G	0.7925	0.0354	0.0046	5.90E-16	0.0005	60.0018	mean cIMT
HDL Cholesterol	rs6073958	20	C	T	0.1987	-0.0733	0.0046	4.90E-56	0.0022	250.6486	mean cIMT
HDL Cholesterol	rs60847460	10	T	C	0.1433	-0.0368	0.0053	4.20E-12	0.0004	48.4227	mean cIMT
HDL Cholesterol	rs61805076	1	C	T	0.3341	-0.0297	0.0039	1.10E-14	0.0005	57.8947	mean cIMT
HDL Cholesterol	rs62101705	18	T	C	0.0136	-0.0891	0.0162	5.30E-09	0.0003	30.2714	mean cIMT
HDL Cholesterol	rs676210	2	A	G	0.2058	0.0697	0.0045	1.40E-52	0.0020	234.5470	mean cIMT
HDL Cholesterol	rs686030	9	A	C	0.8595	0.0480	0.0053	4.50E-20	0.0007	81.6751	mean cIMT
HDL Cholesterol	rs688671	18	G	A	0.2671	-0.0244	0.0042	5.20E-09	0.0003	33.9129	mean cIMT
HDL Cholesterol	rs71205961	8	C	CTTTTTT	0.0890	-0.0423	0.0073	1.20E-08	0.0003	33.9714	mean cIMT
HDL Cholesterol	rs71336055	3	T	C	0.1197	0.0328	0.0057	7.00E-09	0.0003	33.2535	mean cIMT
HDL Cholesterol	rs7134375	12	A	C	0.4302	0.0233	0.0037	1.10E-10	0.0003	39.2677	mean cIMT
HDL Cholesterol	rs7136506	12	C	T	0.2158	-0.0406	0.0046	1.30E-19	0.0007	79.0553	mean cIMT
HDL Cholesterol	rs7241918	18	T	G	0.8229	0.0758	0.0048	3.70E-57	0.0021	244.6408	mean cIMT
HDL Cholesterol	rs72836561	17	T	C	0.0315	-0.1561	0.0106	1.50E-52	0.0019	218.3527	mean cIMT
HDL Cholesterol	rs7308864	12	G	A	0.5233	0.0263	0.0037	2.70E-13	0.0004	50.8790	mean cIMT
HDL Cholesterol	rs737337	19	C	T	0.0768	-0.0598	0.0069	2.90E-18	0.0006	74.4284	mean cIMT
HDL Cholesterol	rs75627662	19	T	C	0.2061	-0.0329	0.0046	1.00E-13	0.0005	51.8734	mean cIMT
HDL Cholesterol	rs75714888	16	A	C	0.0216	-0.0792	0.0127	9.40E-10	0.0003	38.8252	mean cIMT
HDL Cholesterol	rs75911530	16	A	G	0.0329	-0.1588	0.0108	1.10E-50	0.0019	214.8595	mean cIMT
HDL Cholesterol	rs77960347	18	G	A	0.0132	0.2535	0.0161	5.10E-58	0.0021	247.4685	mean cIMT

HDL Cholesterol	rs78058190	2	A	G	0.0501	-0.0624	0.0096	3.90E-10	0.0004	42.6661	mean cIMT
HDL Cholesterol	rs7810507	7	A	G	0.2811	-0.0302	0.0041	1.50E-13	0.0005	54.0836	mean cIMT
HDL Cholesterol	rs7845090	8	A	G	0.7094	0.0234	0.0041	1.60E-08	0.0003	32.8240	mean cIMT
HDL Cholesterol	rs8058512	16	T	C	0.7240	-0.0267	0.0041	1.40E-10	0.0004	41.8874	mean cIMT
HDL Cholesterol	rs921919	12	A	G	0.6695	-0.0418	0.0040	2.10E-28	0.0009	108.8376	mean cIMT
HDL Cholesterol	rs9491697	6	G	A	0.4636	-0.0275	0.0037	3.90E-14	0.0005	54.6829	mean cIMT
Total Cholesterol in Medium HDL	rs10162642	15	A	G	0.2101	-0.0266	0.0047	3.90E-10	0.0003	32.0849	mean cIMT
Total Cholesterol in Medium HDL	rs10184004	2	T	C	0.4061	0.0274	0.0039	3.90E-13	0.0004	49.9893	mean cIMT
Total Cholesterol in Medium HDL	rs1047891	2	A	C	0.3150	-0.0270	0.0041	1.00E-10	0.0004	43.6250	mean cIMT
Total Cholesterol in Medium HDL	rs1054852	12	G	A	0.3775	0.0323	0.0041	1.20E-16	0.0005	61.6797	mean cIMT
Total Cholesterol in Medium HDL	rs1077835	15	G	A	0.2197	0.0734	0.0046	6.70E-60	0.0022	255.0327	mean cIMT
Total Cholesterol in Medium HDL	rs11057692	12	G	A	0.2363	-0.0238	0.0045	1.70E-08	0.0002	27.6186	mean cIMT
Total Cholesterol in Medium HDL	rs112001035	17	A	G	0.0597	-0.0605	0.0082	3.50E-13	0.0005	54.7322	mean cIMT
Total Cholesterol in Medium HDL	rs116843064	19	A	G	0.0199	0.1954	0.0136	1.70E-48	0.0018	205.4330	mean cIMT
Total Cholesterol in Medium HDL	rs11751347	6	T	C	0.1023	-0.0415	0.0063	7.80E-12	0.0004	43.1897	mean cIMT
Total Cholesterol in Medium HDL	rs11789603	9	T	C	0.1088	0.0504	0.0061	1.20E-15	0.0006	67.8310	mean cIMT
Total Cholesterol in Medium HDL	rs12533197	7	G	T	0.4392	-0.0219	0.0039	2.50E-08	0.0003	32.3716	mean cIMT
Total Cholesterol in Medium HDL	rs1260326	2	C	T	0.6040	-0.0250	0.0039	4.60E-10	0.0004	41.5225	mean cIMT
Total Cholesterol in Medium HDL	rs12608026	18	G	T	0.0424	0.0834	0.0095	6.20E-19	0.0007	76.8455	mean cIMT
Total Cholesterol in Medium HDL	rs13107325	4	T	C	0.0743	-0.0709	0.0072	2.30E-23	0.0008	95.6765	mean cIMT
Total Cholesterol in Medium HDL	rs1358980	6	T	C	0.4826	-0.0271	0.0038	1.40E-12	0.0004	50.1814	mean cIMT
Total Cholesterol in Medium HDL	rs139915535	8	G	A	0.0180	-0.1464	0.0143	5.60E-26	0.0009	104.3630	mean cIMT
Total Cholesterol in Medium HDL	rs140584594	1	G	A	0.7301	0.0359	0.0043	1.10E-17	0.0006	71.0347	mean cIMT
Total Cholesterol in Medium HDL	rs141368429	11	T	C	0.0562	-0.0542	0.0089	1.50E-09	0.0003	37.4327	mean cIMT
Total Cholesterol in Medium HDL	rs144311893	19	T	C	0.0225	0.1121	0.0132	1.50E-18	0.0006	71.7112	mean cIMT
Total Cholesterol in Medium HDL	rs148063610	10	C	CAAATAAAT	0.7634	-0.0322	0.0046	9.50E-13	0.0004	49.7247	mean cIMT
Total Cholesterol in Medium HDL	rs15285	8	T	C	0.2865	0.1019	0.0042	7.40E-133	0.0051	587.9318	mean cIMT
Total Cholesterol in Medium HDL	rs1601935	15	T	G	0.6547	-0.0732	0.0040	1.00E-75	0.0029	330.0923	mean cIMT

Total Cholesterol in Medium HDL	rs1761457	19	G	A	0.3267	0.0271	0.0041	1.30E-12	0.0004	44.7196	mean cIMT
Total Cholesterol in Medium HDL	rs1800961	20	T	C	0.0302	-0.1229	0.0111	3.90E-28	0.0011	122.4479	mean cIMT
Total Cholesterol in Medium HDL	rs2229357	12	A	G	0.2409	0.0272	0.0044	2.80E-10	0.0003	37.7577	mean cIMT
Total Cholesterol in Medium HDL	rs2236252	20	T	C	0.1670	-0.0286	0.0051	4.30E-09	0.0003	31.5628	mean cIMT
Total Cholesterol in Medium HDL	rs2245221	8	A	G	0.5590	0.0325	0.0039	4.60E-16	0.0006	71.3199	mean cIMT
Total Cholesterol in Medium HDL	rs2298428	22	T	C	0.1826	-0.0398	0.0049	1.20E-16	0.0006	65.3920	mean cIMT
Total Cholesterol in Medium HDL	rs2395943	6	G	A	0.5884	0.0232	0.0039	2.90E-09	0.0003	36.2227	mean cIMT
Total Cholesterol in Medium HDL	rs2494748	14	T	C	0.6162	-0.0218	0.0039	4.60E-09	0.0003	30.9941	mean cIMT
Total Cholesterol in Medium HDL	rs2642438	1	G	A	0.7038	0.0356	0.0042	1.80E-18	0.0006	73.1810	mean cIMT
Total Cholesterol in Medium HDL	rs267738	1	G	T	0.2196	0.0382	0.0046	6.20E-18	0.0006	69.4697	mean cIMT
Total Cholesterol in Medium HDL	rs2740488	9	C	A	0.2653	-0.0388	0.0043	2.60E-20	0.0007	80.9756	mean cIMT
Total Cholesterol in Medium HDL	rs2792735	10	A	G	0.7203	-0.0388	0.0042	2.20E-21	0.0007	83.8362	mean cIMT
Total Cholesterol in Medium HDL	rs28746853	6	C	T	0.1765	0.0301	0.0055	4.80E-08	0.0003	30.3223	mean cIMT
Total Cholesterol in Medium HDL	rs28818616	3	C	T	0.3498	-0.0215	0.0040	2.30E-08	0.0003	28.8688	mean cIMT
Total Cholesterol in Medium HDL	rs2925979	16	C	T	0.6989	0.0342	0.0041	3.20E-18	0.0006	68.7144	mean cIMT
Total Cholesterol in Medium HDL	rs2943650	2	T	C	0.6448	-0.0336	0.0040	5.40E-19	0.0006	71.7604	mean cIMT
Total Cholesterol in Medium HDL	rs2978615	19	T	C	0.4902	-0.0251	0.0038	2.80E-11	0.0004	43.3960	mean cIMT
Total Cholesterol in Medium HDL	rs35135293	2	T	C	0.5167	-0.0241	0.0038	9.90E-11	0.0003	39.7762	mean cIMT
Total Cholesterol in Medium HDL	rs35184771	11	T	G	0.3528	-0.0365	0.0040	7.10E-20	0.0007	84.5538	mean cIMT
Total Cholesterol in Medium HDL	rs3764261	16	A	C	0.3244	0.1845	0.0040	1.00E-200	0.0177	2075.4093	mean cIMT
Total Cholesterol in Medium HDL	rs3768321	1	T	G	0.1965	-0.0428	0.0048	2.50E-20	0.0007	79.9757	mean cIMT
Total Cholesterol in Medium HDL	rs3795269	1	A	C	0.4386	0.0204	0.0038	4.80E-08	0.0002	28.6267	mean cIMT
Total Cholesterol in Medium HDL	rs4239651	20	C	T	0.7942	0.0356	0.0047	1.90E-15	0.0005	57.4094	mean cIMT
Total Cholesterol in Medium HDL	rs4240624	8	A	G	0.9092	0.1027	0.0066	4.20E-55	0.0021	240.7162	mean cIMT
Total Cholesterol in Medium HDL	rs429358	19	C	T	0.1548	-0.0749	0.0053	8.60E-49	0.0018	202.7585	mean cIMT
Total Cholesterol in Medium HDL	rs4330777	16	A	G	0.4759	-0.0198	0.0038	3.20E-08	0.0002	26.9202	mean cIMT
Total Cholesterol in Medium HDL	rs4656292	1	G	A	0.6196	0.0198	0.0039	2.90E-08	0.0002	25.4952	mean cIMT
Total Cholesterol in Medium HDL	rs4846921	1	A	G	0.6132	0.0521	0.0039	1.60E-42	0.0016	178.8545	mean cIMT

Total Cholesterol in Medium HDL	rs4969141	17	T	C	0.4895	0.0240	0.0038	1.50E-10	0.0003	39.7162	mean cIMT
Total Cholesterol in Medium HDL	rs583104	1	T	G	0.7734	-0.0395	0.0045	3.40E-19	0.0007	75.9449	mean cIMT
Total Cholesterol in Medium HDL	rs59097294	11	C	T	0.1815	0.0653	0.0049	1.00E-42	0.0015	176.0489	mean cIMT
Total Cholesterol in Medium HDL	rs60847460	10	T	C	0.1433	-0.0382	0.0054	5.20E-12	0.0004	49.2095	mean cIMT
Total Cholesterol in Medium HDL	rs61805076	1	C	T	0.3341	-0.0278	0.0040	2.50E-12	0.0004	47.5764	mean cIMT
Total Cholesterol in Medium HDL	rs62101705	18	T	C	0.0136	-0.0906	0.0167	2.10E-08	0.0003	29.4699	mean cIMT
Total Cholesterol in Medium HDL	rs638714	1	T	G	0.3459	-0.0281	0.0040	6.40E-13	0.0004	49.3227	mean cIMT
Total Cholesterol in Medium HDL	rs6589565	11	G	A	0.9322	0.0724	0.0076	1.30E-23	0.0008	91.6128	mean cIMT
Total Cholesterol in Medium HDL	rs6606717	12	C	A	0.5232	0.0270	0.0038	4.60E-13	0.0004	50.6259	mean cIMT
Total Cholesterol in Medium HDL	rs676210	2	A	G	0.2058	0.0750	0.0047	8.80E-57	0.0022	256.4445	mean cIMT
Total Cholesterol in Medium HDL	rs686030	9	A	C	0.8595	0.0391	0.0055	4.10E-13	0.0004	50.9982	mean cIMT
Total Cholesterol in Medium HDL	rs6967917	7	G	A	0.4916	0.0219	0.0038	2.60E-09	0.0003	32.9393	mean cIMT
Total Cholesterol in Medium HDL	rs7134375	12	A	C	0.4302	0.0210	0.0038	1.30E-08	0.0003	29.9373	mean cIMT
Total Cholesterol in Medium HDL	rs7136506	12	C	T	0.2158	-0.0377	0.0047	1.80E-16	0.0006	64.3084	mean cIMT
Total Cholesterol in Medium HDL	rs7241918	18	T	G	0.8229	0.0872	0.0050	6.60E-70	0.0026	305.5062	mean cIMT
Total Cholesterol in Medium HDL	rs72836561	17	T	C	0.0315	-0.1484	0.0109	8.70E-44	0.0016	185.8791	mean cIMT
Total Cholesterol in Medium HDL	rs737337	19	C	T	0.0768	-0.0677	0.0071	1.80E-21	0.0008	89.9663	mean cIMT
Total Cholesterol in Medium HDL	rs75911530	16	A	G	0.0329	-0.1528	0.0112	5.50E-44	0.0016	186.7382	mean cIMT
Total Cholesterol in Medium HDL	rs77960347	18	G	A	0.0132	0.2880	0.0166	6.50E-70	0.0026	301.1697	mean cIMT
Total Cholesterol in Medium HDL	rs7810507	7	A	G	0.2811	-0.0272	0.0042	1.30E-10	0.0004	41.5996	mean cIMT
Total Cholesterol in Medium HDL	rs78296522	11	A	C	0.0445	0.0647	0.0093	2.40E-12	0.0004	48.7623	mean cIMT
Total Cholesterol in Medium HDL	rs7956099	12	C	T	0.4750	-0.0209	0.0038	1.80E-08	0.0003	29.9218	mean cIMT
Total Cholesterol in Medium HDL	rs8058512	16	T	C	0.7240	-0.0250	0.0043	5.60E-09	0.0003	34.5696	mean cIMT
Total Cholesterol in Medium HDL	rs904770	16	C	T	0.8894	-0.0348	0.0060	1.10E-08	0.0003	33.0499	mean cIMT
Total Cholesterol in Medium HDL	rs921919	12	A	G	0.6695	-0.0382	0.0041	2.20E-22	0.0007	85.5947	mean cIMT
Total Cholesterol in Medium HDL	rs9491697	6	G	A	0.4636	-0.0255	0.0038	2.90E-11	0.0004	44.3755	mean cIMT
Total Cholesterol in Medium HDL	rs967645	17	T	C	0.5165	-0.0264	0.0038	2.40E-11	0.0004	48.2309	mean cIMT
Total Cholesterol in Medium HDL	rs9687846	5	A	G	0.2014	-0.0311	0.0047	3.50E-11	0.0004	43.1706	mean cIMT

Free Cholesterol in Medium HDL	rs10184004	2	T	C	0.4061	0.0232	0.0038	2.70E-10	0.0003	36.6234	mean cIMT
Free Cholesterol in Medium HDL	rs11057692	12	G	A	0.2363	-0.0227	0.0045	4.90E-08	0.0002	25.8717	mean cIMT
Free Cholesterol in Medium HDL	rs112001035	17	A	G	0.0597	-0.0526	0.0081	2.00E-10	0.0004	42.3974	mean cIMT
Free Cholesterol in Medium HDL	rs112495680	6	G	A	0.1334	-0.0357	0.0056	8.50E-11	0.0004	41.3581	mean cIMT
Free Cholesterol in Medium HDL	rs116843064	19	A	G	0.0199	0.1671	0.0135	7.00E-37	0.0013	153.4511	mean cIMT
Free Cholesterol in Medium HDL	rs11751347	6	T	C	0.1023	-0.0362	0.0062	2.40E-09	0.0003	33.6904	mean cIMT
Free Cholesterol in Medium HDL	rs11789603	9	T	C	0.1088	0.0551	0.0061	5.00E-19	0.0007	83.0360	mean cIMT
Free Cholesterol in Medium HDL	rs1260326	2	C	T	0.6040	-0.0323	0.0038	2.00E-16	0.0006	70.5787	mean cIMT
Free Cholesterol in Medium HDL	rs12608026	18	G	T	0.0424	0.0884	0.0094	1.50E-21	0.0008	88.2367	mean cIMT
Free Cholesterol in Medium HDL	rs12721046	19	A	G	0.1577	-0.0489	0.0052	1.10E-21	0.0008	89.6323	mean cIMT
Free Cholesterol in Medium HDL	rs13107325	4	T	C	0.0743	-0.0702	0.0072	5.70E-24	0.0008	95.8795	mean cIMT
Free Cholesterol in Medium HDL	rs139915535	8	G	A	0.0180	-0.1270	0.0142	9.70E-21	0.0007	80.1950	mean cIMT
Free Cholesterol in Medium HDL	rs140584594	1	G	A	0.7301	0.0325	0.0042	5.00E-15	0.0005	59.6865	mean cIMT
Free Cholesterol in Medium HDL	rs148063610	10	C	CAAATAAAT	0.7634	-0.0339	0.0045	2.70E-14	0.0005	56.4722	mean cIMT
Free Cholesterol in Medium HDL	rs15285	8	T	C	0.2865	0.0876	0.0042	1.80E-100	0.0038	444.3010	mean cIMT
Free Cholesterol in Medium HDL	rs174574	11	C	A	0.6494	0.0521	0.0039	5.50E-43	0.0015	175.8135	mean cIMT
Free Cholesterol in Medium HDL	rs17696736	12	G	A	0.4302	-0.0189	0.0038	2.70E-08	0.0002	24.7253	mean cIMT
Free Cholesterol in Medium HDL	rs1800961	20	T	C	0.0302	-0.1190	0.0110	4.20E-27	0.0010	117.4397	mean cIMT
Free Cholesterol in Medium HDL	rs193084249	1	G	A	0.0234	-0.0973	0.0127	2.00E-14	0.0005	58.7063	mean cIMT
Free Cholesterol in Medium HDL	rs2043085	15	C	T	0.6127	-0.1120	0.0039	7.29E-191	0.0073	843.9531	mean cIMT
Free Cholesterol in Medium HDL	rs2066714	9	C	T	0.1289	0.0317	0.0056	3.40E-09	0.0003	32.1954	mean cIMT
Free Cholesterol in Medium HDL	rs2070895	15	A	G	0.2187	0.1219	0.0045	1.90E-165	0.0062	719.8112	mean cIMT
Free Cholesterol in Medium HDL	rs2176040	2	G	A	0.6467	-0.0303	0.0039	2.00E-16	0.0005	59.5001	mean cIMT
Free Cholesterol in Medium HDL	rs2229357	12	A	G	0.2409	0.0232	0.0044	4.00E-08	0.0002	28.1418	mean cIMT
Free Cholesterol in Medium HDL	rs2236252	20	T	C	0.1670	-0.0288	0.0050	3.70E-09	0.0003	32.6759	mean cIMT
Free Cholesterol in Medium HDL	rs2245221	8	A	G	0.5590	0.0334	0.0038	1.30E-17	0.0007	76.6039	mean cIMT
Free Cholesterol in Medium HDL	rs2298428	22	T	C	0.1826	-0.0402	0.0049	2.70E-17	0.0006	68.1252	mean cIMT
Free Cholesterol in Medium HDL	rs2494748	14	T	C	0.6162	-0.0198	0.0039	4.00E-08	0.0002	26.0146	mean cIMT

Free Cholesterol in Medium HDL	rs2642438	1	G	A	0.7038	0.0371	0.0041	1.60E-20	0.0007	81.1535	mean cIMT
Free Cholesterol in Medium HDL	rs267738	1	G	T	0.2196	0.0337	0.0045	8.40E-15	0.0005	55.1931	mean cIMT
Free Cholesterol in Medium HDL	rs2740488	9	C	A	0.2653	-0.0433	0.0043	4.20E-25	0.0009	102.8040	mean cIMT
Free Cholesterol in Medium HDL	rs2781752	19	A	G	0.3182	0.0236	0.0041	4.60E-10	0.0003	33.5305	mean cIMT
Free Cholesterol in Medium HDL	rs2792735	10	A	G	0.7203	-0.0414	0.0042	2.90E-24	0.0008	97.8053	mean cIMT
Free Cholesterol in Medium HDL	rs28818616	3	C	T	0.3498	-0.0211	0.0040	4.30E-08	0.0002	28.3329	mean cIMT
Free Cholesterol in Medium HDL	rs2925979	16	C	T	0.6989	0.0308	0.0041	1.90E-15	0.0005	56.6888	mean cIMT
Free Cholesterol in Medium HDL	rs2978615	19	T	C	0.4902	-0.0233	0.0038	4.20E-10	0.0003	38.2643	mean cIMT
Free Cholesterol in Medium HDL	rs34663616	15	A	C	0.1377	0.0367	0.0056	1.70E-11	0.0004	43.3559	mean cIMT
Free Cholesterol in Medium HDL	rs34707604	4	C	T	0.2585	0.0253	0.0045	3.10E-09	0.0003	31.0279	mean cIMT
Free Cholesterol in Medium HDL	rs34955778	16	C	T	0.4200	-0.0208	0.0038	3.80E-08	0.0003	29.8199	mean cIMT
Free Cholesterol in Medium HDL	rs35135293	2	T	C	0.5167	-0.0275	0.0038	9.60E-14	0.0005	53.1208	mean cIMT
Free Cholesterol in Medium HDL	rs35184771	11	T	G	0.3528	-0.0366	0.0039	1.70E-20	0.0008	86.9695	mean cIMT
Free Cholesterol in Medium HDL	rs3735687	7	G	A	0.4228	-0.0243	0.0038	9.60E-10	0.0004	40.3464	mean cIMT
Free Cholesterol in Medium HDL	rs3764261	16	A	C	0.3244	0.1822	0.0040	1.00E-200	0.0177	2070.8081	mean cIMT
Free Cholesterol in Medium HDL	rs3768321	1	T	G	0.1965	-0.0389	0.0047	1.10E-17	0.0006	67.5296	mean cIMT
Free Cholesterol in Medium HDL	rs41272663	2	A	C	0.2640	0.0247	0.0043	6.60E-09	0.0003	33.7077	mean cIMT
Free Cholesterol in Medium HDL	rs4239651	20	C	T	0.7942	0.0364	0.0047	4.20E-16	0.0005	61.3438	mean cIMT
Free Cholesterol in Medium HDL	rs4240624	8	A	G	0.9092	0.0988	0.0065	3.00E-52	0.0020	228.0097	mean cIMT
Free Cholesterol in Medium HDL	rs4846921	1	A	G	0.6132	0.0494	0.0039	5.80E-39	0.0014	164.3395	mean cIMT
Free Cholesterol in Medium HDL	rs4969141	17	T	C	0.4895	0.0211	0.0038	1.60E-08	0.0003	31.2747	mean cIMT
Free Cholesterol in Medium HDL	rs5167	19	G	T	0.3523	0.0480	0.0039	8.10E-35	0.0013	149.2442	mean cIMT
Free Cholesterol in Medium HDL	rs583104	1	T	G	0.7734	-0.0296	0.0045	1.10E-11	0.0004	43.7231	mean cIMT
Free Cholesterol in Medium HDL	rs60847460	10	T	C	0.1433	-0.0380	0.0054	3.10E-12	0.0004	49.8107	mean cIMT
Free Cholesterol in Medium HDL	rs61805076	1	C	T	0.3341	-0.0277	0.0040	1.20E-12	0.0004	48.2745	mean cIMT
Free Cholesterol in Medium HDL	rs61941676	12	A	C	0.1272	-0.0565	0.0058	5.20E-23	0.0008	95.6033	mean cIMT
Free Cholesterol in Medium HDL	rs62101704	18	A	G	0.0137	-0.0900	0.0165	1.20E-08	0.0003	29.8754	mean cIMT
Free Cholesterol in Medium HDL	rs638714	1	T	G	0.3459	-0.0395	0.0040	5.30E-24	0.0009	99.0832	mean cIMT

Free Cholesterol in Medium HDL	rs6509173	19	G	A	0.7937	-0.0264	0.0047	3.20E-08	0.0003	31.9214	mean cIMT
Free Cholesterol in Medium HDL	rs6606717	12	C	A	0.5232	0.0302	0.0038	3.20E-16	0.0006	64.4676	mean cIMT
Free Cholesterol in Medium HDL	rs676210	2	A	G	0.2058	0.0646	0.0046	2.50E-43	0.0017	194.5249	mean cIMT
Free Cholesterol in Medium HDL	rs686030	9	A	C	0.8595	0.0439	0.0054	1.80E-16	0.0006	65.7455	mean cIMT
Free Cholesterol in Medium HDL	rs7241918	18	T	G	0.8229	0.0908	0.0049	8.90E-78	0.0029	338.2758	mean cIMT
Free Cholesterol in Medium HDL	rs72836561	17	T	C	0.0315	-0.1357	0.0108	5.60E-38	0.0014	158.9264	mean cIMT
Free Cholesterol in Medium HDL	rs737337	19	C	T	0.0768	-0.0690	0.0071	1.10E-22	0.0008	95.4810	mean cIMT
Free Cholesterol in Medium HDL	rs75911530	16	A	G	0.0329	-0.1448	0.0111	1.70E-41	0.0015	171.6077	mean cIMT
Free Cholesterol in Medium HDL	rs77960347	18	G	A	0.0132	0.3054	0.0164	1.80E-79	0.0030	346.2468	mean cIMT
Free Cholesterol in Medium HDL	rs7810507	7	A	G	0.2811	-0.0233	0.0042	1.50E-08	0.0003	31.1290	mean cIMT
Free Cholesterol in Medium HDL	rs7956099	12	C	T	0.4750	-0.0223	0.0038	1.50E-09	0.0003	34.7202	mean cIMT
Free Cholesterol in Medium HDL	rs7959043	12	G	A	0.4084	0.0202	0.0038	4.70E-08	0.0002	27.8404	mean cIMT
Free Cholesterol in Medium HDL	rs8058512	16	T	C	0.7240	-0.0250	0.0042	3.70E-09	0.0003	35.1636	mean cIMT
Free Cholesterol in Medium HDL	rs838876	12	G	A	0.6814	-0.0385	0.0041	1.50E-23	0.0008	88.5803	mean cIMT
Free Cholesterol in Medium HDL	rs904770	16	C	T	0.8894	-0.0325	0.0060	4.10E-08	0.0003	29.5149	mean cIMT
Free Cholesterol in Medium HDL	rs9471972	6	A	G	0.5362	0.0228	0.0038	2.70E-09	0.0003	36.7108	mean cIMT
Free Cholesterol in Medium HDL	rs9491697	6	G	A	0.4636	-0.0225	0.0038	2.40E-09	0.0003	35.3113	mean cIMT
Free Cholesterol in Medium HDL	rs967645	17	T	C	0.5165	-0.0268	0.0038	6.30E-12	0.0004	51.0197	mean cIMT
Free Cholesterol in Medium HDL	rs9687846	5	A	G	0.2014	-0.0261	0.0047	1.20E-08	0.0003	31.0168	mean cIMT
Cholesteryl Esters in HDL	rs10015477	4	A	G	0.3683	-0.0211	0.0039	7.30E-09	0.0003	29.9565	mean cIMT
Cholesteryl Esters in HDL	rs10162642	15	A	G	0.2101	-0.0328	0.0046	3.20E-15	0.0004	51.3109	mean cIMT
Cholesteryl Esters in HDL	rs10268632	7	C	A	0.5118	0.0226	0.0037	5.60E-10	0.0003	37.0088	mean cIMT
Cholesteryl Esters in HDL	rs1047891	2	A	C	0.3150	-0.0243	0.0040	3.30E-09	0.0003	36.9786	mean cIMT
Cholesteryl Esters in HDL	rs1054852	12	G	A	0.3775	0.0338	0.0040	1.40E-18	0.0006	70.7347	mean cIMT
Cholesteryl Esters in HDL	rs11057692	12	G	A	0.2363	-0.0260	0.0044	4.30E-10	0.0003	34.6895	mean cIMT
Cholesteryl Esters in HDL	rs112001035	17	A	G	0.0597	-0.0600	0.0080	4.60E-14	0.0005	56.5650	mean cIMT
Cholesteryl Esters in HDL	rs11429307	5	G	GT	0.8085	0.0423	0.0047	4.30E-19	0.0007	80.0801	mean cIMT
Cholesteryl Esters in HDL	rs11434143	8	G	GT	0.8108	-0.0254	0.0048	1.80E-08	0.0002	27.9337	mean cIMT

Cholesteryl Esters in HDL	rs116843064	19	A	G	0.0199	0.2229	0.0133	4.10E-66	0.0024	280.6896	mean cIMT
Cholesteryl Esters in HDL	rs116978226	11	A	C	0.0332	0.0646	0.0104	1.40E-10	0.0003	38.3536	mean cIMT
Cholesteryl Esters in HDL	rs11789603	9	T	C	0.1088	0.0511	0.0060	1.60E-16	0.0006	73.1943	mean cIMT
Cholesteryl Esters in HDL	rs1215112	9	A	G	0.8630	0.0466	0.0054	1.50E-18	0.0006	74.6293	mean cIMT
Cholesteryl Esters in HDL	rs12295878	11	T	C	0.1403	0.0331	0.0053	2.10E-09	0.0003	38.4781	mean cIMT
Cholesteryl Esters in HDL	rs12453682	17	T	C	0.6936	0.0212	0.0040	2.20E-08	0.0002	27.4347	mean cIMT
Cholesteryl Esters in HDL	rs12533197	7	G	T	0.4392	-0.0216	0.0038	4.30E-08	0.0003	33.0838	mean cIMT
Cholesteryl Esters in HDL	rs12608026	18	G	T	0.0424	0.0705	0.0093	1.80E-14	0.0005	57.5896	mean cIMT
Cholesteryl Esters in HDL	rs12721046	19	A	G	0.1577	-0.0366	0.0051	1.00E-13	0.0004	51.4740	mean cIMT
Cholesteryl Esters in HDL	rs13107325	4	T	C	0.0743	-0.0746	0.0071	2.60E-27	0.0010	111.4825	mean cIMT
Cholesteryl Esters in HDL	rs13389219	2	T	C	0.3925	0.0358	0.0038	4.20E-22	0.0008	88.7306	mean cIMT
Cholesteryl Esters in HDL	rs1358980	6	T	C	0.4826	-0.0335	0.0037	2.70E-19	0.0007	80.6400	mean cIMT
Cholesteryl Esters in HDL	rs140584594	1	G	A	0.7301	0.0254	0.0042	7.60E-10	0.0003	37.5641	mean cIMT
Cholesteryl Esters in HDL	rs142110991	6	C	T	0.0773	-0.0468	0.0088	2.30E-08	0.0002	28.4125	mean cIMT
Cholesteryl Esters in HDL	rs142265900	12	A	AGAT	0.9424	-0.0537	0.0080	5.40E-11	0.0004	44.8625	mean cIMT
Cholesteryl Esters in HDL	rs145391587	8	C	A	0.1003	0.1645	0.0062	3.00E-163	0.0062	715.1922	mean cIMT
Cholesteryl Esters in HDL	rs147233090	15	T	C	0.0246	-0.0851	0.0121	5.00E-13	0.0004	49.8110	mean cIMT
Cholesteryl Esters in HDL	rs148063610	10	C	CAAATAAAT	0.7634	-0.0328	0.0045	6.90E-14	0.0005	54.2659	mean cIMT
Cholesteryl Esters in HDL	rs150224153	20	T	C	0.0292	-0.0924	0.0113	4.00E-16	0.0006	66.9244	mean cIMT
Cholesteryl Esters in HDL	rs1601935	15	T	G	0.6547	-0.0830	0.0039	4.50E-102	0.0039	446.7240	mean cIMT
Cholesteryl Esters in HDL	rs1761457	19	G	A	0.3267	0.0296	0.0040	3.30E-15	0.0005	56.0228	mean cIMT
Cholesteryl Esters in HDL	rs17696736	12	G	A	0.4302	-0.0216	0.0037	1.20E-10	0.0003	33.3700	mean cIMT
Cholesteryl Esters in HDL	rs2066714	9	C	T	0.1289	0.0325	0.0055	4.50E-10	0.0003	34.7041	mean cIMT
Cholesteryl Esters in HDL	rs2070895	15	A	G	0.2187	0.0898	0.0045	2.90E-94	0.0035	400.9423	mean cIMT
Cholesteryl Esters in HDL	rs2229357	12	A	G	0.2409	0.0306	0.0043	2.50E-13	0.0004	50.2308	mean cIMT
Cholesteryl Esters in HDL	rs2245221	8	A	G	0.5590	0.0304	0.0038	7.00E-15	0.0006	65.4012	mean cIMT
Cholesteryl Esters in HDL	rs2297402	9	T	C	0.0238	-0.0641	0.0127	3.50E-08	0.0002	25.6418	mean cIMT
Cholesteryl Esters in HDL	rs2298428	22	T	C	0.1826	-0.0357	0.0048	1.00E-14	0.0005	55.3156	mean cIMT

Cholesteryl Esters in HDL	rs2302263	11	T	C	0.0881	-0.0371	0.0066	1.30E-08	0.0003	32.0724	mean cIMT
Cholesteryl Esters in HDL	rs2307111	5	C	T	0.3966	0.0237	0.0038	6.30E-10	0.0003	39.0690	mean cIMT
Cholesteryl Esters in HDL	rs2494748	14	T	C	0.6162	-0.0209	0.0038	6.20E-09	0.0003	29.9823	mean cIMT
Cholesteryl Esters in HDL	rs2642438	1	G	A	0.7038	0.0352	0.0041	6.80E-19	0.0007	75.1042	mean cIMT
Cholesteryl Esters in HDL	rs267738	1	G	T	0.2196	0.0284	0.0045	3.30E-11	0.0004	40.4153	mean cIMT
Cholesteryl Esters in HDL	rs2740488	9	C	A	0.2653	-0.0428	0.0042	1.50E-25	0.0009	103.3090	mean cIMT
Cholesteryl Esters in HDL	rs2792735	10	A	G	0.7203	-0.0394	0.0041	3.70E-23	0.0008	90.9264	mean cIMT
Cholesteryl Esters in HDL	rs28818616	3	C	T	0.3498	-0.0203	0.0039	3.50E-08	0.0002	26.9151	mean cIMT
Cholesteryl Esters in HDL	rs2925979	16	C	T	0.6989	0.0394	0.0040	2.00E-24	0.0008	96.1390	mean cIMT
Cholesteryl Esters in HDL	rs2943650	2	T	C	0.6448	-0.0418	0.0039	1.50E-29	0.0010	116.5459	mean cIMT
Cholesteryl Esters in HDL	rs2978615	19	T	C	0.4902	-0.0203	0.0037	3.00E-08	0.0003	29.6727	mean cIMT
Cholesteryl Esters in HDL	rs34265539	6	A	ATT	0.3561	-0.0267	0.0039	2.70E-13	0.0004	46.6503	mean cIMT
Cholesteryl Esters in HDL	rs35184771	11	T	G	0.3528	-0.0386	0.0039	2.40E-23	0.0009	99.3863	mean cIMT
Cholesteryl Esters in HDL	rs35633876	2	T	G	0.4818	-0.0225	0.0037	6.30E-10	0.0003	36.5109	mean cIMT
Cholesteryl Esters in HDL	rs3735687	7	G	A	0.4228	-0.0226	0.0038	6.80E-09	0.0003	35.9289	mean cIMT
Cholesteryl Esters in HDL	rs3764261	16	A	C	0.3244	0.2097	0.0039	1.00E-200	0.0240	2825.3783	mean cIMT
Cholesteryl Esters in HDL	rs3768321	1	T	G	0.1965	-0.0442	0.0047	5.30E-23	0.0008	89.3450	mean cIMT
Cholesteryl Esters in HDL	rs4078216	12	A	G	0.2409	0.0261	0.0043	2.10E-08	0.0003	36.1459	mean cIMT
Cholesteryl Esters in HDL	rs4240624	8	A	G	0.9092	0.0944	0.0065	1.50E-49	0.0019	214.2529	mean cIMT
Cholesteryl Esters in HDL	rs4330777	16	A	G	0.4759	-0.0202	0.0037	1.10E-08	0.0003	29.5354	mean cIMT
Cholesteryl Esters in HDL	rs4846921	1	A	G	0.6132	0.0495	0.0038	4.60E-41	0.0015	169.7396	mean cIMT
Cholesteryl Esters in HDL	rs4922787	11	T	G	0.7616	0.0271	0.0044	2.30E-10	0.0003	38.3807	mean cIMT
Cholesteryl Esters in HDL	rs4969141	17	T	C	0.4895	0.0238	0.0037	1.40E-10	0.0004	41.0039	mean cIMT
Cholesteryl Esters in HDL	rs5167	19	G	T	0.3523	0.0443	0.0039	4.40E-31	0.0011	130.2284	mean cIMT
Cholesteryl Esters in HDL	rs59104589	2	T	C	0.3588	0.0205	0.0039	1.00E-08	0.0002	28.0376	mean cIMT
Cholesteryl Esters in HDL	rs59299606	16	A	G	0.2491	-0.0279	0.0047	1.30E-09	0.0003	34.4286	mean cIMT
Cholesteryl Esters in HDL	rs6018652	20	A	G	0.7925	0.0352	0.0046	9.50E-16	0.0005	58.6834	mean cIMT
Cholesteryl Esters in HDL	rs6073958	20	C	T	0.1987	-0.0674	0.0047	6.90E-47	0.0018	209.5670	mean cIMT

Cholesteryl Esters in HDL	rs60847460	10	T	C	0.1433	-0.0364	0.0053	7.40E-12	0.0004	47.0029	mean cIMT
Cholesteryl Esters in HDL	rs61805076	1	C	T	0.3341	-0.0297	0.0039	1.90E-14	0.0005	57.3464	mean cIMT
Cholesteryl Esters in HDL	rs62101705	18	T	C	0.0136	-0.0891	0.0163	8.40E-09	0.0003	29.9250	mean cIMT
Cholesteryl Esters in HDL	rs676210	2	A	G	0.2058	0.0720	0.0046	1.70E-55	0.0022	247.9752	mean cIMT
Cholesteryl Esters in HDL	rs688671	18	G	A	0.2671	-0.0248	0.0042	4.30E-09	0.0003	34.6480	mean cIMT
Cholesteryl Esters in HDL	rs71205961	8	C	CTTTTTT	0.0890	-0.0441	0.0073	2.90E-09	0.0003	36.5575	mean cIMT
Cholesteryl Esters in HDL	rs71336055	3	T	C	0.1197	0.0357	0.0057	3.90E-10	0.0003	38.8542	mean cIMT
Cholesteryl Esters in HDL	rs7134375	12	A	C	0.4302	0.0240	0.0037	5.60E-11	0.0004	41.1493	mean cIMT
Cholesteryl Esters in HDL	rs7136506	12	C	T	0.2158	-0.0398	0.0046	9.00E-19	0.0007	75.4329	mean cIMT
Cholesteryl Esters in HDL	rs71562509	6	T	G	0.5933	0.0208	0.0038	3.30E-08	0.0003	30.0825	mean cIMT
Cholesteryl Esters in HDL	rs7241918	18	T	G	0.8229	0.0745	0.0049	1.70E-54	0.0020	233.7084	mean cIMT
Cholesteryl Esters in HDL	rs72836561	17	T	C	0.0315	-0.1592	0.0106	9.10E-54	0.0020	224.8647	mean cIMT
Cholesteryl Esters in HDL	rs7308864	12	G	A	0.5233	0.0250	0.0037	3.80E-12	0.0004	45.6090	mean cIMT
Cholesteryl Esters in HDL	rs737337	19	C	T	0.0768	-0.0589	0.0070	1.20E-17	0.0006	71.5567	mean cIMT
Cholesteryl Esters in HDL	rs75911530	16	A	G	0.0329	-0.1619	0.0109	8.10E-52	0.0019	221.0727	mean cIMT
Cholesteryl Esters in HDL	rs77960347	18	G	A	0.0132	0.2479	0.0162	4.30E-55	0.0020	234.1215	mean cIMT
Cholesteryl Esters in HDL	rs78058190	2	A	G	0.0501	-0.0647	0.0096	9.00E-11	0.0004	45.4375	mean cIMT
Cholesteryl Esters in HDL	rs7810507	7	A	G	0.2811	-0.0315	0.0041	1.50E-14	0.0005	58.5049	mean cIMT
Cholesteryl Esters in HDL	rs7845090	8	A	G	0.7094	0.0236	0.0041	1.40E-08	0.0003	33.2418	mean cIMT
Cholesteryl Esters in HDL	rs8058512	16	T	C	0.7240	-0.0268	0.0041	1.90E-10	0.0004	41.7182	mean cIMT
Cholesteryl Esters in HDL	rs904770	16	C	T	0.8894	-0.0372	0.0059	2.50E-10	0.0003	39.8068	mean cIMT
Cholesteryl Esters in HDL	rs921919	12	A	G	0.6695	-0.0415	0.0040	8.90E-28	0.0009	106.2983	mean cIMT
Cholesteryl Esters in HDL	rs9265113	6	T	C	0.4076	-0.0290	0.0040	2.90E-13	0.0004	51.4973	mean cIMT
Cholesteryl Esters in HDL	rs9491697	6	G	A	0.4636	-0.0284	0.0037	7.10E-15	0.0005	57.9885	mean cIMT
Cholesteryl Esters in Medium HDL	rs10184004	2	T	C	0.4061	0.0283	0.0039	8.80E-14	0.0005	53.1080	mean cIMT
Cholesteryl Esters in Medium HDL	rs1047891	2	A	C	0.3150	-0.0278	0.0041	3.30E-11	0.0004	45.7324	mean cIMT
Cholesteryl Esters in Medium HDL	rs1077835	15	G	A	0.2197	0.0606	0.0046	3.70E-41	0.0015	172.7056	mean cIMT
Cholesteryl Esters in Medium HDL	rs11057692	12	G	A	0.2363	-0.0239	0.0045	1.60E-08	0.0002	27.7482	mean cIMT

Cholesteryl Esters in Medium HDL	rs112001035	17	A	G	0.0597	-0.0621	0.0082	7.90E-14	0.0005	57.2673	mean cIMT
Cholesteryl Esters in Medium HDL	rs11591147	1	T	G	0.0175	0.0926	0.0145	8.00E-11	0.0004	40.5924	mean cIMT
Cholesteryl Esters in Medium HDL	rs116843064	19	A	G	0.0199	0.2013	0.0137	6.10E-51	0.0019	216.5733	mean cIMT
Cholesteryl Esters in Medium HDL	rs11751347	6	T	C	0.1023	-0.0426	0.0063	2.40E-12	0.0004	45.1508	mean cIMT
Cholesteryl Esters in Medium HDL	rs117687565	18	T	C	0.0120	0.1166	0.0181	7.30E-10	0.0004	41.3068	mean cIMT
Cholesteryl Esters in Medium HDL	rs11789603	9	T	C	0.1088	0.0489	0.0061	1.10E-14	0.0005	63.3043	mean cIMT
Cholesteryl Esters in Medium HDL	rs12533197	7	G	T	0.4392	-0.0220	0.0039	2.50E-08	0.0003	32.3373	mean cIMT
Cholesteryl Esters in Medium HDL	rs1260326	2	C	T	0.6040	-0.0230	0.0039	1.30E-08	0.0003	34.6906	mean cIMT
Cholesteryl Esters in Medium HDL	rs12611067	19	T	G	0.3509	0.0246	0.0040	1.60E-09	0.0003	37.5816	mean cIMT
Cholesteryl Esters in Medium HDL	rs13107325	4	T	C	0.0743	-0.0706	0.0073	6.70E-23	0.0008	94.3570	mean cIMT
Cholesteryl Esters in Medium HDL	rs1358980	6	T	C	0.4826	-0.0278	0.0038	3.20E-13	0.0005	52.4635	mean cIMT
Cholesteryl Esters in Medium HDL	rs139915535	8	G	A	0.0180	-0.1503	0.0144	5.80E-27	0.0009	109.2443	mean cIMT
Cholesteryl Esters in Medium HDL	rs140584594	1	G	A	0.7301	0.0365	0.0043	4.20E-18	0.0006	72.8343	mean cIMT
Cholesteryl Esters in Medium HDL	rs141368429	11	T	C	0.0562	-0.0549	0.0089	1.00E-09	0.0003	37.9956	mean cIMT
Cholesteryl Esters in Medium HDL	rs144311893	19	T	C	0.0225	0.1208	0.0133	4.90E-21	0.0007	82.5990	mean cIMT
Cholesteryl Esters in Medium HDL	rs148063610	10	C	CAAATAAAT	0.7634	-0.0315	0.0046	3.30E-12	0.0004	47.3690	mean cIMT
Cholesteryl Esters in Medium HDL	rs15285	8	T	C	0.2865	0.1049	0.0042	5.40E-140	0.0053	618.0180	mean cIMT
Cholesteryl Esters in Medium HDL	rs1601935	15	T	G	0.6547	-0.0626	0.0040	1.70E-55	0.0021	239.7330	mean cIMT
Cholesteryl Esters in Medium HDL	rs1761457	19	G	A	0.3267	0.0280	0.0041	3.60E-13	0.0004	47.3273	mean cIMT
Cholesteryl Esters in Medium HDL	rs1800961	20	T	C	0.0302	-0.1231	0.0111	4.80E-28	0.0011	121.9323	mean cIMT
Cholesteryl Esters in Medium HDL	rs2228671	19	T	C	0.1264	0.0398	0.0058	2.70E-13	0.0004	47.7660	mean cIMT
Cholesteryl Esters in Medium HDL	rs2229357	12	A	G	0.2409	0.0281	0.0044	1.10E-10	0.0003	39.9233	mean cIMT
Cholesteryl Esters in Medium HDL	rs2236252	20	T	C	0.1670	-0.0283	0.0051	5.50E-09	0.0003	30.8025	mean cIMT
Cholesteryl Esters in Medium HDL	rs2245221	8	A	G	0.5590	0.0322	0.0039	1.60E-15	0.0006	69.1014	mean cIMT
Cholesteryl Esters in Medium HDL	rs2298428	22	T	C	0.1826	-0.0394	0.0049	3.50E-16	0.0006	63.5666	mean cIMT
Cholesteryl Esters in Medium HDL	rs2395943	6	G	A	0.5884	0.0236	0.0039	1.70E-09	0.0003	37.4089	mean cIMT
Cholesteryl Esters in Medium HDL	rs2494748	14	T	C	0.6162	-0.0222	0.0039	3.30E-09	0.0003	31.8848	mean cIMT
Cholesteryl Esters in Medium HDL	rs2642438	1	G	A	0.7038	0.0350	0.0042	9.70E-18	0.0006	70.1647	mean cIMT

Cholesteryl Esters in Medium HDL	rs267738	1	G	T	0.2196	0.0391	0.0046	1.80E-18	0.0006	72.1352	mean cIMT
Cholesteryl Esters in Medium HDL	rs2740488	9	C	A	0.2653	-0.0376	0.0043	5.30E-19	0.0007	75.1222	mean cIMT
Cholesteryl Esters in Medium HDL	rs2792735	10	A	G	0.7203	-0.0379	0.0042	2.00E-20	0.0007	79.5041	mean cIMT
Cholesteryl Esters in Medium HDL	rs28746853	6	C	T	0.1765	0.0303	0.0055	4.00E-08	0.0003	30.3503	mean cIMT
Cholesteryl Esters in Medium HDL	rs28818616	3	C	T	0.3498	-0.0215	0.0040	2.30E-08	0.0002	28.6232	mean cIMT
Cholesteryl Esters in Medium HDL	rs2925979	16	C	T	0.6989	0.0349	0.0041	8.20E-19	0.0006	71.0459	mean cIMT
Cholesteryl Esters in Medium HDL	rs2943650	2	T	C	0.6448	-0.0342	0.0040	1.70E-19	0.0006	73.9587	mean cIMT
Cholesteryl Esters in Medium HDL	rs35135293	2	T	C	0.5167	-0.0231	0.0038	6.70E-10	0.0003	36.2075	mean cIMT
Cholesteryl Esters in Medium HDL	rs35184771	11	T	G	0.3528	-0.0362	0.0040	1.90E-19	0.0007	82.7233	mean cIMT
Cholesteryl Esters in Medium HDL	rs3768321	1	T	G	0.1965	-0.0436	0.0048	9.30E-21	0.0007	82.1264	mean cIMT
Cholesteryl Esters in Medium HDL	rs3795269	1	A	C	0.4386	0.0207	0.0038	3.30E-08	0.0003	29.3106	mean cIMT
Cholesteryl Esters in Medium HDL	rs4239651	20	C	T	0.7942	0.0352	0.0047	4.70E-15	0.0005	55.6152	mean cIMT
Cholesteryl Esters in Medium HDL	rs4240624	8	A	G	0.9092	0.1029	0.0066	4.40E-55	0.0021	240.2751	mean cIMT
Cholesteryl Esters in Medium HDL	rs429358	19	C	T	0.1548	-0.0793	0.0053	4.20E-54	0.0020	225.2268	mean cIMT
Cholesteryl Esters in Medium HDL	rs4330777	16	A	G	0.4759	-0.0198	0.0038	3.40E-08	0.0002	26.7583	mean cIMT
Cholesteryl Esters in Medium HDL	rs4656292	1	G	A	0.6196	0.0212	0.0039	3.90E-09	0.0003	29.0616	mean cIMT
Cholesteryl Esters in Medium HDL	rs4846921	1	A	G	0.6132	0.0525	0.0039	1.10E-42	0.0016	179.9243	mean cIMT
Cholesteryl Esters in Medium HDL	rs4969141	17	T	C	0.4895	0.0246	0.0038	5.50E-11	0.0004	41.4358	mean cIMT
Cholesteryl Esters in Medium HDL	rs583104	1	T	G	0.7734	-0.0417	0.0046	5.60E-21	0.0007	84.1185	mean cIMT
Cholesteryl Esters in Medium HDL	rs59097294	11	C	T	0.1815	0.0643	0.0049	3.60E-41	0.0015	169.4712	mean cIMT
Cholesteryl Esters in Medium HDL	rs60847460	10	T	C	0.1433	-0.0380	0.0055	9.40E-12	0.0004	48.3335	mean cIMT
Cholesteryl Esters in Medium HDL	rs61805076	1	C	T	0.3341	-0.0276	0.0040	3.90E-12	0.0004	46.8048	mean cIMT
Cholesteryl Esters in Medium HDL	rs61941676	12	A	C	0.1272	-0.0525	0.0059	1.60E-19	0.0007	79.9683	mean cIMT
Cholesteryl Esters in Medium HDL	rs62101705	18	T	C	0.0136	-0.0903	0.0167	2.60E-08	0.0003	29.0520	mean cIMT
Cholesteryl Esters in Medium HDL	rs6589565	11	G	A	0.9322	0.0785	0.0076	4.70E-27	0.0009	106.7345	mean cIMT
Cholesteryl Esters in Medium HDL	rs6606717	12	C	A	0.5232	0.0261	0.0038	3.90E-12	0.0004	46.7270	mean cIMT
Cholesteryl Esters in Medium HDL	rs676210	2	A	G	0.2058	0.0772	0.0047	1.10E-59	0.0023	269.4181	mean cIMT
Cholesteryl Esters in Medium HDL	rs686030	9	A	C	0.8595	0.0376	0.0055	3.60E-12	0.0004	46.8572	mean cIMT

Cholesteryl Esters in Medium HDL	rs6967917	7	G	A	0.4916	0.0224	0.0038	1.00E-09	0.0003	34.4220	mean cIMT
Cholesteryl Esters in Medium HDL	rs7134375	12	A	C	0.4302	0.0214	0.0038	8.80E-09	0.0003	30.9528	mean cIMT
Cholesteryl Esters in Medium HDL	rs72836561	17	T	C	0.0315	-0.1506	0.0109	1.30E-44	0.0016	189.9495	mean cIMT
Cholesteryl Esters in Medium HDL	rs737337	19	C	T	0.0768	-0.0669	0.0072	5.70E-21	0.0008	87.3732	mean cIMT
Cholesteryl Esters in Medium HDL	rs75911530	16	A	G	0.0329	-0.1538	0.0112	4.20E-44	0.0016	187.9321	mean cIMT
Cholesteryl Esters in Medium HDL	rs77960347	18	G	A	0.0132	0.2821	0.0167	1.10E-66	0.0025	286.7675	mean cIMT
Cholesteryl Esters in Medium HDL	rs7810507	7	A	G	0.2811	-0.0281	0.0042	4.70E-11	0.0004	44.0415	mean cIMT
Cholesteryl Esters in Medium HDL	rs78296522	11	A	C	0.0445	0.0653	0.0093	1.80E-12	0.0004	49.2682	mean cIMT
Cholesteryl Esters in Medium HDL	rs7956099	12	C	T	0.4750	-0.0205	0.0038	4.30E-08	0.0002	28.3889	mean cIMT
Cholesteryl Esters in Medium HDL	rs7959043	12	G	A	0.4084	0.0225	0.0039	1.30E-09	0.0003	33.4677	mean cIMT
Cholesteryl Esters in Medium HDL	rs8058512	16	T	C	0.7240	-0.0249	0.0043	8.70E-09	0.0003	33.8936	mean cIMT
Cholesteryl Esters in Medium HDL	rs838876	12	G	A	0.6814	-0.0379	0.0042	3.80E-22	0.0007	83.3231	mean cIMT
Cholesteryl Esters in Medium HDL	rs904770	16	C	T	0.8894	-0.0351	0.0061	8.60E-09	0.0003	33.4928	mean cIMT
Cholesteryl Esters in Medium HDL	rs9304381	18	T	C	0.8184	0.0848	0.0050	2.60E-67	0.0025	293.4180	mean cIMT
Cholesteryl Esters in Medium HDL	rs9491697	6	G	A	0.4636	-0.0261	0.0038	1.40E-11	0.0004	46.2558	mean cIMT
Cholesteryl Esters in Medium HDL	rs967645	17	T	C	0.5165	-0.0261	0.0038	4.50E-11	0.0004	46.7707	mean cIMT
Cholesteryl Esters in Medium HDL	rs9687846	5	A	G	0.2014	-0.0322	0.0048	9.00E-12	0.0004	46.0723	mean cIMT
Cholesteryl Esters in Large HDL	rs1006656	17	A	G	0.8810	0.0310	0.0056	8.90E-09	0.0003	30.1069	mean cIMT
Cholesteryl Esters in Large HDL	rs1024137	2	T	G	0.6465	-0.0435	0.0038	5.20E-33	0.0011	130.9919	mean cIMT
Cholesteryl Esters in Large HDL	rs10468017	15	T	C	0.2959	0.1345	0.0040	1.00E-200	0.0097	1131.8819	mean cIMT
Cholesteryl Esters in Large HDL	rs1054852	12	G	A	0.3775	0.0325	0.0039	2.40E-17	0.0006	67.5673	mean cIMT
Cholesteryl Esters in Large HDL	rs10913592	1	A	G	0.3813	0.0207	0.0038	1.20E-08	0.0003	29.9900	mean cIMT
Cholesteryl Esters in Large HDL	rs11045171	12	G	A	0.1974	0.0295	0.0046	1.30E-10	0.0004	40.9416	mean cIMT
Cholesteryl Esters in Large HDL	rs11065991	12	T	C	0.4159	-0.0206	0.0037	5.80E-10	0.0003	30.8219	mean cIMT
Cholesteryl Esters in Large HDL	rs11075253	16	A	C	0.2958	0.0293	0.0040	1.50E-14	0.0005	54.1748	mean cIMT
Cholesteryl Esters in Large HDL	rs11122450	1	G	T	0.6115	0.0429	0.0037	1.20E-32	0.0011	131.3106	mean cIMT
Cholesteryl Esters in Large HDL	rs111432585	7	GT	G	0.9077	-0.0369	0.0066	8.50E-09	0.0003	31.0229	mean cIMT
Cholesteryl Esters in Large HDL	rs112001035	17	A	G	0.0597	-0.0483	0.0079	1.70E-09	0.0003	37.8086	mean cIMT

Cholesteryl Esters in Large HDL	rs1139490	4	T	C	0.6970	-0.0241	0.0040	5.70E-10	0.0003	36.7642	mean cIMT
Cholesteryl Esters in Large HDL	rs11429307	5	G	GT	0.8085	0.0501	0.0047	3.60E-29	0.0010	116.2445	mean cIMT
Cholesteryl Esters in Large HDL	rs116843064	19	A	G	0.0199	0.2101	0.0131	2.10E-59	0.0022	257.4355	mean cIMT
Cholesteryl Esters in Large HDL	rs11789603	9	T	C	0.1088	0.0380	0.0059	6.80E-10	0.0004	41.7949	mean cIMT
Cholesteryl Esters in Large HDL	rs11922042	3	C	T	0.0172	-0.0959	0.0151	1.40E-10	0.0004	40.5379	mean cIMT
Cholesteryl Esters in Large HDL	rs12103674	17	C	T	0.0795	-0.0410	0.0068	3.00E-09	0.0003	36.8429	mean cIMT
Cholesteryl Esters in Large HDL	rs1215112	9	A	G	0.8630	0.0474	0.0053	5.60E-20	0.0007	79.7158	mean cIMT
Cholesteryl Esters in Large HDL	rs12369443	12	G	A	0.1995	0.0240	0.0046	3.80E-08	0.0002	27.2912	mean cIMT
Cholesteryl Esters in Large HDL	rs12510382	4	G	A	0.4599	0.0222	0.0037	2.90E-10	0.0003	36.2432	mean cIMT
Cholesteryl Esters in Large HDL	rs1260326	2	C	T	0.6040	0.0408	0.0037	3.60E-29	0.0010	120.0691	mean cIMT
Cholesteryl Esters in Large HDL	rs13107325	4	T	C	0.0743	-0.0672	0.0070	7.50E-24	0.0008	93.4569	mean cIMT
Cholesteryl Esters in Large HDL	rs13118477	4	A	G	0.3904	-0.0236	0.0037	7.20E-10	0.0003	39.7129	mean cIMT
Cholesteryl Esters in Large HDL	rs13191810	6	T	C	0.3123	-0.0228	0.0039	5.00E-09	0.0003	33.6995	mean cIMT
Cholesteryl Esters in Large HDL	rs13217	20	A	G	0.5192	0.0221	0.0037	1.00E-08	0.0003	36.5620	mean cIMT
Cholesteryl Esters in Large HDL	rs13389219	2	T	C	0.3925	0.0405	0.0037	6.20E-29	0.0010	117.4331	mean cIMT
Cholesteryl Esters in Large HDL	rs145391587	8	C	A	0.1003	0.1499	0.0061	5.30E-142	0.0053	612.5617	mean cIMT
Cholesteryl Esters in Large HDL	rs147627829	6	A	G	0.0441	-0.0604	0.0089	3.20E-11	0.0004	45.6526	mean cIMT
Cholesteryl Esters in Large HDL	rs1560390	15	C	T	0.2197	-0.0553	0.0044	2.80E-40	0.0014	156.0969	mean cIMT
Cholesteryl Esters in Large HDL	rs174576	11	A	C	0.3500	-0.0788	0.0038	3.30E-103	0.0037	426.7586	mean cIMT
Cholesteryl Esters in Large HDL	rs17585887	6	C	T	0.5909	0.0243	0.0037	1.10E-11	0.0004	42.8820	mean cIMT
Cholesteryl Esters in Large HDL	rs190543502	15	C	T	0.0241	-0.0957	0.0120	3.00E-16	0.0006	63.9098	mean cIMT
Cholesteryl Esters in Large HDL	rs200644264	11	CG	C	0.7885	-0.0439	0.0045	2.90E-23	0.0008	96.5290	mean cIMT
Cholesteryl Esters in Large HDL	rs2066714	9	C	T	0.1289	0.0295	0.0054	1.00E-08	0.0003	29.5801	mean cIMT
Cholesteryl Esters in Large HDL	rs2197186	4	C	T	0.2637	0.0223	0.0042	2.00E-08	0.0002	28.7092	mean cIMT
Cholesteryl Esters in Large HDL	rs2229357	12	A	G	0.2409	0.0313	0.0043	1.50E-14	0.0005	54.3152	mean cIMT
Cholesteryl Esters in Large HDL	rs2302263	11	T	C	0.0881	-0.0416	0.0065	4.50E-11	0.0004	41.5565	mean cIMT
Cholesteryl Esters in Large HDL	rs2307111	5	C	T	0.3966	0.0269	0.0037	4.80E-13	0.0005	51.9574	mean cIMT
Cholesteryl Esters in Large HDL	rs2642438	1	G	A	0.7038	0.0304	0.0040	4.60E-15	0.0005	58.0141	mean cIMT

Cholesteryl Esters in Large HDL	rs2737205	8	C	T	0.5614	0.0257	0.0037	1.20E-11	0.0004	48.4406	mean cIMT
Cholesteryl Esters in Large HDL	rs2740488	9	C	A	0.2653	-0.0357	0.0041	4.60E-19	0.0006	74.3730	mean cIMT
Cholesteryl Esters in Large HDL	rs2925339	15	G	A	0.6730	-0.0227	0.0039	2.10E-08	0.0003	34.0088	mean cIMT
Cholesteryl Esters in Large HDL	rs2925979	16	C	T	0.6989	0.0393	0.0040	2.70E-24	0.0009	98.6879	mean cIMT
Cholesteryl Esters in Large HDL	rs333947	1	A	G	0.1501	-0.0302	0.0051	8.40E-10	0.0003	35.1139	mean cIMT
Cholesteryl Esters in Large HDL	rs34062580	7	A	G	0.1303	0.0740	0.0054	3.20E-44	0.0016	186.0855	mean cIMT
Cholesteryl Esters in Large HDL	rs36226283	12	A	G	0.3590	0.0371	0.0038	2.70E-22	0.0008	94.6867	mean cIMT
Cholesteryl Esters in Large HDL	rs3764261	16	A	C	0.3244	0.2165	0.0039	1.00E-200	0.0264	3116.4708	mean cIMT
Cholesteryl Esters in Large HDL	rs390387	22	G	A	0.8127	0.0260	0.0048	4.50E-08	0.0003	29.4464	mean cIMT
Cholesteryl Esters in Large HDL	rs4418728	10	T	G	0.4507	0.0286	0.0037	5.10E-15	0.0005	60.6396	mean cIMT
Cholesteryl Esters in Large HDL	rs4795386	17	G	A	0.7230	0.0220	0.0041	5.90E-09	0.0002	28.7286	mean cIMT
Cholesteryl Esters in Large HDL	rs4812994	20	A	C	0.5305	0.0270	0.0037	4.20E-13	0.0005	53.4353	mean cIMT
Cholesteryl Esters in Large HDL	rs488490	15	A	C	0.8526	-0.1318	0.0052	4.00E-148	0.0055	641.7736	mean cIMT
Cholesteryl Esters in Large HDL	rs4922787	11	T	G	0.7616	0.0257	0.0043	8.50E-10	0.0003	35.6868	mean cIMT
Cholesteryl Esters in Large HDL	rs492699	12	C	T	0.2248	0.0286	0.0046	1.90E-10	0.0003	39.3661	mean cIMT
Cholesteryl Esters in Large HDL	rs5082	1	A	G	0.6207	-0.0239	0.0038	6.50E-10	0.0003	40.0858	mean cIMT
Cholesteryl Esters in Large HDL	rs5167	19	G	T	0.3523	0.0392	0.0038	8.90E-26	0.0009	105.5600	mean cIMT
Cholesteryl Esters in Large HDL	rs55714927	17	T	C	0.1910	0.0258	0.0046	5.50E-09	0.0003	30.9160	mean cIMT
Cholesteryl Esters in Large HDL	rs57912727	3	C	A	0.1367	-0.0305	0.0053	1.70E-09	0.0003	32.6640	mean cIMT
Cholesteryl Esters in Large HDL	rs59104589	2	T	C	0.3588	0.0248	0.0038	1.10E-12	0.0004	42.4091	mean cIMT
Cholesteryl Esters in Large HDL	rs6073958	20	C	T	0.1987	-0.1619	0.0046	1.00E-200	0.0107	1249.7225	mean cIMT
Cholesteryl Esters in Large HDL	rs60847460	10	T	C	0.1433	-0.0313	0.0052	1.70E-09	0.0003	35.9363	mean cIMT
Cholesteryl Esters in Large HDL	rs61781392	1	A	C	0.2167	-0.0332	0.0044	1.30E-15	0.0005	56.0965	mean cIMT
Cholesteryl Esters in Large HDL	rs61854123	10	A	G	0.2427	0.0300	0.0043	7.40E-13	0.0004	49.3636	mean cIMT
Cholesteryl Esters in Large HDL	rs6694102	1	A	G	0.3209	-0.0336	0.0039	2.90E-18	0.0006	74.1266	mean cIMT
Cholesteryl Esters in Large HDL	rs673548	2	A	G	0.2061	0.0578	0.0045	2.60E-37	0.0014	165.4949	mean cIMT
Cholesteryl Esters in Large HDL	rs688671	18	G	A	0.2671	-0.0245	0.0041	1.40E-09	0.0003	34.8583	mean cIMT
Cholesteryl Esters in Large HDL	rs6993128	8	T	C	0.7290	0.0240	0.0041	1.40E-08	0.0003	33.7746	mean cIMT

Cholesteryl Esters in Large HDL	rs7009450	8	T	G	0.2100	-0.0258	0.0045	1.10E-09	0.0003	33.0980	mean cIMT
Cholesteryl Esters in Large HDL	rs7012814	8	A	G	0.4743	0.0349	0.0037	6.50E-21	0.0008	89.9191	mean cIMT
Cholesteryl Esters in Large HDL	rs705379	7	A	G	0.4775	-0.0232	0.0037	4.80E-11	0.0003	39.6771	mean cIMT
Cholesteryl Esters in Large HDL	rs71205961	8	C	CTTTTTT	0.0890	-0.0419	0.0072	8.80E-09	0.0003	34.1444	mean cIMT
Cholesteryl Esters in Large HDL	rs7241918	18	T	G	0.8229	0.0513	0.0048	2.70E-27	0.0010	114.2488	mean cIMT
Cholesteryl Esters in Large HDL	rs72836561	17	T	C	0.0315	-0.1444	0.0105	7.80E-47	0.0017	190.9000	mean cIMT
Cholesteryl Esters in Large HDL	rs72934503	6	G	A	0.4514	0.0237	0.0037	4.40E-10	0.0003	39.9991	mean cIMT
Cholesteryl Esters in Large HDL	rs7308864	12	G	A	0.5233	0.0225	0.0036	1.60E-10	0.0003	38.0698	mean cIMT
Cholesteryl Esters in Large HDL	rs737337	19	C	T	0.0768	-0.0453	0.0069	1.60E-11	0.0004	43.7090	mean cIMT
Cholesteryl Esters in Large HDL	rs7475340	10	G	A	0.5156	-0.0184	0.0037	3.40E-08	0.0002	25.2133	mean cIMT
Cholesteryl Esters in Large HDL	rs75627662	19	T	C	0.2061	-0.0421	0.0045	5.70E-22	0.0008	87.0177	mean cIMT
Cholesteryl Esters in Large HDL	rs75714888	16	A	C	0.0216	-0.0839	0.0126	5.20E-11	0.0004	44.6059	mean cIMT
Cholesteryl Esters in Large HDL	rs75911530	16	A	G	0.0329	-0.1490	0.0107	1.40E-45	0.0017	193.5379	mean cIMT
Cholesteryl Esters in Large HDL	rs759819	19	C	T	0.3267	0.0242	0.0039	1.30E-10	0.0003	38.4389	mean cIMT
Cholesteryl Esters in Large HDL	rs77960347	18	G	A	0.0132	0.1810	0.0160	1.10E-30	0.0011	128.7185	mean cIMT
Cholesteryl Esters in Large HDL	rs78058190	2	A	G	0.0501	-0.0652	0.0094	2.80E-11	0.0004	47.6592	mean cIMT
Cholesteryl Esters in Large HDL	rs7810507	7	A	G	0.2811	-0.0343	0.0041	7.40E-17	0.0006	71.6076	mean cIMT
Cholesteryl Esters in Large HDL	rs7924036	10	T	G	0.5042	0.0214	0.0036	6.60E-10	0.0003	34.2659	mean cIMT
Cholesteryl Esters in Large HDL	rs79598313	1	T	C	0.0232	-0.0918	0.0121	2.20E-13	0.0005	57.4285	mean cIMT
Cholesteryl Esters in Large HDL	rs8058512	16	T	C	0.7240	-0.0245	0.0041	3.00E-09	0.0003	36.2543	mean cIMT
Cholesteryl Esters in Large HDL	rs921919	12	A	G	0.6695	-0.0429	0.0040	9.20E-30	0.0010	117.0232	mean cIMT
Cholesteryl Esters in Large HDL	rs9265113	6	T	C	0.4076	-0.0278	0.0040	9.10E-13	0.0004	48.8850	mean cIMT
Cholesteryl Esters in Large HDL	rs9491697	6	G	A	0.4636	-0.0301	0.0037	1.30E-17	0.0006	67.2936	mean cIMT
Cholesteryl Esters in Large HDL	rs9769088	7	C	T	0.3909	-0.0312	0.0038	1.70E-17	0.0006	68.7368	mean cIMT
Cholesteryl Esters in Large HDL	rs9955201	18	A	G	0.0338	0.0595	0.0102	4.40E-09	0.0003	33.9206	mean cIMT
Cholesteryl Esters in Large HDL	rs998584	6	A	C	0.4823	-0.0381	0.0037	2.20E-26	0.0009	108.8079	mean cIMT
Phospholipids in HDL	rs10184004	2	T	C	0.4061	0.0218	0.0038	1.70E-09	0.0003	32.9692	mean cIMT
Phospholipids in HDL	rs1065853	19	T	G	0.0806	0.1036	0.0069	1.00E-54	0.0020	226.1758	mean cIMT

Phospholipids in HDL	rs112001035	17	A	G	0.0597	-0.0476	0.0080	1.10E-08	0.0003	35.1664	mean cIMT
Phospholipids in HDL	rs112495680	6	G	A	0.1334	-0.0364	0.0055	1.80E-11	0.0004	43.6294	mean cIMT
Phospholipids in HDL	rs11671872	19	A	G	0.4125	0.0260	0.0038	1.50E-11	0.0004	46.3437	mean cIMT
Phospholipids in HDL	rs11789603	9	T	C	0.1088	0.0518	0.0060	9.40E-17	0.0006	74.2287	mean cIMT
Phospholipids in HDL	rs12608026	18	G	T	0.0424	0.0930	0.0093	3.40E-24	0.0009	98.9389	mean cIMT
Phospholipids in HDL	rs12976739	19	A	G	0.3962	0.0265	0.0038	9.10E-13	0.0004	47.5890	mean cIMT
Phospholipids in HDL	rs13107325	4	T	C	0.0743	-0.0688	0.0071	2.40E-23	0.0008	93.3465	mean cIMT
Phospholipids in HDL	rs139915535	8	G	A	0.0180	-0.1237	0.0141	4.20E-20	0.0007	77.1031	mean cIMT
Phospholipids in HDL	rs140584594	1	G	A	0.7301	0.0313	0.0042	2.60E-14	0.0005	55.9058	mean cIMT
Phospholipids in HDL	rs141368429	11	T	C	0.0562	-0.0484	0.0087	2.90E-08	0.0003	30.8403	mean cIMT
Phospholipids in HDL	rs148063610	10	C	CAAATAAAT	0.7634	-0.0342	0.0045	1.10E-14	0.0005	58.0435	mean cIMT
Phospholipids in HDL	rs150224153	20	T	C	0.0292	-0.0775	0.0114	3.30E-11	0.0004	46.4559	mean cIMT
Phospholipids in HDL	rs15285	8	T	C	0.2865	0.0812	0.0041	8.50E-88	0.0033	386.7159	mean cIMT
Phospholipids in HDL	rs174574	11	C	A	0.6494	0.0490	0.0039	8.60E-39	0.0014	157.5000	mean cIMT
Phospholipids in HDL	rs193084249	1	G	A	0.0234	-0.0931	0.0126	1.20E-13	0.0005	54.4433	mean cIMT
Phospholipids in HDL	rs2043085	15	C	T	0.6127	-0.1271	0.0038	1.00E-200	0.0095	1103.1801	mean cIMT
Phospholipids in HDL	rs2066714	9	C	T	0.1289	0.0303	0.0056	1.60E-08	0.0003	29.8627	mean cIMT
Phospholipids in HDL	rs2070895	15	A	G	0.2187	0.1399	0.0045	1.00E-200	0.0083	961.1266	mean cIMT
Phospholipids in HDL	rs2176040	2	G	A	0.6467	-0.0291	0.0039	1.80E-15	0.0005	55.5928	mean cIMT
Phospholipids in HDL	rs2236252	20	T	C	0.1670	-0.0297	0.0050	9.80E-10	0.0003	35.3371	mean cIMT
Phospholipids in HDL	rs2243616	12	T	G	0.6611	-0.0230	0.0040	1.50E-08	0.0003	33.7600	mean cIMT
Phospholipids in HDL	rs2476559	1	G	A	0.4111	0.0203	0.0038	1.80E-08	0.0002	28.4641	mean cIMT
Phospholipids in HDL	rs2642438	1	G	A	0.7038	0.0359	0.0041	9.50E-20	0.0007	76.8928	mean cIMT
Phospholipids in HDL	rs267738	1	G	T	0.2196	0.0313	0.0045	5.60E-13	0.0004	48.1437	mean cIMT
Phospholipids in HDL	rs2740488	9	C	A	0.2653	-0.0413	0.0042	1.50E-23	0.0008	94.8005	mean cIMT
Phospholipids in HDL	rs2781751	19	A	G	0.3155	0.0205	0.0040	3.40E-08	0.0002	25.8033	mean cIMT
Phospholipids in HDL	rs2792735	10	A	G	0.7203	-0.0415	0.0042	1.30E-24	0.0009	99.5098	mean cIMT
Phospholipids in HDL	rs28818616	3	C	T	0.3498	-0.0214	0.0039	3.20E-08	0.0003	29.4509	mean cIMT

Phospholipids in HDL	rs2925979	16	C	T	0.6989	0.0301	0.0041	5.30E-15	0.0005	54.9479	mean cIMT
Phospholipids in HDL	rs34663616	15	A	C	0.1377	0.0421	0.0055	6.00E-15	0.0005	58.0632	mean cIMT
Phospholipids in HDL	rs34707604	4	C	T	0.2585	0.0279	0.0045	4.50E-11	0.0003	38.4109	mean cIMT
Phospholipids in HDL	rs34955778	16	C	T	0.4200	-0.0211	0.0038	1.80E-08	0.0003	31.0915	mean cIMT
Phospholipids in HDL	rs35184771	11	T	G	0.3528	-0.0352	0.0039	2.00E-19	0.0007	81.5878	mean cIMT
Phospholipids in HDL	rs3735687	7	G	A	0.4228	-0.0261	0.0038	2.60E-11	0.0004	47.2094	mean cIMT
Phospholipids in HDL	rs3764261	16	A	C	0.3244	0.1784	0.0040	1.00E-200	0.0172	2011.3305	mean cIMT
Phospholipids in HDL	rs3768321	1	T	G	0.1965	-0.0359	0.0047	2.90E-15	0.0005	58.1166	mean cIMT
Phospholipids in HDL	rs41272663	2	A	C	0.2640	0.0247	0.0042	5.00E-09	0.0003	33.9916	mean cIMT
Phospholipids in HDL	rs4240624	8	A	G	0.9092	0.0905	0.0065	2.70E-44	0.0017	193.6032	mean cIMT
Phospholipids in HDL	rs4846921	1	A	G	0.6132	0.0511	0.0038	2.10E-42	0.0015	178.1142	mean cIMT
Phospholipids in HDL	rs599839	1	A	G	0.7721	-0.0382	0.0044	6.30E-19	0.0006	74.0639	mean cIMT
Phospholipids in HDL	rs6018652	20	A	G	0.7925	0.0378	0.0046	2.10E-17	0.0006	66.9133	mean cIMT
Phospholipids in HDL	rs6073958	20	C	T	0.1987	-0.0520	0.0047	6.10E-28	0.0011	123.3046	mean cIMT
Phospholipids in HDL	rs60847460	10	T	C	0.1433	-0.0380	0.0053	1.80E-12	0.0004	50.4830	mean cIMT
Phospholipids in HDL	rs61805076	1	C	T	0.3341	-0.0284	0.0040	2.00E-13	0.0004	51.4666	mean cIMT
Phospholipids in HDL	rs61941676	12	A	C	0.1272	-0.0606	0.0057	1.10E-26	0.0010	111.2957	mean cIMT
Phospholipids in HDL	rs638714	1	T	G	0.3459	-0.0464	0.0039	7.50E-33	0.0012	139.1358	mean cIMT
Phospholipids in HDL	rs6606717	12	C	A	0.5232	0.0324	0.0037	1.00E-18	0.0007	75.4596	mean cIMT
Phospholipids in HDL	rs676210	2	A	G	0.2058	0.0587	0.0046	1.10E-36	0.0014	162.6431	mean cIMT
Phospholipids in HDL	rs686030	9	A	C	0.8595	0.0412	0.0054	3.70E-15	0.0005	58.8035	mean cIMT
Phospholipids in HDL	rs7231011	18	C	T	0.1415	0.0298	0.0054	3.00E-08	0.0003	30.9253	mean cIMT
Phospholipids in HDL	rs7241918	18	T	G	0.8229	0.0950	0.0049	5.10E-86	0.0033	375.2317	mean cIMT
Phospholipids in HDL	rs72836561	17	T	C	0.0315	-0.1283	0.0107	1.00E-34	0.0012	143.8683	mean cIMT
Phospholipids in HDL	rs737338	19	T	C	0.0352	-0.1210	0.0101	1.10E-34	0.0012	142.6237	mean cIMT
Phospholipids in HDL	rs75911530	16	A	G	0.0329	-0.1360	0.0110	2.90E-37	0.0013	153.2119	mean cIMT
Phospholipids in HDL	rs77960347	18	G	A	0.0132	0.3221	0.0163	8.60E-90	0.0034	390.2079	mean cIMT
Phospholipids in HDL	rs7810507	7	A	G	0.2811	-0.0235	0.0042	1.60E-08	0.0003	32.1766	mean cIMT

Phospholipids in HDL	rs7956099	12	C	T	0.4750	-0.0232	0.0038	2.70E-10	0.0003	38.2861	mean cIMT
Phospholipids in HDL	rs8058512	16	T	C	0.7240	-0.0241	0.0042	8.40E-09	0.0003	33.1704	mean cIMT
Phospholipids in HDL	rs838876	12	G	A	0.6814	-0.0401	0.0041	1.80E-25	0.0008	97.4368	mean cIMT
Phospholipids in HDL	rs907866	2	A	G	0.4432	-0.0310	0.0038	9.70E-17	0.0006	67.4286	mean cIMT
Phospholipids in HDL	rs9471972	6	A	G	0.5362	0.0236	0.0037	4.60E-10	0.0003	39.8827	mean cIMT
Phospholipids in HDL	rs9491697	6	G	A	0.4636	-0.0225	0.0038	2.10E-09	0.0003	35.8393	mean cIMT
Phospholipids in HDL	rs967645	17	T	C	0.5165	-0.0288	0.0037	1.00E-13	0.0005	59.5559	mean cIMT
Phospholipids in HDL	rs9687833	5	A	G	0.2070	-0.0259	0.0046	7.50E-09	0.0003	31.5378	mean cIMT
Phospholipids in Large HDL	rs1006656	17	A	G	0.8810	0.0300	0.0056	4.10E-08	0.0002	28.3402	mean cIMT
Phospholipids in Large HDL	rs1024137	2	T	G	0.6465	-0.0370	0.0038	2.90E-25	0.0008	95.4324	mean cIMT
Phospholipids in Large HDL	rs10468017	15	T	C	0.2959	0.1619	0.0040	1.00E-200	0.0142	1654.0007	mean cIMT
Phospholipids in Large HDL	rs1057208	20	T	C	0.1863	-0.1497	0.0047	1.00E-200	0.0088	1025.4914	mean cIMT
Phospholipids in Large HDL	rs1077195	12	T	G	0.4059	0.0216	0.0037	5.60E-10	0.0003	33.1956	mean cIMT
Phospholipids in Large HDL	rs11045171	12	G	A	0.1974	0.0272	0.0046	1.70E-09	0.0003	35.0912	mean cIMT
Phospholipids in Large HDL	rs11057469	12	A	G	0.4239	0.0232	0.0037	5.30E-09	0.0003	38.6178	mean cIMT
Phospholipids in Large HDL	rs112001035	17	A	G	0.0597	-0.0468	0.0078	3.90E-09	0.0003	35.8232	mean cIMT
Phospholipids in Large HDL	rs112875651	8	A	G	0.3924	0.0272	0.0038	6.80E-15	0.0004	51.7809	mean cIMT
Phospholipids in Large HDL	rs1139490	4	T	C	0.6970	-0.0237	0.0040	1.30E-09	0.0003	35.8966	mean cIMT
Phospholipids in Large HDL	rs11429307	5	G	GT	0.8085	0.0425	0.0046	2.10E-21	0.0007	83.8725	mean cIMT
Phospholipids in Large HDL	rs116843064	19	A	G	0.0199	0.1825	0.0131	1.30E-45	0.0017	195.6595	mean cIMT
Phospholipids in Large HDL	rs11751347	6	T	C	0.1023	-0.0491	0.0060	7.50E-17	0.0006	66.2498	mean cIMT
Phospholipids in Large HDL	rs11789603	9	T	C	0.1088	0.0420	0.0059	3.40E-12	0.0004	51.5268	mean cIMT
Phospholipids in Large HDL	rs12510382	4	G	A	0.4599	0.0207	0.0037	9.50E-09	0.0003	31.6234	mean cIMT
Phospholipids in Large HDL	rs12721046	19	A	G	0.1577	-0.0367	0.0050	1.30E-13	0.0005	53.7111	mean cIMT
Phospholipids in Large HDL	rs13107325	4	T	C	0.0743	-0.0697	0.0069	5.00E-25	0.0009	101.1064	mean cIMT
Phospholipids in Large HDL	rs1319424	9	A	G	0.7431	0.0221	0.0042	3.70E-08	0.0002	28.0582	mean cIMT
Phospholipids in Large HDL	rs13389219	2	T	C	0.3925	0.0343	0.0037	1.60E-21	0.0007	84.9550	mean cIMT
Phospholipids in Large HDL	rs145391587	8	C	A	0.1003	0.1344	0.0060	3.40E-116	0.0043	495.8959	mean cIMT

Phospholipids in Large HDL	rs1457489	18	A	G	0.2646	-0.0232	0.0041	1.40E-08	0.0003	31.4457	mean cIMT
Phospholipids in Large HDL	rs147627829	6	A	G	0.0441	-0.0614	0.0089	8.70E-12	0.0004	47.4528	mean cIMT
Phospholipids in Large HDL	rs148063610	10	C	CAAATAAAT	0.7634	-0.0321	0.0044	5.20E-14	0.0005	54.1718	mean cIMT
Phospholipids in Large HDL	rs150224153	20	T	C	0.0292	-0.0696	0.0111	1.30E-10	0.0003	39.4587	mean cIMT
Phospholipids in Large HDL	rs1560390	15	C	T	0.2197	-0.0662	0.0044	2.50E-57	0.0020	225.6426	mean cIMT
Phospholipids in Large HDL	rs174576	11	A	C	0.3500	-0.0763	0.0038	1.30E-97	0.0035	402.2613	mean cIMT
Phospholipids in Large HDL	rs17729883	8	C	T	0.3346	-0.0206	0.0039	3.80E-08	0.0002	27.9785	mean cIMT
Phospholipids in Large HDL	rs200644264	11	CG	C	0.7885	-0.0401	0.0045	8.90E-20	0.0007	81.3279	mean cIMT
Phospholipids in Large HDL	rs2066714	9	C	T	0.1289	0.0292	0.0054	2.50E-08	0.0003	29.2138	mean cIMT
Phospholipids in Large HDL	rs2169387	8	G	A	0.9000	0.0576	0.0061	1.10E-20	0.0008	89.3431	mean cIMT
Phospholipids in Large HDL	rs2229357	12	A	G	0.2409	0.0273	0.0042	1.50E-11	0.0004	41.6197	mean cIMT
Phospholipids in Large HDL	rs2307111	5	C	T	0.3966	0.0215	0.0037	9.30E-09	0.0003	33.5114	mean cIMT
Phospholipids in Large HDL	rs2642438	1	G	A	0.7038	0.0334	0.0040	1.30E-18	0.0006	70.3963	mean cIMT
Phospholipids in Large HDL	rs2696455	17	T	C	0.1959	-0.0270	0.0048	1.40E-08	0.0003	31.2621	mean cIMT
Phospholipids in Large HDL	rs2737205	8	C	T	0.5614	0.0294	0.0037	3.00E-15	0.0006	63.7640	mean cIMT
Phospholipids in Large HDL	rs2740488	9	C	A	0.2653	-0.0359	0.0041	2.50E-19	0.0007	75.5069	mean cIMT
Phospholipids in Large HDL	rs2925979	16	C	T	0.6989	0.0351	0.0039	3.50E-20	0.0007	79.3164	mean cIMT
Phospholipids in Large HDL	rs333947	1	A	G	0.1501	-0.0289	0.0051	3.30E-09	0.0003	32.3734	mean cIMT
Phospholipids in Large HDL	rs34695955	17	G	T	0.0795	-0.0380	0.0067	2.80E-08	0.0003	31.9043	mean cIMT
Phospholipids in Large HDL	rs368166328	4	C	CT	0.7599	-0.0282	0.0050	4.50E-09	0.0003	31.4567	mean cIMT
Phospholipids in Large HDL	rs3735687	7	G	A	0.4228	-0.0331	0.0037	4.50E-19	0.0007	80.3549	mean cIMT
Phospholipids in Large HDL	rs3764261	16	A	C	0.3244	0.2086	0.0039	1.00E-200	0.0247	2909.2573	mean cIMT
Phospholipids in Large HDL	rs4330777	16	A	G	0.4759	-0.0192	0.0036	2.80E-08	0.0002	27.9404	mean cIMT
Phospholipids in Large HDL	rs4418728	10	T	G	0.4507	0.0236	0.0037	1.30E-10	0.0004	41.5911	mean cIMT
Phospholipids in Large HDL	rs4846921	1	A	G	0.6132	0.0476	0.0037	6.40E-40	0.0014	162.9296	mean cIMT
Phospholipids in Large HDL	rs488490	15	A	C	0.8526	-0.1550	0.0052	1.00E-200	0.0077	894.9588	mean cIMT
Phospholipids in Large HDL	rs5030789	15	G	A	0.5673	0.0206	0.0037	5.10E-09	0.0003	31.5378	mean cIMT
Phospholipids in Large HDL	rs5167	19	G	T	0.3523	0.0434	0.0038	2.00E-31	0.0011	130.1795	mean cIMT

Phospholipids in Large HDL	rs55714927	17	T	C	0.1910	0.0244	0.0046	2.40E-08	0.0002	27.9044	mean cIMT
Phospholipids in Large HDL	rs5754100	22	C	T	0.1883	-0.0280	0.0047	1.10E-09	0.0003	36.0115	mean cIMT
Phospholipids in Large HDL	rs57912727	3	C	A	0.1367	-0.0303	0.0053	2.50E-09	0.0003	32.5380	mean cIMT
Phospholipids in Large HDL	rs58388121	1	T	TAA	0.6288	-0.0241	0.0038	6.10E-11	0.0003	39.6955	mean cIMT
Phospholipids in Large HDL	rs59104589	2	T	C	0.3588	0.0239	0.0038	3.80E-12	0.0003	39.8264	mean cIMT
Phospholipids in Large HDL	rs60847460	10	T	C	0.1433	-0.0358	0.0052	3.10E-12	0.0004	47.3602	mean cIMT
Phospholipids in Large HDL	rs61781392	1	A	C	0.2167	-0.0303	0.0044	4.10E-13	0.0004	47.1990	mean cIMT
Phospholipids in Large HDL	rs61941676	12	A	C	0.1272	-0.0618	0.0056	2.10E-29	0.0011	122.3645	mean cIMT
Phospholipids in Large HDL	rs6498540	16	G	A	0.3021	-0.0298	0.0039	6.00E-15	0.0005	57.0247	mean cIMT
Phospholipids in Large HDL	rs6694102	1	A	G	0.3209	-0.0336	0.0039	4.10E-18	0.0006	74.4178	mean cIMT
Phospholipids in Large HDL	rs676210	2	A	G	0.2058	0.0658	0.0045	1.30E-48	0.0019	215.7441	mean cIMT
Phospholipids in Large HDL	rs686030	9	A	C	0.8595	0.0475	0.0052	2.00E-20	0.0007	82.3485	mean cIMT
Phospholipids in Large HDL	rs705379	7	A	G	0.4775	-0.0247	0.0037	4.80E-12	0.0004	45.0227	mean cIMT
Phospholipids in Large HDL	rs7241918	18	T	G	0.8229	0.0665	0.0048	1.70E-45	0.0017	193.8540	mean cIMT
Phospholipids in Large HDL	rs72836561	17	T	C	0.0315	-0.1383	0.0104	5.00E-43	0.0015	176.4050	mean cIMT
Phospholipids in Large HDL	rs72934503	6	G	A	0.4514	0.0243	0.0037	5.00E-11	0.0004	42.5095	mean cIMT
Phospholipids in Large HDL	rs7308864	12	G	A	0.5233	0.0275	0.0036	5.50E-15	0.0005	57.3347	mean cIMT
Phospholipids in Large HDL	rs737337	19	C	T	0.0768	-0.0563	0.0068	2.50E-16	0.0006	68.1045	mean cIMT
Phospholipids in Large HDL	rs75460349	1	C	A	0.0233	-0.0861	0.0122	5.90E-12	0.0004	49.6563	mean cIMT
Phospholipids in Large HDL	rs75714888	16	A	C	0.0216	-0.0792	0.0125	5.80E-10	0.0003	39.9858	mean cIMT
Phospholipids in Large HDL	rs75911530	16	A	G	0.0329	-0.1426	0.0107	3.20E-43	0.0015	178.4305	mean cIMT
Phospholipids in Large HDL	rs77516617	15	A	G	0.0528	-0.0487	0.0082	2.20E-09	0.0003	35.2631	mean cIMT
Phospholipids in Large HDL	rs77960347	18	G	A	0.0132	0.2289	0.0159	9.30E-49	0.0018	207.6353	mean cIMT
Phospholipids in Large HDL	rs7810507	7	A	G	0.2811	-0.0302	0.0040	2.40E-13	0.0005	55.9391	mean cIMT
Phospholipids in Large HDL	rs7924036	10	T	G	0.5042	0.0192	0.0036	1.80E-08	0.0002	27.9804	mean cIMT
Phospholipids in Large HDL	rs79744701	8	A	G	0.1241	0.0348	0.0055	1.40E-10	0.0003	39.9980	mean cIMT
Phospholipids in Large HDL	rs8058512	16	T	C	0.7240	-0.0250	0.0041	1.00E-09	0.0003	37.8011	mean cIMT
Phospholipids in Large HDL	rs837500	12	T	C	0.6385	0.0218	0.0038	5.90E-09	0.0003	32.8871	mean cIMT

Phospholipids in Large HDL	rs838876	12	G	A	0.6814	-0.0419	0.0040	1.70E-29	0.0010	112.0918	mean cIMT
Phospholipids in Large HDL	rs907866	2	A	G	0.4432	-0.0242	0.0037	3.70E-11	0.0004	43.5271	mean cIMT
Phospholipids in Large HDL	rs9265113	6	T	C	0.4076	-0.0287	0.0040	3.20E-13	0.0005	52.4918	mean cIMT
Phospholipids in Large HDL	rs9491697	6	G	A	0.4636	-0.0291	0.0037	2.30E-16	0.0005	63.2021	mean cIMT
Phospholipids in Large HDL	rs9955201	18	A	G	0.0338	0.0760	0.0102	2.40E-14	0.0005	55.8212	mean cIMT
Phospholipids in Large HDL	rs998584	6	A	C	0.4823	-0.0330	0.0036	5.00E-20	0.0007	82.0210	mean cIMT
Mean HDL Particle Size	rs1006656	17	A	G	0.8810	0.0325	0.0057	2.30E-09	0.0003	33.0117	mean cIMT
Mean HDL Particle Size	rs1024137	2	T	G	0.6465	-0.0357	0.0038	3.80E-23	0.0008	87.3663	mean cIMT
Mean HDL Particle Size	rs10457487	6	A	C	0.4843	-0.0282	0.0037	3.20E-15	0.0005	58.5848	mean cIMT
Mean HDL Particle Size	rs1057208	20	T	C	0.1863	-0.1956	0.0047	1.00E-200	0.0148	1728.5608	mean cIMT
Mean HDL Particle Size	rs10756794	9	A	G	0.7424	0.0237	0.0042	1.80E-09	0.0003	32.0575	mean cIMT
Mean HDL Particle Size	rs10899133	11	T	C	0.1055	-0.0362	0.0060	2.80E-09	0.0003	36.6764	mean cIMT
Mean HDL Particle Size	rs11045171	12	G	A	0.1974	0.0258	0.0046	9.40E-09	0.0003	31.1410	mean cIMT
Mean HDL Particle Size	rs1126671	4	C	T	0.6979	-0.0255	0.0040	7.50E-11	0.0004	40.9687	mean cIMT
Mean HDL Particle Size	rs112875651	8	A	G	0.3924	0.0338	0.0038	7.70E-22	0.0007	79.2541	mean cIMT
Mean HDL Particle Size	rs11429307	5	G	GT	0.8085	0.0452	0.0047	2.70E-24	0.0008	93.7537	mean cIMT
Mean HDL Particle Size	rs11434143	8	G	GT	0.8108	-0.0291	0.0048	2.90E-10	0.0003	37.4884	mean cIMT
Mean HDL Particle Size	rs116843064	19	A	G	0.0199	0.1569	0.0131	1.10E-32	0.0012	142.6914	mean cIMT
Mean HDL Particle Size	rs1177562	11	T	C	0.4104	-0.0225	0.0037	1.80E-09	0.0003	36.4608	mean cIMT
Mean HDL Particle Size	rs11789603	9	T	C	0.1088	0.0372	0.0059	5.80E-10	0.0003	39.7772	mean cIMT
Mean HDL Particle Size	rs11922042	3	C	T	0.0172	-0.0851	0.0151	3.30E-08	0.0003	31.6959	mean cIMT
Mean HDL Particle Size	rs1260326	2	C	T	0.6040	0.0380	0.0037	7.60E-26	0.0009	103.2636	mean cIMT
Mean HDL Particle Size	rs12721046	19	A	G	0.1577	-0.0374	0.0050	9.50E-14	0.0005	55.2649	mean cIMT
Mean HDL Particle Size	rs12914626	15	T	C	0.7019	-0.1015	0.0040	6.50E-148	0.0055	639.1987	mean cIMT
Mean HDL Particle Size	rs13107325	4	T	C	0.0743	-0.0625	0.0070	3.30E-20	0.0007	80.3612	mean cIMT
Mean HDL Particle Size	rs13108218	4	G	A	0.6154	0.0257	0.0038	2.60E-11	0.0004	46.0753	mean cIMT
Mean HDL Particle Size	rs13118477	4	A	G	0.3904	-0.0216	0.0038	1.50E-08	0.0003	32.9816	mean cIMT
Mean HDL Particle Size	rs13191810	6	T	C	0.3123	-0.0235	0.0039	3.60E-09	0.0003	35.5585	mean cIMT

Mean HDL Particle Size	rs13389219	2	T	C	0.3925	0.0354	0.0037	1.60E-22	0.0008	89.1272	mean cIMT
Mean HDL Particle Size	rs141920044	16	T	C	0.3508	0.0223	0.0039	1.10E-08	0.0003	32.6839	mean cIMT
Mean HDL Particle Size	rs145391587	8	C	A	0.1003	0.1114	0.0061	9.40E-79	0.0029	335.5217	mean cIMT
Mean HDL Particle Size	rs147627829	6	A	G	0.0441	-0.0594	0.0090	8.00E-11	0.0004	43.7971	mean cIMT
Mean HDL Particle Size	rs1546954	1	G	T	0.6127	0.0416	0.0038	1.10E-30	0.0011	122.7052	mean cIMT
Mean HDL Particle Size	rs1560390	15	C	T	0.2197	-0.0771	0.0044	4.80E-74	0.0026	302.0709	mean cIMT
Mean HDL Particle Size	rs1569721	20	C	T	0.9619	-0.0605	0.0100	2.40E-09	0.0003	36.6098	mean cIMT
Mean HDL Particle Size	rs1943973	18	A	G	0.8493	0.0593	0.0051	1.10E-31	0.0012	133.8698	mean cIMT
Mean HDL Particle Size	rs200644264	11	CG	C	0.7885	-0.0373	0.0045	5.40E-17	0.0006	69.3814	mean cIMT
Mean HDL Particle Size	rs2066714	9	C	T	0.1289	0.0308	0.0054	5.70E-09	0.0003	31.9237	mean cIMT
Mean HDL Particle Size	rs2253398	11	T	G	0.9459	0.0443	0.0081	1.00E-08	0.0003	29.7235	mean cIMT
Mean HDL Particle Size	rs261291	15	C	T	0.3561	0.1766	0.0038	1.00E-200	0.0183	2141.2642	mean cIMT
Mean HDL Particle Size	rs2642438	1	G	A	0.7038	0.0270	0.0040	1.80E-12	0.0004	45.5245	mean cIMT
Mean HDL Particle Size	rs2696455	17	T	C	0.1959	-0.0272	0.0049	1.50E-08	0.0003	31.2664	mean cIMT
Mean HDL Particle Size	rs2737205	8	C	T	0.5614	0.0275	0.0037	1.60E-13	0.0005	55.1327	mean cIMT
Mean HDL Particle Size	rs2740488	9	C	A	0.2653	-0.0339	0.0042	2.60E-17	0.0006	66.5408	mean cIMT
Mean HDL Particle Size	rs2925979	16	C	T	0.6989	0.0328	0.0040	2.30E-17	0.0006	68.1930	mean cIMT
Mean HDL Particle Size	rs3092498	20	C	T	0.5174	0.0260	0.0037	8.30E-12	0.0004	49.7879	mean cIMT
Mean HDL Particle Size	rs34060476	7	G	A	0.1348	0.0657	0.0054	1.40E-35	0.0013	150.0134	mean cIMT
Mean HDL Particle Size	rs35067568	7	A	G	0.0899	-0.0353	0.0064	4.70E-08	0.0003	30.3109	mean cIMT
Mean HDL Particle Size	rs35135293	2	T	C	0.5167	-0.0190	0.0037	4.60E-08	0.0002	26.7064	mean cIMT
Mean HDL Particle Size	rs35473591	11	C	CT	0.6581	0.0732	0.0039	2.70E-87	0.0031	361.1967	mean cIMT
Mean HDL Particle Size	rs36037051	6	G	A	0.0534	-0.0431	0.0082	4.60E-08	0.0002	27.8976	mean cIMT
Mean HDL Particle Size	rs368166328	4	C	CT	0.7599	-0.0289	0.0051	2.30E-09	0.0003	32.7791	mean cIMT
Mean HDL Particle Size	rs4418728	10	T	G	0.4507	0.0259	0.0037	1.20E-12	0.0004	49.6663	mean cIMT
Mean HDL Particle Size	rs5082	1	A	G	0.6207	-0.0329	0.0038	7.40E-18	0.0007	75.7856	mean cIMT
Mean HDL Particle Size	rs5167	19	G	T	0.3523	0.0391	0.0038	3.30E-25	0.0009	104.4887	mean cIMT
Mean HDL Particle Size	rs55707100	15	T	C	0.0259	-0.0622	0.0115	2.60E-08	0.0003	29.2924	mean cIMT

Mean HDL Particle Size	rs55714927	17	T	C	0.1910	0.0358	0.0047	9.10E-16	0.0005	59.2608	mean cIMT
Mean HDL Particle Size	rs57912727	3	C	A	0.1367	-0.0285	0.0053	2.00E-08	0.0002	28.4603	mean cIMT
Mean HDL Particle Size	rs58542926	19	T	C	0.0744	0.0444	0.0070	2.40E-09	0.0004	40.4415	mean cIMT
Mean HDL Particle Size	rs59104589	2	T	C	0.3588	0.0266	0.0038	2.60E-14	0.0004	48.6794	mean cIMT
Mean HDL Particle Size	rs6082	15	G	A	0.0821	0.0433	0.0067	1.70E-10	0.0004	41.8081	mean cIMT
Mean HDL Particle Size	rs60847460	10	T	C	0.1433	-0.0307	0.0052	2.70E-09	0.0003	34.1845	mean cIMT
Mean HDL Particle Size	rs61854123	10	A	G	0.2427	0.0294	0.0043	7.40E-12	0.0004	47.0176	mean cIMT
Mean HDL Particle Size	rs61941676	12	A	C	0.1272	-0.0619	0.0056	6.70E-29	0.0011	121.0670	mean cIMT
Mean HDL Particle Size	rs62433138	7	C	T	0.4291	-0.0347	0.0037	6.10E-21	0.0008	87.6719	mean cIMT
Mean HDL Particle Size	rs635634	9	T	C	0.1835	-0.0331	0.0047	5.10E-13	0.0004	48.6040	mean cIMT
Mean HDL Particle Size	rs6498540	16	G	A	0.3021	-0.0302	0.0040	5.70E-15	0.0005	57.8383	mean cIMT
Mean HDL Particle Size	rs6681348	1	C	A	0.5322	-0.0239	0.0037	1.40E-11	0.0004	42.4428	mean cIMT
Mean HDL Particle Size	rs6694102	1	A	G	0.3209	-0.0332	0.0039	1.30E-17	0.0006	71.6544	mean cIMT
Mean HDL Particle Size	rs676210	2	A	G	0.2058	0.0494	0.0045	1.00E-27	0.0010	119.8289	mean cIMT
Mean HDL Particle Size	rs686030	9	A	C	0.8595	0.0480	0.0053	4.90E-21	0.0007	83.0268	mean cIMT
Mean HDL Particle Size	rs6864091	5	C	T	0.3625	0.0229	0.0038	2.30E-09	0.0003	36.1010	mean cIMT
Mean HDL Particle Size	rs7012814	8	A	G	0.4743	0.0262	0.0037	4.30E-12	0.0004	50.4895	mean cIMT
Mean HDL Particle Size	rs705379	7	A	G	0.4775	-0.0296	0.0037	2.30E-16	0.0006	64.1192	mean cIMT
Mean HDL Particle Size	rs7175905	15	G	A	0.5617	0.0199	0.0037	2.20E-08	0.0003	29.3149	mean cIMT
Mean HDL Particle Size	rs72555385	7	G	A	0.0493	-0.0524	0.0085	1.20E-09	0.0003	37.9816	mean cIMT
Mean HDL Particle Size	rs72836561	17	T	C	0.0315	-0.1182	0.0105	1.40E-31	0.0011	127.1425	mean cIMT
Mean HDL Particle Size	rs72934503	6	G	A	0.4514	0.0255	0.0038	8.80E-12	0.0004	46.2236	mean cIMT
Mean HDL Particle Size	rs7308864	12	G	A	0.5233	0.0254	0.0037	7.90E-13	0.0004	48.4154	mean cIMT
Mean HDL Particle Size	rs737337	19	C	T	0.0768	-0.0471	0.0069	1.20E-11	0.0004	46.8768	mean cIMT
Mean HDL Particle Size	rs75460349	1	C	A	0.0233	-0.0737	0.0123	1.10E-08	0.0003	35.9281	mean cIMT
Mean HDL Particle Size	rs75714888	16	A	C	0.0216	-0.0747	0.0126	5.80E-09	0.0003	35.0986	mean cIMT
Mean HDL Particle Size	rs75911530	16	A	G	0.0329	-0.1262	0.0108	9.40E-34	0.0012	137.7841	mean cIMT
Mean HDL Particle Size	rs7730653	5	T	G	0.5070	0.0200	0.0037	4.60E-08	0.0003	29.6412	mean cIMT

Mean HDL Particle Size	rs7810507	7	A	G	0.2811	-0.0304	0.0041	1.70E-13	0.0005	55.9759	mean cIMT
Mean HDL Particle Size	rs79094524	1	G	GA	0.7654	0.0247	0.0043	1.00E-09	0.0003	32.6420	mean cIMT
Mean HDL Particle Size	rs7924036	10	T	G	0.5042	0.0197	0.0037	2.00E-08	0.0003	28.8683	mean cIMT
Mean HDL Particle Size	rs821840	16	G	A	0.3245	0.1995	0.0039	1.00E-200	0.0223	2621.4216	mean cIMT
Mean HDL Particle Size	rs825510	12	C	A	0.1151	-0.0341	0.0057	1.20E-08	0.0003	35.5579	mean cIMT
Mean HDL Particle Size	rs837500	12	T	C	0.6385	0.0217	0.0038	1.10E-08	0.0003	32.0923	mean cIMT
Mean HDL Particle Size	rs838876	12	G	A	0.6814	-0.0409	0.0040	1.60E-27	0.0009	105.2577	mean cIMT
Mean HDL Particle Size	rs9265113	6	T	C	0.4076	-0.0271	0.0040	1.20E-11	0.0004	46.0642	mean cIMT
Mean HDL Particle Size	rs9429767	1	A	G	0.1975	-0.0262	0.0047	1.80E-08	0.0003	31.6328	mean cIMT
Mean HDL Particle Size	rs998584	6	A	C	0.4823	-0.0343	0.0037	4.20E-21	0.0008	87.5055	mean cIMT
Apolipoprotein A1	rs10184004	2	T	C	0.4061	0.0214	0.0038	5.60E-09	0.0003	30.9579	mean cIMT
Apolipoprotein A1	rs1047891	2	A	C	0.3150	-0.0245	0.0041	4.50E-09	0.0003	36.2454	mean cIMT
Apolipoprotein A1	rs1065853	19	T	G	0.0806	0.0912	0.0070	9.30E-42	0.0015	171.0789	mean cIMT
Apolipoprotein A1	rs1077835	15	G	A	0.2197	0.1199	0.0046	1.70E-157	0.0059	688.3433	mean cIMT
Apolipoprotein A1	rs112001035	17	A	G	0.0597	-0.0527	0.0081	2.80E-10	0.0004	42.0850	mean cIMT
Apolipoprotein A1	rs112495680	6	G	A	0.1334	-0.0357	0.0056	1.30E-10	0.0004	40.8186	mean cIMT
Apolipoprotein A1	rs11671872	19	A	G	0.4125	0.0250	0.0039	1.60E-10	0.0004	41.8469	mean cIMT
Apolipoprotein A1	rs117687565	18	T	C	0.0120	0.1285	0.0180	3.20E-12	0.0004	51.0877	mean cIMT
Apolipoprotein A1	rs11789603	9	T	C	0.1088	0.0591	0.0061	2.10E-21	0.0008	94.4174	mean cIMT
Apolipoprotein A1	rs1260326	2	C	T	0.6040	-0.0389	0.0039	5.70E-23	0.0009	101.2873	mean cIMT
Apolipoprotein A1	rs12904367	15	A	G	0.1413	0.0340	0.0055	6.40E-10	0.0003	37.7592	mean cIMT
Apolipoprotein A1	rs12976739	19	A	G	0.3962	0.0256	0.0039	7.50E-12	0.0004	43.5064	mean cIMT
Apolipoprotein A1	rs13107325	4	T	C	0.0743	-0.0691	0.0072	3.20E-23	0.0008	92.0590	mean cIMT
Apolipoprotein A1	rs1321257	1	A	G	0.6132	0.0472	0.0039	2.80E-35	0.0013	147.9044	mean cIMT
Apolipoprotein A1	rs139915535	8	G	A	0.0180	-0.1191	0.0143	2.10E-18	0.0006	69.8260	mean cIMT
Apolipoprotein A1	rs140168704	10	T	C	0.1535	-0.0275	0.0053	4.60E-08	0.0002	27.3708	mean cIMT
Apolipoprotein A1	rs140584594	1	G	A	0.7301	0.0339	0.0042	7.10E-16	0.0006	64.0838	mean cIMT
Apolipoprotein A1	rs148063610	10	C	CAAATAAAT	0.7634	-0.0349	0.0045	1.00E-14	0.0005	58.9605	mean cIMT

Apolipoprotein A1	rs15285	8	T	C	0.2865	0.0819	0.0042	8.60E-87	0.0033	383.4868	mean cIMT
Apolipoprotein A1	rs17696736	12	G	A	0.4302	-0.0186	0.0038	4.90E-08	0.0002	23.8153	mean cIMT
Apolipoprotein A1	rs1800961	20	T	C	0.0302	-0.1226	0.0110	3.40E-28	0.0011	123.3674	mean cIMT
Apolipoprotein A1	rs193084249	1	G	A	0.0234	-0.0968	0.0128	2.90E-14	0.0005	57.4189	mean cIMT
Apolipoprotein A1	rs2043085	15	C	T	0.6127	-0.1099	0.0039	5.90E-181	0.0069	804.4545	mean cIMT
Apolipoprotein A1	rs2066714	9	C	T	0.1289	0.0367	0.0056	1.00E-11	0.0004	42.6552	mean cIMT
Apolipoprotein A1	rs2176040	2	G	A	0.6467	-0.0294	0.0039	1.60E-15	0.0005	55.5958	mean cIMT
Apolipoprotein A1	rs2236252	20	T	C	0.1670	-0.0308	0.0051	3.70E-10	0.0003	36.9551	mean cIMT
Apolipoprotein A1	rs2298428	22	T	C	0.1826	-0.0407	0.0049	2.00E-17	0.0006	69.2010	mean cIMT
Apolipoprotein A1	rs235314	21	T	C	0.5327	-0.0208	0.0038	4.80E-08	0.0003	30.2199	mean cIMT
Apolipoprotein A1	rs2494748	14	T	C	0.6162	-0.0223	0.0039	1.80E-09	0.0003	32.7211	mean cIMT
Apolipoprotein A1	rs2642438	1	G	A	0.7038	0.0357	0.0041	8.30E-19	0.0006	74.3752	mean cIMT
Apolipoprotein A1	rs267738	1	G	T	0.2196	0.0337	0.0046	1.20E-14	0.0005	54.6394	mean cIMT
Apolipoprotein A1	rs2740488	9	C	A	0.2653	-0.0501	0.0043	1.10E-32	0.0012	136.3459	mean cIMT
Apolipoprotein A1	rs2781752	19	A	G	0.3182	0.0266	0.0041	4.20E-12	0.0004	42.4004	mean cIMT
Apolipoprotein A1	rs2792735	10	A	G	0.7203	-0.0404	0.0042	5.90E-23	0.0008	91.9079	mean cIMT
Apolipoprotein A1	rs2925979	16	C	T	0.6989	0.0300	0.0041	1.40E-14	0.0005	53.2384	mean cIMT
Apolipoprotein A1	rs35135293	2	T	C	0.5167	-0.0286	0.0038	1.50E-14	0.0005	56.7017	mean cIMT
Apolipoprotein A1	rs35184771	11	T	G	0.3528	-0.0366	0.0039	3.10E-20	0.0007	86.1734	mean cIMT
Apolipoprotein A1	rs3735687	7	G	A	0.4228	-0.0231	0.0038	7.10E-09	0.0003	36.2762	mean cIMT
Apolipoprotein A1	rs3764261	16	A	C	0.3244	0.1739	0.0040	1.00E-200	0.0159	1862.5641	mean cIMT
Apolipoprotein A1	rs3768321	1	T	G	0.1965	-0.0407	0.0048	7.00E-19	0.0006	73.0336	mean cIMT
Apolipoprotein A1	rs4239651	20	C	T	0.7942	0.0364	0.0047	6.10E-16	0.0005	60.6364	mean cIMT
Apolipoprotein A1	rs4240624	8	A	G	0.9092	0.1004	0.0066	4.00E-53	0.0020	232.4814	mean cIMT
Apolipoprotein A1	rs4759375	12	T	C	0.0849	0.0402	0.0070	2.20E-08	0.0003	33.3681	mean cIMT
Apolipoprotein A1	rs4969141	17	T	C	0.4895	0.0210	0.0038	2.20E-08	0.0003	30.5821	mean cIMT
Apolipoprotein A1	rs583104	1	T	G	0.7734	-0.0283	0.0045	1.10E-10	0.0003	39.2663	mean cIMT
Apolipoprotein A1	rs60847460	10	T	C	0.1433	-0.0376	0.0054	7.20E-12	0.0004	48.1744	mean cIMT

Apolipoprotein A1	rs61805076	1	C	T	0.3341	-0.0261	0.0040	2.60E-11	0.0004	42.6073	mean cIMT
Apolipoprotein A1	rs61941676	12	A	C	0.1272	-0.0554	0.0058	7.40E-22	0.0008	90.7944	mean cIMT
Apolipoprotein A1	rs62101704	18	A	G	0.0137	-0.0907	0.0166	1.20E-08	0.0003	29.9615	mean cIMT
Apolipoprotein A1	rs638714	1	T	G	0.3459	-0.0458	0.0040	2.10E-31	0.0011	131.8333	mean cIMT
Apolipoprotein A1	rs6606717	12	C	A	0.5232	0.0305	0.0038	2.70E-16	0.0006	65.3691	mean cIMT
Apolipoprotein A1	rs676210	2	A	G	0.2058	0.0564	0.0047	8.20E-33	0.0013	146.1828	mean cIMT
Apolipoprotein A1	rs686030	9	A	C	0.8595	0.0440	0.0054	2.00E-16	0.0006	65.2247	mean cIMT
Apolipoprotein A1	rs72836561	17	T	C	0.0315	-0.1276	0.0108	1.50E-33	0.0012	138.8908	mean cIMT
Apolipoprotein A1	rs737338	19	T	C	0.0352	-0.1182	0.0103	1.90E-32	0.0012	132.6944	mean cIMT
Apolipoprotein A1	rs75911530	16	A	G	0.0329	-0.1394	0.0111	5.10E-38	0.0014	157.0862	mean cIMT
Apolipoprotein A1	rs77960347	18	G	A	0.0132	0.3174	0.0165	8.80E-85	0.0032	369.9055	mean cIMT
Apolipoprotein A1	rs7810507	7	A	G	0.2811	-0.0233	0.0042	2.10E-08	0.0003	30.6874	mean cIMT
Apolipoprotein A1	rs7956099	12	C	T	0.4750	-0.0224	0.0038	1.40E-09	0.0003	34.7393	mean cIMT
Apolipoprotein A1	rs8058512	16	T	C	0.7240	-0.0239	0.0042	1.90E-08	0.0003	31.8810	mean cIMT
Apolipoprotein A1	rs838876	12	G	A	0.6814	-0.0380	0.0041	2.30E-22	0.0007	85.5759	mean cIMT
Apolipoprotein A1	rs9304381	18	T	C	0.8184	0.0937	0.0049	2.70E-83	0.0032	364.9380	mean cIMT
Apolipoprotein A1	rs9471972	6	A	G	0.5362	0.0228	0.0038	2.80E-09	0.0003	36.3604	mean cIMT
Apolipoprotein A1	rs9491697	6	G	A	0.4636	-0.0210	0.0038	3.20E-08	0.0003	30.4624	mean cIMT
Apolipoprotein A1	rs967645	17	T	C	0.5165	-0.0275	0.0038	2.20E-12	0.0005	52.9478	mean cIMT
Apolipoprotein A1	rs9884390	4	C	T	0.2340	0.0249	0.0045	6.70E-09	0.0003	30.4906	mean cIMT

Chr denotes chromosome; EAF, effective allele frequency; SE, standard error; $R^2 = 2 \times MAF \times (1-MAF) \times beta^2$; $F = R^2 \times (N-2)/(1-R^2)$.

Table S4. Causal associations between (apo)lipoprotein profile and mean cIMT from two-sample MR analysis.

Exposure	Outcome	Methods	NSNPs	OR (95%CI)	*pval*
Total Concentration of Lipoprotein Particles	mean cIMT	IVW	60	0.99 (0.98, 1.00)	0.094
Total Concentration of Lipoprotein Particles	mean cIMT	MR Egger	60	0.99 (0.97, 1.00)	0.144
Total Concentration of Lipoprotein Particles	mean cIMT	Weighted median	60	0.99 (0.97, 1.00)	0.070
Total Concentration of Lipoprotein Particles	mean cIMT	leave one out	60	0.99 (0.98, 1.00)	0.135
Total Concentration of Lipoprotein Particles	mean cIMT	Radial IVW	55	0.99 (0.98, 0.999)	0.037
Total Concentration of HDL Particles	mean cIMT	IVW	59	0.99 (0.98, 1.00)	0.094
Total Concentration of HDL Particles	mean cIMT	MR Egger	59	0.99 (0.97, 1.01)	0.235
Total Concentration of HDL Particles	mean cIMT	Weighted median	59	0.99 (0.97, 1.00)	0.096
Total Concentration of HDL Particles	mean cIMT	leave one out	59	0.99 (0.98, 1.00)	0.105
Total Concentration of HDL Particles	mean cIMT	Radial IVW	53	0.99 (0.98, 0.999)	0.033
Total Concentration of Medium HDL Particles	mean cIMT	IVW	66	0.98 (0.97, 0.99)	0.003
Total Concentration of Medium HDL Particles	mean cIMT	MR Egger	66	0.99 (0.97, 1.01)	0.196
Total Concentration of Medium HDL Particles	mean cIMT	Weighted median	66	0.99 (0.97, 1.00)	0.096
Total Concentration of Medium HDL Particles	mean cIMT	leave one out	66	0.98 (0.97, 0.99)	0.003
Total Concentration of Medium HDL Particles	mean cIMT	Radial IVW	58	0.99 (0.98, 0.999)	0.004
Total Lipids in HDL	mean cIMT	IVW	79	0.99 (0.98, 0.999)	0.049
Total Lipids in HDL	mean cIMT	MR Egger	79	0.999 (0.99, 1.02)	0.892
Total Lipids in HDL	mean cIMT	Weighted median	79	0.99 (0.98, 1.01)	0.282
Total Lipids in HDL	mean cIMT	leave one out	79	0.99 (0.98, 0.999)	0.049
Total Lipids in HDL	mean cIMT	Radial IVW	69	0.99 (0.99, 0.999)	0.033
Total Lipids in Medium HDL	mean cIMT	IVW	63	0.98 (0.97, 0.99)	0.003
Total Lipids in Medium HDL	mean cIMT	MR Egger	63	0.98 (0.96, 1.00)	0.064
Total Lipids in Medium HDL	mean cIMT	Weighted median	63	0.99 (0.97, 1.00)	0.086
Total Lipids in Medium HDL	mean cIMT	leave one out	63	0.98 (0.97, 0.99)	0.003
Total Lipids in Medium HDL	mean cIMT	Radial IVW	56	0.99 (0.98, 0.99)	0.001
Total Lipids in Large HDL	mean cIMT	IVW	94	0.999 (0.99, 1.00)	0.302

Total Lipids in Large HDL	mean cIMT	MR Egger	94	0.999 (0.99, 1.02)	0.547
Total Lipids in Large HDL	mean cIMT	Weighted median	94	0.999 (0.99, 1.01)	0.553
Total Lipids in Large HDL	mean cIMT	leave one out	94	0.999 (0.99, 1.00)	0.302
Total Lipids in Large HDL	mean cIMT	Radial IVW	83	0.999 (0.99, 1.00)	0.162
HDL Cholesterol	mean cIMT	IVW	87	0.99 (0.85, 1.15)	0.077
HDL Cholesterol	mean cIMT	MR Egger	87	0.999 (0.98, 1.01)	0.688
HDL Cholesterol	mean cIMT	Weighted median	87	0.99 (0.98, 1.00)	0.168
HDL Cholesterol	mean cIMT	leave one out	87	0.99 (0.98, 1.00)	0.077
HDL Cholesterol	mean cIMT	Radial IVW	74	0.99 (0.99, 0.999)	0.008
Total Cholesterol in Medium HDL	mean cIMT	IVW	78	0.98 (0.97, 0.99)	0.001
Total Cholesterol in Medium HDL	mean cIMT	MR Egger	78	0.99 (0.98, 1.01)	0.382
Total Cholesterol in Medium HDL	mean cIMT	Weighted median	78	0.99 (0.98, 1.00)	0.100
Total Cholesterol in Medium HDL	mean cIMT	leave one out	78	0.98 (0.97, 0.99)	0.001
Total Cholesterol in Medium HDL	mean cIMT	Radial IVW	68	0.99 (0.98, 0.99)	2.73E-04
Free Cholesterol in Medium HDL	mean cIMT	IVW	75	0.99 (0.98, 1.00)	0.071
Free Cholesterol in Medium HDL	mean cIMT	MR Egger	75	0.999 (0.98, 1.02)	0.994
Free Cholesterol in Medium HDL	mean cIMT	Weighted median	75	0.99 (0.98, 1.00)	0.238
Free Cholesterol in Medium HDL	mean cIMT	leave one out	75	0.99 (0.98, 1.00)	0.071
Free Cholesterol in Medium HDL	mean cIMT	Radial IVW	65	0.99 (0.99, 1.00)	0.061
Cholesteryl Esters in HDL	mean cIMT	IVW	89	0.99 (0.98, 0.999)	0.004
Cholesteryl Esters in HDL	mean cIMT	MR Egger	89	0.999 (0.98, 1.01)	0.517
Cholesteryl Esters in HDL	mean cIMT	Weighted median	89	0.99 (0.98, 1.00)	0.183
Cholesteryl Esters in HDL	mean cIMT	leave one out	89	0.99 (0.98, 0.999)	0.004
Cholesteryl Esters in HDL	mean cIMT	Radial IVW	77	0.99 (0.98, 0.99)	3.44E-04
Cholesteryl Esters in Medium HDL	mean cIMT	IVW	77	0.98 (0.97, 0.99)	0.002
Cholesteryl Esters in Medium HDL	mean cIMT	MR Egger	77	0.99 (0.97, 1.01)	0.311
Cholesteryl Esters in Medium HDL	mean cIMT	Weighted median	77	0.99 (0.97, 1.00)	0.135
Cholesteryl Esters in Medium HDL	mean cIMT	leave one out	77	0.98 (0.97, 0.99)	0.002

Cholesteryl Esters in Medium HDL	mean cIMT	Radial IVW	65	0.99 (0.98, 0.999)	0.005
Cholesteryl Esters in Large HDL	mean cIMT	IVW	93	0.99 (0.99, 1.00)	0.218
Cholesteryl Esters in Large HDL	mean cIMT	MR Egger	93	0.999 (0.99, 1.02)	0.552
Cholesteryl Esters in Large HDL	mean cIMT	Weighted median	93	0.999 (0.98, 1.01)	0.372
Cholesteryl Esters in Large HDL	mean cIMT	leave one out	93	0.99 (0.99, 1.00)	0.218
Cholesteryl Esters in Large HDL	mean cIMT	Radial IVW	81	0.999 (0.99, 1.00)	0.180
Phospholipids in HDL	mean cIMT	IVW	66	0.99 (0.98, 1.00)	0.064
Phospholipids in HDL	mean cIMT	MR Egger	66	0.99 (0.97, 1.01)	0.310
Phospholipids in HDL	mean cIMT	Weighted median	66	0.99 (0.98, 1.01)	0.395
Phospholipids in HDL	mean cIMT	leave one out	66	0.99 (0.98, 1.00)	0.064
Phospholipids in HDL	mean cIMT	Radial IVW	60	0.99 (0.99, 0.999)	0.048
Phospholipids in Large HDL	mean cIMT	IVW	82	0.999 (0.99, 1.00)	0.240
Phospholipids in Large HDL	mean cIMT	MR Egger	82	0.999 (0.99, 1.01)	0.769
Phospholipids in Large HDL	mean cIMT	Weighted median	82	0.999 (0.99, 1.01)	0.487
Phospholipids in Large HDL	mean cIMT	leave one out	82	0.999 (0.99, 1.00)	0.240
Phospholipids in Large HDL	mean cIMT	Radial IVW	73	0.999 (0.99, 1.00)	0.188
Mean HDL Particle Size	mean cIMT	IVW	88	0.999 (0.99, 1.01)	0.455
Mean HDL Particle Size	mean cIMT	MR Egger	88	0.999 (0.99, 1.01)	0.805
Mean HDL Particle Size	mean cIMT	Weighted median	88	0.999 (0.99, 1.01)	0.980
Mean HDL Particle Size	mean cIMT	leave one out	88	0.999 (0.99, 1.01)	0.455
Mean HDL Particle Size	mean cIMT	Radial IVW	79	0.999 (0.99, 1.00)	0.343
Apolipoprotein A1	mean cIMT	IVW	66	0.99 (0.90, 1.09)	0.051
Apolipoprotein A1	mean cIMT	MR Egger	66	0.99 (0.97, 1.01)	0.317
Apolipoprotein A1	mean cIMT	Weighted median	66	0.99 (0.98, 1.00)	0.074
Apolipoprotein A1	mean cIMT	leave one out	66	0.99 (0.98, 1.00)	0.051
Apolipoprotein A1	mean cIMT	Radial IVW	59	0.99 (0.98, 0.999)	0.012

IVW denotes inverse variance weighted method; $P < 0.05$ was considered as nominally significant.

Table S5. Cochran Q and MR Egger pleiotropy tests of MR analysis between (apo)lipoprotein profile and mean cIMT.

Exposure	Outcome	IVW Cochran Q test		MR-Egger		
		**Q-statistic**	**Q_p**	**intercept**	**SE**	***pval***
Total Concentration of Lipoprotein Particles	mean cIMT	74.279	0.087	0.00039	0.00048	0.424
Total Concentration of HDL Particles	mean cIMT	62.051	0.334	0.00016	0.00043	0.717
Total Concentration of Medium HDL Particles	mean cIMT	139.89	2.08E-07	-0.0003	0.00052	0.574
Total Lipids in HDL	mean cIMT	120.659	0.001	-0.00906	0.00461	0.049
Total Lipids in Medium HDL	mean cIMT	129.719	1.07E-06	0.0001	0.00057	0.866
Total Lipids in Large HDL	mean cIMT	147.116	2.99E-04	-0.00058	0.00034	0.091
HDL Cholesterol	mean cIMT	146.745	4.92E-05	-0.00035	0.0004	0.377
Total Cholesterol in Medium HDL	mean cIMT	126.195	3.48E-04	-0.00054	0.00042	0.199
Free Cholesterol in Medium HDL	mean cIMT	117.127	0.001	-0.00057	0.00041	0.171
Cholesteryl Esters in HDL	mean cIMT	126.072	0.005	-0.00051	0.00036	0.153
Cholesteryl Esters in Medium HDL	mean cIMT	132.55	6.41E-05	-0.00039	0.0005	0.437
Cholesteryl Esters in Large HDL	mean cIMT	166.928	2.93E-06	-0.00068	0.00037	0.071
Phospholipids in HDL	mean cIMT	142.688	9.46E-08	-0.00007	0.00052	0.894
Phospholipids in Large HDL	mean cIMT	118.351	0.004	-0.0005	0.00035	0.159
Mean HDL Particle Size	mean cIMT	133.53	0.001	-0.00011	0.00036	0.756
Apolipoprotein A1	mean cIMT	136.038	6.06E-07	-0.00011	0.00052	0.834

SE denotes standard error.

Table S6. Genetic instruments for HDL subclasses to CAD and IS in two-sample MR analysis.

Exposure	SNP	Chr	Effect Allele	Alternate Allele	EAF	Beta	SE	*pval*	r2	F statistic	Outcome
Cholesteryl Esters in HDL	rs12295878	11	T	C	0.140	0.033	0.005	2.10E-09	3.34E-04	38.478	CAD
Cholesteryl Esters in HDL	rs12453682	17	T	C	0.694	0.021	0.004	2.20E-08	2.38E-04	27.435	CAD
Cholesteryl Esters in HDL	rs12533197	7	G	T	0.439	-0.022	0.004	4.30E-08	2.87E-04	33.084	CAD
Cholesteryl Esters in HDL	rs12608026	18	G	T	0.042	0.070	0.009	1.80E-14	0.001	57.590	CAD
Cholesteryl Esters in HDL	rs12721046	19	A	G	0.158	-0.037	0.005	1.00E-13	4.47E-04	51.474	CAD
Cholesteryl Esters in HDL	rs13107325	4	T	C	0.074	-0.075	0.007	2.60E-27	0.001	111.482	CAD
Cholesteryl Esters in HDL	rs13389219	2	T	C	0.393	0.036	0.004	4.20E-22	0.001	88.731	CAD
Cholesteryl Esters in HDL	rs1358980	6	T	C	0.483	-0.034	0.004	2.70E-19	0.001	80.640	CAD
Cholesteryl Esters in HDL	rs142110991	6	C	T	0.077	-0.047	0.009	2.30E-08	2.47E-04	28.413	CAD
Cholesteryl Esters in HDL	rs144018203	11	C	G	0.011	-0.192	0.019	1.30E-24	0.001	104.363	CAD
Cholesteryl Esters in HDL	rs145391587	8	C	A	0.100	0.165	0.006	3.00E-163	0.006	715.192	CAD
Cholesteryl Esters in HDL	rs147233090	15	T	C	0.025	-0.085	0.012	5.00E-13	4.33E-04	49.811	CAD
Cholesteryl Esters in HDL	rs150224153	20	T	C	0.029	-0.092	0.011	4.00E-16	0.001	66.924	CAD
Cholesteryl Esters in HDL	rs1561748	8	C	G	0.267	0.062	0.005	3.20E-43	0.002	190.335	CAD
Cholesteryl Esters in HDL	rs1601935	15	T	G	0.655	-0.083	0.004	4.50E-102	0.004	446.724	CAD
Cholesteryl Esters in HDL	rs174578	11	A	T	0.351	-0.062	0.004	5.50E-62	0.002	255.697	CAD
Cholesteryl Esters in HDL	rs1761457	19	G	A	0.327	0.030	0.004	3.30E-15	4.87E-04	56.023	CAD
Cholesteryl Esters in HDL	rs17696736	12	G	A	0.430	-0.022	0.004	1.20E-10	2.90E-04	33.370	CAD
Cholesteryl Esters in HDL	rs191555775	6	T	A	0.106	-0.056	0.006	5.90E-22	0.001	85.312	CAD
Cholesteryl Esters in HDL	rs2066714	9	C	T	0.129	0.032	0.006	4.50E-10	3.01E-04	34.704	CAD
Cholesteryl Esters in HDL	rs2070895	15	A	G	0.219	0.090	0.004	2.90E-94	0.003	400.942	CAD
Cholesteryl Esters in HDL	rs2229357	12	A	G	0.241	0.031	0.004	2.50E-13	4.36E-04	50.231	CAD
Cholesteryl Esters in HDL	rs2245221	8	A	G	0.559	0.030	0.004	7.00E-15	0.001	65.401	CAD
Cholesteryl Esters in HDL	rs2297402	9	T	C	0.024	-0.064	0.013	3.50E-08	2.23E-04	25.642	CAD
Cholesteryl Esters in HDL	rs2298428	22	T	C	0.183	-0.036	0.005	1.00E-14	4.80E-04	55.316	CAD
Cholesteryl Esters in HDL	rs2302263	11	T	C	0.088	-0.037	0.007	1.30E-08	2.79E-04	32.072	CAD

Cholesteryl Esters in HDL	rs2307111	5	C	T	0.397	0.024	0.004	6.30E-10	3.39E-04	39.069	CAD
Cholesteryl Esters in HDL	rs2494748	14	T	C	0.616	-0.021	0.004	6.20E-09	2.60E-04	29.982	CAD
Cholesteryl Esters in HDL	rs2642438	1	G	A	0.704	0.035	0.004	6.80E-19	0.001	75.104	CAD
Cholesteryl Esters in HDL	rs267738	1	G	T	0.220	0.028	0.004	3.30E-11	3.51E-04	40.415	CAD
Cholesteryl Esters in HDL	rs2740488	9	C	A	0.265	-0.043	0.004	1.50E-25	0.001	103.309	CAD
Cholesteryl Esters in HDL	rs2792735	10	A	G	0.720	-0.039	0.004	3.70E-23	0.001	90.926	CAD
Cholesteryl Esters in HDL	rs28818616	3	C	T	0.350	-0.020	0.004	3.50E-08	2.34E-04	26.915	CAD
Cholesteryl Esters in HDL	rs2925979	16	C	T	0.699	0.039	0.004	2.00E-24	0.001	96.139	CAD
Cholesteryl Esters in HDL	rs2943650	2	T	C	0.645	-0.042	0.004	1.50E-29	0.001	116.546	CAD
Cholesteryl Esters in HDL	rs2978615	19	T	C	0.490	-0.020	0.004	3.00E-08	2.58E-04	29.673	CAD
Cholesteryl Esters in HDL	rs35184771	11	T	G	0.353	-0.039	0.004	2.40E-23	0.001	99.386	CAD
Cholesteryl Esters in HDL	rs35493868	7	G	C	0.204	0.036	0.005	2.20E-15	0.001	60.431	CAD
Cholesteryl Esters in HDL	rs35633876	2	T	G	0.482	-0.023	0.004	6.30E-10	3.17E-04	36.511	CAD
Cholesteryl Esters in HDL	rs3735687	7	G	A	0.423	-0.023	0.004	6.80E-09	3.12E-04	35.929	CAD
Cholesteryl Esters in HDL	rs3764261	16	A	C	0.324	0.210	0.004	1.00E-200	0.024	2825.378	CAD
Cholesteryl Esters in HDL	rs3768321	1	T	G	0.197	-0.044	0.005	5.30E-23	0.001	89.345	CAD
Cholesteryl Esters in HDL	rs4078216	12	A	G	0.241	0.026	0.004	2.10E-08	3.14E-04	36.146	CAD
Cholesteryl Esters in HDL	rs4240624	8	A	G	0.909	0.094	0.006	1.50E-49	0.002	214.253	CAD
Cholesteryl Esters in HDL	rs4330777	16	A	G	0.476	-0.020	0.004	1.10E-08	2.57E-04	29.535	CAD
Cholesteryl Esters in HDL	rs4846921	1	A	G	0.613	0.050	0.004	4.60E-41	0.001	169.740	CAD
Cholesteryl Esters in HDL	rs4922787	11	T	G	0.762	0.027	0.004	2.30E-10	3.33E-04	38.381	CAD
Cholesteryl Esters in HDL	rs4969141	17	T	C	0.490	0.024	0.004	1.40E-10	3.56E-04	41.004	CAD
Cholesteryl Esters in HDL	rs4986970	16	T	A	0.034	-0.092	0.010	2.60E-20	0.001	81.412	CAD
Cholesteryl Esters in HDL	rs5167	19	G	T	0.352	0.044	0.004	4.40E-31	0.001	130.228	CAD
Cholesteryl Esters in HDL	rs559355	11	T	A	0.158	-0.051	0.005	2.70E-23	0.001	98.999	CAD
Cholesteryl Esters in HDL	rs59104589	2	T	C	0.359	0.020	0.004	1.00E-08	2.44E-04	28.038	CAD
Cholesteryl Esters in HDL	rs59299606	16	A	G	0.249	-0.028	0.005	1.30E-09	2.99E-04	34.429	CAD
Cholesteryl Esters in HDL	rs6018652	20	A	G	0.793	0.035	0.005	9.50E-16	0.001	58.683	CAD

Cholesteryl Esters in HDL	rs6073958	20	C	T	0.199	-0.067	0.005	6.90E-47	0.002	209.567	CAD
Cholesteryl Esters in HDL	rs60847460	10	T	C	0.143	-0.036	0.005	7.40E-12	4.08E-04	47.003	CAD
Cholesteryl Esters in HDL	rs61805076	1	C	T	0.334	-0.030	0.004	1.90E-14	4.98E-04	57.346	CAD
Cholesteryl Esters in HDL	rs62101705	18	T	C	0.014	-0.089	0.016	8.40E-09	2.60E-04	29.925	CAD
Cholesteryl Esters in HDL	rs676210	2	A	G	0.206	0.072	0.005	1.70E-55	0.002	247.975	CAD
Cholesteryl Esters in HDL	rs688671	18	G	A	0.267	-0.025	0.004	4.30E-09	3.01E-04	34.648	CAD
Cholesteryl Esters in HDL	rs71336055	3	T	C	0.120	0.036	0.006	3.90E-10	3.38E-04	38.854	CAD
Cholesteryl Esters in HDL	rs7134375	12	A	C	0.430	0.024	0.004	5.60E-11	3.57E-04	41.149	CAD
Cholesteryl Esters in HDL	rs7136506	12	C	T	0.216	-0.040	0.005	9.00E-19	0.001	75.433	CAD
Cholesteryl Esters in HDL	rs71562509	6	T	G	0.593	0.021	0.004	3.30E-08	2.61E-04	30.083	CAD
Cholesteryl Esters in HDL	rs7241918	18	T	G	0.823	0.074	0.005	1.70E-54	0.002	233.708	CAD
Cholesteryl Esters in HDL	rs72836561	17	T	C	0.031	-0.159	0.011	9.10E-54	0.002	224.865	CAD
Cholesteryl Esters in HDL	rs7308864	12	G	A	0.523	0.025	0.004	3.80E-12	3.96E-04	45.609	CAD
Cholesteryl Esters in HDL	rs737337	19	C	T	0.077	-0.059	0.007	1.20E-17	0.001	71.557	CAD
Cholesteryl Esters in HDL	rs75911530	16	A	G	0.033	-0.162	0.011	8.10E-52	0.002	221.073	CAD
Cholesteryl Esters in HDL	rs77960347	18	G	A	0.013	0.248	0.016	4.30E-55	0.002	234.122	CAD
Cholesteryl Esters in HDL	rs78058190	2	A	G	0.050	-0.065	0.010	9.00E-11	3.95E-04	45.438	CAD
Cholesteryl Esters in HDL	rs7810507	7	A	G	0.281	-0.032	0.004	1.50E-14	0.001	58.505	CAD
Cholesteryl Esters in HDL	rs7845090	8	A	G	0.709	0.024	0.004	1.40E-08	2.89E-04	33.242	CAD
Cholesteryl Esters in HDL	rs8058512	16	T	C	0.724	-0.027	0.004	1.90E-10	3.62E-04	41.718	CAD
Cholesteryl Esters in HDL	rs904770	16	C	T	0.889	-0.037	0.006	2.50E-10	3.46E-04	39.807	CAD
Cholesteryl Esters in HDL	rs921919	12	A	G	0.670	-0.042	0.004	8.90E-28	0.001	106.298	CAD
Cholesteryl Esters in HDL	rs9265113	6	T	C	0.408	-0.029	0.004	2.90E-13	4.47E-04	51.497	CAD
Cholesteryl Esters in HDL	rs9491697	6	G	A	0.464	-0.028	0.004	7.10E-15	0.001	57.988	CAD
Cholesteryl Esters in HDL	rs964184	11	C	G	0.867	0.097	0.005	4.50E-74	0.003	316.212	CAD
Cholesteryl Esters in HDL	rs10184004	2	T	C	0.406	0.025	0.004	1.80E-12	3.92E-04	45.184	CAD
Cholesteryl Esters in HDL	rs11045171	12	G	A	0.197	0.026	0.005	9.40E-09	2.63E-04	30.286	CAD
Cholesteryl Esters in HDL	rs11057692	12	G	A	0.236	-0.023	0.004	1.80E-08	2.40E-04	27.661	CAD

Cholesteryl Esters in HDL	rs112001035	17	A	G	0.060	-0.051	0.008	5.70E-10	3.54E-04	40.738	CAD
Cholesteryl Esters in HDL	rs116843064	19	A	G	0.020	0.167	0.013	7.50E-38	0.001	158.095	CAD
Cholesteryl Esters in HDL	rs11751347	6	T	C	0.102	-0.037	0.006	4.30E-10	3.21E-04	36.997	CAD
Cholesteryl Esters in HDL	rs11789603	9	T	C	0.109	0.055	0.006	3.50E-19	0.001	84.998	CAD
Cholesteryl Esters in HDL	rs12295878	11	T	C	0.140	0.031	0.005	2.00E-08	2.92E-04	33.624	CAD
Cholesteryl Esters in HDL	rs12608026	18	G	T	0.042	0.087	0.009	8.50E-22	0.001	88.497	CAD
Cholesteryl Esters in HDL	rs12721046	19	A	G	0.158	-0.050	0.005	4.20E-23	0.001	95.833	CAD
Cholesteryl Esters in HDL	rs13107325	4	T	C	0.074	-0.071	0.007	1.60E-25	0.001	101.711	CAD
Cholesteryl Esters in HDL	rs1358980	6	T	C	0.483	-0.026	0.004	5.70E-12	4.26E-04	49.027	CAD
Cholesteryl Esters in HDL	rs139915535	8	G	A	0.018	-0.127	0.014	2.10E-21	0.001	82.312	CAD
Cholesteryl Esters in Medium HDL	rs141368429	11	T	C	0.056	-0.050	0.009	6.40E-09	2.94E-04	33.868	CAD
Cholesteryl Esters in Medium HDL	rs147627829	6	A	G	0.044	-0.058	0.009	2.30E-10	3.55E-04	40.809	CAD
Cholesteryl Esters in Medium HDL	rs150224153	20	T	C	0.029	-0.083	0.011	5.30E-13	4.67E-04	53.765	CAD
Cholesteryl Esters in Medium HDL	rs15285	8	T	C	0.287	0.086	0.004	7.60E-100	0.004	440.342	CAD
Cholesteryl Esters in Medium HDL	rs1616661	19	A	T	0.318	0.024	0.004	1.80E-10	3.03E-04	34.828	CAD
Cholesteryl Esters in Medium HDL	rs174574	11	C	A	0.649	0.054	0.004	4.20E-47	0.002	192.551	CAD
Cholesteryl Esters in Medium HDL	rs17696736	12	G	A	0.430	-0.019	0.004	9.10E-09	2.29E-04	26.370	CAD
Cholesteryl Esters in Medium HDL	rs193084249	1	G	A	0.023	-0.096	0.013	2.10E-14	0.001	58.745	CAD
Cholesteryl Esters in Medium HDL	rs2043085	15	C	T	0.613	-0.125	0.004	1.00E-200	0.009	1079.178	CAD
Cholesteryl Esters in Medium HDL	rs2066714	9	C	T	0.129	0.034	0.006	1.80E-10	3.30E-04	38.009	CAD
Cholesteryl Esters in Medium HDL	rs2070895	15	A	G	0.219	0.139	0.004	1.00E-200	0.008	958.761	CAD
Cholesteryl Esters in Medium HDL	rs2176040	2	G	A	0.647	-0.033	0.004	4.70E-19	0.001	70.769	CAD
Cholesteryl Esters in Medium HDL	rs2229357	12	A	G	0.241	0.024	0.004	9.80E-09	2.67E-04	30.705	CAD
Cholesteryl Esters in Medium HDL	rs2236252	20	T	C	0.167	-0.028	0.005	5.90E-09	2.78E-04	32.010	CAD
Cholesteryl Esters in Medium HDL	rs2243616	12	T	G	0.661	-0.022	0.004	2.30E-08	2.82E-04	32.436	CAD
Cholesteryl Esters in Medium HDL	rs2245221	8	A	G	0.559	0.034	0.004	1.50E-18	0.001	80.691	CAD
Cholesteryl Esters in Medium HDL	rs2298428	22	T	C	0.183	-0.039	0.005	9.00E-17	0.001	65.698	CAD
Cholesteryl Esters in Medium HDL	rs2642438	1	G	A	0.704	0.036	0.004	4.20E-20	0.001	78.174	CAD

Cholesteryl Esters in Medium HDL	rs267738	1	G	T	0.220	0.029	0.004	9.00E-12	3.73E-04	42.915	CAD
Cholesteryl Esters in Medium HDL	rs2740488	9	C	A	0.265	-0.046	0.004	1.20E-28	0.001	117.501	CAD
Cholesteryl Esters in Medium HDL	rs2792735	10	A	G	0.720	-0.041	0.004	9.30E-25	0.001	100.013	CAD
Cholesteryl Esters in Medium HDL	rs28818616	3	C	T	0.350	-0.021	0.004	3.00E-08	2.55E-04	29.364	CAD
Cholesteryl Esters in Medium HDL	rs2925979	16	C	T	0.699	0.032	0.004	3.20E-17	0.001	64.792	CAD
Cholesteryl Esters in Medium HDL	rs2978615	19	T	C	0.490	-0.023	0.004	9.80E-10	3.18E-04	36.607	CAD
Cholesteryl Esters in Medium HDL	rs34663616	15	A	C	0.138	0.042	0.005	7.60E-15	4.97E-04	57.234	CAD
Cholesteryl Esters in Medium HDL	rs34707604	4	C	T	0.259	0.026	0.004	2.30E-10	3.04E-04	34.981	CAD
Cholesteryl Esters in Medium HDL	rs34955778	16	C	T	0.420	-0.022	0.004	4.60E-09	2.89E-04	33.288	CAD
Cholesteryl Esters in Medium HDL	rs35184771	11	T	G	0.353	-0.037	0.004	9.40E-22	0.001	92.169	CAD
Cholesteryl Esters in Medium HDL	rs36057735	6	G	C	0.199	-0.035	0.005	6.20E-14	4.79E-04	55.115	CAD
Cholesteryl Esters in Medium HDL	rs3735687	7	G	A	0.423	-0.027	0.004	6.30E-12	4.30E-04	49.473	CAD
Cholesteryl Esters in Medium HDL	rs3764261	16	A	C	0.324	0.190	0.004	1.00E-200	0.020	2312.340	CAD
Cholesteryl Esters in Medium HDL	rs3768321	1	T	G	0.197	-0.039	0.005	1.10E-17	0.001	68.030	CAD
Cholesteryl Esters in Medium HDL	rs41272663	2	A	C	0.264	0.024	0.004	6.50E-09	2.94E-04	33.795	CAD
Cholesteryl Esters in Medium HDL	rs4240624	8	A	G	0.909	0.092	0.006	1.10E-46	0.002	204.335	CAD
Cholesteryl Esters in Medium HDL	rs4330777	16	A	G	0.476	-0.019	0.004	4.60E-08	2.32E-04	26.760	CAD
Cholesteryl Esters in Medium HDL	rs4759375	12	T	C	0.085	0.039	0.007	4.70E-08	2.77E-04	31.888	CAD
Cholesteryl Esters in Medium HDL	rs4846921	1	A	G	0.613	0.049	0.004	2.50E-40	0.001	168.442	CAD
Cholesteryl Esters in Medium HDL	rs4986970	16	T	A	0.034	-0.077	0.010	8.50E-15	4.91E-04	56.530	CAD
Cholesteryl Esters in Medium HDL	rs5167	19	G	T	0.352	0.050	0.004	2.20E-38	0.001	164.034	CAD
Cholesteryl Esters in Medium HDL	rs55779455	16	C	G	0.020	0.070	0.014	4.00E-08	2.35E-04	27.063	CAD
Cholesteryl Esters in Medium HDL	rs559355	11	T	A	0.158	-0.060	0.005	6.10E-32	0.001	137.701	CAD
Cholesteryl Esters in Medium HDL	rs59347135	8	G	C	0.046	-0.068	0.009	6.10E-14	4.77E-04	54.867	CAD
Cholesteryl Esters in Medium HDL	rs599839	1	A	G	0.772	-0.027	0.004	3.60E-10	3.25E-04	37.417	CAD
Cholesteryl Esters in Medium HDL	rs6018652	20	A	G	0.793	0.037	0.005	3.60E-17	0.001	65.852	CAD
Cholesteryl Esters in Medium HDL	rs6073958	20	C	T	0.199	-0.059	0.005	1.10E-36	0.001	163.222	CAD
Cholesteryl Esters in Medium HDL	rs60847460	10	T	C	0.143	-0.038	0.005	1.50E-12	4.37E-04	50.264	CAD

Cholesteryl Esters in Medium HDL	rs61805076	1	C	T	0.334	-0.029	0.004	4.90E-14	4.72E-04	54.324	CAD
Cholesteryl Esters in Medium HDL	rs61941676	12	A	C	0.127	-0.060	0.006	1.20E-26	0.001	110.867	CAD
Cholesteryl Esters in Medium HDL	rs62101704	18	A	G	0.014	-0.089	0.016	7.00E-09	2.60E-04	29.937	CAD
Cholesteryl Esters in Medium HDL	rs625145	11	T	A	0.188	0.063	0.005	2.60E-43	0.002	177.631	CAD
Cholesteryl Esters in Medium HDL	rs638714	1	T	G	0.346	-0.038	0.004	4.00E-23	0.001	95.329	CAD
Cholesteryl Esters in Medium HDL	rs6509173	19	G	A	0.794	-0.030	0.005	2.00E-10	3.67E-04	42.206	CAD
Cholesteryl Esters in Medium HDL	rs6589565	11	G	A	0.932	0.051	0.007	4.20E-13	4.07E-04	46.905	CAD
Cholesteryl Esters in Medium HDL	rs676210	2	A	G	0.206	0.059	0.005	5.50E-38	0.001	167.609	CAD
Cholesteryl Esters in Medium HDL	rs686030	9	A	C	0.860	0.046	0.005	1.80E-18	0.001	73.557	CAD
Cholesteryl Esters in Medium HDL	rs7241918	18	T	G	0.823	0.090	0.005	6.10E-79	0.003	342.692	CAD
Cholesteryl Esters in Medium HDL	rs72836561	17	T	C	0.031	-0.135	0.011	5.50E-39	0.001	161.729	CAD
Cholesteryl Esters in Medium HDL	rs7308864	12	G	A	0.523	0.031	0.004	1.30E-17	0.001	70.373	CAD
Cholesteryl Esters in Medium HDL	rs73632745	11	T	C	0.074	-0.045	0.007	1.10E-09	3.44E-04	39.587	CAD
Cholesteryl Esters in Medium HDL	rs737337	19	C	T	0.077	-0.068	0.007	5.60E-23	0.001	96.746	CAD
Cholesteryl Esters in Medium HDL	rs7388248	8	C	G	0.277	0.028	0.004	1.40E-11	3.83E-04	44.076	CAD
Cholesteryl Esters in Medium HDL	rs75714888	16	A	C	0.022	-0.071	0.013	3.70E-08	2.65E-04	30.513	CAD
Cholesteryl Esters in Medium HDL	rs75911530	16	A	G	0.033	-0.144	0.011	2.10E-42	0.002	174.811	CAD
Cholesteryl Esters in Medium HDL	rs77960347	18	G	A	0.013	0.303	0.016	4.00E-81	0.003	350.739	CAD
Cholesteryl Esters in Medium HDL	rs7810507	7	A	G	0.281	-0.025	0.004	5.00E-10	3.31E-04	38.095	CAD
Cholesteryl Esters in Medium HDL	rs7956099	12	C	T	0.475	-0.021	0.004	4.80E-09	2.85E-04	32.810	CAD
Cholesteryl Esters in Medium HDL	rs8058512	16	T	C	0.724	-0.025	0.004	1.20E-09	3.19E-04	36.761	CAD
Cholesteryl Esters in Medium HDL	rs838876	12	G	A	0.681	-0.041	0.004	3.50E-27	0.001	103.082	CAD
Cholesteryl Esters in Medium HDL	rs907866	2	A	G	0.443	-0.029	0.004	2.50E-15	0.001	61.820	CAD
Cholesteryl Esters in Medium HDL	rs9491697	6	G	A	0.464	-0.024	0.004	1.60E-10	3.50E-04	40.294	CAD
Cholesteryl Esters in Medium HDL	rs967645	17	T	C	0.517	-0.026	0.004	1.70E-11	4.20E-04	48.302	CAD
Cholesteryl Esters in Medium HDL	rs9687833	5	A	G	0.207	-0.030	0.005	4.50E-11	3.63E-04	41.795	CAD
Cholesteryl Esters in Medium HDL	rs10162642	15	A	G	0.210	-0.027	0.005	3.90E-10	2.79E-04	32.085	CAD
Cholesteryl Esters in Medium HDL	rs10184004	2	T	C	0.406	0.027	0.004	3.90E-13	4.34E-04	49.989	CAD

Cholesteryl Esters in Medium HDL	rs1047891	2	A	C	0.315	-0.027	0.004	1.00E-10	3.79E-04	43.625	CAD
Cholesteryl Esters in Medium HDL	rs1054852	12	G	A	0.377	0.032	0.004	1.20E-16	0.001	61.680	CAD
Cholesteryl Esters in Medium HDL	rs1077835	15	G	A	0.220	0.073	0.005	6.70E-60	0.002	255.033	CAD
Cholesteryl Esters in Medium HDL	rs11057692	12	G	A	0.236	-0.024	0.005	1.70E-08	2.40E-04	27.619	CAD
Cholesteryl Esters in Medium HDL	rs112001035	17	A	G	0.060	-0.060	0.008	3.50E-13	4.75E-04	54.732	CAD
Cholesteryl Esters in Medium HDL	rs114165349	1	C	G	0.023	-0.117	0.013	8.60E-21	0.001	83.576	CAD
Cholesteryl Esters in Medium HDL	rs116843064	19	A	G	0.020	0.195	0.014	1.70E-48	0.002	205.433	CAD
Cholesteryl Esters in Medium HDL	rs11751347	6	T	C	0.102	-0.041	0.006	7.80E-12	3.75E-04	43.190	CAD
Cholesteryl Esters in Medium HDL	rs11789603	9	T	C	0.109	0.050	0.006	1.20E-15	0.001	67.831	CAD
Cholesteryl Esters in Medium HDL	rs12533197	7	G	T	0.439	-0.022	0.004	2.50E-08	2.81E-04	32.372	CAD
Total Cholesterol in Medium HDL	rs10184004	2	T	C	0.406	0.021	0.004	5.60E-09	2.69E-04	30.958	CAD
Total Cholesterol in Medium HDL	rs1047891	2	A	C	0.315	-0.025	0.004	4.50E-09	3.15E-04	36.245	CAD
Total Cholesterol in Medium HDL	rs1065853	19	T	G	0.081	0.091	0.007	9.30E-42	0.001	171.079	CAD
Total Cholesterol in Medium HDL	rs1077835	15	G	A	0.220	0.120	0.005	1.70E-157	0.006	688.343	CAD
Total Cholesterol in Medium HDL	rs112001035	17	A	G	0.060	-0.053	0.008	2.80E-10	3.66E-04	42.085	CAD
Total Cholesterol in Medium HDL	rs112495680	6	G	A	0.133	-0.036	0.006	1.30E-10	3.55E-04	40.819	CAD
Total Cholesterol in Medium HDL	rs11671872	19	A	G	0.412	0.025	0.004	1.60E-10	3.64E-04	41.847	CAD
Total Cholesterol in Medium HDL	rs117687565	18	T	C	0.012	0.128	0.018	3.20E-12	4.44E-04	51.088	CAD
Total Cholesterol in Medium HDL	rs11789603	9	T	C	0.109	0.059	0.006	2.10E-21	0.001	94.417	CAD
Total Cholesterol in Medium HDL	rs1260326	2	C	T	0.604	-0.039	0.004	5.70E-23	0.001	101.287	CAD
Total Cholesterol in Medium HDL	rs12904367	15	A	G	0.141	0.034	0.006	6.40E-10	3.28E-04	37.759	CAD
Total Cholesterol in Medium HDL	rs12976739	19	A	G	0.396	0.026	0.004	7.50E-12	3.78E-04	43.506	CAD
Total Cholesterol in Medium HDL	rs13107325	4	T	C	0.074	-0.069	0.007	3.20E-23	0.001	92.059	CAD
Total Cholesterol in Medium HDL	rs1321257	1	A	G	0.613	0.047	0.004	2.80E-35	0.001	147.904	CAD
Total Cholesterol in Medium HDL	rs139915535	8	G	A	0.018	-0.119	0.014	2.10E-18	0.001	69.826	CAD
Total Cholesterol in Medium HDL	rs140168704	10	T	C	0.154	-0.028	0.005	4.60E-08	2.38E-04	27.371	CAD
Total Cholesterol in Medium HDL	rs144018203	11	C	G	0.011	-0.116	0.019	3.50E-09	3.17E-04	36.507	CAD
Total Cholesterol in Medium HDL	rs15285	8	T	C	0.287	0.082	0.004	8.60E-87	0.003	383.487	CAD

Total Cholesterol in Medium HDL	rs174578	11	A	T	0.351	-0.042	0.004	3.60E-28	0.001	112.159	CAD
Total Cholesterol in Medium HDL	rs17696736	12	G	A	0.430	-0.019	0.004	4.90E-08	2.07E-04	23.815	CAD
Total Cholesterol in Medium HDL	rs1800961	20	T	C	0.030	-0.123	0.011	3.40E-28	0.001	123.367	CAD
Total Cholesterol in Medium HDL	rs193084249	1	G	A	0.023	-0.097	0.013	2.90E-14	4.99E-04	57.419	CAD
Total Cholesterol in Medium HDL	rs2043085	15	C	T	0.613	-0.110	0.004	5.90E-181	0.007	804.454	CAD
Total Cholesterol in Medium HDL	rs2066714	9	C	T	0.129	0.037	0.006	1.00E-11	3.71E-04	42.655	CAD
Total Cholesterol in Medium HDL	rs2176040	2	G	A	0.647	-0.029	0.004	1.60E-15	4.83E-04	55.596	CAD
Total Cholesterol in Medium HDL	rs2236252	20	T	C	0.167	-0.031	0.005	3.70E-10	3.21E-04	36.955	CAD
Total Cholesterol in Medium HDL	rs2298428	22	T	C	0.183	-0.041	0.005	2.00E-17	0.001	69.201	CAD
Total Cholesterol in Medium HDL	rs235314	21	T	C	0.533	-0.021	0.004	4.80E-08	2.63E-04	30.220	CAD
Total Cholesterol in Medium HDL	rs2494748	14	T	C	0.616	-0.022	0.004	1.80E-09	2.84E-04	32.721	CAD
Total Cholesterol in Medium HDL	rs2642438	1	G	A	0.704	0.036	0.004	8.30E-19	0.001	74.375	CAD
Total Cholesterol in Medium HDL	rs267738	1	G	T	0.220	0.034	0.005	1.20E-14	4.75E-04	54.639	CAD
Total Cholesterol in Medium HDL	rs2740488	9	C	A	0.265	-0.050	0.004	1.10E-32	0.001	136.346	CAD
Total Cholesterol in Medium HDL	rs2792735	10	A	G	0.720	-0.040	0.004	5.90E-23	0.001	91.908	CAD
Total Cholesterol in Medium HDL	rs2925979	16	C	T	0.699	0.030	0.004	1.40E-14	4.62E-04	53.238	CAD
Total Cholesterol in Medium HDL	rs35135293	2	T	C	0.517	-0.029	0.004	1.50E-14	4.92E-04	56.702	CAD
Total Cholesterol in Medium HDL	rs35184771	11	T	G	0.353	-0.037	0.004	3.10E-20	0.001	86.173	CAD
Total Cholesterol in Medium HDL	rs36057735	6	G	C	0.199	-0.036	0.005	5.00E-14	4.93E-04	56.706	CAD
Total Cholesterol in Medium HDL	rs3735687	7	G	A	0.423	-0.023	0.004	7.10E-09	3.15E-04	36.276	CAD
Total Cholesterol in Medium HDL	rs3764261	16	A	C	0.324	0.174	0.004	1.00E-200	0.016	1862.564	CAD
Total Cholesterol in Medium HDL	rs3768321	1	T	G	0.197	-0.041	0.005	7.00E-19	0.001	73.034	CAD
Total Cholesterol in Medium HDL	rs4239651	20	C	T	0.794	0.036	0.005	6.10E-16	0.001	60.636	CAD
Total Cholesterol in Medium HDL	rs4240624	8	A	G	0.909	0.100	0.007	4.00E-53	0.002	232.481	CAD
Total Cholesterol in Medium HDL	rs4759375	12	T	C	0.085	0.040	0.007	2.20E-08	2.90E-04	33.368	CAD
Total Cholesterol in Medium HDL	rs4969141	17	T	C	0.490	0.021	0.004	2.20E-08	2.66E-04	30.582	CAD
Total Cholesterol in Medium HDL	rs4986970	16	T	A	0.034	-0.074	0.010	3.10E-13	4.33E-04	49.889	CAD
Total Cholesterol in Medium HDL	rs559355	11	T	A	0.158	-0.063	0.005	6.60E-34	0.001	146.911	CAD

Total Cholesterol in Medium HDL	rs583104	1	T	G	0.773	-0.028	0.005	1.10E-10	3.41E-04	39.266	CAD
Total Cholesterol in Medium HDL	rs59347135	8	G	C	0.046	-0.064	0.009	6.50E-12	4.07E-04	46.822	CAD
Total Cholesterol in Medium HDL	rs60847460	10	T	C	0.143	-0.038	0.005	7.20E-12	4.18E-04	48.174	CAD
Total Cholesterol in Medium HDL	rs61805076	1	C	T	0.334	-0.026	0.004	2.60E-11	3.70E-04	42.607	CAD
Total Cholesterol in Medium HDL	rs61941676	12	A	C	0.127	-0.055	0.006	7.40E-22	0.001	90.794	CAD
Total Cholesterol in Medium HDL	rs62101704	18	A	G	0.014	-0.091	0.017	1.20E-08	2.60E-04	29.962	CAD
Total Cholesterol in Medium HDL	rs625145	11	T	A	0.188	0.074	0.005	4.80E-56	0.002	233.352	CAD
Total Cholesterol in Medium HDL	rs638714	1	T	G	0.346	-0.046	0.004	2.10E-31	0.001	131.833	CAD
Total Cholesterol in Medium HDL	rs6606717	12	C	A	0.523	0.031	0.004	2.70E-16	0.001	65.369	CAD
Total Cholesterol in Medium HDL	rs676210	2	A	G	0.206	0.056	0.005	8.20E-33	0.001	146.183	CAD
Total Cholesterol in Medium HDL	rs686030	9	A	C	0.860	0.044	0.005	2.00E-16	0.001	65.225	CAD
Total Cholesterol in Medium HDL	rs72836561	17	T	C	0.031	-0.128	0.011	1.50E-33	0.001	138.891	CAD
Total Cholesterol in Medium HDL	rs737338	19	T	C	0.035	-0.118	0.010	1.90E-32	0.001	132.694	CAD
Total Cholesterol in Medium HDL	rs7388248	8	C	G	0.277	0.028	0.004	2.50E-11	3.75E-04	43.179	CAD
Total Cholesterol in Medium HDL	rs75911530	16	A	G	0.033	-0.139	0.011	5.10E-38	0.001	157.086	CAD
Total Cholesterol in Medium HDL	rs77960347	18	G	A	0.013	0.317	0.017	8.80E-85	0.003	369.906	CAD
Total Cholesterol in Medium HDL	rs7810507	7	A	G	0.281	-0.023	0.004	2.10E-08	2.67E-04	30.687	CAD
Total Cholesterol in Medium HDL	rs7956099	12	C	T	0.475	-0.022	0.004	1.40E-09	3.02E-04	34.739	CAD
Total Cholesterol in Medium HDL	rs8058512	16	T	C	0.724	-0.024	0.004	1.90E-08	2.77E-04	31.881	CAD
Total Cholesterol in Medium HDL	rs838876	12	G	A	0.681	-0.038	0.004	2.30E-22	0.001	85.576	CAD
Total Cholesterol in Medium HDL	rs9304381	18	T	C	0.818	0.094	0.005	2.70E-83	0.003	364.938	CAD
Total Cholesterol in Medium HDL	rs9471972	6	A	G	0.536	0.023	0.004	2.80E-09	3.16E-04	36.360	CAD
Total Cholesterol in Medium HDL	rs9491697	6	G	A	0.464	-0.021	0.004	3.20E-08	2.65E-04	30.462	CAD
Total Cholesterol in Medium HDL	rs967645	17	T	C	0.517	-0.028	0.004	2.20E-12	4.60E-04	52.948	CAD
Total Cholesterol in Medium HDL	rs9884390	4	C	T	0.234	0.025	0.005	6.70E-09	2.65E-04	30.491	CAD
Total Cholesterol in Medium HDL	rs10015477	4	A	G	0.368	-0.021	0.004	7.30E-09	2.60E-04	29.957	CAD
Total Cholesterol in Medium HDL	rs10162642	15	A	G	0.210	-0.033	0.005	3.20E-15	4.46E-04	51.311	CAD
Total Cholesterol in Medium HDL	rs10268632	7	C	A	0.512	0.023	0.004	5.60E-10	3.21E-04	37.009	CAD

Total Cholesterol in Medium HDL	rs1047891	2	A	C	0.315	-0.024	0.004	3.30E-09	3.21E-04	36.979	CAD
Total Cholesterol in Medium HDL	rs1047964	11	T	G	0.010	0.206	0.019	3.80E-28	0.001	119.010	CAD
Total Cholesterol in Medium HDL	rs1054852	12	G	A	0.377	0.034	0.004	1.40E-18	0.001	70.735	CAD
Total Cholesterol in Medium HDL	rs11057692	12	G	A	0.236	-0.026	0.004	4.30E-10	3.01E-04	34.689	CAD
Total Cholesterol in Medium HDL	rs112001035	17	A	G	0.060	-0.060	0.008	4.60E-14	4.91E-04	56.565	CAD
Total Cholesterol in Medium HDL	rs114165349	1	C	G	0.023	-0.114	0.012	2.90E-20	0.001	84.196	CAD
Total Cholesterol in Medium HDL	rs116843064	19	A	G	0.020	0.223	0.013	4.10E-66	0.002	280.690	CAD
Total Cholesterol in Medium HDL	rs116978226	11	A	C	0.033	0.065	0.010	1.40E-10	3.33E-04	38.354	CAD
Total Cholesterol in Medium HDL	rs11789603	9	T	C	0.109	0.051	0.006	1.60E-16	0.001	73.194	CAD
Total Cholesterol in Medium HDL	rs1215112	9	A	G	0.863	0.047	0.005	1.50E-18	0.001	74.629	CAD
Total Concentration of Medium HDL Particles	rs9491697	6	G	A	0.464	-0.026	0.004	1.40E-11	4.02E-04	46.256	CAD
Total Concentration of Medium HDL Particles	rs9647335	3	T	A	0.192	0.033	0.005	1.20E-11	4.01E-04	46.107	CAD
Total Concentration of Medium HDL Particles	rs967645	17	T	C	0.517	-0.026	0.004	4.50E-11	4.06E-04	46.771	CAD
Total Concentration of Medium HDL Particles	rs9687846	5	A	G	0.201	-0.032	0.005	9.00E-12	4.00E-04	46.072	CAD
Total Concentration of Medium HDL Particles	rs1047891	2	A	C	0.315	-0.025	0.004	2.60E-09	3.15E-04	36.224	CAD
Total Concentration of Medium HDL Particles	rs1065853	19	T	G	0.081	0.162	0.007	2.30E-121	0.005	524.318	CAD
Total Concentration of Medium HDL Particles	rs1077835	15	G	A	0.220	0.102	0.005	9.71E-111	0.004	480.133	CAD
Total Concentration of Medium HDL Particles	rs11039238	11	C	T	0.322	-0.035	0.004	3.90E-18	0.001	74.872	CAD
Total Concentration of Medium HDL Particles	rs112001035	17	A	G	0.060	-0.051	0.008	1.80E-09	3.31E-04	38.095	CAD
Total Concentration of Medium HDL Particles	rs112495680	6	G	A	0.133	-0.034	0.006	5.20E-10	3.22E-04	37.096	CAD
Total Concentration of Medium HDL Particles	rs11601507	11	A	C	0.069	-0.039	0.007	3.60E-08	2.37E-04	27.326	CAD
Total Concentration of Medium HDL Particles	rs117310449	19	T	C	0.012	-0.098	0.018	1.90E-08	2.66E-04	30.644	CAD
Total Concentration of Medium HDL Particles	rs117687565	18	T	C	0.012	0.138	0.018	1.10E-13	4.99E-04	57.506	CAD
Total Concentration of Medium HDL Particles	rs11789603	9	T	C	0.109	0.050	0.006	1.40E-15	0.001	66.660	CAD
Total Concentration of Medium HDL Particles	rs1260326	2	C	T	0.604	-0.046	0.004	3.30E-30	0.001	135.889	CAD
Total Concentration of Medium HDL Particles	rs12904367	15	A	G	0.141	0.031	0.006	4.80E-08	2.57E-04	29.598	CAD
Total Concentration of Medium HDL Particles	rs12976739	19	A	G	0.396	0.025	0.004	8.90E-11	3.38E-04	38.953	CAD
Total Concentration of Medium HDL Particles	rs13107325	4	T	C	0.074	-0.065	0.007	1.10E-19	0.001	79.241	CAD

Total Concentration of Medium HDL Particles	rs1321257	1	A	G	0.613	0.051	0.004	5.10E-39	0.001	165.264	CAD
Total Concentration of Medium HDL Particles	rs139915535	8	G	A	0.018	-0.123	0.014	9.50E-19	0.001	72.176	CAD
Total Concentration of Medium HDL Particles	rs140168704	10	T	C	0.154	-0.029	0.005	1.80E-08	2.50E-04	28.736	CAD
Total Concentration of Medium HDL Particles	rs15285	8	T	C	0.287	0.082	0.004	8.50E-85	0.003	371.535	CAD
Total Concentration of Medium HDL Particles	rs1800961	20	T	C	0.030	-0.113	0.011	2.10E-23	0.001	101.509	CAD
Total Concentration of Medium HDL Particles	rs193084249	1	G	A	0.023	-0.102	0.013	2.10E-15	0.001	62.125	CAD
Total Concentration of Medium HDL Particles	rs2043085	15	C	T	0.613	-0.098	0.004	1.70E-139	0.005	617.631	CAD
Total Concentration of Medium HDL Particles	rs2072113	11	T	C	0.116	-0.045	0.006	7.60E-15	4.91E-04	56.546	CAD
Total Concentration of Medium HDL Particles	rs2176040	2	G	A	0.647	-0.025	0.004	1.30E-11	3.46E-04	39.791	CAD
Total Concentration of Medium HDL Particles	rs2236252	20	T	C	0.167	-0.032	0.005	6.50E-11	3.41E-04	39.264	CAD
Total Concentration of Medium HDL Particles	rs2298428	22	T	C	0.183	-0.042	0.005	4.10E-18	0.001	72.391	CAD
Total Concentration of Medium HDL Particles	rs235314	21	T	C	0.533	-0.021	0.004	4.50E-08	2.71E-04	31.139	CAD
Total Concentration of Medium HDL Particles	rs2569550	19	C	T	0.593	-0.027	0.004	5.40E-12	4.12E-04	47.464	CAD
Total Concentration of Medium HDL Particles	rs2642438	1	G	A	0.704	0.035	0.004	1.90E-17	0.001	68.723	CAD
Total Concentration of Medium HDL Particles	rs267738	1	G	T	0.220	0.039	0.005	5.50E-18	0.001	69.686	CAD
Total Concentration of Medium HDL Particles	rs2740488	9	C	A	0.265	-0.038	0.004	3.40E-19	0.001	75.368	CAD
Total Concentration of Medium HDL Particles	rs2792735	10	A	G	0.720	-0.039	0.004	1.20E-20	0.001	81.882	CAD
Total Concentration of Medium HDL Particles	rs28818616	3	C	T	0.350	-0.022	0.004	4.80E-08	2.49E-04	28.645	CAD
Total Concentration of Medium HDL Particles	rs2925979	16	C	T	0.699	0.028	0.004	2.90E-12	3.80E-04	43.775	CAD
Total Concentration of Medium HDL Particles	rs35135293	2	T	C	0.517	-0.028	0.004	9.30E-14	4.51E-04	51.898	CAD
Total Concentration of Medium HDL Particles	rs36057735	6	G	C	0.199	-0.036	0.005	1.90E-13	4.81E-04	55.364	CAD
Total Concentration of Medium HDL Particles	rs3764261	16	A	C	0.324	0.160	0.004	1.00E-200	0.013	1528.898	CAD
Total Concentration of Medium HDL Particles	rs3768321	1	T	G	0.197	-0.038	0.005	9.00E-16	0.001	60.957	CAD
Total Concentration of Medium HDL Particles	rs3795269	1	A	C	0.439	0.022	0.004	7.00E-09	2.81E-04	32.344	CAD
Total Concentration of Medium HDL Particles	rs3859588	20	A	T	0.217	-0.026	0.005	4.50E-08	2.69E-04	30.980	CAD
Total Concentration of Medium HDL Particles	rs4239651	20	C	T	0.794	0.036	0.005	3.10E-15	0.001	57.937	CAD
Total Concentration of Medium HDL Particles	rs4240624	8	A	G	0.909	0.101	0.007	7.30E-52	0.002	226.539	CAD
Total Concentration of Medium HDL Particles	rs4656292	1	G	A	0.620	0.024	0.004	7.40E-11	3.17E-04	36.545	CAD

Total Concentration of Medium HDL Particles	rs4969141	17	T	C	0.490	0.021	0.004	3.20E-08	2.52E-04	28.961	CAD
Total Concentration of Medium HDL Particles	rs4986970	16	T	A	0.034	-0.061	0.011	1.30E-09	2.89E-04	33.271	CAD
Total Concentration of Medium HDL Particles	rs559355	11	T	A	0.158	-0.069	0.005	1.10E-40	0.002	173.418	CAD
Total Concentration of Medium HDL Particles	rs583104	1	T	G	0.773	-0.051	0.005	1.90E-30	0.001	124.591	CAD
Total Concentration of Medium HDL Particles	rs59347135	8	G	C	0.046	-0.066	0.009	2.70E-12	4.23E-04	48.649	CAD
Total Concentration of Medium HDL Particles	rs60847460	10	T	C	0.143	-0.038	0.005	1.50E-11	4.08E-04	46.919	CAD
Total Concentration of Medium HDL Particles	rs61805076	1	C	T	0.334	-0.026	0.004	6.60E-11	3.57E-04	41.127	CAD
Total Concentration of Medium HDL Particles	rs61941676	12	A	C	0.127	-0.055	0.006	1.40E-20	0.001	86.332	CAD
Total Concentration of Medium HDL Particles	rs62101704	18	A	G	0.014	-0.089	0.017	4.50E-08	2.45E-04	28.242	CAD
Total Concentration of Medium HDL Particles	rs625145	11	T	A	0.188	0.071	0.005	3.40E-50	0.002	208.425	CAD
Total Concentration of Medium HDL Particles	rs638714	1	T	G	0.346	-0.051	0.004	6.30E-37	0.001	157.249	CAD
Total Concentration of Medium HDL Particles	rs6511720	19	T	G	0.120	0.049	0.006	7.70E-18	0.001	69.998	CAD
Total Concentration of Medium HDL Particles	rs6606717	12	C	A	0.523	0.031	0.004	4.30E-16	0.001	64.937	CAD
Total Concentration of Medium HDL Particles	rs676210	2	A	G	0.206	0.061	0.005	2.10E-37	0.001	167.166	CAD
Total Concentration of Medium HDL Particles	rs686030	9	A	C	0.860	0.035	0.006	1.70E-10	3.42E-04	39.317	CAD
Total Concentration of Medium HDL Particles	rs72836561	17	T	C	0.031	-0.125	0.011	2.90E-31	0.001	130.452	CAD
Total Concentration of Medium HDL Particles	rs737338	19	T	C	0.035	-0.124	0.010	7.20E-34	0.001	141.005	CAD
Total Concentration of Medium HDL Particles	rs7388248	8	C	G	0.277	0.030	0.004	4.10E-12	4.26E-04	49.036	CAD
Total Concentration of Medium HDL Particles	rs75911530	16	A	G	0.033	-0.135	0.011	3.30E-34	0.001	142.249	CAD
Total Concentration of Medium HDL Particles	rs77960347	18	G	A	0.013	0.327	0.017	4.10E-87	0.003	381.976	CAD
Total Concentration of Medium HDL Particles	rs7956099	12	C	T	0.475	-0.024	0.004	1.20E-10	3.47E-04	39.924	CAD
Total Concentration of Medium HDL Particles	rs838876	12	G	A	0.681	-0.037	0.004	6.00E-20	0.001	76.999	CAD
Total Concentration of Medium HDL Particles	rs9304381	18	T	C	0.818	0.097	0.005	1.40E-85	0.003	377.650	CAD
Total Concentration of Medium HDL Particles	rs9471972	6	A	G	0.536	0.027	0.004	1.20E-12	4.40E-04	50.702	CAD
Total Concentration of Medium HDL Particles	rs967645	17	T	C	0.517	-0.031	0.004	5.80E-15	0.001	66.109	CAD
Total Concentration of Medium HDL Particles	rs10184004	2	T	C	0.406	0.021	0.004	1.00E-08	2.66E-04	30.570	CAD
Total Concentration of Medium HDL Particles	rs1047891	2	A	C	0.315	-0.026	0.004	5.20E-10	3.45E-04	39.734	CAD
Total Concentration of Medium HDL Particles	rs1065853	19	T	G	0.081	0.139	0.007	8.90E-92	0.003	391.111	CAD

Total Lipids in HDL	rs1260326	2	C	T	0.604	-0.025	0.004	4.60E-10	3.61E-04	41.522	CAD
Total Lipids in HDL	rs12608026	18	G	T	0.042	0.083	0.010	6.20E-19	0.001	76.846	CAD
Total Lipids in HDL	rs13107325	4	T	C	0.074	-0.071	0.007	2.30E-23	0.001	95.677	CAD
Total Lipids in HDL	rs1358980	6	T	C	0.483	-0.027	0.004	1.40E-12	4.36E-04	50.181	CAD
Total Lipids in HDL	rs139915535	8	G	A	0.018	-0.146	0.014	5.60E-26	0.001	104.363	CAD
Total Lipids in HDL	rs141368429	11	T	C	0.056	-0.054	0.009	1.50E-09	3.25E-04	37.433	CAD
Total Lipids in HDL	rs144311893	19	T	C	0.022	0.112	0.013	1.50E-18	0.001	71.711	CAD
Total Lipids in HDL	rs15285	8	T	C	0.287	0.102	0.004	7.40E-133	0.005	587.932	CAD
Total Lipids in HDL	rs1601935	15	T	G	0.655	-0.073	0.004	1.00E-75	0.003	330.092	CAD
Total Lipids in HDL	rs174578	11	A	T	0.351	-0.042	0.004	2.40E-27	0.001	110.084	CAD
Total Lipids in HDL	rs1761457	19	G	A	0.327	0.027	0.004	1.30E-12	3.88E-04	44.720	CAD
Total Lipids in HDL	rs1800961	20	T	C	0.030	-0.123	0.011	3.90E-28	0.001	122.448	CAD
Total Lipids in HDL	rs2229357	12	A	G	0.241	0.027	0.004	2.80E-10	3.28E-04	37.758	CAD
Total Lipids in HDL	rs2236252	20	T	C	0.167	-0.029	0.005	4.30E-09	2.74E-04	31.563	CAD
Total Lipids in HDL	rs2245221	8	A	G	0.559	0.033	0.004	4.60E-16	0.001	71.320	CAD
Total Lipids in HDL	rs2298428	22	T	C	0.183	-0.040	0.005	1.20E-16	0.001	65.392	CAD
Total Lipids in HDL	rs2395943	6	G	A	0.588	0.023	0.004	2.90E-09	3.15E-04	36.223	CAD
Total Lipids in HDL	rs2494748	14	T	C	0.616	-0.022	0.004	4.60E-09	2.69E-04	30.994	CAD
Total Lipids in HDL	rs2642438	1	G	A	0.704	0.036	0.004	1.80E-18	0.001	73.181	CAD
Total Lipids in HDL	rs267738	1	G	T	0.220	0.038	0.005	6.20E-18	0.001	69.470	CAD
Total Lipids in HDL	rs2740488	9	C	A	0.265	-0.039	0.004	2.60E-20	0.001	80.976	CAD
Total Lipids in HDL	rs2792735	10	A	G	0.720	-0.039	0.004	2.20E-21	0.001	83.836	CAD
Total Lipids in HDL	rs28746853	6	C	T	0.176	0.030	0.005	4.80E-08	2.63E-04	30.322	CAD
Total Lipids in HDL	rs28818616	3	C	T	0.350	-0.022	0.004	2.30E-08	2.51E-04	28.869	CAD
Total Lipids in HDL	rs2925979	16	C	T	0.699	0.034	0.004	3.20E-18	0.001	68.714	CAD
Total Lipids in HDL	rs2943650	2	T	C	0.645	-0.034	0.004	5.40E-19	0.001	71.760	CAD
Total Lipids in HDL	rs2978615	19	T	C	0.490	-0.025	0.004	2.80E-11	3.77E-04	43.396	CAD
Total Lipids in HDL	rs35135293	2	T	C	0.517	-0.024	0.004	9.90E-11	3.46E-04	39.776	CAD

Total Lipids in HDL	rs35184771	11	T	G	0.353	-0.036	0.004	7.10E-20	0.001	84.554	CAD
Total Lipids in HDL	rs36057735	6	G	C	0.199	-0.034	0.005	1.00E-12	4.35E-04	50.043	CAD
Total Lipids in HDL	rs3764261	16	A	C	0.324	0.184	0.004	1.00E-200	0.018	2075.409	CAD
Total Lipids in HDL	rs3768321	1	T	G	0.197	-0.043	0.005	2.50E-20	0.001	79.976	CAD
Total Lipids in HDL	rs3795269	1	A	C	0.439	0.020	0.004	4.80E-08	2.49E-04	28.627	CAD
Total Lipids in HDL	rs4239651	20	C	T	0.794	0.036	0.005	1.90E-15	4.99E-04	57.409	CAD
Total Lipids in HDL	rs4240624	8	A	G	0.909	0.103	0.007	4.20E-55	0.002	240.716	CAD
Total Lipids in HDL	rs429358	19	C	T	0.155	-0.075	0.005	8.60E-49	0.002	202.758	CAD
Total Lipids in HDL	rs4330777	16	A	G	0.476	-0.020	0.004	3.20E-08	2.34E-04	26.920	CAD
Total Lipids in HDL	rs4656292	1	G	A	0.620	0.020	0.004	2.90E-08	2.22E-04	25.495	CAD
Total Lipids in HDL	rs4846921	1	A	G	0.613	0.052	0.004	1.60E-42	0.002	178.855	CAD
Total Lipids in HDL	rs4969141	17	T	C	0.490	0.024	0.004	1.50E-10	3.45E-04	39.716	CAD
Total Lipids in HDL	rs4986970	16	T	A	0.034	-0.076	0.011	1.40E-13	4.58E-04	52.672	CAD
Total Lipids in HDL	rs559355	11	T	A	0.158	-0.063	0.005	1.80E-34	0.001	146.595	CAD
Total Lipids in HDL	rs583104	1	T	G	0.773	-0.040	0.005	3.40E-19	0.001	75.945	CAD
Total Lipids in HDL	rs59097294	11	C	T	0.181	0.065	0.005	1.00E-42	0.002	176.049	CAD
Total Lipids in HDL	rs59347135	8	G	C	0.046	-0.077	0.009	3.90E-17	0.001	68.300	CAD
Total Lipids in HDL	rs60847460	10	T	C	0.143	-0.038	0.005	5.20E-12	4.27E-04	49.210	CAD
Total Lipids in HDL	rs61805076	1	C	T	0.334	-0.028	0.004	2.50E-12	4.13E-04	47.576	CAD
Total Lipids in HDL	rs62101705	18	T	C	0.014	-0.091	0.017	2.10E-08	2.56E-04	29.470	CAD
Total Lipids in HDL	rs638714	1	T	G	0.346	-0.028	0.004	6.40E-13	4.28E-04	49.323	CAD
Total Lipids in HDL	rs6589565	11	G	A	0.932	0.072	0.008	1.30E-23	0.001	91.613	CAD
Total Lipids in HDL	rs6606717	12	C	A	0.523	0.027	0.004	4.60E-13	4.40E-04	50.626	CAD
Total Lipids in HDL	rs676210	2	A	G	0.206	0.075	0.005	8.80E-57	0.002	256.445	CAD
Total Lipids in HDL	rs686030	9	A	C	0.860	0.039	0.005	4.10E-13	4.43E-04	50.998	CAD
Total Lipids in HDL	rs6967917	7	G	A	0.492	0.022	0.004	2.60E-09	2.86E-04	32.939	CAD
Total Lipids in HDL	rs7134375	12	A	C	0.430	0.021	0.004	1.30E-08	2.60E-04	29.937	CAD
Total Lipids in HDL	rs7136506	12	C	T	0.216	-0.038	0.005	1.80E-16	0.001	64.308	CAD

Total Lipids in HDL	rs7241918	18	T	G	0.823	0.087	0.005	6.60E-70	0.003	305.506	CAD
Total Lipids in HDL	rs72836561	17	T	C	0.031	-0.148	0.011	8.70E-44	0.002	185.879	CAD
Total Lipids in HDL	rs737337	19	C	T	0.077	-0.068	0.007	1.80E-21	0.001	89.966	CAD
Total Lipids in HDL	rs7388248	8	C	G	0.277	0.026	0.004	1.50E-09	3.17E-04	36.453	CAD
Total Lipids in HDL	rs75911530	16	A	G	0.033	-0.153	0.011	5.50E-44	0.002	186.738	CAD
Total Lipids in HDL	rs77960347	18	G	A	0.013	0.288	0.017	6.50E-70	0.003	301.170	CAD
Total Lipids in HDL	rs7810507	7	A	G	0.281	-0.027	0.004	1.30E-10	3.61E-04	41.600	CAD
Total Lipids in HDL	rs78296522	11	A	C	0.045	0.065	0.009	2.40E-12	4.24E-04	48.762	CAD
Total Lipids in HDL	rs7956099	12	C	T	0.475	-0.021	0.004	1.80E-08	2.60E-04	29.922	CAD
Total Lipids in HDL	rs8058512	16	T	C	0.724	-0.025	0.004	5.60E-09	3.00E-04	34.570	CAD
Total Lipids in HDL	rs904770	16	C	T	0.889	-0.035	0.006	1.10E-08	2.87E-04	33.050	CAD
Total Lipids in HDL	rs921919	12	A	G	0.670	-0.038	0.004	2.20E-22	0.001	85.595	CAD
Total Lipids in HDL	rs9491697	6	G	A	0.464	-0.025	0.004	2.90E-11	3.85E-04	44.376	CAD
Total Lipids in HDL	rs9647335	3	T	A	0.192	0.031	0.005	2.20E-10	3.54E-04	40.708	CAD
Total Lipids in HDL	rs967645	17	T	C	0.517	-0.026	0.004	2.40E-11	4.19E-04	48.231	CAD
Total Lipids in HDL	rs9687846	5	A	G	0.201	-0.031	0.005	3.50E-11	3.75E-04	43.171	CAD
Total Lipids in HDL	rs10184004	2	T	C	0.406	0.028	0.004	8.80E-14	4.61E-04	53.108	CAD
Total Lipids in HDL	rs1047891	2	A	C	0.315	-0.028	0.004	3.30E-11	3.97E-04	45.732	CAD
Total Lipids in HDL	rs1077835	15	G	A	0.220	0.061	0.005	3.70E-41	0.001	172.706	CAD
Total Lipids in HDL	rs11057692	12	G	A	0.236	-0.024	0.005	1.60E-08	2.41E-04	27.748	CAD
Total Lipids in HDL	rs112001035	17	A	G	0.060	-0.062	0.008	7.90E-14	4.97E-04	57.267	CAD
Total Lipids in HDL	rs114165349	1	C	G	0.023	-0.121	0.013	3.90E-22	0.001	89.526	CAD
Total Lipids in HDL	rs11591147	1	T	G	0.017	0.093	0.015	8.00E-11	3.53E-04	40.592	CAD
Total Lipids in HDL	rs116843064	19	A	G	0.020	0.201	0.014	6.10E-51	0.002	216.573	CAD
Total Lipids in HDL	rs11751347	6	T	C	0.102	-0.043	0.006	2.40E-12	3.92E-04	45.151	CAD
Total Lipids in HDL	rs117687565	18	T	C	0.012	0.117	0.018	7.30E-10	3.59E-04	41.307	CAD
Total Lipids in HDL	rs11789603	9	T	C	0.109	0.049	0.006	1.10E-14	0.001	63.304	CAD
Total Lipids in HDL	rs12533197	7	G	T	0.439	-0.022	0.004	2.50E-08	2.81E-04	32.337	CAD

Total Lipids in HDL	rs1260326	2	C	T	0.604	-0.023	0.004	1.30E-08	3.01E-04	34.691	CAD
Total Lipids in Medium HDL	rs12611067	19	T	G	0.351	0.025	0.004	1.60E-09	3.26E-04	37.582	CAD
Total Lipids in Medium HDL	rs13107325	4	T	C	0.074	-0.071	0.007	6.70E-23	0.001	94.357	CAD
Total Lipids in Medium HDL	rs1358980	6	T	C	0.483	-0.028	0.004	3.20E-13	4.56E-04	52.464	CAD
Total Lipids in Medium HDL	rs139915535	8	G	A	0.018	-0.150	0.014	5.80E-27	0.001	109.244	CAD
Total Lipids in Medium HDL	rs141368429	11	T	C	0.056	-0.055	0.009	1.00E-09	3.30E-04	37.996	CAD
Total Lipids in Medium HDL	rs144311893	19	T	C	0.022	0.121	0.013	4.90E-21	0.001	82.599	CAD
Total Lipids in Medium HDL	rs15285	8	T	C	0.287	0.105	0.004	5.40E-140	0.005	618.018	CAD
Total Lipids in Medium HDL	rs1601935	15	T	G	0.655	-0.063	0.004	1.70E-55	0.002	239.733	CAD
Total Lipids in Medium HDL	rs174578	11	A	T	0.351	-0.039	0.004	1.00E-23	0.001	94.661	CAD
Total Lipids in Medium HDL	rs1761457	19	G	A	0.327	0.028	0.004	3.60E-13	4.11E-04	47.327	CAD
Total Lipids in Medium HDL	rs1800961	20	T	C	0.030	-0.123	0.011	4.80E-28	0.001	121.932	CAD
Total Lipids in Medium HDL	rs2228671	19	T	C	0.126	0.040	0.006	2.70E-13	4.15E-04	47.766	CAD
Total Lipids in Medium HDL	rs2229357	12	A	G	0.241	0.028	0.004	1.10E-10	3.47E-04	39.923	CAD
Total Lipids in Medium HDL	rs2236252	20	T	C	0.167	-0.028	0.005	5.50E-09	2.68E-04	30.802	CAD
Total Lipids in Medium HDL	rs2245221	8	A	G	0.559	0.032	0.004	1.60E-15	0.001	69.101	CAD
Total Lipids in Medium HDL	rs2298428	22	T	C	0.183	-0.039	0.005	3.50E-16	0.001	63.567	CAD
Total Lipids in Medium HDL	rs2395943	6	G	A	0.588	0.024	0.004	1.70E-09	3.25E-04	37.409	CAD
Total Lipids in Medium HDL	rs2494748	14	T	C	0.616	-0.022	0.004	3.30E-09	2.77E-04	31.885	CAD
Total Lipids in Medium HDL	rs2642438	1	G	A	0.704	0.035	0.004	9.70E-18	0.001	70.165	CAD
Total Lipids in Medium HDL	rs267738	1	G	T	0.220	0.039	0.005	1.80E-18	0.001	72.135	CAD
Total Lipids in Medium HDL	rs2740488	9	C	A	0.265	-0.038	0.004	5.30E-19	0.001	75.122	CAD
Total Lipids in Medium HDL	rs2792735	10	A	G	0.720	-0.038	0.004	2.00E-20	0.001	79.504	CAD
Total Lipids in Medium HDL	rs28746853	6	C	T	0.176	0.030	0.005	4.00E-08	2.64E-04	30.350	CAD
Total Lipids in Medium HDL	rs28818616	3	C	T	0.350	-0.022	0.004	2.30E-08	2.49E-04	28.623	CAD
Total Lipids in Medium HDL	rs2925979	16	C	T	0.699	0.035	0.004	8.20E-19	0.001	71.046	CAD
Total Lipids in Medium HDL	rs2943650	2	T	C	0.645	-0.034	0.004	1.70E-19	0.001	73.959	CAD
Total Lipids in Medium HDL	rs35135293	2	T	C	0.517	-0.023	0.004	6.70E-10	3.15E-04	36.208	CAD

Total Lipids in Medium HDL	rs35184771	11	T	G	0.353	-0.036	0.004	1.90E-19	0.001	82.723	CAD
Total Lipids in Medium HDL	rs36057735	6	G	C	0.199	-0.033	0.005	3.20E-12	4.15E-04	47.780	CAD
Total Lipids in Medium HDL	rs3768321	1	T	G	0.197	-0.044	0.005	9.30E-21	0.001	82.126	CAD
Total Lipids in Medium HDL	rs3795269	1	A	C	0.439	0.021	0.004	3.30E-08	2.55E-04	29.311	CAD
Total Lipids in Medium HDL	rs4239651	20	C	T	0.794	0.035	0.005	4.70E-15	4.83E-04	55.615	CAD
Total Lipids in Medium HDL	rs4240624	8	A	G	0.909	0.103	0.007	4.40E-55	0.002	240.275	CAD
Total Lipids in Medium HDL	rs429358	19	C	T	0.155	-0.079	0.005	4.20E-54	0.002	225.227	CAD
Total Lipids in Medium HDL	rs4330777	16	A	G	0.476	-0.020	0.004	3.40E-08	2.32E-04	26.758	CAD
Total Lipids in Medium HDL	rs4656292	1	G	A	0.620	0.021	0.004	3.90E-09	2.52E-04	29.062	CAD
Total Lipids in Medium HDL	rs4846921	1	A	G	0.613	0.052	0.004	1.10E-42	0.002	179.924	CAD
Total Lipids in Medium HDL	rs4969141	17	T	C	0.490	0.025	0.004	5.50E-11	3.60E-04	41.436	CAD
Total Lipids in Medium HDL	rs4986970	16	T	A	0.034	-0.076	0.011	2.20E-13	4.51E-04	51.979	CAD
Total Lipids in Medium HDL	rs559355	11	T	A	0.158	-0.063	0.005	7.60E-34	0.001	143.872	CAD
Total Lipids in Medium HDL	rs583104	1	T	G	0.773	-0.042	0.005	5.60E-21	0.001	84.118	CAD
Total Lipids in Medium HDL	rs59097294	11	C	T	0.181	0.064	0.005	3.60E-41	0.001	169.471	CAD
Total Lipids in Medium HDL	rs59347135	8	G	C	0.046	-0.079	0.009	7.00E-18	0.001	71.365	CAD
Total Lipids in Medium HDL	rs60847460	10	T	C	0.143	-0.038	0.005	9.40E-12	4.20E-04	48.334	CAD
Total Lipids in Medium HDL	rs61805076	1	C	T	0.334	-0.028	0.004	3.90E-12	4.07E-04	46.805	CAD
Total Lipids in Medium HDL	rs61941676	12	A	C	0.127	-0.052	0.006	1.60E-19	0.001	79.968	CAD
Total Lipids in Medium HDL	rs62101705	18	T	C	0.014	-0.090	0.017	2.60E-08	2.52E-04	29.052	CAD
Total Lipids in Medium HDL	rs6589565	11	G	A	0.932	0.078	0.008	4.70E-27	0.001	106.734	CAD
Total Lipids in Medium HDL	rs6606717	12	C	A	0.523	0.026	0.004	3.90E-12	4.06E-04	46.727	CAD
Total Lipids in Medium HDL	rs676210	2	A	G	0.206	0.077	0.005	1.10E-59	0.002	269.418	CAD
Total Lipids in Medium HDL	rs686030	9	A	C	0.860	0.038	0.005	3.60E-12	4.07E-04	46.857	CAD
Total Lipids in Medium HDL	rs6967917	7	G	A	0.492	0.022	0.004	1.00E-09	2.99E-04	34.422	CAD
Total Lipids in Medium HDL	rs7134375	12	A	C	0.430	0.021	0.004	8.80E-09	2.69E-04	30.953	CAD
Total Lipids in Medium HDL	rs72836561	17	T	C	0.031	-0.151	0.011	1.30E-44	0.002	189.949	CAD
Total Lipids in Medium HDL	rs737337	19	C	T	0.077	-0.067	0.007	5.70E-21	0.001	87.373	CAD

Total Lipids in Medium HDL	rs7388248	8	C	G	0.277	0.025	0.004	4.00E-09	3.01E-04	34.631	CAD
Total Lipids in Medium HDL	rs7534572	1	G	C	0.647	0.024	0.004	6.00E-10	3.09E-04	35.583	CAD
Total Lipids in Medium HDL	rs75911530	16	A	G	0.033	-0.154	0.011	4.20E-44	0.002	187.932	CAD
Total Lipids in Medium HDL	rs77960347	18	G	A	0.013	0.282	0.017	1.10E-66	0.002	286.767	CAD
Total Lipids in Medium HDL	rs7810507	7	A	G	0.281	-0.028	0.004	4.70E-11	3.83E-04	44.041	CAD
Total Lipids in Medium HDL	rs78296522	11	A	C	0.045	0.065	0.009	1.80E-12	4.28E-04	49.268	CAD
Total Lipids in Medium HDL	rs7956099	12	C	T	0.475	-0.020	0.004	4.30E-08	2.47E-04	28.389	CAD
Total Lipids in Medium HDL	rs7959043	12	G	A	0.408	0.022	0.004	1.30E-09	2.91E-04	33.468	CAD
Total Lipids in Medium HDL	rs8058512	16	T	C	0.724	-0.025	0.004	8.70E-09	2.94E-04	33.894	CAD
Total Lipids in Medium HDL	rs838876	12	G	A	0.681	-0.038	0.004	3.80E-22	0.001	83.323	CAD
Total Lipids in Medium HDL	rs904770	16	C	T	0.889	-0.035	0.006	8.60E-09	2.91E-04	33.493	CAD
Total Lipids in Medium HDL	rs9304381	18	T	C	0.818	0.085	0.005	2.60E-67	0.003	293.418	CAD
Cholesteryl Esters in HDL	rs10015477	4	A	G	0.368	-0.021	0.004	7.30E-09	2.08E-04	23.918	IS
Cholesteryl Esters in HDL	rs10162642	15	A	G	0.210	-0.033	0.005	3.20E-15	3.57E-04	41.068	IS
Cholesteryl Esters in HDL	rs10268632	7	C	A	0.512	0.023	0.004	5.60E-10	2.56E-04	29.421	IS
Cholesteryl Esters in HDL	rs1047891	2	A	C	0.315	-0.024	0.004	3.30E-09	2.54E-04	29.283	IS
Cholesteryl Esters in HDL	rs1054852	12	G	A	0.377	0.034	0.004	1.40E-18	0.001	61.666	IS
Cholesteryl Esters in HDL	rs11057692	12	G	A	0.236	-0.026	0.004	4.30E-10	2.43E-04	28.027	IS
Cholesteryl Esters in HDL	rs114165349	1	C	G	0.023	-0.114	0.012	2.90E-20	0.001	67.240	IS
Cholesteryl Esters in HDL	rs116978226	11	A	C	0.033	0.065	0.010	1.40E-10	2.69E-04	30.927	IS
Cholesteryl Esters in HDL	rs11789603	9	T	C	0.109	0.051	0.006	1.60E-16	0.001	58.252	IS
Cholesteryl Esters in HDL	rs1215112	9	A	G	0.863	0.047	0.005	1.50E-18	0.001	59.227	IS
Cholesteryl Esters in HDL	rs12453682	17	T	C	0.694	0.021	0.004	2.20E-08	1.91E-04	21.965	IS
Cholesteryl Esters in HDL	rs12533197	7	G	T	0.439	-0.022	0.004	4.30E-08	2.30E-04	26.510	IS
Cholesteryl Esters in HDL	rs12608026	18	G	T	0.042	0.070	0.009	1.80E-14	4.03E-04	46.450	IS
Cholesteryl Esters in HDL	rs12721046	19	A	G	0.158	-0.037	0.005	1.00E-13	3.55E-04	40.908	IS
Cholesteryl Esters in HDL	rs13107325	4	T	C	0.074	-0.075	0.007	2.60E-27	0.001	88.207	IS
Cholesteryl Esters in HDL	rs13389219	2	T	C	0.393	0.036	0.004	4.20E-22	0.001	70.267	IS

Cholesteryl Esters in HDL	rs1358980	6	T	C	0.483	-0.034	0.004	2.70E-19	0.001	64.650	IS
Cholesteryl Esters in HDL	rs145391587	8	C	A	0.100	0.165	0.006	3.00E-163	0.005	564.961	IS
Cholesteryl Esters in HDL	rs147233090	15	T	C	0.025	-0.085	0.012	5.00E-13	3.47E-04	39.943	IS
Cholesteryl Esters in HDL	rs150224153	20	T	C	0.029	-0.092	0.011	4.00E-16	4.85E-04	55.804	IS
Cholesteryl Esters in HDL	rs1601935	15	T	G	0.655	-0.083	0.004	4.50E-102	0.003	359.897	IS
Cholesteryl Esters in HDL	rs174578	11	A	T	0.351	-0.062	0.004	5.50E-62	0.002	201.767	IS
Cholesteryl Esters in HDL	rs17696736	12	G	A	0.430	-0.022	0.004	1.20E-10	2.30E-04	26.446	IS
Cholesteryl Esters in HDL	rs191555775	6	T	A	0.106	-0.056	0.006	5.90E-22	0.001	68.020	IS
Cholesteryl Esters in HDL	rs2066714	9	C	T	0.129	0.032	0.006	4.50E-10	2.37E-04	27.271	IS
Cholesteryl Esters in HDL	rs2070895	15	A	G	0.219	0.090	0.004	2.90E-94	0.003	318.161	IS
Cholesteryl Esters in HDL	rs2229357	12	A	G	0.241	0.031	0.004	2.50E-13	3.43E-04	39.513	IS
Cholesteryl Esters in HDL	rs2245221	8	A	G	0.559	0.030	0.004	7.00E-15	4.55E-04	52.434	IS
Cholesteryl Esters in HDL	rs2297402	9	T	C	0.024	-0.064	0.013	3.50E-08	1.91E-04	21.984	IS
Cholesteryl Esters in HDL	rs2298428	22	T	C	0.183	-0.036	0.005	1.00E-14	3.81E-04	43.882	IS
Cholesteryl Esters in HDL	rs2302263	11	T	C	0.088	-0.037	0.007	1.30E-08	2.22E-04	25.496	IS
Cholesteryl Esters in HDL	rs2307111	5	C	T	0.397	0.024	0.004	6.30E-10	2.69E-04	30.994	IS
Cholesteryl Esters in HDL	rs2494748	14	T	C	0.616	-0.021	0.004	6.20E-09	2.07E-04	23.829	IS
Cholesteryl Esters in HDL	rs2642438	1	G	A	0.704	0.035	0.004	6.80E-19	0.001	59.428	IS
Cholesteryl Esters in HDL	rs267738	1	G	T	0.220	0.028	0.004	3.30E-11	2.77E-04	31.916	IS
Cholesteryl Esters in HDL	rs2740488	9	C	A	0.265	-0.043	0.004	1.50E-25	0.001	82.266	IS
Cholesteryl Esters in HDL	rs2792735	10	A	G	0.720	-0.039	0.004	3.70E-23	0.001	71.970	IS
Cholesteryl Esters in HDL	rs28818616	3	C	T	0.350	-0.020	0.004	3.50E-08	1.87E-04	21.542	IS
Cholesteryl Esters in HDL	rs2925979	16	C	T	0.699	0.039	0.004	2.00E-24	0.001	75.399	IS
Cholesteryl Esters in HDL	rs2943650	2	T	C	0.645	-0.042	0.004	1.50E-29	0.001	91.964	IS
Cholesteryl Esters in HDL	rs2978615	19	T	C	0.490	-0.020	0.004	3.00E-08	2.05E-04	23.638	IS
Cholesteryl Esters in HDL	rs35184771	11	T	G	0.353	-0.039	0.004	2.40E-23	0.001	78.265	IS
Cholesteryl Esters in HDL	rs35493868	7	G	C	0.204	0.036	0.005	2.20E-15	4.18E-04	48.121	IS
Cholesteryl Esters in HDL	rs35633876	2	T	G	0.482	-0.023	0.004	6.30E-10	2.53E-04	29.104	IS

Cholesteryl Esters in HDL	rs3735687	7	G	A	0.423	-0.023	0.004	6.80E-09	2.49E-04	28.682	IS
Cholesteryl Esters in HDL	rs3764261	16	A	C	0.324	0.210	0.004	1.00E-200	0.019	2260.949	IS
Cholesteryl Esters in HDL	rs3768321	1	T	G	0.197	-0.044	0.005	5.30E-23	0.001	70.935	IS
Cholesteryl Esters in HDL	rs4078216	12	A	G	0.241	0.026	0.004	2.10E-08	2.48E-04	28.581	IS
Cholesteryl Esters in HDL	rs4240624	8	A	G	0.909	0.094	0.006	1.50E-49	0.001	169.656	IS
Cholesteryl Esters in HDL	rs4846921	1	A	G	0.613	0.050	0.004	4.60E-41	0.001	134.163	IS
Cholesteryl Esters in HDL	rs4922787	11	T	G	0.762	0.027	0.004	2.30E-10	2.67E-04	30.694	IS
Cholesteryl Esters in HDL	rs4969141	17	T	C	0.490	0.024	0.004	1.40E-10	2.84E-04	32.650	IS
Cholesteryl Esters in HDL	rs4986970	16	T	A	0.034	-0.092	0.010	2.60E-20	0.001	64.381	IS
Cholesteryl Esters in HDL	rs5167	19	G	T	0.352	0.044	0.004	4.40E-31	0.001	102.932	IS
Cholesteryl Esters in HDL	rs559355	11	T	A	0.158	-0.051	0.005	2.70E-23	0.001	78.786	IS
Cholesteryl Esters in HDL	rs59104589	2	T	C	0.359	0.020	0.004	1.00E-08	1.93E-04	22.188	IS
Cholesteryl Esters in HDL	rs59299606	16	A	G	0.249	-0.028	0.005	1.30E-09	2.90E-04	33.403	IS
Cholesteryl Esters in HDL	rs6018652	20	A	G	0.793	0.035	0.005	9.50E-16	4.06E-04	46.789	IS
Cholesteryl Esters in HDL	rs6073958	20	C	T	0.199	-0.067	0.005	6.90E-47	0.001	166.662	IS
Cholesteryl Esters in HDL	rs60847460	10	T	C	0.143	-0.036	0.005	7.40E-12	3.26E-04	37.498	IS
Cholesteryl Esters in HDL	rs61805076	1	C	T	0.334	-0.030	0.004	1.90E-14	3.93E-04	45.263	IS
Cholesteryl Esters in HDL	rs676210	2	A	G	0.206	0.072	0.005	1.70E-55	0.002	195.242	IS
Cholesteryl Esters in HDL	rs688671	18	G	A	0.267	-0.025	0.004	4.30E-09	2.40E-04	27.663	IS
Cholesteryl Esters in HDL	rs71336055	3	T	C	0.120	0.036	0.006	3.90E-10	2.68E-04	30.855	IS
Cholesteryl Esters in HDL	rs7134375	12	A	C	0.430	0.024	0.004	5.60E-11	2.82E-04	32.437	IS
Cholesteryl Esters in HDL	rs7136506	12	C	T	0.216	-0.040	0.005	9.00E-19	0.001	61.873	IS
Cholesteryl Esters in HDL	rs71562509	6	T	G	0.593	0.021	0.004	3.30E-08	2.08E-04	23.923	IS
Cholesteryl Esters in HDL	rs7241918	18	T	G	0.823	0.074	0.005	1.70E-54	0.002	186.282	IS
Cholesteryl Esters in HDL	rs72836561	17	T	C	0.031	-0.159	0.011	9.10E-54	0.002	178.241	IS
Cholesteryl Esters in HDL	rs7308864	12	G	A	0.523	0.025	0.004	3.80E-12	3.12E-04	35.944	IS
Cholesteryl Esters in HDL	rs737337	19	C	T	0.077	-0.059	0.007	1.20E-17	4.92E-04	56.593	IS
Cholesteryl Esters in HDL	rs75911530	16	A	G	0.033	-0.162	0.011	8.10E-52	0.002	192.236	IS

Cholesteryl Esters in HDL	rs77960347	18	G	A	0.013	0.248	0.016	4.30E-55	0.002	185.012	IS
Cholesteryl Esters in HDL	rs7810507	7	A	G	0.281	-0.032	0.004	1.50E-14	4.02E-04	46.240	IS
Cholesteryl Esters in HDL	rs7845090	8	A	G	0.709	0.024	0.004	1.40E-08	2.30E-04	26.525	IS
Cholesteryl Esters in HDL	rs8058512	16	T	C	0.724	-0.027	0.004	1.90E-10	2.86E-04	32.972	IS
Cholesteryl Esters in HDL	rs904770	16	C	T	0.889	-0.037	0.006	2.50E-10	2.72E-04	31.313	IS
Cholesteryl Esters in HDL	rs921919	12	A	G	0.670	-0.042	0.004	8.90E-28	0.001	87.858	IS
Cholesteryl Esters in HDL	rs9265113	6	T	C	0.408	-0.029	0.004	2.90E-13	4.06E-04	46.742	IS
Cholesteryl Esters in HDL	rs9491697	6	G	A	0.464	-0.028	0.004	7.10E-15	4.02E-04	46.290	IS
Cholesteryl Esters in HDL	rs964184	11	C	G	0.867	0.097	0.005	4.50E-74	0.002	250.549	IS
Cholesteryl Esters in Medium HDL	rs10184004	2	T	C	0.406	0.028	0.004	8.80E-14	3.86E-04	44.486	IS
Cholesteryl Esters in Medium HDL	rs1047891	2	A	C	0.315	-0.028	0.004	3.30E-11	3.33E-04	38.345	IS
Cholesteryl Esters in Medium HDL	rs1077835	15	G	A	0.220	0.061	0.005	3.70E-41	0.001	145.315	IS
Cholesteryl Esters in Medium HDL	rs11057692	12	G	A	0.236	-0.024	0.005	1.60E-08	2.06E-04	23.737	IS
Cholesteryl Esters in Medium HDL	rs114165349	1	C	G	0.023	-0.121	0.013	3.90E-22	0.001	75.705	IS
Cholesteryl Esters in Medium HDL	rs11591147	1	T	G	0.017	0.093	0.015	8.00E-11	2.94E-04	33.860	IS
Cholesteryl Esters in Medium HDL	rs11751347	6	T	C	0.102	-0.043	0.006	2.40E-12	3.32E-04	38.274	IS
Cholesteryl Esters in Medium HDL	rs117687565	18	T	C	0.012	0.117	0.018	7.30E-10	3.23E-04	37.151	IS
Cholesteryl Esters in Medium HDL	rs11789603	9	T	C	0.109	0.049	0.006	1.10E-14	4.63E-04	53.318	IS
Cholesteryl Esters in Medium HDL	rs12533197	7	G	T	0.439	-0.022	0.004	2.50E-08	2.38E-04	27.428	IS
Cholesteryl Esters in Medium HDL	rs1260326	2	C	T	0.604	-0.023	0.004	1.30E-08	2.52E-04	29.035	IS
Cholesteryl Esters in Medium HDL	rs12611067	19	T	G	0.351	0.025	0.004	1.60E-09	2.76E-04	31.808	IS
Cholesteryl Esters in Medium HDL	rs13107325	4	T	C	0.074	-0.071	0.007	6.70E-23	0.001	79.028	IS
Cholesteryl Esters in Medium HDL	rs1358980	6	T	C	0.483	-0.028	0.004	3.20E-13	3.87E-04	44.521	IS
Cholesteryl Esters in Medium HDL	rs139915535	8	G	A	0.018	-0.150	0.014	5.80E-27	0.001	92.016	IS
Cholesteryl Esters in Medium HDL	rs141368429	11	T	C	0.056	-0.055	0.009	1.00E-09	3.19E-04	36.731	IS
Cholesteryl Esters in Medium HDL	rs144311893	19	T	C	0.022	0.121	0.013	4.90E-21	0.001	73.740	IS
Cholesteryl Esters in Medium HDL	rs15285	8	T	C	0.287	0.105	0.004	5.40E-140	0.004	519.698	IS
Cholesteryl Esters in Medium HDL	rs1601935	15	T	G	0.655	-0.063	0.004	1.70E-55	0.002	204.227	IS

Cholesteryl Esters in Medium HDL	rs174578	11	A	T	0.351	-0.039	0.004	1.00E-23	0.001	79.018	IS
Cholesteryl Esters in Medium HDL	rs1800961	20	T	C	0.030	-0.123	0.011	4.80E-28	0.001	102.147	IS
Cholesteryl Esters in Medium HDL	rs2228671	19	T	C	0.126	0.040	0.006	2.70E-13	3.51E-04	40.349	IS
Cholesteryl Esters in Medium HDL	rs2229357	12	A	G	0.241	0.028	0.004	1.10E-10	2.89E-04	33.250	IS
Cholesteryl Esters in Medium HDL	rs2236252	20	T	C	0.167	-0.028	0.005	5.50E-09	2.24E-04	25.742	IS
Cholesteryl Esters in Medium HDL	rs2245221	8	A	G	0.559	0.032	0.004	1.60E-15	0.001	58.691	IS
Cholesteryl Esters in Medium HDL	rs2298428	22	T	C	0.183	-0.039	0.005	3.50E-16	4.63E-04	53.329	IS
Cholesteryl Esters in Medium HDL	rs2395943	6	G	A	0.588	0.024	0.004	1.70E-09	2.71E-04	31.173	IS
Cholesteryl Esters in Medium HDL	rs2494748	14	T	C	0.616	-0.022	0.004	3.30E-09	2.33E-04	26.807	IS
Cholesteryl Esters in Medium HDL	rs2642438	1	G	A	0.704	0.035	0.004	9.70E-18	0.001	58.782	IS
Cholesteryl Esters in Medium HDL	rs267738	1	G	T	0.220	0.039	0.005	1.80E-18	0.001	60.329	IS
Cholesteryl Esters in Medium HDL	rs2740488	9	C	A	0.265	-0.038	0.004	5.30E-19	0.001	63.300	IS
Cholesteryl Esters in Medium HDL	rs2792735	10	A	G	0.720	-0.038	0.004	2.00E-20	0.001	66.589	IS
Cholesteryl Esters in Medium HDL	rs28746853	6	C	T	0.176	0.030	0.005	4.00E-08	2.66E-04	30.643	IS
Cholesteryl Esters in Medium HDL	rs28818616	3	C	T	0.350	-0.022	0.004	2.30E-08	2.11E-04	24.249	IS
Cholesteryl Esters in Medium HDL	rs2925979	16	C	T	0.699	0.035	0.004	8.20E-19	0.001	59.154	IS
Cholesteryl Esters in Medium HDL	rs2943650	2	T	C	0.645	-0.034	0.004	1.70E-19	0.001	61.770	IS
Cholesteryl Esters in Medium HDL	rs35135293	2	T	C	0.517	-0.023	0.004	6.70E-10	2.66E-04	30.587	IS
Cholesteryl Esters in Medium HDL	rs35184771	11	T	G	0.353	-0.036	0.004	1.90E-19	0.001	68.980	IS
Cholesteryl Esters in Medium HDL	rs36057735	6	G	C	0.199	-0.033	0.005	3.20E-12	3.48E-04	40.052	IS
Cholesteryl Esters in Medium HDL	rs3768321	1	T	G	0.197	-0.044	0.005	9.30E-21	0.001	69.036	IS
Cholesteryl Esters in Medium HDL	rs3795269	1	A	C	0.439	0.021	0.004	3.30E-08	2.12E-04	24.402	IS
Cholesteryl Esters in Medium HDL	rs4239651	20	C	T	0.794	0.035	0.005	4.70E-15	4.05E-04	46.668	IS
Cholesteryl Esters in Medium HDL	rs4240624	8	A	G	0.909	0.103	0.007	4.40E-55	0.002	201.609	IS
Cholesteryl Esters in Medium HDL	rs429358	19	C	T	0.155	-0.079	0.005	4.20E-54	0.002	189.535	IS
Cholesteryl Esters in Medium HDL	rs4656292	1	G	A	0.620	0.021	0.004	3.90E-09	2.12E-04	24.444	IS
Cholesteryl Esters in Medium HDL	rs4846921	1	A	G	0.613	0.052	0.004	1.10E-42	0.001	150.594	IS
Cholesteryl Esters in Medium HDL	rs4969141	17	T	C	0.490	0.025	0.004	5.50E-11	3.03E-04	34.916	IS

Cholesteryl Esters in Medium HDL	rs4986970	16	T	A	0.034	-0.076	0.011	2.20E-13	3.79E-04	43.637	IS
Cholesteryl Esters in Medium HDL	rs559355	11	T	A	0.158	-0.063	0.005	7.60E-34	0.001	121.296	IS
Cholesteryl Esters in Medium HDL	rs583104	1	T	G	0.773	-0.042	0.005	5.60E-21	0.001	70.293	IS
Cholesteryl Esters in Medium HDL	rs59097294	11	C	T	0.181	0.064	0.005	3.60E-41	0.001	141.637	IS
Cholesteryl Esters in Medium HDL	rs59347135	8	G	C	0.046	-0.079	0.009	7.00E-18	0.001	63.167	IS
Cholesteryl Esters in Medium HDL	rs60847460	10	T	C	0.143	-0.038	0.005	9.40E-12	3.54E-04	40.806	IS
Cholesteryl Esters in Medium HDL	rs61805076	1	C	T	0.334	-0.028	0.004	3.90E-12	3.40E-04	39.112	IS
Cholesteryl Esters in Medium HDL	rs61941676	12	A	C	0.127	-0.052	0.006	1.60E-19	0.001	70.376	IS
Cholesteryl Esters in Medium HDL	rs6589565	11	G	A	0.932	0.078	0.008	4.70E-27	0.001	89.714	IS
Cholesteryl Esters in Medium HDL	rs6606717	12	C	A	0.523	0.026	0.004	3.90E-12	3.39E-04	38.977	IS
Cholesteryl Esters in Medium HDL	rs676210	2	A	G	0.206	0.077	0.005	1.10E-59	0.002	224.637	IS
Cholesteryl Esters in Medium HDL	rs686030	9	A	C	0.860	0.038	0.005	3.60E-12	3.42E-04	39.313	IS
Cholesteryl Esters in Medium HDL	rs6967917	7	G	A	0.492	0.022	0.004	1.00E-09	2.51E-04	28.940	IS
Cholesteryl Esters in Medium HDL	rs7134375	12	A	C	0.430	0.021	0.004	8.80E-09	2.24E-04	25.833	IS
Cholesteryl Esters in Medium HDL	rs72836561	17	T	C	0.031	-0.151	0.011	1.30E-44	0.001	159.303	IS
Cholesteryl Esters in Medium HDL	rs737337	19	C	T	0.077	-0.067	0.007	5.70E-21	0.001	73.102	IS
Cholesteryl Esters in Medium HDL	rs7534572	1	G	C	0.647	0.024	0.004	6.00E-10	2.58E-04	29.716	IS
Cholesteryl Esters in Medium HDL	rs75911530	16	A	G	0.033	-0.154	0.011	4.20E-44	0.002	173.487	IS
Cholesteryl Esters in Medium HDL	rs77960347	18	G	A	0.013	0.282	0.017	1.10E-66	0.002	239.808	IS
Cholesteryl Esters in Medium HDL	rs7810507	7	A	G	0.281	-0.028	0.004	4.70E-11	3.20E-04	36.841	IS
Cholesteryl Esters in Medium HDL	rs78296522	11	A	C	0.045	0.065	0.009	1.80E-12	3.62E-04	41.706	IS
Cholesteryl Esters in Medium HDL	rs7956099	12	C	T	0.475	-0.020	0.004	4.30E-08	2.09E-04	24.013	IS
Cholesteryl Esters in Medium HDL	rs7959043	12	G	A	0.408	0.022	0.004	1.30E-09	2.44E-04	28.080	IS
Cholesteryl Esters in Medium HDL	rs8058512	16	T	C	0.724	-0.025	0.004	8.70E-09	2.47E-04	28.442	IS
Cholesteryl Esters in Medium HDL	rs838876	12	G	A	0.681	-0.038	0.004	3.80E-22	0.001	71.771	IS
Cholesteryl Esters in Medium HDL	rs904770	16	C	T	0.889	-0.035	0.006	8.60E-09	2.43E-04	27.973	IS
Cholesteryl Esters in Medium HDL	rs9304381	18	T	C	0.818	0.085	0.005	2.60E-67	0.002	246.631	IS
Cholesteryl Esters in Medium HDL	rs9491697	6	G	A	0.464	-0.026	0.004	1.40E-11	3.40E-04	39.088	IS

Cholesteryl Esters in Medium HDL	rs9647335	3	T	A	0.192	0.033	0.005	1.20E-11	3.38E-04	38.875	IS
Cholesteryl Esters in Medium HDL	rs967645	17	T	C	0.517	-0.026	0.004	4.50E-11	3.40E-04	39.117	IS
Cholesteryl Esters in Medium HDL	rs9687846	5	A	G	0.201	-0.032	0.005	9.00E-12	3.34E-04	38.500	IS
Total Cholesterol in Medium HDL	rs10162642	15	A	G	0.210	-0.027	0.005	3.90E-10	2.34E-04	26.981	IS
Total Cholesterol in Medium HDL	rs10184004	2	T	C	0.406	0.027	0.004	3.90E-13	3.61E-04	41.565	IS
Total Cholesterol in Medium HDL	rs1047891	2	A	C	0.315	-0.027	0.004	1.00E-10	3.15E-04	36.308	IS
Total Cholesterol in Medium HDL	rs1054852	12	G	A	0.377	0.032	0.004	1.20E-16	4.91E-04	56.513	IS
Total Cholesterol in Medium HDL	rs1077835	15	G	A	0.220	0.073	0.005	6.70E-60	0.002	213.075	IS
Total Cholesterol in Medium HDL	rs11057692	12	G	A	0.236	-0.024	0.005	1.70E-08	2.04E-04	23.452	IS
Total Cholesterol in Medium HDL	rs114165349	1	C	G	0.023	-0.117	0.013	8.60E-21	0.001	70.148	IS
Total Cholesterol in Medium HDL	rs11751347	6	T	C	0.102	-0.041	0.006	7.80E-12	3.16E-04	36.342	IS
Total Cholesterol in Medium HDL	rs11789603	9	T	C	0.109	0.050	0.006	1.20E-15	4.93E-04	56.711	IS
Total Cholesterol in Medium HDL	rs12533197	7	G	T	0.439	-0.022	0.004	2.50E-08	2.37E-04	27.254	IS
Total Cholesterol in Medium HDL	rs1260326	2	C	T	0.604	-0.025	0.004	4.60E-10	3.00E-04	34.499	IS
Total Cholesterol in Medium HDL	rs12608026	18	G	T	0.042	0.083	0.010	6.20E-19	0.001	65.080	IS
Total Cholesterol in Medium HDL	rs13107325	4	T	C	0.074	-0.071	0.007	2.30E-23	0.001	79.543	IS
Total Cholesterol in Medium HDL	rs1358980	6	T	C	0.483	-0.027	0.004	1.40E-12	3.67E-04	42.271	IS
Total Cholesterol in Medium HDL	rs139915535	8	G	A	0.018	-0.146	0.014	5.60E-26	0.001	87.265	IS
Total Cholesterol in Medium HDL	rs141368429	11	T	C	0.056	-0.054	0.009	1.50E-09	3.12E-04	35.920	IS
Total Cholesterol in Medium HDL	rs144311893	19	T	C	0.022	0.112	0.013	1.50E-18	0.001	63.552	IS
Total Cholesterol in Medium HDL	rs15285	8	T	C	0.287	0.102	0.004	7.40E-133	0.004	490.697	IS
Total Cholesterol in Medium HDL	rs1601935	15	T	G	0.655	-0.073	0.004	1.00E-75	0.002	279.243	IS
Total Cholesterol in Medium HDL	rs174578	11	A	T	0.351	-0.042	0.004	2.40E-27	0.001	91.223	IS
Total Cholesterol in Medium HDL	rs1800961	20	T	C	0.030	-0.123	0.011	3.90E-28	0.001	101.821	IS
Total Cholesterol in Medium HDL	rs2229357	12	A	G	0.241	0.027	0.004	2.80E-10	2.71E-04	31.214	IS
Total Cholesterol in Medium HDL	rs2236252	20	T	C	0.167	-0.029	0.005	4.30E-09	2.27E-04	26.183	IS
Total Cholesterol in Medium HDL	rs2245221	8	A	G	0.559	0.033	0.004	4.60E-16	0.001	60.138	IS
Total Cholesterol in Medium HDL	rs2298428	22	T	C	0.183	-0.040	0.005	1.20E-16	4.73E-04	54.459	IS

Total Cholesterol in Medium HDL	rs2395943	6	G	A	0.588	0.023	0.004	2.90E-09	2.60E-04	29.963	IS
Total Cholesterol in Medium HDL	rs2494748	14	T	C	0.616	-0.022	0.004	4.60E-09	2.25E-04	25.866	IS
Total Cholesterol in Medium HDL	rs2642438	1	G	A	0.704	0.036	0.004	1.80E-18	0.001	60.858	IS
Total Cholesterol in Medium HDL	rs267738	1	G	T	0.220	0.038	0.005	6.20E-18	0.001	57.670	IS
Total Cholesterol in Medium HDL	rs2740488	9	C	A	0.265	-0.039	0.004	2.60E-20	0.001	67.732	IS
Total Cholesterol in Medium HDL	rs2792735	10	A	G	0.720	-0.039	0.004	2.20E-21	0.001	69.700	IS
Total Cholesterol in Medium HDL	rs28746853	6	C	T	0.176	0.030	0.005	4.80E-08	2.64E-04	30.390	IS
Total Cholesterol in Medium HDL	rs28818616	3	C	T	0.350	-0.022	0.004	2.30E-08	2.11E-04	24.277	IS
Total Cholesterol in Medium HDL	rs2925979	16	C	T	0.699	0.034	0.004	3.20E-18	4.93E-04	56.785	IS
Total Cholesterol in Medium HDL	rs2943650	2	T	C	0.645	-0.034	0.004	5.40E-19	0.001	59.492	IS
Total Cholesterol in Medium HDL	rs2978615	19	T	C	0.490	-0.025	0.004	2.80E-11	3.15E-04	36.306	IS
Total Cholesterol in Medium HDL	rs35135293	2	T	C	0.517	-0.024	0.004	9.90E-11	2.90E-04	33.355	IS
Total Cholesterol in Medium HDL	rs35184771	11	T	G	0.353	-0.036	0.004	7.10E-20	0.001	69.986	IS
Total Cholesterol in Medium HDL	rs36057735	6	G	C	0.199	-0.034	0.005	1.00E-12	3.62E-04	41.643	IS
Total Cholesterol in Medium HDL	rs3764261	16	A	C	0.324	0.184	0.004	1.00E-200	0.015	1742.547	IS
Total Cholesterol in Medium HDL	rs3768321	1	T	G	0.197	-0.043	0.005	2.50E-20	0.001	66.730	IS
Total Cholesterol in Medium HDL	rs3795269	1	A	C	0.439	0.020	0.004	4.80E-08	2.06E-04	23.656	IS
Total Cholesterol in Medium HDL	rs4239651	20	C	T	0.794	0.036	0.005	1.90E-15	4.15E-04	47.819	IS
Total Cholesterol in Medium HDL	rs4240624	8	A	G	0.909	0.103	0.007	4.20E-55	0.002	200.515	IS
Total Cholesterol in Medium HDL	rs429358	19	C	T	0.155	-0.075	0.005	8.60E-49	0.001	169.366	IS
Total Cholesterol in Medium HDL	rs4656292	1	G	A	0.620	0.020	0.004	2.90E-08	1.85E-04	21.285	IS
Total Cholesterol in Medium HDL	rs4846921	1	A	G	0.613	0.052	0.004	1.60E-42	0.001	148.591	IS
Total Cholesterol in Medium HDL	rs4969141	17	T	C	0.490	0.024	0.004	1.50E-10	2.89E-04	33.220	IS
Total Cholesterol in Medium HDL	rs4986970	16	T	A	0.034	-0.076	0.011	1.40E-13	3.81E-04	43.888	IS
Total Cholesterol in Medium HDL	rs559355	11	T	A	0.158	-0.063	0.005	1.80E-34	0.001	122.680	IS
Total Cholesterol in Medium HDL	rs583104	1	T	G	0.773	-0.040	0.005	3.40E-19	0.001	62.991	IS
Total Cholesterol in Medium HDL	rs59097294	11	C	T	0.181	0.065	0.005	1.00E-42	0.001	146.053	IS
Total Cholesterol in Medium HDL	rs59347135	8	G	C	0.046	-0.077	0.009	3.90E-17	0.001	60.015	IS

Total Cholesterol in Medium HDL	rs60847460	10	T	C	0.143	-0.038	0.005	5.20E-12	3.58E-04	41.238	IS
Total Cholesterol in Medium HDL	rs61805076	1	C	T	0.334	-0.028	0.004	2.50E-12	3.43E-04	39.464	IS
Total Cholesterol in Medium HDL	rs638714	1	T	G	0.346	-0.028	0.004	6.40E-13	3.59E-04	41.278	IS
Total Cholesterol in Medium HDL	rs6589565	11	G	A	0.932	0.072	0.008	1.30E-23	0.001	76.426	IS
Total Cholesterol in Medium HDL	rs6606717	12	C	A	0.523	0.027	0.004	4.60E-13	3.64E-04	41.919	IS
Total Cholesterol in Medium HDL	rs676210	2	A	G	0.206	0.075	0.005	8.80E-57	0.002	212.223	IS
Total Cholesterol in Medium HDL	rs686030	9	A	C	0.860	0.039	0.005	4.10E-13	3.69E-04	42.474	IS
Total Cholesterol in Medium HDL	rs6967917	7	G	A	0.492	0.022	0.004	2.60E-09	2.39E-04	27.488	IS
Total Cholesterol in Medium HDL	rs7134375	12	A	C	0.430	0.021	0.004	1.30E-08	2.15E-04	24.801	IS
Total Cholesterol in Medium HDL	rs7136506	12	C	T	0.216	-0.038	0.005	1.80E-16	4.82E-04	55.436	IS
Total Cholesterol in Medium HDL	rs7241918	18	T	G	0.823	0.087	0.005	6.60E-70	0.002	255.800	IS
Total Cholesterol in Medium HDL	rs72836561	17	T	C	0.031	-0.148	0.011	8.70E-44	0.001	154.738	IS
Total Cholesterol in Medium HDL	rs737337	19	C	T	0.077	-0.068	0.007	1.80E-21	0.001	74.730	IS
Total Cholesterol in Medium HDL	rs75911530	16	A	G	0.033	-0.153	0.011	5.50E-44	0.001	171.097	IS
Total Cholesterol in Medium HDL	rs77960347	18	G	A	0.013	0.288	0.017	6.50E-70	0.002	249.999	IS
Total Cholesterol in Medium HDL	rs7810507	7	A	G	0.281	-0.027	0.004	1.30E-10	3.00E-04	34.540	IS
Total Cholesterol in Medium HDL	rs78296522	11	A	C	0.045	0.065	0.009	2.40E-12	3.56E-04	40.973	IS
Total Cholesterol in Medium HDL	rs7956099	12	C	T	0.475	-0.021	0.004	1.80E-08	2.18E-04	25.123	IS
Total Cholesterol in Medium HDL	rs8058512	16	T	C	0.724	-0.025	0.004	5.60E-09	2.50E-04	28.793	IS
Total Cholesterol in Medium HDL	rs904770	16	C	T	0.889	-0.035	0.006	1.10E-08	2.38E-04	27.397	IS
Total Cholesterol in Medium HDL	rs921919	12	A	G	0.670	-0.038	0.004	2.20E-22	0.001	74.347	IS
Total Cholesterol in Medium HDL	rs9491697	6	G	A	0.464	-0.025	0.004	2.90E-11	3.23E-04	37.224	IS
Total Cholesterol in Medium HDL	rs9647335	3	T	A	0.192	0.031	0.005	2.20E-10	2.96E-04	34.068	IS
Total Cholesterol in Medium HDL	rs967645	17	T	C	0.517	-0.026	0.004	2.40E-11	3.48E-04	40.042	IS
Total Cholesterol in Medium HDL	rs9687846	5	A	G	0.201	-0.031	0.005	3.50E-11	3.11E-04	35.809	IS
Total Concentration of Medium HDL Particles	rs10184004	2	T	C	0.406	0.021	0.004	1.00E-08	2.21E-04	25.473	IS
Total Concentration of Medium HDL Particles	rs1047891	2	A	C	0.315	-0.026	0.004	5.20E-10	2.88E-04	33.145	IS
Total Concentration of Medium HDL Particles	rs1077835	15	G	A	0.220	0.103	0.005	2.10E-115	0.004	418.904	IS

Total Concentration of Medium HDL Particles	rs112495680	6	G	A	0.133	-0.035	0.006	2.10E-10	2.87E-04	33.018	IS
Total Concentration of Medium HDL Particles	rs112771035	11	G	C	0.071	-0.042	0.007	4.10E-08	2.37E-04	27.256	IS
Total Concentration of Medium HDL Particles	rs117687565	18	T	C	0.012	0.133	0.018	7.70E-13	4.21E-04	48.411	IS
Total Concentration of Medium HDL Particles	rs11789603	9	T	C	0.109	0.054	0.006	6.00E-18	0.001	65.480	IS
Total Concentration of Medium HDL Particles	rs1260326	2	C	T	0.604	-0.039	0.004	4.30E-22	0.001	81.841	IS
Total Concentration of Medium HDL Particles	rs12904367	15	A	G	0.141	0.031	0.006	3.70E-08	2.27E-04	26.103	IS
Total Concentration of Medium HDL Particles	rs12976739	19	A	G	0.396	0.026	0.004	5.80E-12	3.18E-04	36.656	IS
Total Concentration of Medium HDL Particles	rs13107325	4	T	C	0.074	-0.068	0.007	7.00E-22	0.001	73.358	IS
Total Concentration of Medium HDL Particles	rs1321257	1	A	G	0.613	0.051	0.004	5.60E-40	0.001	140.627	IS
Total Concentration of Medium HDL Particles	rs139915535	8	G	A	0.018	-0.128	0.014	1.60E-20	0.001	66.699	IS
Total Concentration of Medium HDL Particles	rs15285	8	T	C	0.287	0.086	0.004	8.00E-96	0.003	352.021	IS
Total Concentration of Medium HDL Particles	rs174578	11	A	T	0.351	-0.035	0.004	4.20E-20	0.001	64.773	IS
Total Concentration of Medium HDL Particles	rs1800961	20	T	C	0.030	-0.119	0.011	3.20E-26	0.001	95.305	IS
Total Concentration of Medium HDL Particles	rs193084249	1	G	A	0.023	-0.105	0.013	1.90E-16	0.001	58.029	IS
Total Concentration of Medium HDL Particles	rs2043085	15	C	T	0.613	-0.097	0.004	5.00E-141	0.005	520.731	IS
Total Concentration of Medium HDL Particles	rs2066714	9	C	T	0.129	0.032	0.006	4.30E-09	2.28E-04	26.285	IS
Total Concentration of Medium HDL Particles	rs2176040	2	G	A	0.647	-0.029	0.004	1.20E-14	3.76E-04	43.238	IS
Total Concentration of Medium HDL Particles	rs2229357	12	A	G	0.241	0.024	0.004	4.50E-08	2.05E-04	23.598	IS
Total Concentration of Medium HDL Particles	rs2236252	20	T	C	0.167	-0.032	0.005	1.20E-10	2.77E-04	31.933	IS
Total Concentration of Medium HDL Particles	rs2245221	8	A	G	0.559	0.034	0.004	7.90E-18	0.001	65.763	IS
Total Concentration of Medium HDL Particles	rs2298428	22	T	C	0.183	-0.041	0.005	8.50E-18	0.001	59.036	IS
Total Concentration of Medium HDL Particles	rs235314	21	T	C	0.533	-0.021	0.004	3.50E-08	2.26E-04	25.975	IS
Total Concentration of Medium HDL Particles	rs2494748	14	T	C	0.616	-0.022	0.004	4.20E-09	2.28E-04	26.239	IS
Total Concentration of Medium HDL Particles	rs2642438	1	G	A	0.704	0.035	0.004	3.40E-18	0.001	59.933	IS
Total Concentration of Medium HDL Particles	rs267738	1	G	T	0.220	0.037	0.005	8.10E-17	4.66E-04	53.595	IS
Total Concentration of Medium HDL Particles	rs2740488	9	C	A	0.265	-0.043	0.004	1.00E-24	0.001	83.639	IS
Total Concentration of Medium HDL Particles	rs2792735	10	A	G	0.720	-0.040	0.004	5.80E-22	0.001	72.807	IS
Total Concentration of Medium HDL Particles	rs28818616	3	C	T	0.350	-0.022	0.004	3.00E-08	2.16E-04	24.908	IS

Total Concentration of Medium HDL Particles	rs2925979	16	C	T	0.699	0.030	0.004	1.80E-14	3.80E-04	43.706	IS
Total Concentration of Medium HDL Particles	rs35135293	2	T	C	0.517	-0.028	0.004	1.30E-13	3.79E-04	43.573	IS
Total Concentration of Medium HDL Particles	rs35184771	11	T	G	0.353	-0.036	0.004	4.70E-19	0.001	67.234	IS
Total Concentration of Medium HDL Particles	rs36057735	6	G	C	0.199	-0.035	0.005	1.30E-13	3.99E-04	45.957	IS
Total Concentration of Medium HDL Particles	rs3764261	16	A	C	0.324	0.170	0.004	1.00E-200	0.013	1475.717	IS
Total Concentration of Medium HDL Particles	rs3768321	1	T	G	0.197	-0.040	0.005	3.70E-18	0.001	59.003	IS
Total Concentration of Medium HDL Particles	rs3795269	1	A	C	0.439	0.021	0.004	3.20E-08	2.14E-04	24.639	IS
Total Concentration of Medium HDL Particles	rs3859588	20	A	T	0.217	-0.026	0.005	3.70E-08	2.27E-04	26.185	IS
Total Concentration of Medium HDL Particles	rs4239651	20	C	T	0.794	0.037	0.005	5.50E-16	4.40E-04	50.699	IS
Total Concentration of Medium HDL Particles	rs4240624	8	A	G	0.909	0.101	0.007	1.00E-52	0.002	192.328	IS
Total Concentration of Medium HDL Particles	rs4969141	17	T	C	0.490	0.022	0.004	8.00E-09	2.32E-04	26.724	IS
Total Concentration of Medium HDL Particles	rs4986970	16	T	A	0.034	-0.067	0.011	3.10E-11	2.94E-04	33.847	IS
Total Concentration of Medium HDL Particles	rs559355	11	T	A	0.158	-0.066	0.005	2.00E-37	0.001	134.200	IS
Total Concentration of Medium HDL Particles	rs583104	1	T	G	0.773	-0.045	0.005	6.30E-24	0.001	80.036	IS
Total Concentration of Medium HDL Particles	rs59347135	8	G	C	0.046	-0.068	0.009	2.70E-13	4.05E-04	46.606	IS
Total Concentration of Medium HDL Particles	rs60847460	10	T	C	0.143	-0.038	0.005	6.30E-12	3.54E-04	40.803	IS
Total Concentration of Medium HDL Particles	rs61805076	1	C	T	0.334	-0.027	0.004	1.90E-11	3.14E-04	36.130	IS
Total Concentration of Medium HDL Particles	rs61941676	12	A	C	0.127	-0.055	0.006	6.70E-21	0.001	76.512	IS
Total Concentration of Medium HDL Particles	rs62101704	18	A	G	0.014	-0.090	0.017	1.80E-08	2.21E-04	25.409	IS
Total Concentration of Medium HDL Particles	rs625145	11	T	A	0.188	0.071	0.005	1.90E-51	0.002	178.120	IS
Total Concentration of Medium HDL Particles	rs638714	1	T	G	0.346	-0.046	0.004	3.40E-31	0.001	109.900	IS
Total Concentration of Medium HDL Particles	rs6606717	12	C	A	0.523	0.030	0.004	2.00E-15	4.46E-04	51.402	IS
Total Concentration of Medium HDL Particles	rs676210	2	A	G	0.206	0.062	0.005	8.50E-39	0.001	144.038	IS
Total Concentration of Medium HDL Particles	rs686030	9	A	C	0.860	0.038	0.005	1.20E-12	3.52E-04	40.490	IS
Total Concentration of Medium HDL Particles	rs72836561	17	T	C	0.031	-0.131	0.011	1.50E-34	0.001	119.967	IS
Total Concentration of Medium HDL Particles	rs737338	19	T	C	0.035	-0.120	0.010	5.90E-33	0.001	113.311	IS
Total Concentration of Medium HDL Particles	rs75911530	16	A	G	0.033	-0.140	0.011	2.00E-37	0.001	143.640	IS
Total Concentration of Medium HDL Particles	rs76213248	19	T	C	0.410	0.031	0.004	6.80E-15	4.52E-04	52.050	IS

Total Concentration of Medium HDL Particles	rs7783857	7	G	C	0.281	-0.024	0.004	1.50E-08	2.34E-04	26.915	IS
Total Concentration of Medium HDL Particles	rs77960347	18	G	A	0.013	0.322	0.017	4.90E-86	0.003	311.895	IS
Total Concentration of Medium HDL Particles	rs7956099	12	C	T	0.475	-0.023	0.004	6.60E-10	2.68E-04	30.861	IS
Total Concentration of Medium HDL Particles	rs7959043	12	G	A	0.408	0.021	0.004	2.30E-08	2.12E-04	24.425	IS
Total Concentration of Medium HDL Particles	rs8058512	16	T	C	0.724	-0.024	0.004	3.30E-08	2.24E-04	25.810	IS
Total Concentration of Medium HDL Particles	rs838876	12	G	A	0.681	-0.038	0.004	1.90E-21	0.001	70.696	IS
Total Concentration of Medium HDL Particles	rs9304381	18	T	C	0.818	0.096	0.005	5.00E-85	0.003	312.809	IS
Total Concentration of Medium HDL Particles	rs9471972	6	A	G	0.536	0.026	0.004	1.00E-11	3.40E-04	39.168	IS
Total Concentration of Medium HDL Particles	rs9491697	6	G	A	0.464	-0.022	0.004	1.00E-08	2.42E-04	27.864	IS
Total Concentration of Medium HDL Particles	rs967645	17	T	C	0.517	-0.030	0.004	8.00E-14	4.35E-04	50.111	IS
Total Lipids in HDL	rs10184004	2	T	C	0.406	0.025	0.004	1.80E-12	3.11E-04	35.783	IS
Total Lipids in HDL	rs11045171	12	G	A	0.197	0.026	0.005	9.40E-09	2.11E-04	24.237	IS
Total Lipids in HDL	rs11057692	12	G	A	0.236	-0.023	0.004	1.80E-08	1.94E-04	22.367	IS
Total Lipids in HDL	rs11751347	6	T	C	0.102	-0.037	0.006	4.30E-10	2.58E-04	29.653	IS
Total Lipids in HDL	rs11789603	9	T	C	0.109	0.055	0.006	3.50E-19	0.001	67.696	IS
Total Lipids in HDL	rs12608026	18	G	T	0.042	0.087	0.009	8.50E-22	0.001	71.381	IS
Total Lipids in HDL	rs12721046	19	A	G	0.158	-0.050	0.005	4.20E-23	0.001	76.247	IS
Total Lipids in HDL	rs13107325	4	T	C	0.074	-0.071	0.007	1.60E-25	0.001	80.541	IS
Total Lipids in HDL	rs1358980	6	T	C	0.483	-0.026	0.004	5.70E-12	3.42E-04	39.339	IS
Total Lipids in HDL	rs139915535	8	G	A	0.018	-0.127	0.014	2.10E-21	0.001	65.581	IS
Total Lipids in HDL	rs141368429	11	T	C	0.056	-0.050	0.009	6.40E-09	2.69E-04	30.953	IS
Total Lipids in HDL	rs147627829	6	A	G	0.044	-0.058	0.009	2.30E-10	2.84E-04	32.702	IS
Total Lipids in HDL	rs150224153	20	T	C	0.029	-0.083	0.011	5.30E-13	3.90E-04	44.852	IS
Total Lipids in HDL	rs15285	8	T	C	0.287	0.086	0.004	7.60E-100	0.003	349.829	IS
Total Lipids in HDL	rs174574	11	C	A	0.649	0.054	0.004	4.20E-47	0.001	152.054	IS
Total Lipids in HDL	rs17696736	12	G	A	0.430	-0.019	0.004	9.10E-09	1.82E-04	20.915	IS
Total Lipids in HDL	rs193084249	1	G	A	0.023	-0.096	0.013	2.10E-14	4.21E-04	48.466	IS
Total Lipids in HDL	rs2043085	15	C	T	0.613	-0.125	0.004	1.00E-200	0.007	858.335	IS

Total Lipids in HDL	rs2066714	9	C	T	0.129	0.034	0.006	1.80E-10	2.60E-04	29.888	IS
Total Lipids in HDL	rs2070895	15	A	G	0.219	0.139	0.004	1.00E-200	0.007	762.690	IS
Total Lipids in HDL	rs2176040	2	G	A	0.647	-0.033	0.004	4.70E-19	4.85E-04	55.785	IS
Total Lipids in HDL	rs2229357	12	A	G	0.241	0.024	0.004	9.80E-09	2.10E-04	24.170	IS
Total Lipids in HDL	rs2236252	20	T	C	0.167	-0.028	0.005	5.90E-09	2.20E-04	25.293	IS
Total Lipids in HDL	rs2243616	12	T	G	0.661	-0.022	0.004	2.30E-08	2.24E-04	25.761	IS
Total Lipids in HDL	rs2245221	8	A	G	0.559	0.034	0.004	1.50E-18	0.001	64.846	IS
Total Lipids in HDL	rs2298428	22	T	C	0.183	-0.039	0.005	9.00E-17	4.53E-04	52.135	IS
Total Lipids in HDL	rs2642438	1	G	A	0.704	0.036	0.004	4.20E-20	0.001	61.908	IS
Total Lipids in HDL	rs267738	1	G	T	0.220	0.029	0.004	9.00E-12	2.95E-04	33.919	IS
Total Lipids in HDL	rs2740488	9	C	A	0.265	-0.046	0.004	1.20E-28	0.001	93.639	IS
Total Lipids in HDL	rs2792735	10	A	G	0.720	-0.041	0.004	9.30E-25	0.001	79.204	IS
Total Lipids in HDL	rs28818616	3	C	T	0.350	-0.021	0.004	3.00E-08	2.04E-04	23.521	IS
Total Lipids in HDL	rs2925979	16	C	T	0.699	0.032	0.004	3.20E-17	4.43E-04	50.948	IS
Total Lipids in HDL	rs2978615	19	T	C	0.490	-0.023	0.004	9.80E-10	2.54E-04	29.187	IS
Total Lipids in HDL	rs34663616	15	A	C	0.138	0.042	0.005	7.60E-15	4.10E-04	47.212	IS
Total Lipids in HDL	rs34955778	16	C	T	0.420	-0.022	0.004	4.60E-09	2.28E-04	26.258	IS
Total Lipids in HDL	rs35184771	11	T	G	0.353	-0.037	0.004	9.40E-22	0.001	72.666	IS
Total Lipids in HDL	rs36057735	6	G	C	0.199	-0.035	0.005	6.20E-14	3.80E-04	43.689	IS
Total Lipids in HDL	rs3735687	7	G	A	0.423	-0.027	0.004	6.30E-12	3.43E-04	39.525	IS
Total Lipids in HDL	rs3764261	16	A	C	0.324	0.190	0.004	1.00E-200	0.016	1849.165	IS
Total Lipids in HDL	rs3768321	1	T	G	0.197	-0.039	0.005	1.10E-17	4.69E-04	54.049	IS
Total Lipids in HDL	rs41272663	2	A	C	0.264	0.024	0.004	6.50E-09	2.32E-04	26.731	IS
Total Lipids in HDL	rs4240624	8	A	G	0.909	0.092	0.006	1.10E-46	0.001	162.162	IS
Total Lipids in HDL	rs4759375	12	T	C	0.085	0.039	0.007	4.70E-08	2.31E-04	26.625	IS
Total Lipids in HDL	rs4846921	1	A	G	0.613	0.049	0.004	2.50E-40	0.001	133.245	IS
Total Lipids in HDL	rs4986970	16	T	A	0.034	-0.077	0.010	8.50E-15	3.89E-04	44.823	IS
Total Lipids in HDL	rs5167	19	G	T	0.352	0.050	0.004	2.20E-38	0.001	129.787	IS

Total Lipids in HDL	rs55779455	16	C	G	0.020	0.070	0.014	4.00E-08	1.92E-04	22.108	IS
Total Lipids in HDL	rs559355	11	T	A	0.158	-0.060	0.005	6.10E-32	0.001	109.749	IS
Total Lipids in HDL	rs59347135	8	G	C	0.046	-0.068	0.009	6.10E-14	3.99E-04	45.942	IS
Total Lipids in HDL	rs599839	1	A	G	0.772	-0.027	0.004	3.60E-10	2.56E-04	29.493	IS
Total Lipids in HDL	rs6018652	20	A	G	0.793	0.037	0.005	3.60E-17	4.56E-04	52.537	IS
Total Lipids in HDL	rs6073958	20	C	T	0.199	-0.059	0.005	1.10E-36	0.001	129.835	IS
Total Lipids in HDL	rs60847460	10	T	C	0.143	-0.038	0.005	1.50E-12	3.49E-04	40.119	IS
Total Lipids in HDL	rs61805076	1	C	T	0.334	-0.029	0.004	4.90E-14	3.73E-04	42.912	IS
Total Lipids in HDL	rs61941676	12	A	C	0.127	-0.060	0.006	1.20E-26	0.001	92.244	IS
Total Lipids in HDL	rs62101704	18	A	G	0.014	-0.089	0.016	7.00E-09	2.13E-04	24.571	IS
Total Lipids in HDL	rs625145	11	T	A	0.188	0.063	0.005	2.60E-43	0.001	140.528	IS
Total Lipids in HDL	rs638714	1	T	G	0.346	-0.038	0.004	4.00E-23	0.001	75.996	IS
Total Lipids in HDL	rs6509173	19	G	A	0.794	-0.030	0.005	2.00E-10	2.95E-04	33.918	IS
Total Lipids in HDL	rs6589565	11	G	A	0.932	0.051	0.007	4.20E-13	3.24E-04	37.257	IS
Total Lipids in HDL	rs676210	2	A	G	0.206	0.059	0.005	5.50E-38	0.001	132.025	IS
Total Lipids in HDL	rs686030	9	A	C	0.860	0.046	0.005	1.80E-18	0.001	58.361	IS
Total Lipids in HDL	rs7241918	18	T	G	0.823	0.090	0.005	6.10E-79	0.002	273.308	IS
Total Lipids in HDL	rs72836561	17	T	C	0.031	-0.135	0.011	5.50E-39	0.001	128.235	IS
Total Lipids in HDL	rs7308864	12	G	A	0.523	0.031	0.004	1.30E-17	4.82E-04	55.517	IS
Total Lipids in HDL	rs73632745	11	T	C	0.074	-0.045	0.007	1.10E-09	2.73E-04	31.369	IS
Total Lipids in HDL	rs737337	19	C	T	0.077	-0.068	0.007	5.60E-23	0.001	76.591	IS
Total Lipids in HDL	rs75714888	16	A	C	0.022	-0.071	0.013	3.70E-08	2.11E-04	24.320	IS
Total Lipids in HDL	rs75911530	16	A	G	0.033	-0.144	0.011	2.10E-42	0.001	152.389	IS
Total Lipids in HDL	rs77960347	18	G	A	0.013	0.303	0.016	4.00E-81	0.002	277.342	IS
Total Lipids in HDL	rs7810507	7	A	G	0.281	-0.025	0.004	5.00E-10	2.62E-04	30.125	IS
Total Lipids in HDL	rs7956099	12	C	T	0.475	-0.021	0.004	4.80E-09	2.28E-04	26.233	IS
Total Lipids in HDL	rs8058512	16	T	C	0.724	-0.025	0.004	1.20E-09	2.53E-04	29.136	IS
Total Lipids in HDL	rs838876	12	G	A	0.681	-0.041	0.004	3.50E-27	0.001	83.939	IS

Total Lipids in HDL	rs907866	2	A	G	0.443	-0.029	0.004	2.50E-15	4.28E-04	49.309	IS
Total Lipids in HDL	rs9491697	6	G	A	0.464	-0.024	0.004	1.60E-10	2.80E-04	32.196	IS
Total Lipids in HDL	rs967645	17	T	C	0.517	-0.026	0.004	1.70E-11	3.32E-04	38.203	IS
Total Lipids in HDL	rs9687833	5	A	G	0.207	-0.030	0.005	4.50E-11	2.87E-04	33.067	IS
Total Lipids in Medium HDL	rs1047891	2	A	C	0.315	-0.025	0.004	2.60E-09	2.67E-04	30.682	IS
Total Lipids in Medium HDL	rs1077835	15	G	A	0.220	0.102	0.005	9.71E-111	0.004	408.578	IS
Total Lipids in Medium HDL	rs11039238	11	C	T	0.322	-0.035	0.004	3.90E-18	0.001	63.213	IS
Total Lipids in Medium HDL	rs112495680	6	G	A	0.133	-0.034	0.006	5.20E-10	2.75E-04	31.660	IS
Total Lipids in Medium HDL	rs11601507	11	A	C	0.069	-0.039	0.007	3.60E-08	1.96E-04	22.591	IS
Total Lipids in Medium HDL	rs117687565	18	T	C	0.012	0.138	0.018	1.10E-13	4.54E-04	52.223	IS
Total Lipids in Medium HDL	rs11789603	9	T	C	0.109	0.050	0.006	1.40E-15	4.93E-04	56.712	IS
Total Lipids in Medium HDL	rs1260326	2	C	T	0.604	-0.046	0.004	3.30E-30	0.001	114.988	IS
Total Lipids in Medium HDL	rs12904367	15	A	G	0.141	0.031	0.006	4.80E-08	2.26E-04	26.047	IS
Total Lipids in Medium HDL	rs12976739	19	A	G	0.396	0.025	0.004	8.90E-11	2.89E-04	33.248	IS
Total Lipids in Medium HDL	rs13107325	4	T	C	0.074	-0.065	0.007	1.10E-19	0.001	67.044	IS
Total Lipids in Medium HDL	rs1321257	1	A	G	0.613	0.051	0.004	5.10E-39	0.001	139.755	IS
Total Lipids in Medium HDL	rs139915535	8	G	A	0.018	-0.123	0.014	9.50E-19	0.001	61.458	IS
Total Lipids in Medium HDL	rs15285	8	T	C	0.287	0.082	0.004	8.50E-85	0.003	315.371	IS
Total Lipids in Medium HDL	rs1800961	20	T	C	0.030	-0.113	0.011	2.10E-23	0.001	85.879	IS
Total Lipids in Medium HDL	rs193084249	1	G	A	0.023	-0.102	0.013	2.10E-15	4.76E-04	54.776	IS
Total Lipids in Medium HDL	rs2043085	15	C	T	0.613	-0.098	0.004	1.70E-139	0.005	524.066	IS
Total Lipids in Medium HDL	rs2072113	11	T	C	0.116	-0.045	0.006	7.60E-15	4.14E-04	47.704	IS
Total Lipids in Medium HDL	rs2176040	2	G	A	0.647	-0.025	0.004	1.30E-11	2.91E-04	33.508	IS
Total Lipids in Medium HDL	rs2236252	20	T	C	0.167	-0.032	0.005	6.50E-11	2.88E-04	33.145	IS
Total Lipids in Medium HDL	rs2298428	22	T	C	0.183	-0.042	0.005	4.10E-18	0.001	61.338	IS
Total Lipids in Medium HDL	rs235314	21	T	C	0.533	-0.021	0.004	4.50E-08	2.29E-04	26.343	IS
Total Lipids in Medium HDL	rs2569550	19	C	T	0.593	-0.027	0.004	5.40E-12	3.51E-04	40.427	IS
Total Lipids in Medium HDL	rs2642438	1	G	A	0.704	0.035	0.004	1.90E-17	0.001	58.159	IS

Total Lipids in Medium HDL	rs267738	1	G	T	0.220	0.039	0.005	5.50E-18	0.001	58.871	IS
Total Lipids in Medium HDL	rs2740488	9	C	A	0.265	-0.038	0.004	3.40E-19	0.001	64.149	IS
Total Lipids in Medium HDL	rs2792735	10	A	G	0.720	-0.039	0.004	1.20E-20	0.001	69.271	IS
Total Lipids in Medium HDL	rs28818616	3	C	T	0.350	-0.022	0.004	4.80E-08	2.13E-04	24.517	IS
Total Lipids in Medium HDL	rs2925979	16	C	T	0.699	0.028	0.004	2.90E-12	3.20E-04	36.878	IS
Total Lipids in Medium HDL	rs35135293	2	T	C	0.517	-0.028	0.004	9.30E-14	3.85E-04	44.296	IS
Total Lipids in Medium HDL	rs36057735	6	G	C	0.199	-0.036	0.005	1.90E-13	4.07E-04	46.888	IS
Total Lipids in Medium HDL	rs3764261	16	A	C	0.324	0.160	0.004	1.00E-200	0.011	1303.901	IS
Total Lipids in Medium HDL	rs3768321	1	T	G	0.197	-0.038	0.005	9.00E-16	4.50E-04	51.753	IS
Total Lipids in Medium HDL	rs3795269	1	A	C	0.439	0.022	0.004	7.00E-09	2.36E-04	27.201	IS
Total Lipids in Medium HDL	rs3859588	20	A	T	0.217	-0.026	0.005	4.50E-08	2.31E-04	26.641	IS
Total Lipids in Medium HDL	rs4239651	20	C	T	0.794	0.036	0.005	3.10E-15	4.27E-04	49.105	IS
Total Lipids in Medium HDL	rs4240624	8	A	G	0.909	0.101	0.007	7.30E-52	0.002	192.198	IS
Total Lipids in Medium HDL	rs4656292	1	G	A	0.620	0.024	0.004	7.40E-11	2.70E-04	31.052	IS
Total Lipids in Medium HDL	rs4969141	17	T	C	0.490	0.021	0.004	3.20E-08	2.14E-04	24.651	IS
Total Lipids in Medium HDL	rs4986970	16	T	A	0.034	-0.061	0.011	1.30E-09	2.46E-04	28.262	IS
Total Lipids in Medium HDL	rs559355	11	T	A	0.158	-0.069	0.005	1.10E-40	0.001	147.772	IS
Total Lipids in Medium HDL	rs583104	1	T	G	0.773	-0.051	0.005	1.90E-30	0.001	105.202	IS
Total Lipids in Medium HDL	rs59347135	8	G	C	0.046	-0.066	0.009	2.70E-12	3.78E-04	43.536	IS
Total Lipids in Medium HDL	rs60847460	10	T	C	0.143	-0.038	0.005	1.50E-11	3.48E-04	40.009	IS
Total Lipids in Medium HDL	rs61805076	1	C	T	0.334	-0.026	0.004	6.60E-11	3.02E-04	34.715	IS
Total Lipids in Medium HDL	rs61941676	12	A	C	0.127	-0.055	0.006	1.40E-20	0.001	76.764	IS
Total Lipids in Medium HDL	rs62101704	18	A	G	0.014	-0.089	0.017	4.50E-08	2.15E-04	24.757	IS
Total Lipids in Medium HDL	rs625145	11	T	A	0.188	0.071	0.005	3.40E-50	0.002	176.287	IS
Total Lipids in Medium HDL	rs638714	1	T	G	0.346	-0.051	0.004	6.30E-37	0.001	134.034	IS
Total Lipids in Medium HDL	rs6511720	19	T	G	0.120	0.049	0.006	7.70E-18	0.001	59.072	IS
Total Lipids in Medium HDL	rs6606717	12	C	A	0.523	0.031	0.004	4.30E-16	4.75E-04	54.733	IS
Total Lipids in Medium HDL	rs676210	2	A	G	0.206	0.061	0.005	2.10E-37	0.001	140.707	IS

Total Lipids in Medium HDL	rs686030	9	A	C	0.860	0.035	0.006	1.70E-10	2.89E-04	33.318	IS
Total Lipids in Medium HDL	rs72836561	17	T	C	0.031	-0.125	0.011	2.90E-31	0.001	110.479	IS
Total Lipids in Medium HDL	rs737338	19	T	C	0.035	-0.124	0.010	7.20E-34	0.001	119.386	IS
Total Lipids in Medium HDL	rs75911530	16	A	G	0.033	-0.135	0.011	3.30E-34	0.001	132.841	IS
Total Lipids in Medium HDL	rs77960347	18	G	A	0.013	0.327	0.017	4.10E-87	0.003	322.720	IS
Total Lipids in Medium HDL	rs7956099	12	C	T	0.475	-0.024	0.004	1.20E-10	2.96E-04	34.121	IS
Total Lipids in Medium HDL	rs838876	12	G	A	0.681	-0.037	0.004	6.00E-20	0.001	67.005	IS
Total Lipids in Medium HDL	rs9304381	18	T	C	0.818	0.097	0.005	1.40E-85	0.003	320.683	IS
Total Lipids in Medium HDL	rs9471972	6	A	G	0.536	0.027	0.004	1.20E-12	3.73E-04	42.886	IS
Total Lipids in Medium HDL	rs967645	17	T	C	0.517	-0.031	0.004	5.80E-15	4.85E-04	55.864	IS

Chr denotes chromosome; EAF, effective allele frequency; SE, standard error; $R^2 = 2 \times MAF \times (1-MAF) \times beta^2$; $F = R^2 \times (N-2)/(1-R^2)$.

Table S7. Causal associations between HDL subclasses and CAD and IS.

**Exposure**	**Outcome**	**Methods**	**NSNPs**	**OR (95%CI)**	***pval***
Total Concentration of Medium HDL Particles	CAD	IVW	74	0.84 (0.77, 0.93)	0.001
Total Concentration of Medium HDL Particles	CAD	MR Egger	74	0.89 (0.75, 1.05)	0.165
Total Concentration of Medium HDL Particles	CAD	Weighted median	74	0.88 (0.82, 0.93)	5.50E-05
Total Concentration of Medium HDL Particles	CAD	leave one out	74	0.84 (0.77, 0.93)	0.001
Total Concentration of Medium HDL Particles	CAD	Radial IVW	32	0.84 (0.80, 0.89)	3.99E-07
Total Lipids in HDL	CAD	IVW	85	0.87 (0.79, 0.96)	0.006
Total Lipids in HDL	CAD	MR Egger	85	0.95 (0.81, 1.13)	0.587
Total Lipids in HDL	CAD	Weighted median	85	0.90 (0.84, 0.96)	0.003
Total Lipids in HDL	CAD	leave one out	85	0.87 (0.79, 0.96)	0.006
Total Lipids in HDL	CAD	Radial IVW	34	0.86 (0.81, 0.90)	1.16E-06
Total Lipids in Medium HDL	CAD	IVW	67	0.83 (0.74, 0.92)	0.001
Total Lipids in Medium HDL	CAD	MR Egger	67	0.86 (0.71, 1.05)	0.135
Total Lipids in Medium HDL	CAD	Weighted median	67	0.88 (0.82, 0.95)	4.40E-04
Total Lipids in Medium HDL	CAD	leave one out	67	0.83 (0.74, 0.92)	0.001
Total Lipids in Medium HDL	CAD	Radial IVW	31	0.84 (0.79, 0.88)	2.53E-07
Total Cholesterol in Medium HDL	CAD	IVW	84	0.80 (0.73, 0.88)	8.65E-06
Total Cholesterol in Medium HDL	CAD	MR Egger	84	0.80 (0.68, 0.95)	0.012
Total Cholesterol in Medium HDL	CAD	Weighted median	84	0.84 (0.79, 0.88)	3.79E-10
Total Cholesterol in Medium HDL	CAD	leave one out	84	0.80 (0.73, 0.88)	8.65E-06
Total Cholesterol in Medium HDL	CAD	Radial IVW	36	0.82 (0.79, 0.86)	4.99E-10
Cholesteryl Esters in HDL	CAD	IVW	92	0.84 (0.77, 0.91)	1.49E-05
Cholesteryl Esters in HDL	CAD	MR Egger	92	0.86 (0.75, 0.99)	0.032
Cholesteryl Esters in HDL	CAD	Weighted median	92	0.85 (0.81, 0.89)	4.37E-11
Cholesteryl Esters in HDL	CAD	leave one out	92	0.84 (0.77, 0.91)	1.49E-05

Cholesteryl Esters in HDL	CAD	Radial IVW	45	0.84 (0.81, 0.88)	2.57E-10
Cholesteryl Esters in Medium HDL	CAD	IVW	84	0.76 (0.67, 0.86)	6.45E-06
Cholesteryl Esters in Medium HDL	CAD	MR Egger	84	0.72 (0.57, 0.91)	0.006
Cholesteryl Esters in Medium HDL	CAD	Weighted median	84	0.87 (0.79, 0.95)	0.002
Cholesteryl Esters in Medium HDL	CAD	leave one out	84	0.76 (0.67, 0.86)	6.45E-06
Cholesteryl Esters in Medium HDL	CAD	Radial IVW	39	0.75 (0.71, 0.81)	5.86E-10
Total Concentration of Medium HDL Particles	IS	IVW	69	0.93 (0.87, 0.98)	0.009
Total Concentration of Medium HDL Particles	IS	MR Egger	69	0.97 (0.87, 1.07)	0.522
Total Concentration of Medium HDL Particles	IS	Weighted median	69	0.95 (0.89, 1.02)	0.174
Total Concentration of Medium HDL Particles	IS	leave one out	69	0.93 (0.87, 0.98)	0.009
Total Concentration of Medium HDL Particles	IS	Radial IVW	60	0.94 (0.89, 0.98)	0.011
Total Lipids in HDL	IS	IVW	78	0.95 (0.89, 1.01)	0.097
Total Lipids in HDL	IS	MR Egger	78	1.03 (0.93, 1.14)	0.548
Total Lipids in HDL	IS	Weighted median	78	0.99 (0.93, 1.06)	0.828
Total Lipids in HDL	IS	leave one out	78	0.95 (0.89, 1.01)	0.097
Total Lipids in HDL	IS	Radial IVW	65	0.97 (0.94, 1.01)	0.164
Total Lipids in Medium HDL	IS	IVW	62	0.91 (0.86, 0.97)	0.005
Total Lipids in Medium HDL	IS	MR Egger	62	0.97 (0.87, 1.08)	0.576
Total Lipids in Medium HDL	IS	Weighted median	62	0.94 (0.88, 1.01)	0.107
Total Lipids in Medium HDL	IS	leave one out	62	0.91 (0.86, 0.97)	0.005
Total Lipids in Medium HDL	IS	Radial IVW	52	0.92 (0.88, 0.97)	0.004
Total Cholesterol in Medium HDL	IS	IVW	78	0.93 (0.88, 0.98)	0.010
Total Cholesterol in Medium HDL	IS	MR Egger	78	0.99 (0.90, 1.09)	0.860
Total Cholesterol in Medium HDL	IS	Weighted median	78	0.99 (0.93, 1.07)	0.908
Total Cholesterol in Medium HDL	IS	leave one out	78	0.93 (0.88, 0.98)	0.010
Total Cholesterol in Medium HDL	IS	Radial IVW	68	0.94 (0.89, 0.99)	0.035

Cholesteryl Esters in HDL	IS	IVW	81	0.97 (0.91, 1.03)	0.316
Cholesteryl Esters in HDL	IS	MR Egger	81	1.07 (0.97, 1.18)	0.154
Cholesteryl Esters in HDL	IS	Weighted median	81	1.00 (0.93, 1.08)	0.926
Cholesteryl Esters in HDL	IS	leave one out	81	0.97 (0.91, 1.03)	0.316
Cholesteryl Esters in HDL	IS	Radial IVW	70	0.99 (0.95, 1.04)	0.730
Cholesteryl Esters in Medium HDL	IS	IVW	78	0.90 (0.84, 0.96)	0.002
Cholesteryl Esters in Medium HDL	IS	MR Egger	78	0.93 (0.82, 1.06)	0.303
Cholesteryl Esters in Medium HDL	IS	Weighted median	78	0.90 (0.83, 0.98)	0.014
Cholesteryl Esters in Medium HDL	IS	leave one out	78	0.90 (0.84, 0.96)	0.002
Cholesteryl Esters in Medium HDL	IS	Radial IVW	66	0.89 (0.85, 0.93)	7.16E-06

IVW denotes inverse variance weighted method.

Table S8. Cochran Q and MR Egger pleiotropy tests of MR analysis of HDL subclasses with CAD and IS.

**Exposure**	**Outcome**	**IVW Cochran Q test**		**MR-Egger**		
		**Q-statistic**	**Q_p**	**intercept**	**SE**	***pval***
Total Concentration of Medium HDL Particles	CAD	956.269	5.19E-154	-0.003	0.005	0.490
Total Lipids in HDL	CAD	1352.677	6.45E-228	-0.006	0.005	0.171
Total Lipids in Medium HDL	CAD	1017.957	1.50E-170	-0.003	0.005	0.616
Total Cholesterol in Medium HDL	CAD	1146.401	1.23E-186	-1.67E-04	0.004	0.969
Cholesteryl Esters in HDL	CAD	988.267	1.17E-150	-0.002	0.004	0.567
Cholesteryl Esters in Medium HDL	CAD	1303.039	2.11E-218	0.003	0.005	0.591
Total Concentration of Medium HDL Particles	IS	113.173	4.83E-04	-0.003	0.003	0.294
Total Lipids in HDL	IS	165.034	2.30E-08	-0.006	0.003	0.053
Total Lipids in Medium HDL	IS	110.383	1.12E-04	-0.004	0.003	0.229
Total Cholesterol in Medium HDL	IS	127.831	2.45E-04	-0.004	0.003	0.108
Cholesteryl Esters in HDL	IS	161.884	1.71E-07	-0.007	0.003	0.011
Cholesteryl Esters in Medium HDL	IS	129.774	1.60E-04	-0.002	0.003	0.532

SE denotes standard error.

Table S9. Univariate and multivariate Causal associations of HDL subclasses with CAD and IS.

Exposure	Outcome	Models	Methods	NSNPs	OR (95%CI)	*pval*	*FDR*
Total Concentration of Medium HDL Particles	CAD	Model 1	IVW	74	0.84 (0.77, 0.93)	0.001	0.001
		Model 2	MV-IVW	60	0.87 (0.79, 0.95)	0.002	0.003
		Model 3	MV-IVW	36	0.90 (0.82, 0.99)	0.027	0.032
Total Lipids in HDL	CAD	Model 1	IVW	85	0.87 (0.79, 0.96)	0.006	0.006
		Model 2	MV-IVW	69	0.87 (0.78, 0.96)	0.007	0.007
		Model 3	MV-IVW	39	0.89 (0.82, 0.98)	0.015	0.023
Total Lipids in Medium HDL	CAD	Model 1	IVW	67	0.83 (0.74, 0.92)	0.001	0.001
		Model 2	MV-IVW	54	0.85 (0.77, 0.95)	0.003	0.003
		Model 3	MV-IVW	33	0.90 (0.82, 1.00)	0.055	0.055
Total Cholesterol in Medium HDL	CAD	Model 1	IVW	84	0.80 (0.73, 0.88)	8.65E-06	2.60E-05
		Model 2	MV-IVW	71	0.80 (0.72, 0.88)	1.49E-05	2.99E-05
		Model 3	MV-IVW	38	0.83 (0.75, 0.92)	2.34E-04	4.68E-04
Cholesteryl Esters in HDL	CAD	Model 1	IVW	92	0.84 (0.77, 0.91)	1.49E-05	2.98E-05
		Model 2	MV-IVW	74	0.82 (0.75, 0.89)	8.38E-06	2.51E-05
		Model 3	MV-IVW	44	0.84 (0.77, 0.92)	2.32E-04	4.68E-04
Cholesteryl Esters in Medium HDL	CAD	Model 1	IVW	84	0.76 (0.67, 0.86)	6.45E-06	2.60E-05
		Model 2	MV-IVW	71	0.77 (0.69, 0.86)	1.34E-06	8.05E-06
		Model 3	MV-IVW	39	0.82 (0.74, 0.90)	6.21E-05	3.73E-04
Total Concentration of Medium HDL Particles	IS	Model 1	IVW	69	0.93 (0.87, 0.98)	0.009	0.015
		Model 2	MV-IVW	59	0.95 (0.89, 1.01)	0.083	0.099
		Model 3	MV-IVW	35	0.94 (0.88, 1.00)	0.061	0.073
Total Lipids in HDL	IS	Model 1	IVW	78	0.95 (0.89, 1.01)	0.097	0.116
		Model 2	MV-IVW	66	0.96 (0.90, 1.02)	0.202	0.202
		Model 3	MV-IVW	36	0.93 (0.88, 0.99)	0.031	0.062

		Model 1	IVW	62	0.91 (0.86, 0.97)	0.005	0.015
Total Lipids in Medium HDL	IS	Model 2	MV-IVW	53	0.93 (0.88, 1.00)	0.037	0.055
		Model 3	MV-IVW	33	0.94 (0.87, 1.00)	0.049	0.073
		Model 1	IVW	78	0.93 (0.88, 0.98)	0.01	0.015
Total Cholesterol in Medium HDL	IS	Model 2	MV-IVW	68	0.91 (0.86, 0.97)	0.002	0.007
		Model 3	MV-IVW	35	0.92 (0.86, 0.99)	0.019	0.062
		Model 1	IVW	81	0.97 (0.91, 1.03)	0.316	0.316
Cholesteryl Esters in HDL	IS	Model 2	MV-IVW	70	0.93 (0.88, 0.99)	0.025	0.05
		Model 3	MV-IVW	41	0.94 (0.89, 1.01)	0.075	0.075
		Model 1	IVW	78	0.90 (0.84, 0.96)	0.002	0.012
Cholesteryl Esters in Medium HDL	IS	Model 2	MV-IVW	68	0.91 (0.86, 0.97)	0.002	0.007
		Model 3	MV-IVW	36	0.92 (0.86, 0.99)	0.023	0.062

Model 1 was the unadjusted model, Model 2 was adjusted for smoking and alcohol drinking, and Model 3 was adjusted for LDL-c. IVW denotes inverse variance weighted method; *FDR* < 0.05 was considered significant.

Table S10. Colocalization of HDL subclasses with CAD and IS.

Exposure	Outcome	SNP	Chr	BP	NSNPs	PP.H3	PP.H4
Cholesteryl esters in medium HDL	CAD	rs3795269	1	2338126	4294	0.9635	0.0005
Cholesteryl esters in medium HDL	CAD	rs114165349	1	27021913	2696	0.0137	0.9859
Cholesteryl esters in medium HDL	CAD	rs3768321	1	40035928	3495	0.1716	0.0415
Cholesteryl esters in medium HDL	CAD	rs11591147	1	55505647	4640	7.20E-08	0.9999
Cholesteryl esters in medium HDL	CAD	rs7534572	1	62999675	3655	0.2324	0.0027
Cholesteryl esters in medium HDL	CAD	rs583104	1	109821307	3505	0.0204	0.9796
Cholesteryl esters in medium HDL	CAD	rs267738	1	150940625	2741	1.0000	2.44E-10
Cholesteryl esters in medium HDL	CAD	rs4656292	1	161194641	4178	0.1294	0.0029
Cholesteryl esters in medium HDL	CAD	rs61805076	1	182154990	3821	0.0621	0.0294
Cholesteryl esters in medium HDL	CAD	rs2642438	1	220970028	3676	0.0828	0.0192
Cholesteryl esters in medium HDL	CAD	rs4846921	1	230304352	4012	0.0816	0.9130
Cholesteryl esters in medium HDL	CAD	rs35135293	2	20363666	4173	0.9991	5.98E-06
Cholesteryl esters in medium HDL	CAD	rs676210	2	21231524	3659	1.0000	2.96E-15
Cholesteryl esters in medium HDL	CAD	rs1260326	2	27730940	2458	0.0796	0.2225
Cholesteryl esters in medium HDL	CAD	rs10184004	2	165508389	3149	0.9553	0.0404
Cholesteryl esters in medium HDL	CAD	rs1047891	2	211540507	3857	0.4301	0.3549
Cholesteryl esters in medium HDL	CAD	rs2943650	2	227105921	3444	0.0582	0.9418
Cholesteryl esters in medium HDL	CAD	rs28818616	3	15592561	3958	0.1425	0.0025
Cholesteryl esters in medium HDL	CAD	rs9647335	3	135880410	2452	0.0256	0.9744
Cholesteryl esters in medium HDL	CAD	rs13107325	4	103188709	3697	0.0776	0.0064
Cholesteryl esters in medium HDL	CAD	rs9687846	5	55861894	4399	0.0129	0.9871
Cholesteryl esters in medium HDL	CAD	rs36057735	6	31319923	14424	0.9999	0.0001
Cholesteryl esters in medium HDL	CAD	rs28746853	6	32634646	24188	0.9523	0.0080
Cholesteryl esters in medium HDL	CAD	rs2395943	6	42940673	3062	0.9981	0.0003

Cholesteryl esters in medium HDL	CAD	rs1358980	6	43764551	3490	0.0262	0.9738
Cholesteryl esters in medium HDL	CAD	rs9491697	6	127456122	3072	0.9956	0.0012
Cholesteryl esters in medium HDL	CAD	rs11751347	6	161092438	4350	1.0000	7.62E-07
Cholesteryl esters in medium HDL	CAD	rs6967917	7	17984297	4335	0.0845	0.0029
Cholesteryl esters in medium HDL	CAD	rs12533197	7	36192606	3718	0.1171	0.0589
Cholesteryl esters in medium HDL	CAD	rs7810507	7	130437476	3190	0.1688	0.2734
Cholesteryl esters in medium HDL	CAD	rs4240624	8	9184231	6007	0.1060	0.0067
Cholesteryl esters in medium HDL	CAD	rs59347135	8	19750044	5176	0.0579	0.9421
Cholesteryl esters in medium HDL	CAD	rs139915535	8	19766233	5202	0.0579	0.9421
Cholesteryl esters in medium HDL	CAD	rs15285	8	19824667	5272	0.0579	0.9421
Cholesteryl esters in medium HDL	CAD	rs2245221	8	116624879	2846	0.0829	0.0046
Cholesteryl esters in medium HDL	CAD	rs7388248	8	144305353	4730	0.1504	0.0038
Cholesteryl esters in medium HDL	CAD	rs686030	9	15304782	4634	0.1036	0.0043
Cholesteryl esters in medium HDL	CAD	rs11789603	9	107647019	4777	0.9665	0.0199
Cholesteryl esters in medium HDL	CAD	rs2740488	9	107661742	4768	0.9665	0.0199
Cholesteryl esters in medium HDL	CAD	rs2792735	10	113921825	3443	0.0681	0.0042
Cholesteryl esters in medium HDL	CAD	rs60847460	10	113983758	3391	0.0673	0.0042
Cholesteryl esters in medium HDL	CAD	rs35184771	11	47475189	2832	0.1227	0.0099
Cholesteryl esters in medium HDL	CAD	rs174578	11	61605499	3497	0.1733	0.0136
Cholesteryl esters in medium HDL	CAD	rs559355	11	75451281	3040	1.0000	5.83E-11
Cholesteryl esters in medium HDL	CAD	rs6589565	11	116640237	4703	0.3264	0.6736
Cholesteryl esters in medium HDL	CAD	rs78296522	11	116648776	4694	0.3264	0.6736
Cholesteryl esters in medium HDL	CAD	rs59097294	11	116964437	4529	0.3264	0.6736
Cholesteryl esters in medium HDL	CAD	rs141368429	11	117221862	4250	0.9619	0.0005
Cholesteryl esters in medium HDL	CAD	rs7134375	12	20473758	4771	0.9766	0.0002
Cholesteryl esters in medium HDL	CAD	rs2229357	12	57843711	2559	0.0260	0.9729

Cholesteryl esters in medium HDL	CAD	rs7956099	12	103556109	3820	0.0974	0.0417
Cholesteryl esters in medium HDL	CAD	rs6606717	12	109873227	3637	0.1830	0.0021
Cholesteryl esters in medium HDL	CAD	rs7959043	12	124336478	4019	0.0290	0.9709
Cholesteryl esters in medium HDL	CAD	rs11057692	12	125071027	4615	1.0000	1.34E-14
Cholesteryl esters in medium HDL	CAD	rs838876	12	125259888	4539	1.0000	1.34E-14
Cholesteryl esters in medium HDL	CAD	rs61941676	12	125324798	4552	1.0000	1.34E-14
Cholesteryl esters in medium HDL	CAD	rs2494748	14	105258892	4352	0.1445	0.1099
Cholesteryl esters in medium HDL	CAD	rs1601935	15	58671765	4149	0.9990	0.0000
Cholesteryl esters in medium HDL	CAD	rs1077835	15	58723426	4031	0.9990	0.0000
Cholesteryl esters in medium HDL	CAD	rs904770	16	56702000	4213	0.0322	0.9678
Cholesteryl esters in medium HDL	CAD	rs75911530	16	57049137	4392	0.0322	0.9678
Cholesteryl esters in medium HDL	CAD	rs8058512	16	57151796	4348	0.0322	0.9678
Cholesteryl esters in medium HDL	CAD	rs4330777	16	57235644	4303	0.0322	0.9678
Cholesteryl esters in medium HDL	CAD	rs4986970	16	67976320	2691	0.0871	0.0081
Cholesteryl esters in medium HDL	CAD	rs2925979	16	81534790	6494	0.9321	0.0663
Cholesteryl esters in medium HDL	CAD	rs967645	17	26713970	2005	0.4443	0.0200
Cholesteryl esters in medium HDL	CAD	rs72836561	17	41926126	2991	0.1652	0.8202
Cholesteryl esters in medium HDL	CAD	rs112001035	17	66823805	3369	0.9452	0.0539
Cholesteryl esters in medium HDL	CAD	rs4969141	17	76391653	4529	0.9130	0.0002
Cholesteryl esters in medium HDL	CAD	rs77960347	18	47109955	4319	0.9999	0.0000
Cholesteryl esters in medium HDL	CAD	rs62101705	18	47135834	4352	0.9999	0.0000
Cholesteryl esters in medium HDL	CAD	rs117687565	18	47147524	4387	0.9999	0.0000
Cholesteryl esters in medium HDL	CAD	rs9304381	18	47158234	4404	0.9999	0.0000
Cholesteryl esters in medium HDL	CAD	rs116843064	19	8429323	4173	2.33E-15	0.9999
Cholesteryl esters in medium HDL	CAD	rs2228671	19	11210912	3617	1.0000	0.0000
Cholesteryl esters in medium HDL	CAD	rs12611067	19	11230402	3606	1.0000	0.0000

Cholesteryl esters in medium HDL	CAD	rs737337	19	11347493	3663	1.0000	0.0000
Cholesteryl esters in medium HDL	CAD	rs429358	19	45411941	4259	0.3737	0.6263
Cholesteryl esters in medium HDL	CAD	rs144311893	19	45423944	4210	0.3737	0.6263
Cholesteryl esters in medium HDL	CAD	rs1761457	19	54814524	5485	0.1232	0.1002
Cholesteryl esters in medium HDL	CAD	rs2236252	20	17597531	4626	0.0159	0.9832
Cholesteryl esters in medium HDL	CAD	rs1800961	20	43042364	3585	0.2117	0.0076
Cholesteryl esters in medium HDL	CAD	rs4239651	20	46340596	3764	0.0899	0.0035
Cholesteryl esters in medium HDL	CAD	rs2298428	22	21982892	2427	0.0485	0.0086
Total Cholesterol in medium HDL	CAD	rs3795269	1	2338126	4294	0.9466	0.0007
Total Cholesterol in medium HDL	CAD	rs114165349	1	27021913	2696	0.0137	0.9860
Total Cholesterol in medium HDL	CAD	rs3768321	1	40035928	3495	0.1718	0.0404
Total Cholesterol in medium HDL	CAD	rs638714	1	62906489	3755	0.1268	0.0030
Total Cholesterol in medium HDL	CAD	rs583104	1	109821307	3505	0.0211	0.9789
Total Cholesterol in medium HDL	CAD	rs267738	1	150940625	2741	1.0000	2.60E-10
Total Cholesterol in medium HDL	CAD	rs4656292	1	161194641	4178	0.1143	0.0038
Total Cholesterol in medium HDL	CAD	rs61805076	1	182154990	3821	0.0621	0.0295
Total Cholesterol in medium HDL	CAD	rs2642438	1	220970028	3676	0.0828	0.0192
Total Cholesterol in medium HDL	CAD	rs4846921	1	230304352	4012	0.0819	0.9126
Total Cholesterol in medium HDL	CAD	rs35135293	2	20363666	4173	0.9999	8.64E-07
Total Cholesterol in medium HDL	CAD	rs676210	2	21231524	3659	1.0000	2.96E-15
Total Cholesterol in medium HDL	CAD	rs1260326	2	27730940	2458	0.0784	0.2379
Total Cholesterol in medium HDL	CAD	rs10184004	2	165508389	3149	0.9543	0.0415
Total Cholesterol in medium HDL	CAD	rs1047891	2	211540507	3857	0.4620	0.3070
Total Cholesterol in medium HDL	CAD	rs2943650	2	227105921	3444	0.0581	0.9419
Total Cholesterol in medium HDL	CAD	rs28818616	3	15592561	3958	0.1422	0.0026
Total Cholesterol in medium HDL	CAD	rs9647335	3	135880410	2452	0.0312	0.9688

Total Cholesterol in medium HDL	CAD	rs13107325	4	103188709	3697	0.0776	0.0064
Total Cholesterol in medium HDL	CAD	rs9687846	5	55861894	4399	0.0123	0.9877
Total Cholesterol in medium HDL	CAD	rs36057735	6	31319923	14424	0.9999	4.66E-05
Total Cholesterol in medium HDL	CAD	rs28746853	6	32634646	24188	0.9522	0.0084
Total Cholesterol in medium HDL	CAD	rs2395943	6	42940673	3062	0.9981	0.0003
Total Cholesterol in medium HDL	CAD	rs1358980	6	43764551	3490	0.0269	0.9731
Total Cholesterol in medium HDL	CAD	rs9491697	6	127456122	3072	0.9956	0.0013
Total Cholesterol in medium HDL	CAD	rs11751347	6	161092438	4350	1.0000	1.09E-06
Total Cholesterol in medium HDL	CAD	rs6967917	7	17984297	4335	0.0844	0.0029
Total Cholesterol in medium HDL	CAD	rs12533197	7	36192606	3718	0.1178	0.0528
Total Cholesterol in medium HDL	CAD	rs7810507	7	130437476	3190	0.1692	0.2715
Total Cholesterol in medium HDL	CAD	rs4240624	8	9184231	6007	0.1060	0.0067
Total Cholesterol in medium HDL	CAD	rs59347135	8	19750044	5176	0.0585	0.9415
Total Cholesterol in medium HDL	CAD	rs139915535	8	19766233	5202	0.0585	0.9415
Total Cholesterol in medium HDL	CAD	rs15285	8	19824667	5272	0.0585	0.9415
Total Cholesterol in medium HDL	CAD	rs2245221	8	116624879	2846	0.0829	0.0046
Total Cholesterol in medium HDL	CAD	rs7388248	8	144305353	4730	0.1507	0.0036
Total Cholesterol in medium HDL	CAD	rs686030	9	15304782	4634	0.1036	0.0042
Total Cholesterol in medium HDL	CAD	rs11789603	9	107647019	4777	0.9680	0.0183
Total Cholesterol in medium HDL	CAD	rs2740488	9	107661742	4768	0.9680	0.0183
Total Cholesterol in medium HDL	CAD	rs2792735	10	113921825	3443	0.0681	0.0042
Total Cholesterol in medium HDL	CAD	rs60847460	10	113983758	3391	0.0673	0.0042
Total Cholesterol in medium HDL	CAD	rs35184771	11	47475189	2832	0.1226	0.0103
Total Cholesterol in medium HDL	CAD	rs174578	11	61605499	3497	0.1731	0.0146
Total Cholesterol in medium HDL	CAD	rs559355	11	75451281	3040	1.0000	5.82E-11
Total Cholesterol in medium HDL	CAD	rs6589565	11	116640237	4703	1.0000	1.59E-07

Total Cholesterol in medium HDL	CAD	rs78296522	11	116648776	4694	1.0000	1.59E-07
Total Cholesterol in medium HDL	CAD	rs59097294	11	116964437	4529	1.0000	1.59E-07
Total Cholesterol in medium HDL	CAD	rs141368429	11	117221862	4250	0.9619	0.0005
Total Cholesterol in medium HDL	CAD	rs7134375	12	20473758	4771	0.9663	0.0003
Total Cholesterol in medium HDL	CAD	rs2229357	12	57843711	2559	0.0261	0.9727
Total Cholesterol in medium HDL	CAD	rs7956099	12	103556109	3820	0.0991	0.0424
Total Cholesterol in medium HDL	CAD	rs6606717	12	109873227	3637	0.1830	0.0021
Total Cholesterol in medium HDL	CAD	rs1054852	12	124496316	4104	0.0290	0.9710
Total Cholesterol in medium HDL	CAD	rs11057692	12	125071027	4615	1.0000	3.46E-15
Total Cholesterol in medium HDL	CAD	rs921919	12	125265201	4557	1.0000	3.46E-15
Total Cholesterol in medium HDL	CAD	rs7136506	12	125326153	4550	1.0000	3.46E-15
Total Cholesterol in medium HDL	CAD	rs2494748	14	105258892	4352	0.1433	0.1087
Total Cholesterol in medium HDL	CAD	rs10162642	15	58577163	4218	0.9990	0.0000
Total Cholesterol in medium HDL	CAD	rs1601935	15	58671765	4149	0.9990	0.0000
Total Cholesterol in medium HDL	CAD	rs1077835	15	58723426	4031	0.9990	0.0000
Total Cholesterol in medium HDL	CAD	rs904770	16	56702000	4213	0.0324	0.9676
Total Cholesterol in medium HDL	CAD	rs3764261	16	56993324	4328	0.0324	0.9676
Total Cholesterol in medium HDL	CAD	rs75911530	16	57049137	4392	0.0324	0.9676
Total Cholesterol in medium HDL	CAD	rs8058512	16	57151796	4348	0.0324	0.9676
Total Cholesterol in medium HDL	CAD	rs4330777	16	57235644	4303	0.0324	0.9676
Total Cholesterol in medium HDL	CAD	rs4986970	16	67976320	2691	0.0871	0.0083
Total Cholesterol in medium HDL	CAD	rs2925979	16	81534790	6494	0.9321	0.0662
Total Cholesterol in medium HDL	CAD	rs967645	17	26713970	2005	0.4442	0.0201
Total Cholesterol in medium HDL	CAD	rs72836561	17	41926126	2991	0.1652	0.8202
Total Cholesterol in medium HDL	CAD	rs112001035	17	66823805	3369	0.9450	0.0540
Total Cholesterol in medium HDL	CAD	rs4969141	17	76391653	4529	0.9129	0.0002

Total Cholesterol in medium HDL	CAD	rs77960347	18	47109955	4319	0.9999	0.0000
Total Cholesterol in medium HDL	CAD	rs12608026	18	47120499	4333	0.9999	0.0000
Total Cholesterol in medium HDL	CAD	rs62101705	18	47135834	4352	0.9999	0.0000
Total Cholesterol in medium HDL	CAD	rs7241918	18	47160953	4408	0.9999	0.0000
Total Cholesterol in medium HDL	CAD	rs116843064	19	8429323	4173	2.33E-15	0.9999
Total Cholesterol in medium HDL	CAD	rs2978615	19	11243260	3629	1.0000	9.64E-09
Total Cholesterol in medium HDL	CAD	rs737337	19	11347493	3663	1.0000	9.64E-09
Total Cholesterol in medium HDL	CAD	rs429358	19	45411941	4259	0.9995	0.0005
Total Cholesterol in medium HDL	CAD	rs144311893	19	45423944	4210	0.9995	0.0005
Total Cholesterol in medium HDL	CAD	rs1761457	19	54814524	5485	0.1221	0.1078
Total Cholesterol in medium HDL	CAD	rs2236252	20	17597531	4626	0.0165	0.9829
Total Cholesterol in medium HDL	CAD	rs1800961	20	43042364	3585	0.2117	0.0076
Total Cholesterol in medium HDL	CAD	rs4239651	20	46340596	3764	0.0899	0.0035
Total Cholesterol in medium HDL	CAD	rs2298428	22	21982892	2427	0.0485	0.0086
Total lipids in medium HDL	CAD	rs3795269	1	2338126	4294	0.9842	0.0001
Total lipids in medium HDL	CAD	rs193084249	1	26987646	2707	0.0181	0.9814
Total lipids in medium HDL	CAD	rs3768321	1	40035928	3495	0.1723	0.0375
Total lipids in medium HDL	CAD	rs638714	1	62906489	3755	0.1268	0.0030
Total lipids in medium HDL	CAD	rs583104	1	109821307	3505	0.0183	0.9817
Total lipids in medium HDL	CAD	rs267738	1	150940625	2741	1.0000	2.63E-10
Total lipids in medium HDL	CAD	rs4656292	1	161194641	4178	0.1347	0.0026
Total lipids in medium HDL	CAD	rs61805076	1	182154990	3821	0.0621	0.0292
Total lipids in medium HDL	CAD	rs2642438	1	220970028	3676	0.0828	0.0194
Total lipids in medium HDL	CAD	rs1321257	1	230305312	4009	0.0835	0.9109
Total lipids in medium HDL	CAD	rs35135293	2	20363666	4173	1.0000	2.84E-10
Total lipids in medium HDL	CAD	rs676210	2	21231524	3659	1.0000	2.93E-15

Total lipids in medium HDL	CAD	rs1260326	2	27730940	2458	0.0730	0.2896
Total lipids in medium HDL	CAD	rs1047891	2	211540507	3857	0.6142	0.0785
Total lipids in medium HDL	CAD	rs2176040	2	227092802	3440	0.0584	0.9416
Total lipids in medium HDL	CAD	rs28818616	3	15592561	3958	0.1383	0.0027
Total lipids in medium HDL	CAD	rs13107325	4	103188709	3697	0.0776	0.0064
Total lipids in medium HDL	CAD	rs36057735	6	31319923	14424	0.9999	0.0000
Total lipids in medium HDL	CAD	rs112495680	6	34223098	5397	0.9453	0.0545
Total lipids in medium HDL	CAD	rs9471972	6	42915021	3083	0.9982	0.0003
Total lipids in medium HDL	CAD	rs4240624	8	9184231	6007	0.1060	0.0067
Total lipids in medium HDL	CAD	rs59347135	8	19750044	5176	0.0643	0.9357
Total lipids in medium HDL	CAD	rs139915535	8	19766233	5202	0.0643	0.9357
Total lipids in medium HDL	CAD	rs15285	8	19824667	5272	0.0643	0.9357
Total lipids in medium HDL	CAD	rs7388248	8	144305353	4730	0.1510	0.0033
Total lipids in medium HDL	CAD	rs686030	9	15304782	4634	0.1036	0.0039
Total lipids in medium HDL	CAD	rs11789603	9	107647019	4777	0.9590	0.0275
Total lipids in medium HDL	CAD	rs2740488	9	107661742	4768	0.9590	0.0275
Total lipids in medium HDL	CAD	rs140168704	10	5262778	6120	0.0900	0.0048
Total lipids in medium HDL	CAD	rs2792735	10	113921825	3443	0.0681	0.0040
Total lipids in medium HDL	CAD	rs60847460	10	113983758	3391	0.0673	0.0040
Total lipids in medium HDL	CAD	rs11601507	11	5701074	6640	0.0000	0.9997
Total lipids in medium HDL	CAD	rs11039238	11	47460901	2803	0.1119	0.0119
Total lipids in medium HDL	CAD	rs2072113	11	61604967	3491	0.1748	0.0048
Total lipids in medium HDL	CAD	rs559355	11	75451281	3040	1.0000	5.84E-11
Total lipids in medium HDL	CAD	rs625145	11	116727936	4472	1.0000	1.69E-12
Total lipids in medium HDL	CAD	rs7956099	12	103556109	3820	0.1009	0.0413
Total lipids in medium HDL	CAD	rs6606717	12	109873227	3637	0.1830	0.0022

Total lipids in medium HDL	CAD	rs838876	12	125259888	4539	1.0000	1.33E-15
Total lipids in medium HDL	CAD	rs61941676	12	125324798	4552	1.0000	1.33E-15
Total lipids in medium HDL	CAD	rs12904367	15	58547878	4240	0.9990	0.0001
Total lipids in medium HDL	CAD	rs2043085	15	58680954	4130	0.9990	0.0001
Total lipids in medium HDL	CAD	rs1077835	15	58723426	4031	0.9990	0.0001
Total lipids in medium HDL	CAD	rs3764261	16	56993324	4328	0.0335	0.9665
Total lipids in medium HDL	CAD	rs75911530	16	57049137	4392	0.0335	0.9665
Total lipids in medium HDL	CAD	rs4986970	16	67976320	2691	0.0866	0.0077
Total lipids in medium HDL	CAD	rs2925979	16	81534790	6494	0.9336	0.0647
Total lipids in medium HDL	CAD	rs967645	17	26713970	2005	0.4441	0.0203
Total lipids in medium HDL	CAD	rs72836561	17	41926126	2991	0.1655	0.8198
Total lipids in medium HDL	CAD	rs112001035	17	66823805	3369	0.9420	0.0563
Total lipids in medium HDL	CAD	rs4969141	17	76391653	4529	0.8885	0.0003
Total lipids in medium HDL	CAD	rs77960347	18	47109955	4319	0.9992	0.0006
Total lipids in medium HDL	CAD	rs62101704	18	47134250	4347	0.9992	0.0006
Total lipids in medium HDL	CAD	rs117687565	18	47147524	4387	0.9992	0.0006
Total lipids in medium HDL	CAD	rs9304381	18	47158234	4404	0.9992	0.0006
Total lipids in medium HDL	CAD	rs12976739	19	8461663	4163	2.50E-15	0.9999
Total lipids in medium HDL	CAD	rs6511720	19	11202306	3610	1.0000	2.69E-14
Total lipids in medium HDL	CAD	rs2569550	19	11228745	3610	1.0000	2.69E-14
Total lipids in medium HDL	CAD	rs737338	19	11347657	3664	1.0000	2.69E-14
Total lipids in medium HDL	CAD	rs117310449	19	45393516	4378	0.0092	0.9908
Total lipids in medium HDL	CAD	rs1065853	19	45413233	4259	0.0092	0.9908
Total lipids in medium HDL	CAD	rs2236252	20	17597531	4626	0.0195	0.9803
Total lipids in medium HDL	CAD	rs1800961	20	43042364	3585	0.2117	0.0076
Total lipids in medium HDL	CAD	rs4239651	20	46340596	3764	0.0899	0.0035

Total lipids in medium HDL	CAD	rs3859588	20	46476143	4069	0.0946	0.0035
Total lipids in medium HDL	CAD	rs235314	21	46271452	4989	0.2100	0.0405
Total lipids in medium HDL	CAD	rs2298428	22	21982892	2427	0.0485	0.0088
Cholesteryl esters in medium HDL	IS	rs3795269	1	2338126	2542	0.0014	0.0019
Cholesteryl esters in medium HDL	IS	rs114165349	1	27021913	1855	0.0040	0.0205
Cholesteryl esters in medium HDL	IS	rs3768321	1	40035928	2359	0.0008	0.0011
Cholesteryl esters in medium HDL	IS	rs11591147	1	55505647	3443	0.0041	0.0118
Cholesteryl esters in medium HDL	IS	rs7534572	1	62999675	2716	0.0031	0.9795
Cholesteryl esters in medium HDL	IS	rs583104	1	109821307	2497	0.0144	0.5488
Cholesteryl esters in medium HDL	IS	rs267738	1	150940625	1600	0.0004	0.0007
Cholesteryl esters in medium HDL	IS	rs4656292	1	161194641	2676	0.0020	0.0091
Cholesteryl esters in medium HDL	IS	rs61805076	1	182154990	2737	0.0033	0.0081
Cholesteryl esters in medium HDL	IS	rs2642438	1	220970028	2498	0.0012	0.0014
Cholesteryl esters in medium HDL	IS	rs4846921	1	230304352	2910	0.0654	0.0055
Cholesteryl esters in medium HDL	IS	rs35135293	2	20363666	3179	0.0036	0.0207
Cholesteryl esters in medium HDL	IS	rs676210	2	21231524	2641	0.0019	0.0043
Cholesteryl esters in medium HDL	IS	rs1260326	2	27730940	1239	0.0005	0.0061
Cholesteryl esters in medium HDL	IS	rs10184004	2	165508389	2194	0.0017	0.0056
Cholesteryl esters in medium HDL	IS	rs1047891	2	211540507	2655	0.0031	0.0104
Cholesteryl esters in medium HDL	IS	rs2943650	2	227105921	2407	0.0022	0.0096
Cholesteryl esters in medium HDL	IS	rs28818616	3	15592561	2420	0.0011	0.0018
Cholesteryl esters in medium HDL	IS	rs9647335	3	135880410	1335	0.0005	0.0008
Cholesteryl esters in medium HDL	IS	rs13107325	4	103188709	2380	0.0009	0.0034
Cholesteryl esters in medium HDL	IS	rs9687846	5	55861894	3385	0.0036	0.0196
Cholesteryl esters in medium HDL	IS	rs36057735	6	31319923	9410	0.0366	0.5252
Cholesteryl esters in medium HDL	IS	rs28746853	6	32634646	12931	0.0317	0.9320

Cholesteryl esters in medium HDL	IS	rs2395943	6	42940673	2055	0.9957	0.0003
Cholesteryl esters in medium HDL	IS	rs1358980	6	43764551	2496	0.0094	0.9905
Cholesteryl esters in medium HDL	IS	rs9491697	6	127456122	2152	0.0219	0.1242
Cholesteryl esters in medium HDL	IS	rs11751347	6	161092438	3025	0.0041	0.2138
Cholesteryl esters in medium HDL	IS	rs6967917	7	17984297	3151	0.0041	0.0106
Cholesteryl esters in medium HDL	IS	rs12533197	7	36192606	2684	0.0016	0.0034
Cholesteryl esters in medium HDL	IS	rs7810507	7	130437476	2213	0.0033	0.0244
Cholesteryl esters in medium HDL	IS	rs4240624	8	9184231	4272	0.0042	0.1517
Cholesteryl esters in medium HDL	IS	rs59347135	8	19750044	3831	0.0037	0.0090
Cholesteryl esters in medium HDL	IS	rs139915535	8	19766233	3845	0.0037	0.0090
Cholesteryl esters in medium HDL	IS	rs15285	8	19824667	1450	0.0003	0.0002
Cholesteryl esters in medium HDL	IS	rs2245221	8	116624879	1657	0.0004	0.0006
Cholesteryl esters in medium HDL	IS	rs686030	9	15304782	3249	0.0515	0.0109
Cholesteryl esters in medium HDL	IS	rs11789603	9	107647019	3591	0.0049	0.2402
Cholesteryl esters in medium HDL	IS	rs2740488	9	107661742	3590	0.0821	0.0042
Cholesteryl esters in medium HDL	IS	rs2792735	10	113921825	2238	0.0013	0.0023
Cholesteryl esters in medium HDL	IS	rs60847460	10	113983758	2226	0.0013	0.0024
Cholesteryl esters in medium HDL	IS	rs35184771	11	47475189	1532	0.0102	0.1373
Cholesteryl esters in medium HDL	IS	rs174578	11	61605499	2270	0.0040	0.1318
Cholesteryl esters in medium HDL	IS	rs559355	11	75451281	2034	0.0094	0.0444
Cholesteryl esters in medium HDL	IS	rs6589565	11	116640237	3529	0.0056	0.0175
Cholesteryl esters in medium HDL	IS	rs78296522	11	116648776	3525	0.0056	0.0175
Cholesteryl esters in medium HDL	IS	rs59097294	11	116964437	3454	0.0051	0.0174
Cholesteryl esters in medium HDL	IS	rs141368429	11	117221862	3203	0.0047	0.0095
Cholesteryl esters in medium HDL	IS	rs7134375	12	20473758	3554	0.0015	0.9976
Cholesteryl esters in medium HDL	IS	rs2229357	12	57843711	1489	0.0034	0.0634

Cholesteryl esters in medium HDL	IS	rs7956099	12	103556109	2662	0.0056	0.0192
Cholesteryl esters in medium HDL	IS	rs6606717	12	109873227	2730	0.0061	0.1255
Cholesteryl esters in medium HDL	IS	rs7959043	12	124336478	2793	0.0035	0.0062
Cholesteryl esters in medium HDL	IS	rs11057692	12	125071027	3461	0.0588	0.0097
Cholesteryl esters in medium HDL	IS	rs838876	12	125259888	3347	0.0522	0.0098
Cholesteryl esters in medium HDL	IS	rs61941676	12	125324798	3404	0.0024	0.0045
Cholesteryl esters in medium HDL	IS	rs2494748	14	105258892	2830	0.0020	0.0027
Cholesteryl esters in medium HDL	IS	rs1601935	15	58671765	2999	0.0028	0.1134
Cholesteryl esters in medium HDL	IS	rs1077835	15	58723426	2875	0.0020	0.0105
Cholesteryl esters in medium HDL	IS	rs904770	16	56702000	3147	0.0081	0.2568
Cholesteryl esters in medium HDL	IS	rs75911530	16	57049137	3309	0.0104	0.2567
Cholesteryl esters in medium HDL	IS	rs8058512	16	57151796	3232	0.0103	0.2572
Cholesteryl esters in medium HDL	IS	rs4986970	16	67976320	1300	0.0003	0.0005
Cholesteryl esters in medium HDL	IS	rs2925979	16	81534790	4687	0.0172	0.7821
Cholesteryl esters in medium HDL	IS	rs967645	17	26713970	1135	0.0015	0.0798
Cholesteryl esters in medium HDL	IS	rs72836561	17	41926126	2058	0.0025	0.0073
Cholesteryl esters in medium HDL	IS	rs4969141	17	76391653	3278	0.0545	0.0039
Cholesteryl esters in medium HDL	IS	rs77960347	18	47109955	3089	0.0224	0.7507
Cholesteryl esters in medium HDL	IS	rs117687565	18	47147524	3175	0.0228	0.7496
Cholesteryl esters in medium HDL	IS	rs9304381	18	47158234	3206	0.0229	0.7493
Cholesteryl esters in medium HDL	IS	rs2228671	19	11210912	2454	0.0300	0.9697
Cholesteryl esters in medium HDL	IS	rs12611067	19	11230402	2444	0.0289	0.9708
Cholesteryl esters in medium HDL	IS	rs737337	19	11347493	2454	0.0146	0.9851
Cholesteryl esters in medium HDL	IS	rs429358	19	45411941	2671	0.0028	0.0249
Cholesteryl esters in medium HDL	IS	rs144311893	19	45423944	2682	0.0028	0.0247
Cholesteryl esters in medium HDL	IS	rs2236252	20	17597531	3396	0.0075	0.2277

Cholesteryl esters in medium HDL	IS	rs1800961	20	43042364	2489	0.0050	0.1110
Cholesteryl esters in medium HDL	IS	rs4239651	20	46340596	2735	0.0037	0.0285
Cholesteryl esters in medium HDL	IS	rs2298428	22	21982892	1521	0.0011	0.0032
Total Cholesterol in medium HDL	IS	rs3795269	1	2338126	2542	0.0014	0.0018
Total Cholesterol in medium HDL	IS	rs114165349	1	27021913	1856	0.0040	0.0205
Total Cholesterol in medium HDL	IS	rs3768321	1	40035928	2358	0.0008	0.0011
Total Cholesterol in medium HDL	IS	rs638714	1	62906489	2759	0.0031	0.9796
Total Cholesterol in medium HDL	IS	rs583104	1	109821307	2498	0.0144	0.5488
Total Cholesterol in medium HDL	IS	rs267738	1	150940625	1600	0.0004	0.0007
Total Cholesterol in medium HDL	IS	rs4656292	1	161194641	2678	0.0020	0.0091
Total Cholesterol in medium HDL	IS	rs61805076	1	182154990	2735	0.0033	0.0081
Total Cholesterol in medium HDL	IS	rs2642438	1	220970028	2497	0.0012	0.0014
Total Cholesterol in medium HDL	IS	rs4846921	1	230304352	2909	0.0043	0.0080
Total Cholesterol in medium HDL	IS	rs35135293	2	20363666	3180	0.0036	0.0207
Total Cholesterol in medium HDL	IS	rs676210	2	21231524	2640	0.0019	0.0043
Total Cholesterol in medium HDL	IS	rs1260326	2	27730940	1240	0.0005	0.0061
Total Cholesterol in medium HDL	IS	rs10184004	2	165508389	2195	0.0017	0.0056
Total Cholesterol in medium HDL	IS	rs1047891	2	211540507	2654	0.0031	0.0103
Total Cholesterol in medium HDL	IS	rs2943650	2	227105921	2408	0.0022	0.0096
Total Cholesterol in medium HDL	IS	rs28818616	3	15592561	2420	0.0011	0.0018
Total Cholesterol in medium HDL	IS	rs9647335	3	135880410	1334	0.0005	0.0008
Total Cholesterol in medium HDL	IS	rs13107325	4	103188709	2380	0.0305	0.0092
Total Cholesterol in medium HDL	IS	rs9687846	5	55861894	3386	0.0588	0.0350
Total Cholesterol in medium HDL	IS	rs36057735	6	31319923	9404	0.2761	0.0116
Total Cholesterol in medium HDL	IS	rs28746853	6	32634646	12926	0.0315	0.9322
Total Cholesterol in medium HDL	IS	rs2395943	6	42940673	2054	0.0103	0.9896

Total Cholesterol in medium HDL	IS	rs1358980	6	43764551	2497	0.0094	0.9905
Total Cholesterol in medium HDL	IS	rs9491697	6	127456122	2154	0.0219	0.1242
Total Cholesterol in medium HDL	IS	rs11751347	6	161092438	3023	0.0042	0.2143
Total Cholesterol in medium HDL	IS	rs6967917	7	17984297	3151	0.0041	0.0106
Total Cholesterol in medium HDL	IS	rs12533197	7	36192606	2684	0.0016	0.0034
Total Cholesterol in medium HDL	IS	rs7810507	7	130437476	2212	0.0033	0.0244
Total Cholesterol in medium HDL	IS	rs4240624	8	9184231	4273	0.0042	0.1517
Total Cholesterol in medium HDL	IS	rs59347135	8	19750044	3830	0.0037	0.0090
Total Cholesterol in medium HDL	IS	rs139915535	8	19766233	3844	0.0037	0.0090
Total Cholesterol in medium HDL	IS	rs15285	8	19824667	3887	0.0037	0.0089
Total Cholesterol in medium HDL	IS	rs2245221	8	116624879	1657	0.0187	0.0044
Total Cholesterol in medium HDL	IS	rs686030	9	15304782	3250	0.0026	0.0214
Total Cholesterol in medium HDL	IS	rs11789603	9	107647019	3596	0.0049	0.2402
Total Cholesterol in medium HDL	IS	rs2740488	9	107661742	3595	0.0049	0.2402
Total Cholesterol in medium HDL	IS	rs2792735	10	113921825	2236	0.0013	0.0023
Total Cholesterol in medium HDL	IS	rs60847460	10	113983758	2224	0.0013	0.0024
Total Cholesterol in medium HDL	IS	rs35184771	11	47475189	1531	0.0102	0.1373
Total Cholesterol in medium HDL	IS	rs174578	11	61605499	2270	0.0040	0.1318
Total Cholesterol in medium HDL	IS	rs559355	11	75451281	2033	0.0094	0.0444
Total Cholesterol in medium HDL	IS	rs6589565	11	116640237	3526	0.0056	0.0175
Total Cholesterol in medium HDL	IS	rs78296522	11	116648776	3522	0.0055	0.0175
Total Cholesterol in medium HDL	IS	rs59097294	11	116964437	3450	0.0050	0.0173
Total Cholesterol in medium HDL	IS	rs141368429	11	117221862	3199	0.0638	0.0757
Total Cholesterol in medium HDL	IS	rs7134375	12	20473758	3551	0.0015	0.9976
Total Cholesterol in medium HDL	IS	rs2229357	12	57843711	1489	0.0034	0.0634
Total Cholesterol in medium HDL	IS	rs7956099	12	103556109	2663	0.0729	0.0185

Total Cholesterol in medium HDL	IS	rs6606717	12	109873227	2731	0.0061	0.1255
Total Cholesterol in medium HDL	IS	rs1054852	12	124496316	2925	0.0040	0.0084
Total Cholesterol in medium HDL	IS	rs11057692	12	125071027	3461	0.0035	0.0072
Total Cholesterol in medium HDL	IS	rs921919	12	125265201	3366	0.0028	0.0059
Total Cholesterol in medium HDL	IS	rs7136506	12	125326153	3403	0.0023	0.0045
Total Cholesterol in medium HDL	IS	rs2494748	14	105258892	2830	0.0020	0.0027
Total Cholesterol in medium HDL	IS	rs10162642	15	58577163	3110	0.0030	0.1131
Total Cholesterol in medium HDL	IS	rs1601935	15	58671765	3001	0.0028	0.1134
Total Cholesterol in medium HDL	IS	rs1077835	15	58723426	2876	0.0020	0.0105
Total Cholesterol in medium HDL	IS	rs904770	16	56702000	3146	0.0081	0.2568
Total Cholesterol in medium HDL	IS	rs3764261	16	56993324	3095	0.0037	0.0013
Total Cholesterol in medium HDL	IS	rs75911530	16	57049137	3308	0.1193	0.0041
Total Cholesterol in medium HDL	IS	rs8058512	16	57151796	3231	0.0103	0.2572
Total Cholesterol in medium HDL	IS	rs4986970	16	67976320	1300	0.0003	0.0005
Total Cholesterol in medium HDL	IS	rs2925979	16	81534790	4687	0.0172	0.7821
Total Cholesterol in medium HDL	IS	rs967645	17	26713970	1136	0.0015	0.0798
Total Cholesterol in medium HDL	IS	rs72836561	17	41926126	2058	0.0025	0.0073
Total Cholesterol in medium HDL	IS	rs4969141	17	76391653	3278	0.0030	0.0085
Total Cholesterol in medium HDL	IS	rs77960347	18	47109955	3089	0.0224	0.7507
Total Cholesterol in medium HDL	IS	rs12608026	18	47120499	3106	0.3038	0.0043
Total Cholesterol in medium HDL	IS	rs7241918	18	47160953	3211	0.0230	0.7492
Total Cholesterol in medium HDL	IS	rs2978615	19	11243260	2458	0.0278	0.9719
Total Cholesterol in medium HDL	IS	rs737337	19	11347493	2455	0.0146	0.9851
Total Cholesterol in medium HDL	IS	rs429358	19	45411941	2671	0.0028	0.0249
Total Cholesterol in medium HDL	IS	rs144311893	19	45423944	2682	0.0028	0.0247
Total Cholesterol in medium HDL	IS	rs2236252	20	17597531	3397	0.0075	0.2277

Total Cholesterol in medium HDL	IS	rs1800961	20	43042364	2487	0.0050	0.1110
Total Cholesterol in medium HDL	IS	rs4239651	20	46340596	2734	0.0037	0.0285
Total Cholesterol in medium HDL	IS	rs2298428	22	21982892	1521	0.0011	0.0032
Total Concentration of medium HDL particles	IS	rs3795269	1	2338126	2542	0.0014	0.0019
Total Concentration of medium HDL particles	IS	rs193084249	1	26987646	1854	0.0041	0.0206
Total Concentration of medium HDL particles	IS	rs3768321	1	40035928	2359	0.0290	0.0049
Total Concentration of medium HDL particles	IS	rs638714	1	62906489	2759	0.0031	0.9796
Total Concentration of medium HDL particles	IS	rs583104	1	109821307	2498	0.0144	0.5488
Total Concentration of medium HDL particles	IS	rs267738	1	150940625	1600	0.0004	0.0007
Total Concentration of medium HDL particles	IS	rs61805076	1	182154990	2735	0.0033	0.0081
Total Concentration of medium HDL particles	IS	rs2642438	1	220970028	2497	0.0012	0.0014
Total Concentration of medium HDL particles	IS	rs1321257	1	230305312	2907	0.0043	0.0080
Total Concentration of medium HDL particles	IS	rs35135293	2	20363666	3179	0.0036	0.0207
Total Concentration of medium HDL particles	IS	rs676210	2	21231524	2641	0.0438	0.0106
Total Concentration of medium HDL particles	IS	rs1260326	2	27730940	1239	0.0218	0.0055
Total Concentration of medium HDL particles	IS	rs10184004	2	165508389	2194	0.0017	0.0056
Total Concentration of medium HDL particles	IS	rs1047891	2	211540507	2654	0.0031	0.0103
Total Concentration of medium HDL particles	IS	rs2176040	2	227092802	2400	0.0022	0.0096
Total Concentration of medium HDL particles	IS	rs28818616	3	15592561	2419	0.0011	0.0018
Total Concentration of medium HDL particles	IS	rs13107325	4	103188709	2381	0.0009	0.0034
Total Concentration of medium HDL particles	IS	rs36057735	6	31319923	9412	0.0366	0.5251
Total Concentration of medium HDL particles	IS	rs112495680	6	34223098	3107	0.0037	0.0186
Total Concentration of medium HDL particles	IS	rs9471972	6	42915021	2055	0.9957	0.0003
Total Concentration of medium HDL particles	IS	rs9491697	6	127456122	2154	0.0219	0.1242
Total Concentration of medium HDL particles	IS	rs7783857	7	130439058	2210	0.0034	0.0245
Total Concentration of medium HDL particles	IS	rs4240624	8	9184231	4270	0.0042	0.1517

Total Concentration of medium HDL particles	IS	rs59347135	8	19750044	3831	0.0037	0.0090
Total Concentration of medium HDL particles	IS	rs139915535	8	19766233	3845	0.0037	0.0090
Total Concentration of medium HDL particles	IS	rs15285	8	19824667	3888	0.0037	0.0089
Total Concentration of medium HDL particles	IS	rs2245221	8	116624879	1657	0.0004	0.0006
Total Concentration of medium HDL particles	IS	rs686030	9	15304782	3248	0.0026	0.0214
Total Concentration of medium HDL particles	IS	rs2066714	9	107586753	3614	0.0065	0.3597
Total Concentration of medium HDL particles	IS	rs11789603	9	107647019	3591	0.0049	0.2402
Total Concentration of medium HDL particles	IS	rs2740488	9	107661742	3590	0.0049	0.2402
Total Concentration of medium HDL particles	IS	rs2792735	10	113921825	2237	0.0013	0.0023
Total Concentration of medium HDL particles	IS	rs60847460	10	113983758	2225	0.0013	0.0024
Total Concentration of medium HDL particles	IS	rs35184771	11	47475189	1532	0.0102	0.1373
Total Concentration of medium HDL particles	IS	rs174578	11	61605499	2270	0.0040	0.1318
Total Concentration of medium HDL particles	IS	rs559355	11	75451281	2033	0.0094	0.0444
Total Concentration of medium HDL particles	IS	rs625145	11	116727936	3350	0.0051	0.0173
Total Concentration of medium HDL particles	IS	rs112771035	11	126225876	3230	0.0019	0.0038
Total Concentration of medium HDL particles	IS	rs2229357	12	57843711	1489	0.0034	0.0634
Total Concentration of medium HDL particles	IS	rs7956099	12	103556109	2662	0.0741	0.0190
Total Concentration of medium HDL particles	IS	rs6606717	12	109873227	2730	0.0061	0.1255
Total Concentration of medium HDL particles	IS	rs7959043	12	124336478	2796	0.0588	0.0047
Total Concentration of medium HDL particles	IS	rs838876	12	125259888	3349	0.0028	0.0059
Total Concentration of medium HDL particles	IS	rs61941676	12	125324798	3405	0.0024	0.0045
Total Concentration of medium HDL particles	IS	rs2494748	14	105258892	2830	0.0020	0.0028
Total Concentration of medium HDL particles	IS	rs12904367	15	58547878	3114	0.0030	0.1131
Total Concentration of medium HDL particles	IS	rs2043085	15	58680954	2986	0.0021	0.0106
Total Concentration of medium HDL particles	IS	rs1077835	15	58723426	2871	0.0020	0.0105
Total Concentration of medium HDL particles	IS	rs3764261	16	56993324	3624	0.0152	0.0100

Total Concentration of medium HDL particles	IS	rs75911530	16	57049137	3308	0.0104	0.2567
Total Concentration of medium HDL particles	IS	rs8058512	16	57151796	3231	0.0103	0.2572
Total Concentration of medium HDL particles	IS	rs4986970	16	67976320	1300	0.0003	0.0005
Total Concentration of medium HDL particles	IS	rs2925979	16	81534790	4685	0.2817	0.0173
Total Concentration of medium HDL particles	IS	rs967645	17	26713970	1135	0.0015	0.0798
Total Concentration of medium HDL particles	IS	rs72836561	17	41926126	2058	0.0025	0.0073
Total Concentration of medium HDL particles	IS	rs4969141	17	76391653	3278	0.0030	0.0085
Total Concentration of medium HDL particles	IS	rs77960347	18	47109955	3089	0.0224	0.7507
Total Concentration of medium HDL particles	IS	rs62101704	18	47134250	3133	0.0227	0.7500
Total Concentration of medium HDL particles	IS	rs117687565	18	47147524	3175	0.0228	0.7496
Total Concentration of medium HDL particles	IS	rs9304381	18	47158234	3206	0.0229	0.7493
Total Concentration of medium HDL particles	IS	rs12976739	19	8461663	2770	0.0016	0.0021
Total Concentration of medium HDL particles	IS	rs76213248	19	11269893	2472	0.0212	0.9785
Total Concentration of medium HDL particles	IS	rs737338	19	11347657	2456	0.0146	0.9851
Total Concentration of medium HDL particles	IS	rs2236252	20	17597531	3396	0.0075	0.2277
Total Concentration of medium HDL particles	IS	rs1800961	20	43042364	2488	0.0050	0.1110
Total Concentration of medium HDL particles	IS	rs4239651	20	46340596	2735	0.0037	0.0285
Total Concentration of medium HDL particles	IS	rs3859588	20	46476143	3044	0.0038	0.0248
Total Concentration of medium HDL particles	IS	rs235314	21	46271452	3243	0.0036	0.0105
Total Concentration of medium HDL particles	IS	rs2298428	22	21982892	1521	0.0011	0.0032
Total lipids in medium HDL	IS	rs3795269	1	2338126	2542	0.0014	0.0019
Total lipids in medium HDL	IS	rs193084249	1	26987646	1853	0.0041	0.0206
Total lipids in medium HDL	IS	rs3768321	1	40035928	2358	0.0008	0.0011
Total lipids in medium HDL	IS	rs638714	1	62906489	2760	0.0031	0.9796
Total lipids in medium HDL	IS	rs583104	1	109821307	2498	0.0144	0.5488
Total lipids in medium HDL	IS	rs267738	1	150940625	1600	0.0004	0.0007

Total lipids in medium HDL	IS	rs4656292	1	161194641	2678	0.0020	0.0091
Total lipids in medium HDL	IS	rs61805076	1	182154990	2735	0.0033	0.0081
Total lipids in medium HDL	IS	rs2642438	1	220970028	2497	0.0012	0.0014
Total lipids in medium HDL	IS	rs1321257	1	230305312	2905	0.0043	0.0080
Total lipids in medium HDL	IS	rs35135293	2	20363666	3181	0.0609	0.0038
Total lipids in medium HDL	IS	rs676210	2	21231524	2640	0.0019	0.0043
Total lipids in medium HDL	IS	rs1260326	2	27730940	1240	0.0005	0.0061
Total lipids in medium HDL	IS	rs1047891	2	211540507	2655	0.0031	0.0103
Total lipids in medium HDL	IS	rs2176040	2	227092802	2401	0.0022	0.0096
Total lipids in medium HDL	IS	rs28818616	3	15592561	2418	0.0011	0.0018
Total lipids in medium HDL	IS	rs13107325	4	103188709	2382	0.0009	0.0034
Total lipids in medium HDL	IS	rs36057735	6	31319923	9410	0.0366	0.5251
Total lipids in medium HDL	IS	rs112495680	6	34223098	3107	0.0037	0.0186
Total lipids in medium HDL	IS	rs9471972	6	42915021	2054	0.0103	0.9896
Total lipids in medium HDL	IS	rs4240624	8	9184231	4270	0.0042	0.1517
Total lipids in medium HDL	IS	rs59347135	8	19750044	3832	0.0037	0.0090
Total lipids in medium HDL	IS	rs139915535	8	19766233	3846	0.0037	0.0090
Total lipids in medium HDL	IS	rs15285	8	19824667	3889	0.0037	0.0089
Total lipids in medium HDL	IS	rs686030	9	15304782	3249	0.0026	0.0214
Total lipids in medium HDL	IS	rs11789603	9	107647019	3594	0.0049	0.2402
Total lipids in medium HDL	IS	rs2740488	9	107661742	3593	0.0049	0.2402
Total lipids in medium HDL	IS	rs2792735	10	113921825	2239	0.0353	0.0075
Total lipids in medium HDL	IS	rs60847460	10	113983758	2227	0.0361	0.0074
Total lipids in medium HDL	IS	rs11601507	11	5701074	5088	0.0050	0.0078
Total lipids in medium HDL	IS	rs11039238	11	47460901	1513	0.0102	0.1374
Total lipids in medium HDL	IS	rs2072113	11	61604967	2266	0.0040	0.1318

Total lipids in medium HDL	IS	rs559355	11	75451281	2034	0.0776	0.2179
Total lipids in medium HDL	IS	rs625145	11	116727936	3348	0.0050	0.0173
Total lipids in medium HDL	IS	rs7956099	12	103556109	2663	0.0056	0.0192
Total lipids in medium HDL	IS	rs6606717	12	109873227	2730	0.0061	0.1255
Total lipids in medium HDL	IS	rs838876	12	125259888	3347	0.0028	0.0059
Total lipids in medium HDL	IS	rs61941676	12	125324798	3404	0.0024	0.0045
Total lipids in medium HDL	IS	rs12904367	15	58547878	3122	0.0030	0.1131
Total lipids in medium HDL	IS	rs2043085	15	58680954	2994	0.0021	0.0106
Total lipids in medium HDL	IS	rs1077835	15	58723426	2878	0.0020	0.0105
Total lipids in medium HDL	IS	rs3764261	16	56993324	3625	0.0190	0.0134
Total lipids in medium HDL	IS	rs75911530	16	57049137	3307	0.0104	0.2567
Total lipids in medium HDL	IS	rs4986970	16	67976320	1300	0.0003	0.0005
Total lipids in medium HDL	IS	rs2925979	16	81534790	4687	0.0172	0.7821
Total lipids in medium HDL	IS	rs967645	17	26713970	1135	0.0015	0.0798
Total lipids in medium HDL	IS	rs72836561	17	41926126	2060	0.0025	0.0073
Total lipids in medium HDL	IS	rs4969141	17	76391653	3277	0.0030	0.0085
Total lipids in medium HDL	IS	rs77960347	18	47109955	3089	0.0224	0.7507
Total lipids in medium HDL	IS	rs62101704	18	47134250	3133	0.0227	0.7500
Total lipids in medium HDL	IS	rs117687565	18	47147524	3175	0.0228	0.7496
Total lipids in medium HDL	IS	rs9304381	18	47158234	3206	0.0229	0.7493
Total lipids in medium HDL	IS	rs12976739	19	8461663	2768	0.0016	0.0021
Total lipids in medium HDL	IS	rs6511720	19	11202306	2450	0.9949	0.0000
Total lipids in medium HDL	IS	rs2569550	19	11228745	2448	0.0296	0.9701
Total lipids in medium HDL	IS	rs737338	19	11347657	2456	0.0146	0.9851
Total lipids in medium HDL	IS	rs2236252	20	17597531	3397	0.0075	0.2277
Total lipids in medium HDL	IS	rs1800961	20	43042364	2487	0.0050	0.1110

Total lipids in medium HDL	IS	rs4239651	20	46340596	2734	0.0037	0.0285
Total lipids in medium HDL	IS	rs3859588	20	46476143	3042	0.0621	0.0099
Total lipids in medium HDL	IS	rs235314	21	46271452	3243	0.0036	0.0105
Total lipids in medium HDL	IS	rs2298428	22	21982892	1520	0.0011	0.0032

Chr denotes chromosome; BP, base pair position; PP.H3 denotes association with CAD or IS risk as well as (apo)lipoprotein measurements, but at distinct causal variants; PP.H4 denotes association with both traits, with a shared causal variant. A PP.H4 greater than 0.8 indicates significant colocalization.

Table S11. Circulating genes associated with shared genetic variant between HDL subclasses and CAD/IS retrieved from eQTLGen database.

Outcome	SNP	Chr	BP	Effect Allele	Alternate Allele	EAF	Gene	Gene_Chr	Gene_BP	Beta	SE	*pval*
CAD	rs1054852	12	124496316	G	A	0.347043	TCTN2	12	124174304	-0.036	0.008	1.70e-05
CAD	rs1054852	12	124496316	G	A	0.347043	ATP6V0A2	12	124221207	-0.029	0.008	0.001
CAD	rs1054852	12	124496316	G	A	0.347043	ENSG00000270028	12	124410319	0.186	0.021	4.13e-19
CAD	rs1054852	12	124496316	G	A	0.347043	ENSG00000250091	12	124415251	0.046	0.010	4.43e-06
CAD	rs1054852	12	124496316	G	A	0.347043	CCDC92	12	124430292	0.107	0.008	9.93e-37
CAD	rs1054852	12	124496316	G	A	0.347043	ZNF664	12	124478189	-0.157	0.008	6.21e-77
CAD	rs114165349	1	27021913	C	G	0.0365514	SH3BGRL3	1	26606837	-0.053	0.026	0.041
CAD	rs114165349	1	27021913	C	G	0.0365514	ARID1A	1	27065559	-0.853	0.025	7.02e-247
CAD	rs114165349	1	27021913	C	G	0.0365514	GPN2	1	27211280	-0.076	0.026	0.004
CAD	rs11591147	1	55505647	TRUE	G	0.0202403	USP24	1	55606535	0.093	0.042	0.025
CAD	rs11601507	11	5701074	A	C	0.0718798	HBG2	11	5470719	0.077	0.029	0.007
CAD	rs11601507	11	5701074	A	C	0.0718798	UBQLNL	11	5536779	-0.254	0.017	2.08e-51
CAD	rs11601507	11	5701074	A	C	0.0718798	ENSG00000224295	11	5542928	-0.133	0.041	0.001
CAD	rs11601507	11	5701074	A	C	0.0718798	TRIM6	11	5625763	-0.084	0.017	8.65e-07
CAD	rs11601507	11	5701074	A	C	0.0718798	TRIM34	11	5653311	-0.038	0.017	0.028
CAD	rs11601507	11	5701074	A	C	0.0718798	TRIM5	11	5822137	0.097	0.017	1.04e-08
CAD	rs11601507	11	5701074	A	C	0.0718798	FHIP1B	11	6244253	0.035	0.017	0.041
CAD	rs116843064	19	8429323	A	G	0.0201278	RPS28	19	8387133	-0.253	0.067	1.75e-04
CAD	rs116843064	19	8429323	A	G	0.0201278	MYO1F	19	8614067	0.093	0.031	0.003
CAD	rs116843064	19	8429323	A	G	0.0201278	ZNF558	19	8931692	-0.154	0.067	0.022
CAD	rs117310449	19	45393516	TRUE	C	0.0130585	IGSF23	19	45128510	0.183	0.078	0.019
CAD	rs117310449	19	45393516	TRUE	C	0.0130585	NECTIN2	19	45370958	1.142	0.059	7.35e-83
CAD	rs117310449	19	45393516	TRUE	C	0.0130585	CLASRP	19	45558256	0.349	0.119	0.003
CAD	rs12976739	19	8461663	A	G	0.404947	CD320	19	8370125	-0.059	0.008	4.63e-13

CAD	rs12976739	19	8461663	A	G	0.404947	MARCHF2	19	8491027	0.022	0.008	0.006
CAD	rs12976739	19	8461663	A	G	0.404947	CERS4	19	8299462	0.026	0.009	0.004
CAD	rs12976739	19	8461663	A	G	0.404947	NDUFA7	19	8379885	0.019	0.009	0.034
CAD	rs12976739	19	8461663	A	G	0.404947	RAB11B	19	8462091	-0.036	0.008	9.19e-06
CAD	rs12976739	19	8461663	A	G	0.404947	OR7D2	19	9297886	0.020	0.009	0.028
CAD	rs1321257	1	230305312	G	A	0.408451	GALNT2	1	230305703	-0.043	0.008	1.41e-07
CAD	rs1321257	1	230305312	G	A	0.408451	PGBD5	1	230509433	0.020	0.008	0.016
CAD	rs1358980	6	43764551	TRUE	C	0.478378	PEX6	6	42939283	0.064	0.009	1.92e-12
CAD	rs1358980	6	43764551	TRUE	C	0.478378	MRPS18A	6	43647284	-0.021	0.009	0.024
CAD	rs139915535	8	19766233	G	A	0.0181211	LZTS1	8	20132575	0.068	0.032	0.033
CAD	rs15285	8	19824667	TRUE	C	0.27489	CSGALNACT1	8	19438606	0.026	0.009	0.003
CAD	rs15285	8	19824667	TRUE	C	0.27489	INTS10	8	19692122	0.030	0.009	0.001
CAD	rs15285	8	19824667	TRUE	C	0.27489	LPL	8	19791998	0.196	0.009	3.96e-108
CAD	rs15285	8	19824667	TRUE	C	0.27489	SLC18A1	8	20021541	0.026	0.009	0.003
CAD	rs15285	8	19824667	TRUE	C	0.27489	ATP6V1B2	8	20069604	0.035	0.009	9.49e-05
CAD	rs193084249	1	26987646	G	A	0.0280541	MAN1C1	1	26028328	0.079	0.030	0.009
CAD	rs193084249	1	26987646	G	A	0.0280541	CEP85	1	26582995	0.237	0.031	2.80e-14
CAD	rs193084249	1	26987646	G	A	0.0280541	CD52	1	26645731	-0.155	0.027	1.16e-08
CAD	rs193084249	1	26987646	G	A	0.0280541	ZNF683	1	26694569	-0.089	0.027	0.001
CAD	rs193084249	1	26987646	G	A	0.0280541	LOC101928728	1	27019622	-0.180	0.031	8.92e-09
CAD	rs193084249	1	26987646	G	A	0.0280541	PIGV	1	27119426	-0.529	0.027	1.11e-85
CAD	rs193084249	1	26987646	G	A	0.0280541	ZDHHC18	1	27168647	0.613	0.027	3.17e-115
CAD	rs193084249	1	26987646	G	A	0.0280541	SYTL1	1	27674467	-0.380	0.041	3.99e-20
CAD	rs193084249	1	26987646	G	A	0.0280541	MAP3K6	1	27687529	-0.248	0.027	5.12e-20
CAD	rs193084249	1	26987646	G	A	0.0280541	FGR	1	27950181	-0.061	0.027	0.026
CAD	rs193084249	1	26987646	G	A	0.0280541	STMN1	1	26222077	0.078	0.030	0.010

CAD	rs193084249	1	26987646	G	A	0.0280541	CNKSR1	1	26510135	-0.108	0.027	7.10e-05
CAD	rs193084249	1	26987646	G	A	0.0280541	HMGN2	1	26800702	0.099	0.030	0.001
CAD	rs193084249	1	26987646	G	A	0.0280541	TRNP1	1	27323793	-0.055	0.027	0.045
CAD	rs2176040	2	227092802	A	G	0.360534	IRS1	2	227632116	-0.025	0.008	0.003
CAD	rs2229357	12	57843711	A	G	0.231306	NEMP1	12	57465636	0.084	0.010	3.09e-16
CAD	rs2229357	12	57843711	A	G	0.231306	STAT6	12	57507556	-0.094	0.009	4.81e-23
CAD	rs2229357	12	57843711	A	G	0.231306	LRP1	12	57564705	0.031	0.009	0.001
CAD	rs2229357	12	57843711	A	G	0.231306	NDUFA4L2	12	57631592	0.033	0.010	0.001
CAD	rs2229357	12	57843711	A	G	0.231306	ARHGAP9	12	57874317	0.037	0.009	1.04e-04
CAD	rs2229357	12	57843711	A	G	0.231306	MARS1	12	57890290	-0.076	0.014	5.99e-08
CAD	rs2229357	12	57843711	A	G	0.231306	DDIT3	12	57912335	-0.037	0.009	1.11e-04
CAD	rs2229357	12	57843711	A	G	0.231306	MBD6	12	57919212	-0.020	0.009	0.036
CAD	rs2229357	12	57843711	A	G	0.231306	DCTN2	12	57932499	-0.019	0.009	0.045
CAD	rs2229357	12	57843711	A	G	0.231306	PIP4K2C	12	57991077	-0.022	0.009	0.022
CAD	rs2229357	12	57843711	A	G	0.231306	ENSG00000257499	12	58118496	-0.045	0.023	0.047
CAD	rs2229357	12	57843711	A	G	0.231306	TSPAN31	12	58137895	0.029	0.009	0.002
CAD	rs2229357	12	57843711	A	G	0.231306	EEF1AKMT3	12	58170799	0.086	0.011	2.63e-16
CAD	rs2229357	12	57843711	A	G	0.231306	AVIL	12	58201823	0.053	0.014	1.40e-04
CAD	rs2229357	12	57843711	A	G	0.231306	GIHCG	12	58327634	-0.078	0.023	0.001
CAD	rs2229357	12	57843711	A	G	0.231306	ATP23	12	58343188	-0.092	0.014	5.87e-11
CAD	rs2236252	20	17597531	TRUE	C	0.180448	BFSP1	20	17512207	0.032	0.010	0.002
CAD	rs2236252	20	17597531	TRUE	C	0.180448	DSTN	20	17569697	-0.185	0.010	1.81e-72
CAD	rs2236252	20	17597531	TRUE	C	0.180448	RRBP1	20	17628631	-0.171	0.010	6.59e-62
CAD	rs2236252	20	17597531	TRUE	C	0.180448	MGME1	20	17960660	0.052	0.012	5.82e-06
CAD	rs3764261	16	56993324	A	C	0.317026	GNAO1	16	56308329	0.020	0.009	0.017
CAD	rs3764261	16	56993324	A	C	0.317026	NUDT21	16	56474578	0.026	0.009	0.002

CAD	rs3764261	16	56993324	A	C	0.317026	MT1F	16	56693108	0.021	0.009	0.012
CAD	rs3764261	16	56993324	A	C	0.317026	SLC12A3	16	56924440	-0.103	0.013	6.42e-16
CAD	rs3764261	16	56993324	A	C	0.317026	HERPUD1	16	56971879	-0.056	0.009	5.22e-11
CAD	rs3764261	16	56993324	A	C	0.317026	CETP	16	57006759	-0.085	0.009	3.56e-23
CAD	rs3764261	16	56993324	A	C	0.317026	NLRC5	16	57070420	-0.069	0.013	7.07e-08
CAD	rs3764261	16	56993324	A	C	0.317026	ENSG00000260145	16	57089413	-0.060	0.022	0.007
CAD	rs3764261	16	56993324	A	C	0.317026	COQ9	16	57488262	-0.020	0.009	0.019
CAD	rs3764261	16	56993324	A	C	0.317026	ADGRG3	16	57713037	0.020	0.009	0.021
CAD	rs583104	1	109821307	G	TRUE	0.232199	EEIG2	1	109145116	0.028	0.010	0.008
CAD	rs583104	1	109821307	G	TRUE	0.232199	CLCC1	1	109489120	0.036	0.010	1.76e-04
CAD	rs583104	1	109821307	G	TRUE	0.232199	ELAPOR1	1	109702851	-0.143	0.010	1.62e-50
CAD	rs583104	1	109821307	G	TRUE	0.232199	PSRC1	1	109823993	0.469	0.009	7.02e-250
CAD	rs583104	1	109821307	G	TRUE	0.232199	CYB561D1	1	110041114	-0.023	0.010	0.029
CAD	rs583104	1	109821307	G	TRUE	0.232199	AMIGO1	1	110049578	0.061	0.009	1.28e-10
CAD	rs583104	1	109821307	G	TRUE	0.232199	ENSG00000225113	1	110139625	-0.048	0.023	0.032
CAD	rs583104	1	109821307	G	TRUE	0.232199	GSTM1	1	110240871	-0.074	0.011	1.15e-11
CAD	rs72836561	17	41926126	TRUE	C	0.0248146	IFI35	17	41162607	-0.188	0.028	3.11e-11
CAD	rs72836561	17	41926126	TRUE	C	0.0248146	BRCA1	17	41259301	0.087	0.028	0.002
CAD	rs72836561	17	41926126	TRUE	C	0.0248146	NBR2	17	41291657	-0.090	0.028	0.002
CAD	rs72836561	17	41926126	TRUE	C	0.0248146	MPP2	17	41969896	0.277	0.028	1.24e-22
CAD	rs72836561	17	41926126	TRUE	C	0.0248146	TMEM101	17	42094935	0.070	0.028	0.013
CAD	rs72836561	17	41926126	TRUE	C	0.0248146	ASB16-AS1	17	42258713	0.121	0.032	1.92e-04
CAD	rs72836561	17	41926126	TRUE	C	0.0248146	TMUB2	17	42266718	0.093	0.028	0.001
CAD	rs72836561	17	41926126	TRUE	C	0.0248146	UBTF	17	42290697	-0.072	0.028	0.011
CAD	rs72836561	17	41926126	TRUE	C	0.0248146	GRN	17	42426542	0.088	0.028	0.002
CAD	rs75911530	16	57049137	A	G	0.0248805	ENSG00000261114	16	56975289	-0.210	0.066	0.002

CAD	rs75911530	16	57049137	A	G	0.0248805	ADGRG5	16	57600963	0.080	0.029	0.006
CAD	rs7959043	12	124336478	G	A	0.393851	KMT5A	12	123881112	-0.037	0.015	0.015
CAD	rs7959043	12	124336478	G	A	0.393851	TMED2	12	124076097	-0.033	0.008	6.90e-05
CAD	rs7959043	12	124336478	G	A	0.393851	EIF2B1	12	124111633	-0.083	0.008	3.29e-24
CAD	rs7959043	12	124336478	G	A	0.393851	ARL6IP4	12	123465894	0.020	0.008	0.013
CAD	rs7959043	12	124336478	G	A	0.393851	RILPL2	12	123910600	-0.046	0.009	1.86e-07
CAD	rs7959043	12	124336478	G	A	0.393851	SNRNP35	12	123949944	-0.025	0.009	0.004
CAD	rs7959043	12	124336478	G	A	0.393851	GTF2H3	12	124132764	0.019	0.008	0.020
CAD	rs8058512	16	57151796	C	TRUE	0.304097	RSPRY1	16	57247218	-0.060	0.009	2.53e-10
CAD	rs8058512	16	57151796	C	TRUE	0.304097	CPNE2	16	57154163	0.043	0.009	5.65e-06
CAD	rs8058512	16	57151796	C	TRUE	0.304097	PSME3IP1	16	57203203	-0.062	0.010	5.41e-11
CAD	rs8058512	16	57151796	C	TRUE	0.304097	ARL2BP	16	57283263	-0.042	0.009	1.07e-05
CAD	rs8058512	16	57151796	C	TRUE	0.304097	PLLP	16	57304301	-0.050	0.010	1.51e-07
CAD	rs904770	16	56702000	TRUE	C	0.112023	BBS2	16	56527471	0.093	0.013	2.43e-13
CAD	rs904770	16	56702000	TRUE	C	0.112023	MT1E	16	56660205	0.117	0.014	3.70e-17
CAD	rs904770	16	56702000	TRUE	C	0.112023	MT1X	16	56717222	0.217	0.013	7.19e-66
CAD	rs904770	16	56702000	TRUE	C	0.112023	NUP93-DT	16	56753282	0.113	0.037	0.002
CAD	rs904770	16	56702000	TRUE	C	0.112023	MT3	16	56623993	0.047	0.013	2.46e-04
CAD	rs904770	16	56702000	TRUE	C	0.112023	ENSG00000260828	16	57637329	0.086	0.030	0.005
CAD	rs904770	16	56702000	TRUE	C	0.112023	ENSG00000205361	16	56678157	0.032	0.013	0.016
CAD	rs9647335	3	135880410	TRUE	A	0.167611	PPP2R3A	3	135775624	-0.035	0.011	0.001
CAD	rs9647335	3	135880410	TRUE	A	0.167611	PCCB	3	136012943	-0.274	0.011	3.64e-149
CAD	rs9687846	5	55861894	A	G	0.182482	LINC01948	5	55765697	-0.038	0.012	0.002
CAD	rs9687846	5	55861894	A	G	0.182482	ENSG00000234553	5	55833031	-0.102	0.028	2.39e-04
CAD	rs9687846	5	55861894	A	G	0.182482	GPBP1	5	56515140	-0.036	0.010	4.65e-04
IS	rs12611067	19	11230402	TRUE	G	0.350883	ICAM3	19	10447475	-0.027	0.009	0.003

IS	rs12611067	19	11230402	TRUE	G	0.350883	KEAP1	19	10605606	-0.019	0.009	0.037
IS	rs12611067	19	11230402	TRUE	G	0.350883	AP1M2	19	10690669	0.027	0.009	0.003
IS	rs12611067	19	11230402	TRUE	G	0.350883	ZNF441	19	11886354	0.018	0.009	0.040
IS	rs12611067	19	11230402	TRUE	G	0.350883	ZNF491	19	11914459	-0.028	0.009	0.002
IS	rs12611067	19	11230402	TRUE	G	0.350883	ZNF20	19	12227440	-0.040	0.020	0.043
IS	rs2228671	19	11210912	TRUE	C	0.106845	CARM1	19	11007821	0.048	0.013	2.35e-04
IS	rs2228671	19	11210912	TRUE	C	0.106845	S1PR5	19	10626115	0.047	0.013	3.32e-04
IS	rs2228671	19	11210912	TRUE	C	0.106845	SLC44A2	19	10734184	-0.051	0.013	7.87e-05
IS	rs2228671	19	11210912	TRUE	C	0.106845	C19orf38	19	10963858	-0.046	0.016	0.004
IS	rs2228671	19	11210912	TRUE	C	0.106845	TIMM29	19	11041810	-0.058	0.013	6.36e-06
IS	rs2228671	19	11210912	TRUE	C	0.106845	SMARCA4	19	11123834	-0.085	0.013	3.82e-11
IS	rs2228671	19	11210912	TRUE	C	0.106845	ICAM1	19	10389401	0.059	0.013	4.94e-06
IS	rs2228671	19	11210912	TRUE	C	0.106845	PDE4A	19	10553877	0.042	0.013	0.001
IS	rs2228671	19	11210912	TRUE	C	0.106845	ILF3-DT	19	10763529	-0.059	0.018	0.001
IS	rs2228671	19	11210912	TRUE	C	0.106845	ACP5	19	11687649	0.038	0.013	0.003
IS	rs2395943	6	42940673	A	G	0.431865	MRPS18A	6	43647284	0.021	0.008	0.010
IS	rs2395943	6	42940673	A	G	0.431865	CCND3	6	41960383	-0.027	0.012	0.025
IS	rs2395943	6	42940673	A	G	0.431865	BICRAL	6	42793031	-0.102	0.009	1.36e-29
IS	rs2395943	6	42940673	A	G	0.431865	GNMT	6	42930057	0.434	0.011	4.00e-248
IS	rs2395943	6	42940673	A	G	0.431865	PPP2R5D	6	42966158	0.108	0.008	1.53e-41
IS	rs2395943	6	42940673	A	G	0.431865	KLHDC3	6	42985493	0.038	0.008	1.80e-06
IS	rs2395943	6	42940673	A	G	0.431865	PTCRA	6	42888650	0.025	0.008	0.002
IS	rs2395943	6	42940673	A	G	0.431865	CUL7	6	43013519	0.029	0.008	3.07e-04
IS	rs2395943	6	42940673	A	G	0.431865	DLK2	6	43421230	-0.017	0.008	0.035
IS	rs2569550	19	11228745	TRUE	C	0.409224	KRI1	19	10670237	0.043	0.010	8.65e-06
IS	rs2569550	19	11228745	TRUE	C	0.409224	TMED1	19	10945054	0.029	0.009	0.001

IS	rs2569550	19	11228745	TRUE	C	0.409224	SPC24	19	11254340	-0.032	0.009	2.00e-04
IS	rs2569550	19	11228745	TRUE	C	0.409224	TSPAN16	19	11422248	0.025	0.010	0.012
IS	rs28746853	6	32634646	C	TRUE	0.179075	LY6G5C	6	31648139	-0.054	0.020	0.006
IS	rs28746853	6	32634646	C	TRUE	0.179075	DDAH2	6	31696604	-0.039	0.020	0.049
IS	rs28746853	6	32634646	C	TRUE	0.179075	MSH5	6	31720173	0.079	0.030	0.009
IS	rs28746853	6	32634646	C	TRUE	0.179075	VWA7	6	31739237	0.110	0.035	0.002
IS	rs28746853	6	32634646	C	TRUE	0.179075	C4A	6	31960129	-0.076	0.021	2.57e-04
IS	rs28746853	6	32634646	C	TRUE	0.179075	C4B	6	31992867	0.131	0.035	1.85e-04
IS	rs28746853	6	32634646	C	TRUE	0.179075	TNXB	6	32046021	0.067	0.030	0.027
IS	rs28746853	6	32634646	C	TRUE	0.179075	RNF5	6	32149030	-0.098	0.035	0.005
IS	rs28746853	6	32634646	C	TRUE	0.179075	AGER	6	32150423	0.065	0.020	0.001
IS	rs28746853	6	32634646	C	TRUE	0.179075	HLA-DRB5	6	32491592	-0.261	0.014	1.28e-80
IS	rs28746853	6	32634646	C	TRUE	0.179075	ENSG00000229391	6	32524144	-0.333	0.012	1.32e-166
IS	rs28746853	6	32634646	C	TRUE	0.179075	HLA-DRB1	6	32552085	-0.665	0.033	2.78e-92
IS	rs28746853	6	32634646	C	TRUE	0.179075	HLA-DQB1-AS1	6	32628081	-0.504	0.037	1.14e-41
IS	rs28746853	6	32634646	C	TRUE	0.179075	HLA-DQB1	6	32631702	-0.328	0.014	2.32e-120
IS	rs28746853	6	32634646	C	TRUE	0.179075	HLA-DQA2	6	32712055	0.884	0.031	6.19e-182
IS	rs28746853	6	32634646	C	TRUE	0.179075	HLA-DQB2	6	32727593	0.229	0.021	1.27e-28
IS	rs28746853	6	32634646	C	TRUE	0.179075	HLA-DOB	6	32782682	0.109	0.021	1.50e-07
IS	rs28746853	6	32634646	C	TRUE	0.179075	TAP2	6	32798083	-0.106	0.020	6.64e-08
IS	rs28746853	6	32634646	C	TRUE	0.179075	PSMB9	6	32819637	-0.076	0.020	1.16e-04
IS	rs28746853	6	32634646	C	TRUE	0.179075	ENSG00000234515	6	32847286	-0.061	0.020	0.003
IS	rs28746853	6	32634646	C	TRUE	0.179075	HLA-DMA	6	32926630	-0.047	0.021	0.025
IS	rs28746853	6	32634646	C	TRUE	0.179075	HLA-DPB1	6	33049340	-0.071	0.021	0.001
IS	rs28746853	6	32634646	C	TRUE	0.179075	VPS52	6	33228936	0.045	0.020	0.023
IS	rs2978615	19	11243260	C	TRUE	0.537367	KANK2	19	11291705	-0.039	0.010	4.72e-05

IS	rs2978615	19	11243260	C	TRUE	0.537367	LDLR	19	11222265	0.032	0.010	0.001
IS	rs2978615	19	11243260	C	TRUE	0.537367	ENSG00000226104	19	10751904	-0.117	0.047	0.013
IS	rs2978615	19	11243260	C	TRUE	0.537367	SWSAP1	19	11486494	0.031	0.011	0.005
IS	rs2978615	19	11243260	C	TRUE	0.537367	EPOR	19	11491627	0.032	0.010	0.001
IS	rs737337	19	11347493	C	TRUE	0.0945724	DOCK6	19	11341564	-0.028	0.014	0.043
IS	rs737337	19	11347493	C	TRUE	0.0945724	RGL3	19	11512517	-0.107	0.025	2.14e-05
IS	rs737337	19	11347493	C	TRUE	0.0945724	PRKCSH	19	11553946	-0.042	0.014	0.002
IS	rs737338	19	11347657	TRUE	C	0.0563413	ENSG00000267174	19	11423285	-0.447	0.041	9.41e-28
IS	rs737338	19	11347657	TRUE	C	0.0563413	DOCK6-AS1	19	11320574	-0.108	0.041	0.009
IS	rs737338	19	11347657	TRUE	C	0.0563413	TMEM205	19	11455323	0.072	0.017	2.95e-05
IS	rs737338	19	11347657	TRUE	C	0.0563413	CCDC159	19	11460490	0.109	0.032	0.001
IS	rs737338	19	11347657	TRUE	C	0.0563413	PLPPR2	19	11471218	0.058	0.018	0.001
IS	rs737338	19	11347657	TRUE	C	0.0563413	ELOF1	19	11666006	0.060	0.017	0.001
IS	rs737338	19	11347657	TRUE	C	0.0563413	ZNF844	19	12183947	0.051	0.021	0.017
IS	rs76213248	19	11269893	TRUE	C	0.385862	ILF3	19	10784015	0.020	0.008	0.015
IS	rs76213248	19	11269893	TRUE	C	0.385862	MRPL4	19	10366649	0.023	0.008	0.005
IS	rs76213248	19	11269893	TRUE	C	0.385862	CDKN2D	19	10678436	0.024	0.008	0.004
IS	rs9471972	6	42915021	G	A	0.481725	PEX6	6	42939283	-0.886	0.006	2.00e-248
IS	rs9471972	6	42915021	G	A	0.481725	RPL7L1	6	42852509	0.124	0.015	5.00e-17
IS	rs9471972	6	42915021	G	A	0.481725	CNPY3	6	42901981	-0.099	0.008	2.15e-35
IS	rs9471972	6	42915021	G	A	0.481725	ENSG00000231113	6	42909905	-0.178	0.019	1.17e-20
IS	rs9471972	6	42915021	G	A	0.481725	MEA1	6	42980769	0.042	0.008	1.08e-07
IS	rs9471972	6	42915021	G	A	0.481725	RRP36	6	42993359	-0.030	0.008	1.45e-04
IS	rs9471972	6	42915021	G	A	0.481725	MRPL2	6	43024655	-0.097	0.008	4.23e-34
IS	rs9471972	6	42915021	G	A	0.481725	DNPH1	6	43195294	0.069	0.009	7.92e-15
IS	rs9471972	6	42915021	G	A	0.481725	ZNF318	6	43318347	-0.034	0.008	2.07e-05

IS	rs9471972	6	42915021	G	A	0.481725	POLR1C	6	43487381	0.018	0.008	0.024
IS	rs9471972	6	42915021	G	A	0.481725	MAD2L1BP	6	43602983	-0.026	0.008	0.001

Chr denotes chromosome; BP, base pair position; EAF, effective allele frequency; SE, standard error. The data in this table was from eQTLGen consortium (https://eqtlgen.org/) and GTEx databases (https://www.gtexportal.org/).

Table S12. Genetic instruments for circulating gene expression levels in two-sample MR analysis.

Exposure (Genes)	SNP	Chr	Effect Allele	Alternate Allele	EAF	Beta	SE	*pval*	r^2	F statistic	Outcome
ADGRG3	rs12596635	16	C	G	0.41	0.269	0.009	1E-200	0.035	1151.781	CAD
ADGRG3	rs8049441	16	A	C	0.063	-0.174	0.02	9.07E-18	0.004	114.23	CAD
ADGRG5	rs35480010	16	T	C	0.039	0.351	0.021	3.81E-60	0.009	298.486	CAD
ADGRG5	rs6499882	16	G	A	0.321	-0.209	0.008	1.22E-135	0.019	614.396	CAD
AMIGO1	rs534135	1	T	C	0.476	-0.235	0.008	1.9E-196	0.027	894.02	CAD
AMIGO1	rs534135	1	T	C	0.476	-0.235	0.008	1.9E-196	0.027	894.02	CAD
ARHGAP9	rs486699	12	A	C	0.046	-0.62	0.02	1E-200	0.034	1108.976	CAD
ARHGAP9	rs703832	12	G	C	0.336	-0.08	0.008	2.35E-21	0.003	90.359	CAD
ARID1A	rs116725411	1	A	G	0.103	0.104	0.014	2.77E-14	0.002	63.968	CAD
ARID1A	rs60659886	1	T	A	0.091	-0.628	0.014	1E-200	0.065	2206.844	CAD
ARID1A	rs116725411	1	A	G	0.103	0.104	0.014	2.77E-14	0.002	63.968	CAD
ARID1A	rs60659886	1	T	A	0.091	-0.628	0.014	1E-200	0.065	2206.844	CAD
ARL2BP	rs10431929	16	G	T	0.501	-0.057	0.009	3.73E-11	0.002	52.076	CAD
ARL2BP	rs11642750	16	G	A	0.5	-0.084	0.008	2.55E-26	0.004	113.086	CAD
ARL2BP	rs118069881	16	C	T	0.028	-0.237	0.027	5.61E-18	0.003	95.885	CAD
ARL6IP4	rs11608811	12	A	G	0.301	-0.202	0.009	9.18E-120	0.017	551.817	CAD
ASB16-AS1	rs376816	17	A	C	0.209	-0.344	0.011	1E-200	0.039	1290.138	CAD
ASB16-AS1	rs5015	17	A	G	0.034	0.152	0.026	5.41E-09	0.002	48.07	CAD
ASB16-AS1	rs78403836	17	G	C	0.048	-0.183	0.024	4.54E-14	0.003	97.097	CAD
ATP23	rs10783853	12	A	G	0.296	-0.427	0.012	1E-200	0.076	2613.228	CAD
ATP23	rs11172462	12	T	C	0.384	0.209	0.012	1.32E-67	0.021	670.44	CAD
ATP6V0A2	rs11609591	12	A	T	0.379	0.109	0.008	2.05E-40	0.006	177.77	CAD
ATP6V0A2	rs145761741	12	A	G	0.019	-0.191	0.031	1.17E-09	0.001	42.339	CAD
ATP6V1B2	rs2088340	8	T	C	0.237	-0.055	0.01	6.62E-09	0.001	34.968	CAD

ATP6V1B2	rs7816924	8	T	C	0.152	-0.064	0.011	1.03E-08	0.001	33.574	CAD
AVIL	rs2014886	12	T	C	0.335	0.409	0.013	1E-200	0.075	2552.387	CAD
BBS2	rs11640851	16	C	A	0.359	-0.155	0.008	7.34E-78	0.011	356.336	CAD
BBS2	rs116909591	16	C	A	0.028	0.262	0.025	1.24E-25	0.004	117.632	CAD
BBS2	rs3764264	16	T	C	0.089	0.082	0.014	4.15E-09	0.001	34.788	CAD
BBS2	rs72814499	16	C	G	0.203	0.259	0.01	1.02E-153	0.022	704.918	CAD
BBS2	rs76284582	16	T	C	0.081	-0.472	0.015	1E-200	0.033	1091.089	CAD
BBS2	rs76424073	16	T	G	0.016	-0.574	0.034	9.54E-64	0.01	333.085	CAD
BBS2	rs11640851	16	C	A	0.359	-0.155	0.008	7.34E-78	0.011	356.336	CAD
BBS2	rs116909591	16	C	A	0.028	0.262	0.025	1.24E-25	0.004	117.632	CAD
BBS2	rs3764264	16	T	C	0.089	0.082	0.014	4.15E-09	0.001	34.788	CAD
BBS2	rs72814499	16	C	G	0.203	0.259	0.01	1.02E-153	0.022	704.918	CAD
BBS2	rs76284582	16	T	C	0.081	-0.472	0.015	1E-200	0.033	1091.089	CAD
BBS2	rs76424073	16	T	G	0.016	-0.574	0.034	9.54E-64	0.01	333.085	CAD
BFSP1	rs4814623	20	A	G	0.278	0.218	0.009	6.65E-136	0.019	615.6	CAD
BFSP1	rs911349	20	G	T	0.252	0.065	0.009	2.56E-12	0.002	49.784	CAD
BRCA1	rs34766339	17	T	C	0.352	0.266	0.009	8.91E-184	0.032	1056.904	CAD
CCDC92	rs144196238	12	T	C	0.166	-0.117	0.011	4.76E-28	0.004	121.022	CAD
CCDC92	rs3768	12	T	C	0.179	0.279	0.01	6.02E-163	0.023	739.946	CAD
CD320	rs147793891	19	A	G	0.206	-0.076	0.011	1.4E-12	0.002	60.024	CAD
CD320	rs2434434	19	A	G	0.256	-0.105	0.009	2.8E-30	0.004	133.587	CAD
CD320	rs2927714	19	T	C	0.174	-0.151	0.01	6.01E-47	0.007	208.456	CAD
CD320	rs7408511	19	T	C	0.348	0.082	0.008	2.91E-22	0.003	96.189	CAD
CD320	rs9426	19	T	C	0.036	-0.693	0.022	1E-200	0.033	1093.363	CAD
CD320	rs147793891	19	A	G	0.206	-0.076	0.011	1.4E-12	0.002	60.024	CAD
CD320	rs2434434	19	A	G	0.256	-0.105	0.009	2.8E-30	0.004	133.587	CAD

CD320	rs2927714	19	T	C	0.174	-0.151	0.01	6.01E-47	0.007	208.456	CAD
CD320	rs7408511	19	T	C	0.348	0.082	0.008	2.91E-22	0.003	96.189	CAD
CD320	rs9426	19	T	C	0.036	-0.693	0.022	1E-200	0.033	1093.363	CAD
CD52	rs11577318	1	G	A	0.179	-0.245	0.01	1E-124	0.018	568.04	CAD
CD52	rs149176316	1	A	G	0.019	-0.18	0.032	0.000000027	0.001	37.654	CAD
CD52	rs78395193	1	G	A	0.051	-0.184	0.019	1.4E-22	0.003	104.92	CAD
CD52	rs807253	1	G	A	0.231	-0.067	0.009	9.82E-13	0.002	51.221	CAD
CD52	rs11577318	1	G	A	0.179	-0.245	0.01	1E-124	0.018	568.04	CAD
CD52	rs149176316	1	A	G	0.019	-0.18	0.032	0.000000027	0.001	37.654	CAD
CD52	rs78395193	1	G	A	0.051	-0.184	0.019	1.4E-22	0.003	104.92	CAD
CD52	rs807253	1	G	A	0.231	-0.067	0.009	9.82E-13	0.002	51.221	CAD
CEP85	rs112752634	1	A	G	0.173	0.365	0.011	1E-200	0.038	1257.047	CAD
CEP85	rs11584185	1	C	T	0.311	0.116	0.01	1.62E-33	0.006	184.038	CAD
CEP85	rs112752634	1	A	G	0.173	0.365	0.011	1E-200	0.038	1257.047	CAD
CEP85	rs11584185	1	C	T	0.311	0.116	0.01	1.62E-33	0.006	184.038	CAD
CERS4	rs2016145	19	C	T	0.374	0.37	0.011	1E-200	0.064	2167.984	CAD
CERS4	rs2967629	19	T	C	0.3	0.082	0.01	1.63E-17	0.003	89.775	CAD
CERS4	rs4804251	19	T	A	0.5	0.074	0.011	4.62E-11	0.003	87.753	CAD
CERS4	rs72995486	19	C	T	0.034	0.356	0.026	1.18E-41	0.008	264.489	CAD
CERS4	rs8105180	19	T	C	0.047	0.147	0.022	4.58E-11	0.002	61.121	CAD
CETP	rs1532624	16	A	C	0.437	-0.13	0.008	1.1E-58	0.008	265.896	CAD
CETP	rs1651666	16	T	C	0.312	0.107	0.009	5.77E-36	0.005	157.819	CAD
CLASRP	rs1560725	19	C	T	0.467	-0.124	0.015	4.27E-17	0.008	243.6	CAD
CLCC1	rs11102668	1	T	C	0.041	0.187	0.02	2.14E-20	0.003	87.472	CAD
CLCC1	rs11102668	1	T	C	0.041	0.187	0.02	2.14E-20	0.003	87.472	CAD
CNKSR1	rs12122817	1	A	G	0.096	-0.104	0.013	1.48E-14	0.002	59.332	CAD

CNKSR1	rs2232648	1	T	C	0.312	0.132	0.009	8.7E-54	0.008	239.978	CAD
COQ9	rs223869	16	A	C	0.067	-0.251	0.016	1.52E-56	0.008	251.957	CAD
CPNE2	rs11076181	16	A	G	0.459	0.061	0.008	2.67E-14	0.002	57.968	CAD
CPNE2	rs13330423	16	T	C	0.442	-0.08	0.008	1.21E-23	0.003	100.458	CAD
CPNE2	rs16966632	16	A	G	0.042	0.525	0.02	1.86E-155	0.022	713.154	CAD
CPNE2	rs75443589	16	A	C	0.098	-0.131	0.014	7.93E-20	0.003	97.216	CAD
CSGALNACT1	rs11204066	8	C	T	0.526	0.145	0.008	4.04E-74	0.01	335.343	CAD
CSGALNACT1	rs4256608	8	G	A	0.442	0.265	0.008	1E-200	0.035	1135.905	CAD
CSGALNACT1	rs4922080	8	G	T	0.377	0.433	0.008	1E-200	0.088	3056.375	CAD
CSGALNACT1	rs6586878	8	T	C	0.287	0.062	0.009	1.67E-12	0.002	50.362	CAD
CSGALNACT1	rs78786063	8	C	T	0.161	0.064	0.011	4.03E-09	0.001	35.212	CAD
CYB561D1	rs17575205	1	C	G	0.24	-0.144	0.01	4.57E-45	0.008	240.064	CAD
CYB561D1	rs17575205	1	C	G	0.24	-0.144	0.01	4.57E-45	0.008	240.064	CAD
DCTN2	rs11172247	12	G	C	0.383	-0.047	0.008	1.17E-08	0.001	33.355	CAD
DDIT3	rs697221	12	A	G	0.144	0.191	0.012	4.9E-59	0.009	287.597	CAD
DSTN	rs11087234	20	A	G	0.131	-0.073	0.012	7.89E-10	0.001	37.928	CAD
DSTN	rs1887689	20	G	A	0.339	0.048	0.008	1.08E-08	0.001	32.811	CAD
DSTN	rs3790325	20	C	T	0.419	0.264	0.008	1E-200	0.034	1113.717	CAD
DSTN	rs6080717	20	A	G	0.281	0.189	0.009	2.21E-103	0.014	466.116	CAD
DSTN	rs6080767	20	T	C	0.204	-0.174	0.01	3.3E-70	0.01	313.738	CAD
EEF1AKMT3	rs111828051	12	T	C	0.018	-0.375	0.047	1.05E-15	0.005	159.313	CAD
EEF1AKMT3	rs11613303	12	T	C	0.015	-0.547	0.1	4.14E-08	0.009	279.807	CAD
EEF1AKMT3	rs117258237	12	T	C	0.038	0.776	0.024	1E-200	0.044	1463.424	CAD
EEF1AKMT3	rs117470861	12	A	G	0.037	-0.4	0.025	2.67E-58	0.011	359.952	CAD
EEF1AKMT3	rs183590952	12	A	G	0.012	0.966	0.096	9.7E-24	0.022	723.044	CAD
EEF1AKMT3	rs3923885	12	G	T	0.494	0.293	0.009	1E-200	0.043	1419.413	CAD

EEF1AKMT3	rs75209574	12	A	G	0.018	-0.525	0.043	2.07E-34	0.01	310.372	CAD
EEF1AKMT3	rs75470977	12	A	G	0.019	0.59	0.036	1.46E-61	0.013	421.434	CAD
EEF1AKMT3	rs75699640	12	T	C	0.018	0.834	0.039	6.51E-104	0.025	809.604	CAD
EEIG2	rs41278478	1	A	G	0.046	0.327	0.022	1.51E-51	0.009	300.318	CAD
EEIG2	rs41278478	1	A	G	0.046	0.327	0.022	1.51E-51	0.009	300.318	CAD
EIF2B1	rs28833471	12	C	G	0.182	0.327	0.011	1E-200	0.032	1042.671	CAD
EIF2B1	rs34705855	12	C	T	0.063	0.37	0.017	2.82E-107	0.016	521.905	CAD
EIF2B1	rs75634720	12	G	A	0.062	0.11	0.019	6.17E-09	0.001	44.99	CAD
ELAPOR1	rs113906101	1	A	T	0.084	-0.481	0.014	1E-200	0.036	1175.372	CAD
ELAPOR1	rs115263101	1	A	G	0.031	0.19	0.025	1.26E-14	0.002	68.547	CAD
ELAPOR1	rs17033886	1	G	A	0.241	-0.699	0.009	1E-200	0.179	6894.063	CAD
ELAPOR1	rs41278488	1	G	T	0.025	-0.156	0.026	3.67E-09	0.001	37.066	CAD
ELAPOR1	rs604500	1	C	T	0.249	-0.342	0.009	1E-200	0.044	1451.652	CAD
ELAPOR1	rs839855	1	A	G	0.046	0.187	0.02	1.86E-21	0.003	98.917	CAD
ELAPOR1	rs113906101	1	A	T	0.084	-0.481	0.014	1E-200	0.036	1175.372	CAD
ELAPOR1	rs115263101	1	A	G	0.031	0.19	0.025	1.26E-14	0.002	68.547	CAD
ELAPOR1	rs17033886	1	G	A	0.241	-0.699	0.009	1E-200	0.179	6894.063	CAD
ELAPOR1	rs41278488	1	G	T	0.025	-0.156	0.026	3.67E-09	0.001	37.066	CAD
ELAPOR1	rs604500	1	C	T	0.249	-0.342	0.009	1E-200	0.044	1451.652	CAD
ELAPOR1	rs839855	1	A	G	0.046	0.187	0.02	1.86E-21	0.003	98.917	CAD
ENSG00000205361	rs74024338	16	T	A	0.175	0.077	0.011	1.73E-12	0.002	53.649	CAD
ENSG00000224295	rs34225151	11	A	C	0.281	0.35	0.023	2.58E-53	0.05	1652.324	CAD
ENSG00000225113	rs4970768	1	A	G	0.437	0.178	0.019	9.76E-21	0.016	502.177	CAD
ENSG00000225113	rs4970768	1	A	G	0.437	0.178	0.019	9.76E-21	0.016	502.177	CAD
ENSG00000234553	rs1292144	5	A	G	0.495	-0.237	0.02	2.74E-31	0.028	916.985	CAD
ENSG00000234553	rs1292144	5	A	G	0.495	-0.237	0.02	2.74E-31	0.028	916.985	CAD

ENSG00000250091	rs111821796	12	T	C	0.018	0.239	0.04	3.05E-09	0.002	62.483	CAD
ENSG00000257499	rs923828	12	A	G	0.415	-0.213	0.019	7.44E-29	0.022	715.432	CAD
ENSG00000260145	rs117587884	16	A	G	0.024	-0.655	0.074	1.27E-18	0.02	641.644	CAD
ENSG00000260145	rs7206703	16	C	T	0.064	-0.747	0.041	2.71E-74	0.067	2262.642	CAD
ENSG00000260828	rs4784831	16	G	A	0.261	-0.316	0.021	7.61E-50	0.039	1272.255	CAD
ENSG00000261114	rs2399594	16	G	A	0.394	-0.125	0.019	1.23E-10	0.007	238.961	CAD
ENSG00000270028	rs79533202	12	C	T	0.028	0.604	0.057	2.99E-26	0.02	646.963	CAD
FGR	rs34806307	1	T	C	0.058	0.195	0.017	5.57E-29	0.004	132.098	CAD
FGR	rs34806307	1	T	C	0.058	0.195	0.017	5.57E-29	0.004	132.098	CAD
FHIP1B	rs117202587	11	T	A	0.047	0.97	0.018	1E-200	0.084	2902.872	CAD
FHIP1B	rs12365470	11	T	A	0.174	-0.073	0.011	5.08E-12	0.002	48.621	CAD
FHIP1B	rs2615951	11	G	A	0.069	0.366	0.016	6.99E-123	0.017	557.783	CAD
FHIP1B	rs61876722	11	C	T	0.015	-0.311	0.041	4.91E-14	0.003	91.902	CAD
GALNT2	rs12139970	1	T	G	0.281	0.072	0.009	2.31E-16	0.002	67.338	CAD
GALNT2	rs4846853	1	G	A	0.385	0.051	0.008	5.11E-10	0.001	38.778	CAD
GIHCG	rs3751325	12	T	A	0.33	-0.382	0.02	1.69E-84	0.065	2184.62	CAD
GNAO1	rs1809348	16	C	T	0.487	0.09	0.008	1.29E-29	0.004	127.745	CAD
GNAO1	rs55752378	16	A	G	0.02	0.223	0.029	1.25E-14	0.002	61.577	CAD
GNAO1	rs74523911	16	T	G	0.046	-0.37	0.019	8.82E-85	0.012	384.653	CAD
GPBP1	rs1862174	5	T	C	0.276	0.192	0.009	4.94E-105	0.015	474.3	CAD
GPBP1	rs1862174	5	T	C	0.276	0.192	0.009	4.94E-105	0.015	474.3	CAD
GPN2	rs7527776	1	T	C	0.345	0.078	0.009	6.67E-19	0.003	87.393	CAD
GPN2	rs7527776	1	T	C	0.345	0.078	0.009	6.67E-19	0.003	87.393	CAD
GRN	rs114641762	17	A	C	0.091	0.34	0.014	3.02E-122	0.019	615.338	CAD
GRN	rs12451763	17	T	C	0.028	-0.172	0.026	1.97E-11	0.002	51.057	CAD
GSTM1	rs10776700	1	A	G	0.021	-0.3	0.036	9.3E-17	0.004	115.746	CAD

GSTM1	rs113846254	1	T	C	0.013	-0.378	0.062	1.24E-09	0.004	114.281	CAD
GSTM1	rs114259186	1	G	T	0.019	0.536	0.036	1.94E-50	0.011	349.342	CAD
GSTM1	rs115181845	1	T	C	0.068	0.674	0.018	1E-200	0.057	1931.378	CAD
GSTM1	rs115929572	1	A	G	0.072	0.954	0.017	1E-200	0.122	4421.827	CAD
GSTM1	rs140610504	1	G	A	0.019	-0.234	0.039	1.34E-09	0.002	66.284	CAD
GSTM1	rs2269340	1	C	T	0.094	0.564	0.015	1E-200	0.054	1825.537	CAD
GSTM1	rs79707220	1	C	A	0.015	-0.385	0.061	2.38E-10	0.004	139.143	CAD
GSTM1	rs9651098	1	T	C	0.023	-0.505	0.081	4.57E-10	0.012	372.997	CAD
GSTM1	rs10776700	1	A	G	0.021	-0.3	0.036	9.3E-17	0.004	115.746	CAD
GSTM1	rs113846254	1	T	C	0.013	-0.378	0.062	1.24E-09	0.004	114.281	CAD
GSTM1	rs114259186	1	G	T	0.019	0.536	0.036	1.94E-50	0.011	349.342	CAD
GSTM1	rs115181845	1	T	C	0.068	0.674	0.018	1E-200	0.057	1931.378	CAD
GSTM1	rs115929572	1	A	G	0.072	0.954	0.017	1E-200	0.122	4421.827	CAD
GSTM1	rs140610504	1	G	A	0.019	-0.234	0.039	1.34E-09	0.002	66.284	CAD
GSTM1	rs2269340	1	C	T	0.094	0.564	0.015	1E-200	0.054	1825.537	CAD
GSTM1	rs79707220	1	C	A	0.015	-0.385	0.061	2.38E-10	0.004	139.143	CAD
GSTM1	rs9651098	1	T	C	0.023	-0.505	0.081	4.57E-10	0.012	372.997	CAD
GTF2H3	rs11573002	12	T	C	0.019	0.547	0.033	8.92E-62	0.011	360.704	CAD
HBG2	rs12365420	11	G	T	0.338	-0.104	0.016	2.23E-11	0.005	154.415	CAD
HBG2	rs3759074	11	A	G	0.302	0.538	0.015	1E-200	0.122	4408.674	CAD
HERPUD1	rs12720918	16	C	T	0.28	0.052	0.009	4.05E-09	0.001	34.731	CAD
HERPUD1	rs28728226	16	C	A	0.19	0.122	0.01	1.78E-33	0.005	145.917	CAD
HERPUD1	rs8044753	16	G	A	0.478	0.216	0.008	1.36E-165	0.023	754.927	CAD
HMGN2	rs2925523	1	C	T	0.057	0.262	0.021	1.93E-35	0.007	235.915	CAD
IFI35	rs138923296	17	G	A	0.033	-0.177	0.023	2.85E-14	0.002	62.887	CAD
IFI35	rs77106873	17	T	C	0.013	-0.703	0.039	3.48E-71	0.013	404.651	CAD

IGSF23	rs846858	19	A	G	0.332	-0.11	0.01	3.44E-28	0.005	170.713	CAD
INTS10	rs1826418	8	G	A	0.514	0.251	0.008	1E-200	0.032	1031.092	CAD
INTS10	rs1994786	8	T	C	0.184	0.151	0.01	1.16E-48	0.007	217.175	CAD
IRS1	rs115786869	2	G	A	0.018	0.38	0.034	1.42E-28	0.005	159.246	CAD
IRS1	rs13018683	2	T	A	0.275	0.243	0.009	1.43E-152	0.023	761.706	CAD
IRS1	rs13400941	2	G	A	0.484	0.068	0.008	1.99E-17	0.002	72.422	CAD
IRS1	rs115786869	2	G	A	0.018	0.38	0.034	1.42E-28	0.005	159.246	CAD
IRS1	rs13018683	2	T	A	0.275	0.243	0.009	1.43E-152	0.023	761.706	CAD
IRS1	rs13400941	2	G	A	0.484	0.068	0.008	1.99E-17	0.002	72.422	CAD
KMT5A	rs28780730	12	C	A	0.039	0.426	0.067	2.64E-10	0.014	436.17	CAD
KMT5A	rs6488882	12	T	C	0.02	0.435	0.066	3.28E-11	0.007	235.777	CAD
KMT5A	rs7139321	12	T	G	0.323	0.467	0.015	1E-200	0.095	3340.423	CAD
LINC01948	rs158214	5	C	T	0.367	0.152	0.01	2.48E-54	0.011	345.208	CAD
LINC01948	rs158214	5	C	T	0.367	0.152	0.01	2.48E-54	0.011	345.208	CAD
LOC101928728	rs17162313	1	G	A	0.078	-0.198	0.018	1.69E-29	0.006	179.133	CAD
LOC101928728	rs17162313	1	G	A	0.078	-0.198	0.018	1.69E-29	0.006	179.133	CAD
LPL	rs10106796	8	C	T	0.196	0.15	0.01	2.65E-51	0.007	227.016	CAD
LPL	rs17408078	8	T	A	0.178	-0.064	0.01	7.8E-10	0.001	37.953	CAD
LPL	rs34770253	8	C	T	0.19	-0.132	0.01	5.36E-39	0.005	171.313	CAD
LPL	rs3779787	8	T	G	0.152	0.351	0.011	1E-200	0.032	1034.537	CAD
LRP1	rs55909821	12	T	C	0.063	0.111	0.018	4.62E-10	0.001	46.645	CAD
LZTS1	rs2735950	8	G	A	0.183	0.271	0.01	6.12E-156	0.022	710.413	CAD
LZTS1	rs56222973	8	A	C	0.027	-0.168	0.025	2.04E-11	0.001	47.555	CAD
MAN1C1	rs2280999	1	C	T	0.327	0.112	0.009	5.02E-33	0.006	175.809	CAD
MAN1C1	rs4075441	1	G	A	0.238	0.079	0.01	2.04E-14	0.002	71.754	CAD
MAN1C1	rs2280999	1	C	T	0.327	0.112	0.009	5.02E-33	0.006	175.809	CAD

MAN1C1	rs4075441	1	G	A	0.238	0.079	0.01	2.04E-14	0.002	71.754	CAD
MAP3K6	rs111620626	1	T	G	0.015	0.251	0.038	5.45E-11	0.002	60.39	CAD
MAP3K6	rs114445029	1	A	G	0.04	-0.132	0.023	8.23E-09	0.001	41.764	CAD
MAP3K6	rs11548322	1	C	T	0.019	0.94	0.041	5.84E-118	0.033	1097.409	CAD
MAP3K6	rs145209848	1	A	G	0.03	-0.155	0.025	6.6E-10	0.001	44.305	CAD
MAP3K6	rs191328307	1	A	G	0.02	-1.04	0.084	1.41E-35	0.043	1416.746	CAD
MAP3K6	rs41291086	1	T	C	0.043	-0.417	0.02	5.08E-94	0.014	460.025	CAD
MAP3K6	rs56004188	1	G	A	0.034	-0.187	0.024	1.73E-14	0.002	72.647	CAD
MAP3K6	rs111620626	1	T	G	0.015	0.251	0.038	5.45E-11	0.002	60.39	CAD
MAP3K6	rs114445029	1	A	G	0.04	-0.132	0.023	8.23E-09	0.001	41.764	CAD
MAP3K6	rs11548322	1	C	T	0.019	0.94	0.041	5.84E-118	0.033	1097.409	CAD
MAP3K6	rs145209848	1	A	G	0.03	-0.155	0.025	6.6E-10	0.001	44.305	CAD
MAP3K6	rs191328307	1	A	G	0.02	-1.04	0.084	1.41E-35	0.043	1416.746	CAD
MAP3K6	rs41291086	1	T	C	0.043	-0.417	0.02	5.08E-94	0.014	460.025	CAD
MAP3K6	rs56004188	1	G	A	0.034	-0.187	0.024	1.73E-14	0.002	72.647	CAD
MARCHF2	rs1133893	19	A	G	0.306	-0.306	0.009	1E-200	0.04	1310.728	CAD
MARCHF2	rs141903508	19	A	G	0.025	0.197	0.028	1.25E-12	0.002	60.806	CAD
MARCHF2	rs60318332	19	G	A	0.092	-0.095	0.014	2.18E-11	0.002	48.304	CAD
MARCHF2	rs1133893	19	A	G	0.306	-0.306	0.009	1E-200	0.04	1310.728	CAD
MARCHF2	rs141903508	19	A	G	0.025	0.197	0.028	1.25E-12	0.002	60.806	CAD
MARCHF2	rs60318332	19	G	A	0.092	-0.095	0.014	2.18E-11	0.002	48.304	CAD
MARS1	rs3782125	12	G	A	0.364	0.154	0.012	3.34E-36	0.011	350.649	CAD
MBD6	rs1148557	12	T	C	0.144	0.11	0.012	1.05E-20	0.003	95.765	CAD
MBD6	rs117076209	12	T	C	0.013	-0.241	0.041	0.000000006	0.002	47.724	CAD
MGME1	rs113805511	20	A	G	0.036	0.309	0.028	3.8E-29	0.007	209.997	CAD
MGME1	rs117151396	20	G	A	0.027	0.489	0.032	1.01E-51	0.013	403.543	CAD

MGME1	rs141855599	20	A	G	0.012	-1.175	0.059	4.59E-87	0.034	1102.393	CAD
MGME1	rs180863608	20	T	C	0.039	-0.808	0.147	3.61E-08	0.049	1621.387	CAD
MGME1	rs56017653	20	C	A	0.046	-0.942	0.022	1E-200	0.077	2659.277	CAD
MPP2	rs231485	17	A	G	0.365	0.186	0.008	1.6E-112	0.016	514.427	CAD
MPP2	rs376816	17	A	C	0.209	-0.072	0.01	2.36E-13	0.002	54.465	CAD
MRPS18A	rs75886301	6	C	T	0.018	0.195	0.032	1.09E-09	0.001	42.389	CAD
MRPS18A	rs75886301	6	C	T	0.018	0.195	0.032	1.09E-09	0.001	42.389	CAD
MT1E	rs1610216	16	G	A	0.198	-0.101	0.011	9.44E-20	0.003	102.204	CAD
MT1E	rs2270836	16	T	C	0.405	-0.263	0.009	7.67E-198	0.033	1094.505	CAD
MT1F	rs2011186	16	T	C	0.434	0.251	0.008	1E-200	0.031	1013.433	CAD
MT1F	rs58105980	16	G	A	0.176	0.334	0.01	1E-200	0.032	1058.763	CAD
MT1F	rs626942	16	C	A	0.296	-0.097	0.009	1.06E-28	0.004	124.026	CAD
MT1F	rs8048695	16	A	G	0.163	-0.079	0.011	2.27E-13	0.002	54.313	CAD
MT1F	rs9930761	16	C	T	0.058	-0.137	0.017	1.32E-15	0.002	64.779	CAD
MT1X	rs10636	16	C	G	0.256	0.116	0.01	2.66E-34	0.005	164.804	CAD
MT1X	rs12447019	16	G	T	0.434	0.289	0.008	1E-200	0.041	1354.127	CAD
MT1X	rs8062526	16	C	T	0.186	-0.065	0.01	2.37E-10	0.001	40.135	CAD
MT3	rs1580833	16	A	C	0.299	-0.15	0.009	9.25E-65	0.009	300.839	CAD
MT3	rs4784714	16	A	G	0.481	0.05	0.008	4.92E-10	0.001	39.742	CAD
MYO1F	rs113576178	19	T	G	0.112	0.201	0.013	3.54E-57	0.008	257.622	CAD
MYO1F	rs11880897	19	C	T	0.357	0.053	0.008	3.01E-10	0.001	40.243	CAD
NBR2	rs12051788	17	C	T	0.31	-0.265	0.009	1E-200	0.03	984.487	CAD
NBR2	rs12603963	17	C	T	0.372	-0.054	0.009	5.84E-10	0.001	43.785	CAD
NDUFA4L2	rs3923885	12	G	T	0.494	0.046	0.008	0.000000036	0.001	34.241	CAD
NDUFA7	rs12459489	19	A	C	0.099	-0.089	0.016	9.55E-09	0.001	45.244	CAD
NDUFA7	rs138174609	19	T	C	0.049	0.251	0.024	5.58E-25	0.006	185.946	CAD

NDUFA7	rs148238005	19	T	C	0.021	-0.219	0.032	4.75E-12	0.002	61.339	CAD
NDUFA7	rs561	19	A	G	0.167	-0.372	0.013	1.39E-175	0.039	1272.696	CAD
NECTIN2	rs10406338	19	C	T	0.332	0.279	0.009	1E-200	0.035	1132.506	CAD
NECTIN2	rs17561351	19	G	A	0.059	1.532	0.015	1E-200	0.259	11096.422	CAD
NECTIN2	rs2695801	19	T	C	0.336	0.052	0.009	1.09E-09	0.001	38.198	CAD
NECTIN2	rs62118499	19	T	C	0.082	0.108	0.016	4.57E-12	0.002	55.373	CAD
NEMP1	rs150470545	12	T	C	0.023	1.039	0.032	1E-200	0.048	1602.395	CAD
NEMP1	rs17546153	12	G	A	0.035	0.243	0.025	7.3E-23	0.004	127.55	CAD
NLRC5	rs1167742	16	G	C	0.315	0.424	0.012	1E-200	0.078	2669.772	CAD
NLRC5	rs75003587	16	G	A	0.102	-0.135	0.02	3.91E-11	0.003	105.578	CAD
NLRC5	rs8062446	16	T	C	0.375	0.211	0.012	3.84E-68	0.021	675.91	CAD
NLRC5	rs1167742	16	G	C	0.315	0.424	0.012	1E-200	0.078	2669.772	CAD
NLRC5	rs75003587	16	G	A	0.102	-0.135	0.02	3.91E-11	0.003	105.578	CAD
NLRC5	rs8062446	16	T	C	0.375	0.211	0.012	3.84E-68	0.021	675.91	CAD
NUDT21	rs4784669	16	A	G	0.389	0.103	0.008	8.09E-37	0.005	160.861	CAD
NUP93-DT	rs4238789	16	T	C	0.181	-0.268	0.029	6.2E-20	0.021	690.987	CAD
OR7D2	rs10422989	19	C	T	0.031	0.264	0.033	2.37E-15	0.004	133.703	CAD
OR7D2	rs11673664	19	G	A	0.448	-0.065	0.01	1.47E-11	0.002	67.054	CAD
OR7D2	rs36034490	19	A	C	0.036	0.373	0.025	1.44E-49	0.01	310.985	CAD
OR7D2	rs4804118	19	G	T	0.071	-0.102	0.018	7.47E-09	0.001	43.123	CAD
OR7D2	rs59149515	19	C	A	0.066	1.145	0.017	1E-200	0.161	6091.44	CAD
PCCB	rs3772390	3	G	A	0.033	-0.231	0.022	5.82E-25	0.003	107.787	CAD
PCCB	rs6439610	3	G	A	0.412	0.243	0.008	1E-200	0.029	934.703	CAD
PCCB	rs76578737	3	A	G	0.09	0.301	0.014	6E-97	0.015	474.67	CAD
PCCB	rs3772390	3	G	A	0.033	-0.231	0.022	5.82E-25	0.003	107.787	CAD
PCCB	rs6439610	3	G	A	0.412	0.243	0.008	1E-200	0.029	934.703	CAD

PCCB	rs76578737	3	A	G	0.09	0.301	0.014	6E-97	0.015	474.67	CAD
PEX6	rs1106841	6	C	A	0.39	0.064	0.008	2.93E-15	0.002	62.308	CAD
PEX6	rs139863435	6	T	C	0.016	0.363	0.04	1.95E-19	0.004	132.395	CAD
PEX6	rs149747900	6	C	A	0.013	-0.473	0.064	1.21E-13	0.006	185.55	CAD
PEX6	rs3729617	6	A	G	0.373	0.349	0.008	1E-200	0.057	1913.423	CAD
PEX6	rs62402409	6	C	T	0.283	-0.144	0.009	2.56E-60	0.008	269.369	CAD
PEX6	rs72859402	6	G	A	0.019	-0.25	0.031	1.33E-15	0.002	73.371	CAD
PEX6	rs73428430	6	G	A	0.325	0.271	0.009	1E-200	0.032	1058.098	CAD
PEX6	rs1106841	6	C	A	0.39	0.064	0.008	2.93E-15	0.002	62.308	CAD
PEX6	rs139863435	6	T	C	0.016	0.363	0.04	1.95E-19	0.004	132.395	CAD
PEX6	rs149747900	6	C	A	0.013	-0.473	0.064	1.21E-13	0.006	185.55	CAD
PEX6	rs3729617	6	A	G	0.373	0.349	0.008	1E-200	0.057	1913.423	CAD
PEX6	rs62402409	6	C	T	0.283	-0.144	0.009	2.56E-60	0.008	269.369	CAD
PEX6	rs72859402	6	G	A	0.019	-0.25	0.031	1.33E-15	0.002	73.371	CAD
PEX6	rs73428430	6	G	A	0.325	0.271	0.009	1E-200	0.032	1058.098	CAD
PGBD5	rs3761949	1	T	C	0.181	0.151	0.01	2.18E-47	0.007	214.987	CAD
PIGV	rs34935290	1	T	C	0.222	0.052	0.01	4.13E-08	0.001	30.167	CAD
PIGV	rs60659886	1	T	A	0.091	-0.472	0.014	1E-200	0.037	1208.381	CAD
PIGV	rs34935290	1	T	C	0.222	0.052	0.01	4.13E-08	0.001	30.167	CAD
PIGV	rs60659886	1	T	A	0.091	-0.472	0.014	1E-200	0.037	1208.381	CAD
PIP4K2C	rs7313599	12	G	A	0.412	0.122	0.008	7.13E-52	0.007	229.633	CAD
PLLP	rs117139586	16	A	C	0.014	-0.405	0.042	2.99E-22	0.005	149.01	CAD
PLLP	rs2305693	16	C	G	0.097	0.119	0.014	6.25E-18	0.002	78.743	CAD
PLLP	rs72778735	16	A	G	0.064	-0.159	0.019	1.38E-16	0.003	95.917	CAD
PPP2R3A	rs12695644	3	T	C	0.254	0.122	0.009	9.03E-41	0.006	180.657	CAD
PPP2R3A	rs12695644	3	T	C	0.254	0.122	0.009	9.03E-41	0.006	180.657	CAD

PSME3IP1	rs12599485	16	C	T	0.054	-0.32	0.018	1E-73	0.01	335.225	CAD
PSME3IP1	rs4784774	16	T	A	0.369	-0.078	0.009	1.95E-18	0.003	89.826	CAD
PSRC1	rs413380	1	T	C	0.031	0.367	0.023	4.18E-58	0.008	261.883	CAD
PSRC1	rs4970834	1	T	C	0.188	0.418	0.01	1E-200	0.053	1789.751	CAD
PSRC1	rs413380	1	T	C	0.031	0.367	0.023	4.18E-58	0.008	261.883	CAD
PSRC1	rs4970834	1	T	C	0.188	0.418	0.01	1E-200	0.053	1789.751	CAD
RAB11B	rs1136888	19	T	C	0.341	0.063	0.008	3.84E-14	0.002	57.458	CAD
RILPL2	rs138808037	12	T	C	0.031	0.16	0.027	4.5E-09	0.002	48.786	CAD
RILPL2	rs34506612	12	A	G	0.056	0.235	0.02	1.15E-32	0.006	185.396	CAD
RILPL2	rs4759404	12	T	C	0.17	-0.106	0.012	9.72E-20	0.003	100.542	CAD
RILPL2	rs56116847	12	A	G	0.368	-0.289	0.009	1E-200	0.039	1284.251	CAD
RPS28	rs2972572	19	A	G	0.45	0.257	0.015	1.35E-69	0.033	1072.381	CAD
RRBP1	rs2618604	20	A	C	0.059	0.104	0.017	1.12E-09	0.001	38.236	CAD
RRBP1	rs6034875	20	G	A	0.412	0.255	0.008	1E-200	0.031	1029.623	CAD
RRBP1	rs6075218	20	G	T	0.335	0.07	0.008	1.14E-16	0.002	69.92	CAD
RSPRY1	rs11076181	16	A	G	0.459	0.147	0.008	3.41E-77	0.011	345.843	CAD
SH3BGRL3	rs7551751	1	G	A	0.426	0.125	0.008	1.85E-54	0.008	242.468	CAD
SLC12A3	rs56228609	16	T	C	0.306	-0.109	0.013	6.11E-17	0.005	159.125	CAD
SLC12A3	rs8044753	16	G	A	0.478	0.076	0.012	1.75E-10	0.003	90.474	CAD
SLC18A1	rs112971957	8	T	C	0.05	0.164	0.018	2.54E-19	0.003	82.035	CAD
SLC18A1	rs73210887	8	T	G	0.438	0.299	0.008	1E-200	0.044	1457.721	CAD
SLC18A1	rs76385722	8	A	G	0.021	0.164	0.03	0.000000029	0.001	35.128	CAD
SNRNP35	rs11516178	12	T	C	0.153	-0.112	0.012	4.28E-20	0.003	102.876	CAD
SNRNP35	rs56399357	12	G	C	0.211	0.068	0.011	2.85E-10	0.002	48.556	CAD
STAT6	rs117852993	12	T	C	0.032	0.207	0.023	4.6E-19	0.003	84.522	CAD
STAT6	rs703818	12	T	A	0.224	0.372	0.011	1E-200	0.048	1607.694	CAD

STAT6	rs703849	12	T	C	0.358	0.285	0.008	1E-200	0.037	1230.875	CAD
STAT6	rs71461327	12	T	C	0.048	0.229	0.02	7.27E-32	0.005	153.576	CAD
STMN1	rs1257156	1	G	T	0.19	-0.204	0.011	7.51E-77	0.013	411.689	CAD
SYTL1	rs28385651	1	C	T	0.041	-0.546	0.029	4.58E-77	0.024	766.892	CAD
SYTL1	rs28385651	1	C	T	0.041	-0.546	0.029	4.58E-77	0.024	766.892	CAD
TCTN2	rs10847570	12	A	G	0.329	0.056	0.008	2.99E-11	0.001	44.346	CAD
TCTN2	rs11834492	12	C	T	0.36	0.176	0.008	1.87E-101	0.014	459.043	CAD
TCTN2	rs80334313	12	G	A	0.027	-0.249	0.025	1.3E-23	0.003	104.38	CAD
TMED2	rs4930709	12	T	C	0.246	0.173	0.009	4E-77	0.011	355.241	CAD
TMEM101	rs35071596	17	T	C	0.037	-0.688	0.022	1E-200	0.033	1092.077	CAD
TMEM101	rs6503486	17	T	C	0.046	0.208	0.019	1.03E-27	0.004	120.757	CAD
TMUB2	rs11079983	17	T	C	0.28	-0.116	0.009	3.14E-39	0.005	171.688	CAD
TRIM34	rs10838482	11	T	A	0.038	0.134	0.022	5.39E-10	0.001	41.966	CAD
TRIM34	rs12787013	11	G	A	0.118	0.071	0.012	1.18E-08	0.001	33.399	CAD
TRIM5	rs11604371	11	T	C	0.397	0.095	0.008	1.22E-31	0.004	136.961	CAD
TRIM5	rs12795021	11	C	T	0.042	0.142	0.022	1.09E-10	0.002	51.059	CAD
TRIM5	rs68062776	11	T	C	0.109	-0.419	0.013	1E-200	0.034	1114.521	CAD
TRIM6	rs10769115	11	T	C	0.239	0.227	0.01	9.78E-111	0.019	606.752	CAD
TRIM6	rs11038369	11	A	G	0.047	0.258	0.021	2.16E-35	0.006	189.444	CAD
TRIM6	rs117350827	11	T	C	0.029	0.162	0.027	1.72E-09	0.002	47.819	CAD
TRIM6	rs75742187	11	T	C	0.019	-0.361	0.039	1.12E-20	0.005	152.633	CAD
TRNP1	rs71514284	1	C	T	0.053	0.194	0.019	2.27E-23	0.004	120.992	CAD
TRNP1	rs7530420	1	T	C	0.37	-0.069	0.008	9.01E-17	0.002	69.428	CAD
TSPAN31	rs10877051	12	G	T	0.036	-0.17	0.025	5.68E-12	0.002	63.776	CAD
TSPAN31	rs111566142	12	G	A	0.06	-0.557	0.018	1E-200	0.035	1152.836	CAD
TSPAN31	rs113514774	12	A	G	0.017	0.274	0.037	1.39E-13	0.003	81.292	CAD

UBQLNL	rs11038546	11	A	C	0.428	-0.18	0.008	1.87E-113	0.016	512.397	CAD
UBQLNL	rs3740999	11	C	A	0.473	-0.259	0.008	1E-200	0.034	1099.342	CAD
UBQLNL	rs7934223	11	C	T	0.349	0.051	0.009	1.28E-08	0.001	37.31	CAD
UBQLNL	rs904364	11	C	T	0.293	0.055	0.009	2.2E-10	0.001	40.439	CAD
UBQLNL	rs979752	11	A	G	0.308	-0.086	0.009	1.08E-23	0.003	100.683	CAD
UBTF	rs113844752	17	A	C	0.305	0.118	0.009	7.16E-38	0.006	186.918	CAD
USP24	rs17111584	1	C	T	0.045	-0.356	0.019	3.54E-75	0.011	349.543	CAD
USP24	rs187758512	1	G	A	0.38	-0.051	0.009	2.76E-08	0.001	39.499	CAD
ZDHHC18	rs17370283	1	G	C	0.084	0.274	0.014	1.28E-82	0.012	372.105	CAD
ZDHHC18	rs4075636	1	T	C	0.096	-0.077	0.014	2.72E-08	0.001	32.543	CAD
ZDHHC18	rs60659886	1	T	A	0.091	0.498	0.014	1E-200	0.041	1354.292	CAD
ZDHHC18	rs17370283	1	G	C	0.084	0.274	0.014	1.28E-82	0.012	372.105	CAD
ZDHHC18	rs4075636	1	T	C	0.096	-0.077	0.014	2.72E-08	0.001	32.543	CAD
ZDHHC18	rs60659886	1	T	A	0.091	0.498	0.014	1E-200	0.041	1354.292	CAD
ZNF558	rs12979932	19	T	C	0.385	0.094	0.015	7.08E-10	0.004	132.259	CAD
ZNF558	rs2910355	19	G	C	0.291	-0.375	0.019	7.06E-88	0.058	1949.131	CAD
ZNF664	rs10744165	12	C	T	0.126	0.067	0.012	2.99E-08	0.001	31.041	CAD
ZNF664	rs7958691	12	T	G	0.326	-0.177	0.008	9.83E-97	0.014	440.164	CAD
ZNF683	rs11247938	1	G	T	0.43	-0.056	0.008	3.65E-12	0.002	48.805	CAD
ZNF683	rs55675162	1	A	G	0.212	-0.316	0.01	1E-200	0.033	1092.4	CAD
ZNF683	rs11247938	1	G	T	0.43	-0.056	0.008	3.65E-12	0.002	48.805	CAD
ZNF683	rs55675162	1	A	G	0.212	-0.316	0.01	1E-200	0.033	1092.4	CAD
ACP5	rs12609856	19	C	A	0.52	-0.135	0.008	1.98E-63	0.009	291.809	IS
ACP5	rs12977116	19	G	T	0.269	0.054	0.009	5.96E-09	0.001	36.015	IS
ACP5	rs4804604	19	A	C	0.415	-0.407	0.008	1E-200	0.08	2773.383	IS
ACP5	rs73498702	19	C	T	0.215	0.382	0.01	1E-200	0.049	1638.509	IS

AGER	rs17840121	6	C	G	0.135	0.193	0.019	7.39E-24	0.009	279.552	IS
AGER	rs2507991	6	A	C	0.266	-0.076	0.014	3.33E-08	0.002	71.235	IS
AGER	rs693906	6	C	G	0.137	0.197	0.018	3.78E-29	0.009	293.382	IS
AP1M2	rs1821279	19	C	G	0.212	0.326	0.011	1.27E-200	0.036	1169.563	IS
AP1M2	rs58116906	19	T	C	0.017	-0.323	0.036	1.58E-19	0.003	110.82	IS
AP1M2	rs7260034	19	C	T	0.059	0.102	0.018	1.84E-08	0.001	36.72	IS
AP1M2	rs75864794	19	T	C	0.034	-0.291	0.023	5.38E-38	0.006	176.334	IS
BICRAL	rs6458308	6	C	T	0.47	0.174	0.009	3.48E-82	0.015	483.637	IS
BICRAL	rs73434663	6	A	G	0.213	0.078	0.012	1.44E-11	0.002	65.53	IS
C19orf38	rs140789286	19	T	C	0.028	-0.255	0.032	6.77E-16	0.003	110.512	IS
C4A	rs1269852	6	C	G	0.103	-0.588	0.023	5.85E-148	0.064	2152.099	IS
C4A	rs140054334	6	T	C	0.02	0.539	0.059	3.63E-20	0.011	360.703	IS
C4A	rs9264533	6	T	C	0.197	0.218	0.017	4.57E-37	0.015	484.66	IS
C4B	rs3094222	6	G	A	0.112	0.899	0.03	1E-200	0.161	6074.586	IS
C4B	rs3134954	6	C	T	0.136	-0.68	0.028	4.81E-129	0.108	3851.286	IS
CARM1	rs117786851	19	A	G	0.173	0.083	0.011	2.9E-15	0.002	62.983	IS
CARM1	rs78301016	19	A	G	0.121	-0.275	0.012	7.25E-114	0.016	519.809	IS
CCDC159	rs651774	19	A	G	0.187	0.617	0.024	5.66E-152	0.116	4154.759	IS
CCND3	rs72853854	6	A	G	0.209	-0.181	0.01	3.4E-77	0.011	345.892	IS
CDKN2D	rs8108051	19	C	T	0.458	0.161	0.009	3.71E-80	0.013	415.643	IS
CNPY3	rs4714634	6	A	G	0.439	0.131	0.008	1.7E-60	0.009	272.087	IS
CUL7	rs141136987	6	A	G	0.061	-0.1	0.018	4.89E-08	0.001	36.397	IS
CUL7	rs2273918	6	C	T	0.076	-0.42	0.015	3.79E-177	0.025	805.255	IS
DDAH2	rs144229847	6	A	T	0.029	-0.2	0.036	2.67E-08	0.002	72.251	IS
DDAH2	rs9267576	6	T	G	0.128	0.423	0.018	6.86E-127	0.04	1315.292	IS
DLK2	rs1214749	6	A	G	0.293	-0.251	0.009	2.72E-186	0.026	847.524	IS

DLK2	rs2651203	6	C	T	0.204	0.058	0.01	4.66E-09	0.001	35.192	IS
DNPH1	rs111763904	6	A	C	0.028	-0.385	0.027	9.84E-46	0.008	262.297	IS
DNPH1	rs116189428	6	G	T	0.044	0.172	0.025	6.62E-12	0.002	79.103	IS
DNPH1	rs1761768	6	A	G	0.388	-0.091	0.009	1.77E-23	0.004	125.62	IS
DNPH1	rs78797716	6	A	C	0.064	-0.545	0.018	1E-200	0.036	1173.789	IS
DOCK6	rs651774	19	A	G	0.187	0.11	0.011	1.44E-23	0.004	116.135	IS
DOCK6-AS1	rs12980879	19	A	G	0.063	-0.777	0.038	8.6E-94	0.071	2430.432	IS
ELOF1	rs181454479	19	A	G	0.03	0.241	0.024	2.1E-24	0.003	106.992	IS
ELOF1	rs7359902	19	A	G	0.149	0.307	0.011	1.01E-170	0.024	775.697	IS
ENSG00000226104	rs9305017	19	A	G	0.426	-0.112	0.02	1.59E-08	0.006	195.969	IS
ENSG00000229391	rs149234868	6	G	A	0.354	0.43	0.01	1E-200	0.085	2929.303	IS
ENSG00000229391	rs2070121	6	A	G	0.084	0.09	0.015	1.38E-09	0.001	39.914	IS
ENSG00000229391	rs9469082	6	G	A	0.109	0.156	0.013	2.53E-32	0.005	149.755	IS
ENSG00000231113	rs56106855	6	C	G	0.284	0.26	0.021	1.46E-35	0.027	894.346	IS
ENSG00000234515	rs4148882	6	G	A	0.381	0.112	0.013	1.62E-18	0.006	187.11	IS
ENSG00000267174	rs11668477	19	G	A	0.186	-0.169	0.025	1.78E-11	0.009	277.335	IS
ENSG00000267174	rs2116876	19	A	G	0.241	-0.626	0.021	1E-200	0.144	5310.938	IS
ENSG00000267174	rs7417	19	G	A	0.101	-0.654	0.031	1.91E-96	0.078	2664.304	IS
ENSG00000267174	rs11668477	19	G	A	0.186	-0.169	0.025	1.78E-11	0.009	277.335	IS
ENSG00000267174	rs2116876	19	A	G	0.241	-0.626	0.021	1E-200	0.144	5310.938	IS
ENSG00000267174	rs7417	19	G	A	0.101	-0.654	0.031	1.91E-96	0.078	2664.304	IS
EPOR	rs651774	19	A	G	0.187	0.333	0.011	1E-200	0.034	1104.964	IS
GNMT	rs12195802	6	C	T	0.246	-0.098	0.014	9.41E-13	0.004	113.198	IS
GNMT	rs3763236	6	C	T	0.49	-0.365	0.011	1E-200	0.066	2256.456	IS
GNMT	rs6940142	6	G	T	0.14	-0.176	0.017	1.26E-24	0.007	238.748	IS
HLA-DMA	rs1050391	6	A	G	0.076	0.36	0.024	1.52E-52	0.018	583.011	IS

HLA-DOB	rs146253626	6	T	C	0.018	0.445	0.054	3.16E-16	0.007	222.962	IS
HLA-DOB	rs2857114	6	G	A	0.29	0.429	0.013	1E-200	0.076	2594.489	IS
HLA-DOB	rs4148879	6	A	G	0.13	0.29	0.019	4.19E-55	0.019	612.413	IS
HLA-DPB1	rs11965463	6	A	G	0.043	-0.559	0.03	1.9E-75	0.026	846.276	IS
HLA-DPB1	rs13205975	6	A	G	0.053	-0.349	0.028	8.9E-36	0.012	390.54	IS
HLA-DPB1	rs2076775	6	G	C	0.391	-0.093	0.013	1.58E-13	0.004	130.182	IS
HLA-DPB1	rs9277935	6	T	G	0.211	-0.423	0.015	3.08E-175	0.06	2008.861	IS
HLA-DQA2	rs2760994	6	C	T	0.413	-0.31	0.021	1.14E-47	0.047	1552.149	IS
HLA-DQA2	rs75635252	6	G	A	0.277	1.059	0.035	1E-200	0.449	25771.746	IS
HLA-DQB1	rs114437758	6	G	A	0.037	-0.146	0.025	8.73E-09	0.002	47.634	IS
HLA-DQB1	rs191785247	6	A	G	0.105	0.646	0.017	1E-200	0.078	2688.455	IS
HLA-DQB1	rs41267086	6	A	G	0.062	0.213	0.019	6.06E-28	0.005	166.925	IS
HLA-DQB1-AS1	rs9270911	6	C	T	0.465	0.747	0.02	1E-200	0.278	12192.414	IS
HLA-DQB1-AS1	rs9271377	6	G	T	0.387	-0.628	0.021	2.29E-191	0.187	7290.121	IS
HLA-DQB2	rs7751376	6	C	G	0.44	-0.447	0.015	1E-200	0.099	3465.417	IS
HLA-DQB2	rs79022332	6	A	G	0.161	-0.219	0.024	1.69E-19	0.013	415.273	IS
HLA-DRB1	rs28383456	6	T	C	0.305	-0.911	0.019	1E-200	0.352	17190.106	IS
HLA-DRB1	rs3130100	6	T	C	0.46	0.097	0.016	2.44E-09	0.005	148.89	IS
HLA-DRB1	rs71534539	6	G	A	0.471	-0.468	0.037	2.4E-37	0.109	3876.011	IS
HLA-DRB1	rs75252378	6	T	C	0.432	-1.035	0.034	1E-200	0.526	35167.339	IS
HLA-DRB5	rs116520501	6	T	C	0.039	0.159	0.026	5.88E-10	0.002	60.487	IS
HLA-DRB5	rs116729028	6	C	T	0.029	-0.365	0.03	1.83E-33	0.008	240.358	IS
HLA-DRB5	rs2267647	6	A	G	0.34	0.139	0.009	2.38E-49	0.009	277.732	IS
HLA-DRB5	rs2523504	6	T	C	0.28	0.391	0.01	1E-200	0.062	2081.825	IS
HLA-DRB5	rs73728204	6	C	A	0.043	-0.348	0.024	1.77E-46	0.01	320.122	IS
ICAM1	rs281437	19	T	C	0.243	0.182	0.01	2.88E-74	0.012	389.838	IS

ICAM3	rs61198164	19	A	C	0.067	0.113	0.016	1.96E-12	0.002	50.594	IS
ICAM3	rs7409490	19	G	A	0.165	0.443	0.011	1E-200	0.054	1814.681	IS
ILF3	rs12610411	19	A	G	0.312	0.051	0.009	3.92E-09	0.001	34.789	IS
ILF3-DT	rs138995968	19	A	G	0.02	0.373	0.04	1.1E-20	0.005	170.308	IS
ILF3-DT	rs150696463	19	T	C	0.028	1.047	0.034	1E-200	0.061	2044.405	IS
ILF3-DT	rs8113381	19	C	T	0.209	-0.147	0.013	1.11E-27	0.007	227.295	IS
KANK2	rs147762096	19	T	C	0.059	-0.269	0.017	1.52E-56	0.008	256.381	IS
KEAP1	rs75407602	19	T	C	0.034	-0.202	0.03	1.5E-11	0.003	85.404	IS
KEAP1	rs79342923	19	T	G	0.088	0.104	0.014	1.88E-13	0.002	54.712	IS
KLHDC3	rs4987174	6	T	C	0.054	-0.185	0.019	7.28E-23	0.003	110.681	IS
KRI1	rs11881803	19	G	T	0.066	-0.135	0.018	2.03E-14	0.002	71.922	IS
KRI1	rs1465701	19	T	C	0.253	-0.249	0.011	4.27E-117	0.023	758.265	IS
LDLR	rs8110515	19	G	T	0.125	0.092	0.012	2.94E-14	0.002	58.389	IS
LY6G5C	rs1144709	6	T	C	0.236	-0.522	0.014	1E-200	0.098	3444.914	IS
LY6G5C	rs2075798	6	A	C	0.077	-0.278	0.023	1.68E-34	0.011	352.664	IS
LY6G5C	rs2534658	6	G	A	0.117	0.108	0.019	1.24E-08	0.002	75.751	IS
MEA1	rs111320577	6	A	G	0.028	-0.141	0.024	5.42E-09	0.001	34.387	IS
MEA1	rs112446807	6	T	C	0.041	0.119	0.02	3.45E-09	0.001	35.039	IS
MEA1	rs116750300	6	A	C	0.04	0.257	0.022	2.08E-30	0.005	161.745	IS
MRPL2	rs61695510	6	C	T	0.076	0.264	0.015	4.92E-70	0.01	314.122	IS
MRPL2	rs9462853	6	C	T	0.259	0.205	0.009	4.09E-114	0.016	517.435	IS
MRPL4	rs1059840	19	A	T	0.115	0.28	0.013	5.72E-96	0.016	514.886	IS
MRPS18A	rs75886301	6	C	T	0.018	0.195	0.032	1.09E-09	0.001	42.389	IS
MSH5	rs1802127	6	T	C	0.02	-0.631	0.091	4.57E-12	0.015	498.144	IS
MSH5	rs2299851	6	A	G	0.104	-0.28	0.03	3.32E-20	0.015	469.273	IS
PDE4A	rs10416139	19	T	C	0.242	-0.066	0.009	1.85E-12	0.002	51.145	IS

PDE4A	rs77376335	19	C	T	0.087	-0.233	0.014	5.72E-62	0.009	276.949	IS
PEX6	rs1106841	6	C	A	0.39	0.064	0.008	2.93E-15	0.002	62.308	IS
PEX6	rs139863435	6	T	C	0.016	0.363	0.04	1.95E-19	0.004	132.395	IS
PEX6	rs3729617	6	A	G	0.373	0.349	0.008	1E-200	0.057	1913.423	IS
PEX6	rs62402409	6	C	T	0.283	-0.144	0.009	2.56E-60	0.008	269.369	IS
PEX6	rs72859402	6	G	A	0.019	-0.25	0.031	1.33E-15	0.002	73.371	IS
PEX6	rs73428430	6	G	A	0.325	0.271	0.009	1E-200	0.032	1058.098	IS
PEX6	rs1106841	6	C	A	0.39	0.064	0.008	2.93E-15	0.002	62.308	IS
PEX6	rs139863435	6	T	C	0.016	0.363	0.04	1.95E-19	0.004	132.395	IS
PEX6	rs3729617	6	A	G	0.373	0.349	0.008	1E-200	0.057	1913.423	IS
PEX6	rs62402409	6	C	T	0.283	-0.144	0.009	2.56E-60	0.008	269.369	IS
PEX6	rs72859402	6	G	A	0.019	-0.25	0.031	1.33E-15	0.002	73.371	IS
PEX6	rs73428430	6	G	A	0.325	0.271	0.009	1E-200	0.032	1058.098	IS
PLPPR2	rs112145494	19	G	A	0.02	0.194	0.033	5.02E-09	0.001	45.778	IS
PLPPR2	rs651774	19	A	G	0.187	0.422	0.011	1E-200	0.054	1816.904	IS
POLR1C	rs61018535	6	A	C	0.03	-0.237	0.027	5.18E-18	0.003	103.263	IS
PPP2R5D	rs10080728	6	C	T	0.134	-0.171	0.012	6.53E-48	0.007	216.409	IS
PPP2R5D	rs2234185	6	C	T	0.46	-0.06	0.008	3.9E-14	0.002	57.215	IS
PRKCSH	rs160841	19	G	A	0.071	0.256	0.015	9.34E-62	0.009	276.086	IS
PRKCSH	rs6511728	19	G	A	0.164	-0.062	0.011	1.47E-08	0.001	32.964	IS
PRKCSH	rs160841	19	G	A	0.071	0.256	0.015	9.34E-62	0.009	276.086	IS
PRKCSH	rs6511728	19	G	A	0.164	-0.062	0.011	1.47E-08	0.001	32.964	IS
PSMB9	rs114311872	6	T	C	0.047	0.317	0.028	8.3E-29	0.009	289.699	IS
PSMB9	rs116802814	6	C	T	0.016	0.339	0.05	1.33E-11	0.004	112.58	IS
PSMB9	rs2071466	6	T	C	0.275	0.403	0.013	1E-200	0.065	2192.466	IS
PSMB9	rs41267086	6	A	G	0.062	0.183	0.025	4.09E-13	0.004	122.849	IS

PTCRA	rs9471941	6	G	A	0.227	-0.085	0.01	2.82E-19	0.003	81.099	IS
RGL3	rs10413578	19	G	A	0.08	0.788	0.031	7.58E-143	0.092	3198.654	IS
RGL3	rs12459282	19	G	A	0.083	-0.233	0.027	1.87E-18	0.008	265.196	IS
RNF5	rs62402717	6	A	G	0.027	0.656	0.063	2.15E-25	0.022	728.49	IS
RNF5	rs9273109	6	G	A	0.296	-0.205	0.027	8.47E-14	0.017	563.537	IS
RPL7L1	rs1150797	6	A	G	0.226	-0.352	0.017	1.36E-92	0.043	1436.418	IS
RRP36	rs61169091	6	A	G	0.141	-0.166	0.011	4.93E-48	0.007	212.816	IS
RRP36	rs73733781	6	T	G	0.016	-0.223	0.038	6.63E-09	0.002	49.404	IS
S1PR5	rs113540846	19	A	G	0.158	0.078	0.011	8.74E-13	0.002	51.669	IS
S1PR5	rs28580742	19	C	T	0.367	0.177	0.009	1E-86	0.014	465.499	IS
SLC44A2	rs117719127	19	T	C	0.016	-0.826	0.035	1.09E-124	0.021	693.136	IS
SLC44A2	rs142641751	19	C	G	0.034	0.322	0.022	4.69E-48	0.007	215.764	IS
SLC44A2	rs62131824	19	A	G	0.054	0.202	0.019	2.06E-25	0.004	131.979	IS
SLC44A2	rs73005679	19	A	G	0.016	0.302	0.038	1.97E-15	0.003	92.139	IS
SLC44A2	rs76490264	19	T	C	0.02	0.228	0.036	1.59E-10	0.002	65.017	IS
SLC44A2	rs77587461	19	T	C	0.033	-0.695	0.022	1E-200	0.031	1018.833	IS
SMARCA4	rs73013167	19	C	G	0.299	-0.166	0.009	2.04E-82	0.012	371.151	IS
SPC24	rs10402457	19	T	C	0.232	-0.103	0.009	6.96E-28	0.004	120.617	IS
SWSAP1	rs651774	19	A	G	0.187	0.212	0.012	5.71E-66	0.014	437.445	IS
TAP2	rs241424	6	A	G	0.454	-0.089	0.012	2.31E-13	0.004	125.454	IS
TAP2	rs4148876	6	A	G	0.067	-1.197	0.034	1E-200	0.179	6921.489	IS
TAP2	rs55869430	6	G	A	0.062	-0.395	0.025	3.6E-56	0.018	582.622	IS
TIMM29	rs36059375	19	C	T	0.411	-0.136	0.008	5.28E-63	0.009	284.754	IS
TMED1	rs140789286	19	T	C	0.028	-0.78	0.025	1E-200	0.033	1066.304	IS
TMED1	rs5030370	19	A	G	0.284	-0.07	0.011	4.92E-10	0.002	63.368	IS
TMEM205	rs2163839	19	G	A	0.054	0.111	0.018	4.09E-10	0.001	39.481	IS

TMEM205	rs651774	19	A	G	0.187	0.369	0.011	1E-200	0.041	1367.268	IS
TNXB	rs2269426	6	A	G	0.402	0.435	0.018	1.6E-126	0.091	3168.781	IS
TNXB	rs9469079	6	T	C	0.139	-0.24	0.027	3.75E-19	0.014	442.758	IS
TSPAN16	rs651774	19	A	G	0.187	-0.232	0.012	6.37E-81	0.016	529.261	IS
VPS52	rs1800837	6	T	C	0.188	-0.151	0.015	2.21E-22	0.007	221.18	IS
VWA7	rs139194483	6	T	C	0.027	0.462	0.068	1.55E-11	0.011	362.224	IS
VWA7	rs139938004	6	A	G	0.048	-0.444	0.037	2.51E-32	0.018	586.262	IS
VWA7	rs28745895	6	A	C	0.041	-0.517	0.041	5.81E-37	0.021	675.002	IS
VWA7	rs3131382	6	T	C	0.059	0.275	0.048	6.66E-09	0.008	268.116	IS
ZNF20	rs8104980	19	G	C	0.13	0.148	0.013	1.46E-31	0.005	158.46	IS
ZNF318	rs140665652	6	G	A	0.021	-0.313	0.037	1.27E-17	0.004	129.165	IS
ZNF318	rs61729324	6	A	G	0.074	0.533	0.015	1E-200	0.039	1276.053	IS
ZNF318	rs75317537	6	T	C	0.027	0.311	0.028	3.5E-29	0.005	159.396	IS
ZNF441	rs286221	19	G	A	0.421	0.074	0.008	4.91E-20	0.003	85.231	IS
ZNF491	rs286232	19	G	A	0.173	-0.108	0.011	7.74E-25	0.003	106.615	IS
ZNF844	rs60722046	19	A	G	0.077	0.186	0.018	3.47E-24	0.005	157.833	IS

Chr denotes chromosome; EAF, effective allele frequency; SE, standard error; $R^2 = 2 \times MAF \times (1-MAF) \times beta^2$; $F = R^2 \times (N-2)/(1-R^2)$.

Table S13. Causal associations of circulating genes with CAD and IS.

Outcome	Tissue	Genes	Methods	NSNPs	OR (95%CI)	*pval*	*FDR*
CAD	Blood	ADGRG3	IVW	2	1.04 (1.00, 1.08)	0.037	0.214
CAD	Blood	ADGRG5	IVW	2	0.96 (0.92, 1.01)	0.148	0.442
CAD	Blood	AMIGO1	Wald ratio	1	0.94 (0.90, 0.98)	0.003	0.045
CAD	Blood	ARHGAP9	IVW	2	0.98 (0.89, 1.08)	0.659	0.922
CAD	Blood	ARID1A	IVW	2	0.98 (0.95, 1.01)	0.133	0.442
CAD	Blood	ARL2BP	MR Egger	3	1.16 (0.97, 1.38)	0.350	0.990
CAD	Blood	ARL2BP	Weighted median	3	1.09 (1.00, 1.19)	0.064	0.314
CAD	Blood	ARL2BP	IVW	3	1.06 (0.98, 1.15)	0.120	0.442
CAD	Blood	ARL6IP4	Wald ratio	1	1.00 (0.95, 1.06)	0.874	0.949
CAD	Blood	ASB16-AS1	MR Egger	3	1.05 (0.94, 1.18)	0.537	0.990
CAD	Blood	ASB16-AS1	Weighted median	3	1.03 (0.99, 1.06)	0.153	0.489
CAD	Blood	ASB16-AS1	IVW	3	1.02 (0.99, 1.06)	0.155	0.442
CAD	Blood	ATP23	IVW	2	1.00 (0.98, 1.03)	0.868	0.949
CAD	Blood	ATP6V0A2	IVW	2	1.08 (0.97, 1.21)	0.141	0.442
CAD	Blood	ATP6V1B2	IVW	2	0.97 (0.84, 1.13)	0.705	0.922
CAD	Blood	AVIL	Wald ratio	1	1.02 (0.99, 1.04)	0.201	0.458
CAD	Blood	BBS2	MR Egger	6	0.99 (0.94, 1.04)	0.697	0.990
CAD	Blood	BBS2	Weighted median	6	1.00 (0.97, 1.03)	0.764	0.923
CAD	Blood	BBS2	IVW	6	1.00 (0.98, 1.03)	0.696	0.922
CAD	Blood	BFSP1	IVW	2	1.00 (0.79, 1.27)	1.000	1.000
CAD	Blood	BRCA1	Wald ratio	1	1.07 (1.03, 1.12)	0.001	0.014
CAD	Blood	CCDC92	IVW	2	0.93 (0.84, 1.02)	0.115	0.442
CAD	Blood	CD320	MR Egger	5	1.06 (1.02, 1.11)	0.077	0.990
CAD	Blood	CD320	Weighted median	5	1.05 (1.02, 1.09)	0.003	0.043
CAD	Blood	CD320	IVW	5	1.05 (1.01, 1.08)	0.006	0.067
CAD	Blood	CD52	MR Egger	4	1.03 (0.93, 1.13)	0.644	0.990

CAD	Blood	CD52	Weighted median	4	0.97 (0.92, 1.02)	0.239	0.528
CAD	Blood	CD52	IVW	4	0.96 (0.92, 1.01)	0.117	0.442
CAD	Blood	CEP85	IVW	2	1.02 (0.99, 1.06)	0.157	0.442
CAD	Blood	CERS4	MR Egger	4	0.97 (0.89, 1.06)	0.604	0.990
CAD	Blood	CERS4	Weighted median	4	0.98 (0.96, 1.01)	0.253	0.540
CAD	Blood	CERS4	IVW	4	0.99 (0.95, 1.03)	0.604	0.882
CAD	Blood	CETP	IVW	2	1.13 (0.94, 1.35)	0.203	0.458
CAD	Blood	CLASRP	Wald ratio	1	1 (0.92, 1.08)	0.955	0.986
CAD	Blood	CLCC1	Wald ratio	1	1 (0.89, 1.13)	0.979	0.996
CAD	Blood	CNKSR1	IVW	2	0.94 (0.88, 1.01)	0.112	0.442
CAD	Blood	COQ9	Wald ratio	1	0.96 (0.89, 1.03)	0.240	0.504
CAD	Blood	CPNE2	MR Egger	4	0.98 (0.93, 1.03)	0.484	0.990
CAD	Blood	CPNE2	Weighted median	4	0.99 (0.95, 1.03)	0.507	0.754
CAD	Blood	CPNE2	IVW	4	0.99 (0.95, 1.03)	0.568	0.869
CAD	Blood	CSGALNACT1	MR Egger	5	0.96 (0.92, 1)	0.163	0.990
CAD	Blood	CSGALNACT1	Weighted median	5	0.98 (0.96, 1.01)	0.142	0.477
CAD	Blood	CSGALNACT1	IVW	5	0.98 (0.96, 1.01)	0.155	0.442
CAD	Blood	CYB561D1	Wald ratio	1	1.1 (1.02, 1.19)	0.015	0.129
CAD	Blood	DCTN2	Wald ratio	1	0.8 (0.65, 0.99)	0.039	0.214
CAD	Blood	DDIT3	Wald ratio	1	0.96 (0.89, 1.02)	0.199	0.458
CAD	Blood	DSTN	MR Egger	5	0.94 (0.88, 1)	0.125	0.990
CAD	Blood	DSTN	Weighted median	5	0.97 (0.94, 1.01)	0.109	0.434
CAD	Blood	DSTN	IVW	5	0.98 (0.95, 1.01)	0.209	0.458
CAD	Blood	EEF1AKMT3	MR Egger	9	1.04 (1, 1.08)	0.093	0.990
CAD	Blood	EEF1AKMT3	Weighted median	9	1.01 (0.99, 1.03)	0.475	0.742
CAD	Blood	EEF1AKMT3	IVW	9	1 (0.99, 1.02)	0.774	0.927
CAD	Blood	EEIG2	Wald ratio	1	1.01 (0.93, 1.09)	0.886	0.954
CAD	Blood	EIF2B1	MR Egger	3	1.03 (0.81, 1.31)	0.838	0.990

CAD	Blood	EIF2B1	Weighted median	3	1 (0.97, 1.04)	0.994	0.994
CAD	Blood	EIF2B1	IVW	3	1.01 (0.96, 1.07)	0.652	0.922
CAD	Blood	ELAPOR1	MR Egger	6	1.04 (1, 1.08)	0.092	0.990
CAD	Blood	ELAPOR1	Weighted median	6	1.01 (0.99, 1.03)	0.187	0.500
CAD	Blood	ELAPOR1	IVW	6	1 (0.98, 1.02)	0.816	0.949
CAD	Blood	ENSG00000205361	Wald ratio	1	0.99 (0.84, 1.18)	0.949	0.986
CAD	Blood	ENSG00000224295	Wald ratio	1	1.01 (0.98, 1.04)	0.673	0.922
CAD	Blood	ENSG00000225113	Wald ratio	1	1.01 (0.96, 1.07)	0.728	0.922
CAD	Blood	ENSG00000234553	Wald ratio	1	1.01 (0.97, 1.05)	0.604	0.882
CAD	Blood	ENSG00000250091	Wald ratio	1	0.87 (0.74, 1.01)	0.074	0.361
CAD	Blood	ENSG00000257499	Wald ratio	1	1.03 (0.98, 1.08)	0.194	0.458
CAD	Blood	ENSG00000260145	IVW	2	0.99 (0.96, 1.01)	0.242	0.504
CAD	Blood	ENSG00000260828	Wald ratio	1	0.98 (0.95, 1.02)	0.354	0.638
CAD	Blood	ENSG00000261114	Wald ratio	1	1.04 (0.96, 1.13)	0.334	0.633
CAD	Blood	ENSG00000270028	Wald ratio	1	0.99 (0.94, 1.04)	0.592	0.882
CAD	Blood	FGR	Wald ratio	1	0.94 (0.85, 1.03)	0.197	0.458
CAD	Blood	FHIP1B	MR Egger	4	1.01 (0.97, 1.04)	0.756	0.990
CAD	Blood	FHIP1B	Weighted median	4	1.01 (0.99, 1.03)	0.336	0.632
CAD	Blood	FHIP1B	IVW	4	1.01 (0.99, 1.03)	0.317	0.610
CAD	Blood	GALNT2	IVW	2	0.99 (0.82, 1.2)	0.936	0.986
CAD	Blood	GIHCG	Wald ratio	1	1.01 (0.98, 1.04)	0.454	0.768
CAD	Blood	GNAO1	MR Egger	3	1 (0.92, 1.09)	0.990	0.990
CAD	Blood	GNAO1	Weighted median	3	0.98 (0.93, 1.03)	0.368	0.673
CAD	Blood	GNAO1	IVW	3	0.97 (0.92, 1.02)	0.277	0.558
CAD	Blood	GPBP1	Wald ratio	1	1.04 (0.99, 1.11)	0.127	0.442
CAD	Blood	GPN2	Wald ratio	1	0.91 (0.79, 1.04)	0.149	0.442
CAD	Blood	GRN	IVW	2	0.93 (0.89, 0.98)	0.004	0.047
CAD	Blood	GSTM1	MR Egger	9	0.99 (0.92, 1.06)	0.804	0.990

CAD	Blood	GSTM1	Weighted median	9	1 (0.98, 1.02)	0.934	0.983
CAD	Blood	GSTM1	IVW	9	1 (0.98, 1.02)	0.771	0.927
CAD	Blood	GTF2H3	Wald ratio	1	0.97 (0.91, 1.04)	0.424	0.728
CAD	Blood	HBG2	IVW	2	1 (0.98, 1.02)	0.760	0.927
CAD	Blood	HERPUD1	MR Egger	3	0.83 (0.76, 0.9)	0.147	0.990
CAD	Blood	HERPUD1	Weighted median	3	0.98 (0.94, 1.03)	0.455	0.728
CAD	Blood	HERPUD1	IVW	3	1 (0.87, 1.15)	0.998	1.000
CAD	Blood	HMGN2	Wald ratio	1	1.08 (0.99, 1.19)	0.091	0.411
CAD	Blood	IFI35	IVW	2	0.98 (0.9, 1.08)	0.740	0.922
CAD	Blood	IGSF23	Wald ratio	1	0.99 (0.9, 1.09)	0.844	0.949
CAD	Blood	INTS10	IVW	2	0.96 (0.9, 1.02)	0.161	0.444
CAD	Blood	IRS1	MR Egger	3	0.95 (0.83, 1.09)	0.605	0.990
CAD	Blood	IRS1	Weighted median	3	1 (0.96, 1.04)	0.945	0.983
CAD	Blood	IRS1	IVW	3	1.01 (0.94, 1.08)	0.830	0.949
CAD	Blood	KMT5A	MR Egger	3	1.27 (0.38, 4.18)	0.764	0.990
CAD	Blood	KMT5A	Weighted median	3	1 (0.98, 1.02)	0.889	0.965
CAD	Blood	KMT5A	IVW	3	1 (0.98, 1.02)	0.980	0.996
CAD	Blood	LINC01948	Wald ratio	1	0.94 (0.88, 1.01)	0.086	0.406
CAD	Blood	LOC101928728	Wald ratio	1	0.93 (0.85, 1.01)	0.095	0.415
CAD	Blood	LPL	MR Egger	4	0.83 (0.72, 0.96)	0.123	0.990
CAD	Blood	LPL	Weighted median	4	0.94 (0.9, 0.98)	0.003	0.043
CAD	Blood	LPL	IVW	4	0.95 (0.85, 1.05)	0.306	0.599
CAD	Blood	LRP1	Wald ratio	1	0.83 (0.68, 1.01)	0.068	0.346
CAD	Blood	LZTS1	IVW	2	1.03 (0.98, 1.07)	0.221	0.475
CAD	Blood	MAN1C1	IVW	2	1.02 (0.9, 1.16)	0.740	0.922
CAD	Blood	MAP3K6	MR Egger	7	0.97 (0.91, 1.02)	0.303	0.990
CAD	Blood	MAP3K6	Weighted median	7	0.96 (0.93, 0.99)	0.018	0.162
CAD	Blood	MAP3K6	IVW	7	0.97 (0.93, 1)	0.036	0.214

CAD	Blood	MARCHF2	MR Egger	3	0.83 (0.75, 0.91)	0.163	0.990
CAD	Blood	MARCHF2	Weighted median	3	1.03 (0.99, 1.06)	0.107	0.434
CAD	Blood	MARCHF2	IVW	3	1.04 (0.93, 1.17)	0.499	0.815
CAD	Blood	MARS1	Wald ratio	1	1.08 (1.01, 1.16)	0.016	0.129
CAD	Blood	MBD6	IVW	2	0.91 (0.82, 1)	0.057	0.302
CAD	Blood	MGME1	MR Egger	5	1 (0.96, 1.05)	0.868	0.990
CAD	Blood	MGME1	Weighted median	5	1.02 (1, 1.04)	0.108	0.434
CAD	Blood	MGME1	IVW	5	1.02 (1, 1.04)	0.135	0.442
CAD	Blood	MPP2	IVW	2	0.97 (0.87, 1.08)	0.549	0.869
CAD	Blood	MRPS18A	Wald ratio	1	1.04 (0.85, 1.26)	0.710	0.922
CAD	Blood	MT1E	IVW	2	0.98 (0.95, 1.02)	0.287	0.570
CAD	Blood	MT1F	MR Egger	5	0.99 (0.93, 1.04)	0.687	0.990
CAD	Blood	MT1F	Weighted median	5	0.99 (0.96, 1.02)	0.567	0.772
CAD	Blood	MT1F	IVW	5	0.99 (0.97, 1.02)	0.567	0.869
CAD	Blood	MT1X	MR Egger	3	1.03 (0.89, 1.18)	0.769	0.990
CAD	Blood	MT1X	Weighted median	3	1.01 (0.97, 1.04)	0.706	0.886
CAD	Blood	MT1X	IVW	3	1.01 (0.95, 1.06)	0.834	0.949
CAD	Blood	MT3	IVW	2	1.01 (0.9, 1.13)	0.873	0.949
CAD	Blood	MYO1F	IVW	2	0.84 (0.78, 0.9)	1.36E-06	5.75E-05
CAD	Blood	NBR2	IVW	2	0.92 (0.88, 0.96)	0.001	0.009
CAD	Blood	NDUFA4L2	Wald ratio	1	0.87 (0.71, 1.08)	0.209	0.458
CAD	Blood	NDUFA7	MR Egger	4	1.01 (0.93, 1.09)	0.827	0.990
CAD	Blood	NDUFA7	Weighted median	4	1 (0.97, 1.04)	0.793	0.939
CAD	Blood	NDUFA7	IVW	4	1 (0.97, 1.03)	0.944	0.986
CAD	Blood	NECTIN2	MR Egger	4	1.02 (0.99, 1.04)	0.320	0.990
CAD	Blood	NECTIN2	Weighted median	4	1.02 (1.01, 1.03)	0.003	0.043
CAD	Blood	NECTIN2	IVW	4	1.02 (1, 1.04)	0.022	0.164
CAD	Blood	NEMP1	IVW	2	1.01 (0.98, 1.03)	0.720	0.922

CAD	Blood	NLRC5	MR Egger	3	0.97 (0.92, 1.02)	0.469	0.990
CAD	Blood	NLRC5	Weighted median	3	1 (0.98, 1.02)	0.971	0.986
CAD	Blood	NLRC5	IVW	3	1 (0.98, 1.03)	0.802	0.943
CAD	Blood	NUDT21	Wald ratio	1	0.9 (0.81, 0.99)	0.028	0.184
CAD	Blood	NUP93-DT	Wald ratio	1	1 (0.96, 1.05)	0.855	0.949
CAD	Blood	OR7D2	MR Egger	5	1.01 (0.99, 1.03)	0.432	0.990
CAD	Blood	OR7D2	Weighted median	5	1 (0.98, 1.02)	0.654	0.855
CAD	Blood	OR7D2	IVW	5	1 (0.98, 1.02)	0.906	0.967
CAD	Blood	PCCB	MR Egger	3	1.15 (0.77, 1.72)	0.619	0.990
CAD	Blood	PCCB	Weighted median	3	0.99 (0.95, 1.02)	0.496	0.754
CAD	Blood	PCCB	IVW	3	0.99 (0.96, 1.02)	0.461	0.771
CAD	Blood	PEX6	MR Egger	7	0.99 (0.89, 1.1)	0.852	0.990
CAD	Blood	PEX6	Weighted median	7	0.96 (0.93, 0.99)	0.023	0.186
CAD	Blood	PEX6	IVW	7	0.98 (0.94, 1.02)	0.357	0.638
CAD	Blood	PGBD5	Wald ratio	1	1.02 (0.93, 1.1)	0.712	0.922
CAD	Blood	PIGV	IVW	2	0.97 (0.94, 1.01)	0.141	0.442
CAD	Blood	PIP4K2C	Wald ratio	1	1.01 (0.93, 1.1)	0.752	0.927
CAD	Blood	PLLP	MR Egger	3	1.04 (0.79, 1.36)	0.842	0.990
CAD	Blood	PLLP	Weighted median	3	1.06 (0.97, 1.16)	0.211	0.500
CAD	Blood	PLLP	IVW	3	1.06 (0.97, 1.15)	0.190	0.458
CAD	Blood	PPP2R3A	Wald ratio	1	1.29 (1.18, 1.41)	1.78E-08	1.13E-06
CAD	Blood	PSME3IP1	IVW	2	0.95 (0.85, 1.06)	0.353	0.638
CAD	Blood	PSRC1	IVW	2	0.82 (0.8, 0.84)	7.57E-41	9.62E-39
CAD	Blood	RAB11B	Wald ratio	1	1.44 (1.23, 1.69)	7.34E-06	2.33E-04
CAD	Blood	RILPL2	MR Egger	4	0.99 (0.87, 1.12)	0.848	0.990
CAD	Blood	RILPL2	Weighted median	4	1 (0.97, 1.04)	0.808	0.941
CAD	Blood	RILPL2	IVW	4	1.02 (0.98, 1.06)	0.403	0.704
CAD	Blood	RPS28	Wald ratio	1	1.07 (1.03, 1.11)	4.26E-04	0.009

CAD	Blood	RRBP1	MR Egger	3	0.98 (0.85, 1.13)	0.839	0.990
CAD	Blood	RRBP1	Weighted median	3	0.98 (0.94, 1.02)	0.303	0.606
CAD	Blood	RRBP1	IVW	3	0.99 (0.94, 1.03)	0.554	0.869
CAD	Blood	RSPRY1	Wald ratio	1	1.01 (0.95, 1.08)	0.726	0.922
CAD	Blood	SH3BGRL3	Wald ratio	1	0.98 (0.91, 1.07)	0.703	0.922
CAD	Blood	SLC12A3	IVW	2	1.19 (0.79, 1.78)	0.405	0.704
CAD	Blood	SLC18A1	MR Egger	3	0.93 (0.79, 1.09)	0.517	0.990
CAD	Blood	SLC18A1	Weighted median	3	0.99 (0.96, 1.02)	0.407	0.704
CAD	Blood	SLC18A1	IVW	3	0.99 (0.96, 1.02)	0.533	0.856
CAD	Blood	SNRNP35	IVW	2	0.98 (0.78, 1.23)	0.870	0.949
CAD	Blood	STAT6	MR Egger	4	1.02 (0.89, 1.18)	0.777	0.990
CAD	Blood	STAT6	Weighted median	4	0.98 (0.95, 1.01)	0.181	0.500
CAD	Blood	STAT6	IVW	4	0.98 (0.96, 1)	0.113	0.442
CAD	Blood	STMN1	Wald ratio	1	1.09 (1.02, 1.15)	0.007	0.073
CAD	Blood	SYTL1	Wald ratio	1	0.96 (0.92, 1.01)	0.152	0.442
CAD	Blood	TCTN2	MR Egger	3	1.1 (0.99, 1.23)	0.326	0.990
CAD	Blood	TCTN2	Weighted median	3	1.05 (1, 1.11)	0.053	0.306
CAD	Blood	TCTN2	IVW	3	1.05 (1, 1.11)	0.038	0.214
CAD	Blood	TMED2	Wald ratio	1	1.04 (0.98, 1.11)	0.191	0.458
CAD	Blood	TMEM101	IVW	2	0.99 (0.93, 1.06)	0.790	0.938
CAD	Blood	TMUB2	Wald ratio	1	1.13 (1.03, 1.25)	0.009	0.083
CAD	Blood	TRIM34	IVW	2	1.19 (1.02, 1.39)	0.027	0.184
CAD	Blood	TRIM5	MR Egger	3	1 (0.95, 1.06)	0.986	0.990
CAD	Blood	TRIM5	Weighted median	3	0.99 (0.96, 1.03)	0.699	0.886
CAD	Blood	TRIM5	IVW	3	0.99 (0.96, 1.02)	0.595	0.882
CAD	Blood	TRIM6	MR Egger	4	0.91 (0.61, 1.34)	0.673	0.990
CAD	Blood	TRIM6	Weighted median	4	1.03 (0.99, 1.08)	0.166	0.500
CAD	Blood	TRIM6	IVW	4	1.01 (0.96, 1.07)	0.689	0.922

CAD	Blood	TRNP1	IVW	2	0.95 (0.87, 1.03)	0.199	0.458
CAD	Blood	TSPAN31	MR Egger	3	0.89 (0.82, 0.96)	0.210	0.990
CAD	Blood	TSPAN31	Weighted median	3	0.94 (0.91, 0.98)	0.001	0.043
CAD	Blood	TSPAN31	IVW	3	0.94 (0.91, 0.98)	0.008	0.073
CAD	Blood	UBQLNL	MR Egger	5	0.99 (0.91, 1.09)	0.903	0.990
CAD	Blood	UBQLNL	Weighted median	5	1.01 (0.97, 1.05)	0.543	0.755
CAD	Blood	UBQLNL	IVW	5	0.98 (0.94, 1.02)	0.346	0.638
CAD	Blood	UBTF	Wald ratio	1	0.94 (0.86, 1.03)	0.183	0.458
CAD	Blood	USP24	IVW	2	1.04 (0.98, 1.11)	0.197	0.458
CAD	Blood	ZDHHC18	MR Egger	3	0.96 (0.91, 1.02)	0.395	0.990
CAD	Blood	ZDHHC18	Weighted median	3	1.03 (1, 1.06)	0.038	0.269
CAD	Blood	ZDHHC18	IVW	3	1.04 (0.97, 1.11)	0.274	0.558
CAD	Blood	ZNF558	IVW	2	1.02 (0.97, 1.07)	0.500	0.815
CAD	Blood	ZNF664	IVW	2	1.19 (1.09, 1.3)	8.72E-05	0.002
CAD	Blood	ZNF683	IVW	2	0.99 (0.96, 1.03)	0.730	0.922
IS	Blood	ACP5	MR Egger	4	0.97 (0.91, 1.03)	0.449	0.990
IS	Blood	ACP5	Weighted median	4	1.02 (0.98, 1.06)	0.318	0.618
IS	Blood	ACP5	IVW	4	1.02 (0.98, 1.07)	0.259	1.000
IS	Blood	AGER	MR Egger	3	0.9 (0.68, 1.2)	0.604	0.990
IS	Blood	AGER	Weighted median	3	1 (0.9, 1.11)	0.952	0.983
IS	Blood	AGER	IVW	3	1 (0.9, 1.1)	0.926	1.000
IS	Blood	AP1M2	MR Egger	4	1.02 (0.82, 1.28)	0.871	0.990
IS	Blood	AP1M2	Weighted median	4	1.04 (0.98, 1.1)	0.200	0.500
IS	Blood	AP1M2	IVW	4	1.05 (1, 1.11)	0.074	0.705
IS	Blood	BICRAL	IVW	2	1.06 (0.93, 1.21)	0.363	1.000
IS	Blood	C19orf38	Wald ratio	1	0.87 (0.68, 1.11)	0.265	1.000
IS	Blood	C4A	MR Egger	3	0.98 (0.89, 1.09)	0.772	0.990
IS	Blood	C4A	Weighted median	3	1 (0.95, 1.05)	0.857	0.962

IS	Blood	C4A	IVW	3	0.99 (0.94, 1.03)	0.536	1.000
IS	Blood	C4B	IVW	2	1.01 (0.98, 1.04)	0.588	1.000
IS	Blood	CARM1	IVW	2	1.04 (0.75, 1.43)	0.824	1.000
IS	Blood	CCDC159	Wald ratio	1	1 (0.96, 1.04)	0.920	1.000
IS	Blood	CCND3	Wald ratio	1	1.05 (0.94, 1.18)	0.406	1.000
IS	Blood	CDKN2D	Wald ratio	1	0.87 (0.78, 0.95)	0.004	0.098
IS	Blood	CNPY3	Wald ratio	1	1.13 (1, 1.28)	0.046	0.541
IS	Blood	CUL7	IVW	2	0.99 (0.88, 1.11)	0.855	1.000
IS	Blood	DDAH2	IVW	2	0.95 (0.89, 1.01)	0.105	0.810
IS	Blood	DLK2	IVW	2	1.03 (0.81, 1.3)	0.834	1.000
IS	Blood	DNPH1	MR Egger	4	0.95 (0.85, 1.06)	0.429	0.990
IS	Blood	DNPH1	Weighted median	4	1 (0.94, 1.06)	0.880	0.965
IS	Blood	DNPH1	IVW	4	1.01 (0.92, 1.1)	0.862	1.000
IS	Blood	DOCK6	Wald ratio	1	1.01 (0.82, 1.25)	0.920	1.000
IS	Blood	DOCK6-AS1	Wald ratio	1	1 (0.96, 1.04)	0.980	1.000
IS	Blood	ELOF1	IVW	2	1.04 (0.96, 1.12)	0.360	1.000
IS	Blood	ENSG00000226104	Wald ratio	1	1.14 (0.98, 1.31)	0.083	0.705
IS	Blood	ENSG00000229391	MR Egger	3	1 (0.91, 1.11)	0.984	0.990
IS	Blood	ENSG00000229391	Weighted median	3	1.02 (0.97, 1.08)	0.405	0.704
IS	Blood	ENSG00000229391	IVW	3	1.03 (0.98, 1.09)	0.229	1.000
IS	Blood	ENSG00000231113	Wald ratio	1	1.08 (1.01, 1.16)	0.020	0.420
IS	Blood	ENSG00000234515	Wald ratio	1	0.99 (0.85, 1.15)	0.883	1.000
IS	Blood	ENSG00000267174	MR Egger	3	0.89 (0.85, 0.94)	0.149	0.990
IS	Blood	ENSG00000267174	Weighted median	3	0.97 (0.95, 0.99)	0.015	0.162
IS	Blood	ENSG00000267174	IVW	3	0.98 (0.92, 1.04)	0.503	1.000
IS	Blood	EPOR	Wald ratio	1	1 (0.94, 1.08)	0.920	1.000
IS	Blood	GNMT	MR Egger	3	1.02 (0.91, 1.13)	0.808	0.990
IS	Blood	GNMT	Weighted median	3	0.98 (0.94, 1.02)	0.236	0.528

IS	Blood	GNMT	IVW	3	0.97 (0.93, 1.02)	0.215	1.000
IS	Blood	HLA-DMA	Wald ratio	1	1.02 (0.95, 1.11)	0.543	1.000
IS	Blood	HLA-DOB	MR Egger	3	1.01 (0.81, 1.25)	0.947	0.990
IS	Blood	HLA-DOB	Weighted median	3	1.04 (1, 1.08)	0.045	0.286
IS	Blood	HLA-DOB	IVW	3	1.04 (1, 1.08)	0.031	0.530
IS	Blood	HLA-DPB1	MR Egger	3	1 (0.77, 1.31)	0.990	0.990
IS	Blood	HLA-DPB1	Weighted median	3	0.98 (0.95, 1.02)	0.424	0.715
IS	Blood	HLA-DPB1	IVW	3	0.98 (0.95, 1.02)	0.387	1.000
IS	Blood	HLA-DQA2	IVW	2	1 (0.98, 1.02)	0.826	1.000
IS	Blood	HLA-DQB1	MR Egger	3	1.04 (0.95, 1.15)	0.539	0.990
IS	Blood	HLA-DQB1	Weighted median	3	1.05 (1, 1.11)	0.062	0.314
IS	Blood	HLA-DQB1	IVW	3	1.05 (1, 1.1)	0.057	0.601
IS	Blood	HLA-DQB1-AS1	IVW	2	1.01 (0.98, 1.04)	0.506	1.000
IS	Blood	HLA-DQB2	Wald ratio	1	0.91 (0.8, 1.03)	0.128	0.902
IS	Blood	HLA-DRB1	MR Egger	4	1.01 (0.99, 1.04)	0.467	0.990
IS	Blood	HLA-DRB1	Weighted median	4	1 (0.99, 1.02)	0.742	0.913
IS	Blood	HLA-DRB1	IVW	4	1 (0.99, 1.02)	0.715	1.000
IS	Blood	HLA-DRB5	MR Egger	5	1.01 (0.91, 1.11)	0.924	0.990
IS	Blood	HLA-DRB5	Weighted median	5	0.97 (0.93, 1.01)	0.181	0.500
IS	Blood	HLA-DRB5	IVW	5	0.97 (0.93, 1.01)	0.136	0.902
IS	Blood	ICAM1	Wald ratio	1	0.96 (0.87, 1.07)	0.474	1.000
IS	Blood	ICAM3	IVW	2	1.02 (0.97, 1.07)	0.499	1.000
IS	Blood	ILF3	Wald ratio	1	0.59 (0.42, 0.82)	0.002	0.068
IS	Blood	ILF3-DT	MR Egger	3	0.94 (0.85, 1.05)	0.484	0.990
IS	Blood	ILF3-DT	Weighted median	3	1 (0.95, 1.04)	0.828	0.946
IS	Blood	ILF3-DT	IVW	3	1 (0.9, 1.1)	0.971	1.000
IS	Blood	KANK2	Wald ratio	1	0.99 (0.88, 1.12)	0.883	1.000
IS	Blood	KEAP1	IVW	2	0.9 (0.62, 1.3)	0.579	1.000

IS	Blood	KLHDC3	Wald ratio	1	0.84 (0.71, 1)	0.047	0.541
IS	Blood	KRI1	IVW	2	0.97 (0.91, 1.04)	0.438	1.000
IS	Blood	LDLR	Wald ratio	1	0.79 (0.61, 1.03)	0.083	0.705
IS	Blood	LY6G5C	MR Egger	3	1.02 (0.95, 1.11)	0.652	0.990
IS	Blood	LY6G5C	Weighted median	3	1.01 (0.98, 1.05)	0.537	0.755
IS	Blood	LY6G5C	IVW	3	1.01 (0.98, 1.05)	0.555	1.000
IS	Blood	MAD2L1BP	MR Egger	3	0.92 (0.87, 0.98)	0.243	0.990
IS	Blood	MAD2L1BP	Weighted median	3	0.94 (0.9, 0.98)	0.002	0.043
IS	Blood	MAD2L1BP	IVW	3	0.94 (0.9, 0.98)	0.002	0.068
IS	Blood	MEA1	MR Egger	3	0.62 (0.37, 1.05)	0.328	0.990
IS	Blood	MEA1	Weighted median	3	0.86 (0.71, 1.05)	0.141	0.477
IS	Blood	MEA1	IVW	3	0.85 (0.72, 0.99)	0.039	0.541
IS	Blood	MRPL2	IVW	2	1.01 (0.93, 1.1)	0.732	1.000
IS	Blood	MRPL4	Wald ratio	1	1 (0.92, 1.09)	0.920	1.000
IS	Blood	MRPS18A	Wald ratio	1	1.07 (0.72, 1.6)	0.729	1.000
IS	Blood	MSH5	IVW	2	1.04 (0.97, 1.11)	0.249	1.000
IS	Blood	PDE4A	IVW	2	0.94 (0.83, 1.07)	0.374	1.000
IS	Blood	PEX6	MR Egger	6	1.13 (1.04, 1.24)	0.049	0.990
IS	Blood	PEX6	Weighted median	6	1.01 (0.97, 1.05)	0.525	0.755
IS	Blood	PEX6	IVW	6	0.99 (0.93, 1.07)	0.867	1.000
IS	Blood	PLPPR2	IVW	2	1 (0.91, 1.1)	0.951	1.000
IS	Blood	POLR1C	Wald ratio	1	0.92 (0.76, 1.11)	0.399	1.000
IS	Blood	PPP2R5D	IVW	2	0.94 (0.81, 1.1)	0.435	1.000
IS	Blood	PRKCSH	IVW	2	0.89 (0.81, 0.99)	0.033	0.530
IS	Blood	PSMB9	MR Egger	4	0.84 (0.7, 1)	0.193	0.990
IS	Blood	PSMB9	Weighted median	4	0.97 (0.93, 1.02)	0.207	0.500
IS	Blood	PSMB9	IVW	4	0.98 (0.93, 1.03)	0.377	1.000
IS	Blood	PTCRA	Wald ratio	1	0.98 (0.78, 1.23)	0.878	1.000

IS	Blood	RGL3	IVW	2	1.03 (0.96, 1.11)	0.349	1.000
IS	Blood	RNF5	IVW	2	0.98 (0.91, 1.04)	0.483	1.000
IS	Blood	RPL7L1	Wald ratio	1	0.99 (0.94, 1.05)	0.719	1.000
IS	Blood	RRP36	IVW	2	1.02 (0.78, 1.33)	0.891	1.000
IS	Blood	S1PR5	IVW	2	0.95 (0.84, 1.07)	0.418	1.000
IS	Blood	SLC44A2	MR Egger	6	1.01 (0.87, 1.18)	0.892	0.990
IS	Blood	SLC44A2	Weighted median	6	1.03 (0.97, 1.09)	0.298	0.606
IS	Blood	SLC44A2	IVW	6	1.02 (0.96, 1.09)	0.508	1.000
IS	Blood	SMARCA4	Wald ratio	1	1.2 (1.08, 1.33)	0.001	0.037
IS	Blood	SPC24	Wald ratio	1	0.94 (0.79, 1.12)	0.498	1.000
IS	Blood	SWSAP1	Wald ratio	1	1.01 (0.9, 1.12)	0.920	1.000
IS	Blood	TAP2	MR Egger	3	0.97 (0.93, 1)	0.324	0.990
IS	Blood	TAP2	Weighted median	3	0.98 (0.95, 1.01)	0.118	0.443
IS	Blood	TAP2	IVW	3	0.98 (0.95, 1.02)	0.304	1.000
IS	Blood	TIMM29	Wald ratio	1	1.24 (1.1, 1.39)	3.27E-04	0.037
IS	Blood	TMED1	IVW	2	0.96 (0.89, 1.04)	0.291	1.000
IS	Blood	TMEM205	IVW	2	1 (0.94, 1.07)	0.894	1.000
IS	Blood	TNXB	IVW	2	1.03 (0.99, 1.07)	0.142	0.902
IS	Blood	TSPAN16	Wald ratio	1	0.99 (0.9, 1.1)	0.920	1.000
IS	Blood	VPS52	Wald ratio	1	1.03 (0.89, 1.19)	0.734	1.000
IS	Blood	VWA7	MR Egger	4	1.09 (0.71, 1.68)	0.720	0.990
IS	Blood	VWA7	Weighted median	4	0.97 (0.9, 1.05)	0.449	0.728
IS	Blood	VWA7	IVW	4	1 (0.92, 1.09)	0.994	1.000
IS	Blood	ZNF20	Wald ratio	1	0.98 (0.85, 1.14)	0.840	1.000
IS	Blood	ZNF318	MR Egger	3	1.17 (0.88, 1.56)	0.470	0.990
IS	Blood	ZNF318	Weighted median	3	1.02 (0.96, 1.08)	0.626	0.835
IS	Blood	ZNF318	IVW	3	1.01 (0.94, 1.07)	0.865	1.000
IS	Blood	ZNF441	Wald ratio	1	0.86 (0.7, 1.07)	0.184	1.000

IS	Blood	ZNF491	Wald ratio	1	1.05 (0.88, 1.26)	0.586	1.000
IS	Blood	ZNF844	Wald ratio	1	1.14 (0.97, 1.34)	0.108	0.810

FDR < 0.05 was considered as significant.

Table S14. Cochran Q and MR Egger pleiotropy tests of MR analysis between circulating genes and CAD/IS.

Outcome	Tissue	Genes	IVW Cochran Q test		MR-Egger		
			Q-statistic	Q_p	intercept	SE	*pval*
CAD	Blood	ADGRG3	0.970	0.325	-	-	-
CAD	Blood	ADGRG5	1.569	0.210	-	-	-
CAD	Blood	AMIGO1	-	-	-	-	-
CAD	Blood	ARHGAP9	7.318	0.007	-	-	-
CAD	Blood	ARID1A	0.002	0.961	-	-	-
CAD	Blood	ARL2BP	1.998	0.368	-0.008	0.008	0.481
CAD	Blood	ARL6IP4	-	-	-	-	-
CAD	Blood	ASB16-AS1	1.161	0.560	-0.009	0.018	0.697
CAD	Blood	ATP23	0.037	0.847	-	-	-
CAD	Blood	ATP6V0A2	1.722	0.189	-	-	-
CAD	Blood	ATP6V1B2	0.000	0.999	-	-	-
CAD	Blood	AVIL	-	-	-	-	-
CAD	Blood	BBS2	4.636	0.462	0.005	0.007	0.524
CAD	Blood	BFSP1	22.808	0.000	-	-	-
CAD	Blood	BRCA1	-	-	-	-	-
CAD	Blood	CCDC92	5.049	0.025	-	-	-
CAD	Blood	CD320	3.513	0.476	-0.004	0.004	0.392
CAD	Blood	CD52	2.285	0.515	-0.013	0.009	0.289
CAD	Blood	CEP85	0.147	0.702	-	-	-
CAD	Blood	CERS4	6.005	0.111	0.005	0.012	0.700
CAD	Blood	CETP	9.596	0.002	-	-	-
CAD	Blood	CLASRP	-	-	-	-	-
CAD	Blood	CLCC1	-	-	-	-	-
CAD	Blood	CNKSR1	0.818	0.366	-	-	-
CAD	Blood	COQ9	-	-	-	-	-
CAD	Blood	CPNE2	0.651	0.885	0.003	0.004	0.590
CAD	Blood	CSGALNACT1	6.649	0.156	0.006	0.005	0.312
CAD	Blood	CYB561D1	-	-	-	-	-
CAD	Blood	DCTN2	-	-	-	-	-
CAD	Blood	DDIT3	-	-	-	-	-
CAD	Blood	DSTN	5.027	0.285	0.009	0.005	0.198
CAD	Blood	EEF1AKMT3	8.568	0.380	-0.018	0.009	0.084
CAD	Blood	EEIG2	-	-	-	-	-
CAD	Blood	EIF2B1	6.266	0.044	-0.006	0.038	0.895
CAD	Blood	ELAPOR1	10.458	0.063	-0.021	0.009	0.086
CAD	Blood	ENSG00000205361	-	-	-	-	-
CAD	Blood	ENSG00000224295	-	-	-	-	-
CAD	Blood	ENSG00000225113	-	-	-	-	-
CAD	Blood	ENSG00000234553	-	-	-	-	-

CAD	Blood	ENSG00000250091	-	-	-	-	-
CAD	Blood	ENSG00000257499	-	-	-	-	-
CAD	Blood	ENSG00000260145	0.802	0.370	-	-	-
CAD	Blood	ENSG00000260828	-	-	-	-	-
CAD	Blood	ENSG00000261114	-	-	-	-	-
CAD	Blood	ENSG00000270028	-	-	-	-	-
CAD	Blood	FGR	-	-	-	-	-
CAD	Blood	FHIP1B	0.584	0.900	0.004	0.007	0.635
CAD	Blood	GALNT2	2.410	0.121	-	-	-
CAD	Blood	GIHCG	-	-	-	-	-
CAD	Blood	GNAO1	0.697	0.706	-0.006	0.007	0.568
CAD	Blood	GPBP1	-	-	-	-	-
CAD	Blood	GPN2	-	-	-	-	-
CAD	Blood	GRN	0.307	0.579	-	-	-
CAD	Blood	GSTM1	17.671	0.024	0.009	0.024	0.728
CAD	Blood	GTF2H3	-	-	-	-	-
CAD	Blood	HBG2	0.373	0.541	-	-	-
CAD	Blood	HERPUD1	22.954	0.000	0.033	0.007	0.131
CAD	Blood	HMGN2	-	-	-	-	-
CAD	Blood	IFI35	1.928	0.165	-	-	-
CAD	Blood	IGSF23	-	-	-	-	-
CAD	Blood	INTS10	3.166	0.075	-	-	-
CAD	Blood	IRS1	7.234	0.027	0.013	0.013	0.509
CAD	Blood	KMT5A	2.522	0.283	-0.109	0.282	0.765
CAD	Blood	LINC01948	-	-	-	-	-
CAD	Blood	LOC101928728	-	-	-	-	-
CAD	Blood	LPL	33.367	0.000	0.030	0.014	0.170
CAD	Blood	LRP1	-	-	-	-	-
CAD	Blood	LZTS1	0.995	0.318	-	-	-
CAD	Blood	MAN1C1	2.478	0.115	-	-	-
CAD	Blood	MAP3K6	8.111	0.230	-0.001	0.013	0.933
CAD	Blood	MARCHF2	24.586	0.000	0.064	0.013	0.127
CAD	Blood	MARS1	-	-	-	-	-
CAD	Blood	MBD6	0.385	0.535	-	-	-
CAD	Blood	MGME1	1.574	0.813	0.009	0.018	0.664
CAD	Blood	MPP2	4.229	0.040	-	-	-
CAD	Blood	MRPS18A	-	-	-	-	-
CAD	Blood	MT1E	0.324	0.569	-	-	-
CAD	Blood	MT1F	0.828	0.935	0.001	0.006	0.853
CAD	Blood	MT1X	5.240	0.073	-0.005	0.014	0.787
CAD	Blood	MT3	2.874	0.090	-	-	-
CAD	Blood	MYO1F	0.624	0.429	-	-	-
CAD	Blood	NBR2	1.420	0.233	-	-	-
CAD	Blood	NDUFA4L2	-	-	-	-	-

CAD	Blood	NDUFA7	1.302	0.729	-0.003	0.012	0.833
CAD	Blood	NECTIN2	6.899	0.075	0.005	0.006	0.490
CAD	Blood	NEMP1	0.220	0.639	-	-	-
CAD	Blood	NLRC5	2.276	0.321	0.011	0.009	0.416
CAD	Blood	NUDT21	-	-	-	-	-
CAD	Blood	NUP93-DT	-	-	-	-	-
CAD	Blood	OR7D2	2.536	0.638	-0.007	0.005	0.245
CAD	Blood	PCCB	1.967	0.374	-0.039	0.052	0.593
CAD	Blood	PEX6	26.433	0.000	-0.003	0.013	0.831
CAD	Blood	PGBD5	-	-	-	-	-
CAD	Blood	PIGV	0.060	0.807	-	-	-
CAD	Blood	PIP4K2C	-	-	-	-	-
CAD	Blood	PLLP	2.572	0.276	0.004	0.024	0.903
CAD	Blood	PPP2R3A	-	-	-	-	-
CAD	Blood	PSME3IP1	3.146	0.076	-	-	-
CAD	Blood	PSRC1	1.062	0.303	-	-	-
CAD	Blood	RAB11B	-	-	-	-	-
CAD	Blood	RILPL2	4.856	0.183	0.008	0.015	0.660
CAD	Blood	RPS28	-	-	-	-	-
CAD	Blood	RRBP1	3.651	0.161	0.001	0.014	0.958
CAD	Blood	RSPRY1	-	-	-	-	-
CAD	Blood	SH3BGRL3	-	-	-	-	-
CAD	Blood	SLC12A3	27.223	0.000	-	-	-
CAD	Blood	SLC18A1	0.877	0.645	0.019	0.022	0.555
CAD	Blood	SNRNP35	5.366	0.021	-	-	-
CAD	Blood	STAT6	1.189	0.756	-0.013	0.022	0.604
CAD	Blood	STMN1	-	-	-	-	-
CAD	Blood	SYTL1	-	-	-	-	-
CAD	Blood	TCTN2	1.871	0.392	-0.008	0.008	0.519
CAD	Blood	TMED2	-	-	-	-	-
CAD	Blood	TMEM101	3.134	0.077	-	-	-
CAD	Blood	TMUB2	-	-	-	-	-
CAD	Blood	TRIM34	1.110	0.292	-	-	-
CAD	Blood	TRIM5	0.759	0.684	-0.003	0.007	0.769
CAD	Blood	TRIM6	5.882	0.118	0.026	0.048	0.636
CAD	Blood	TRNP1	0.422	0.516	-	-	-
CAD	Blood	TSPAN31	3.255	0.196	0.030	0.018	0.341
CAD	Blood	UBQLNL	7.342	0.119	-0.002	0.007	0.741
CAD	Blood	UBTF	-	-	-	-	-
CAD	Blood	USP24	0.540	0.462	-	-	-
CAD	Blood	ZDHHC18	10.342	0.006	0.031	0.010	0.196
CAD	Blood	ZNF558	3.214	0.073	-	-	-
CAD	Blood	ZNF664	2.498	0.114	-	-	-
CAD	Blood	ZNF683	1.120	0.290	-	-	-

IS	Blood	ACP5	4.618	0.202	0.018	0.009	0.184
IS	Blood	AGER	2.468	0.291	0.016	0.022	0.591
IS	Blood	AP1M2	2.636	0.451	0.009	0.034	0.826
IS	Blood	BICRAL	2.197	0.138	-	-	-
IS	Blood	C19orf38	-	-	-	-	-
IS	Blood	C4A	0.749	0.688	0.002	0.021	0.926
IS	Blood	C4B	1.388	0.239	-	-	-
IS	Blood	CARM1	14.629	0.000	-	-	-
IS	Blood	CCDC159	-	-	-	-	-
IS	Blood	CCND3	-	-	-	-	-
IS	Blood	CDKN2D	-	-	-	-	-
IS	Blood	CNPY3	-	-	-	-	-
IS	Blood	CUL7	4.050	0.044	-	-	-
IS	Blood	DDAH2	0.168	0.682	-	-	-
IS	Blood	DLK2	12.777	0.000	-	-	-
IS	Blood	DNPH1	7.718	0.052	0.022	0.014	0.271
IS	Blood	DOCK6	-	-	-	-	-
IS	Blood	DOCK6-AS1	-	-	-	-	-
IS	Blood	ELOF1	1.229	0.268	-	-	-
IS	Blood	ENSG00000226104	-	-	-	-	-
IS	Blood	ENSG00000229391	1.077	0.584	0.010	0.014	0.603
IS	Blood	ENSG00000231113	-	-	-	-	-
IS	Blood	ENSG00000234515	-	-	-	-	-
IS	Blood	ENSG00000267174	14.545	0.001	0.054	0.014	0.164
IS	Blood	EPOR	-	-	-	-	-
IS	Blood	GNMT	3.076	0.215	-0.014	0.014	0.510
IS	Blood	HLA-DMA	-	-	-	-	-
IS	Blood	HLA-DOB	0.596	0.742	0.012	0.042	0.818
IS	Blood	HLA-DPB1	0.021	0.990	-0.008	0.060	0.913
IS	Blood	HLA-DQA2	0.202	0.653	-	-	-
IS	Blood	HLA-DQB1	0.869	0.648	0.003	0.021	0.920
IS	Blood	HLA-DQB1-AS1	2.471	0.116	-	-	-
IS	Blood	HLA-DQB2	-	-	-	-	-
IS	Blood	HLA-DRB1	1.033	0.793	-0.008	0.009	0.490
IS	Blood	HLA-DRB5	3.908	0.419	-0.011	0.014	0.491
IS	Blood	ICAM1	-	-	-	-	-
IS	Blood	ICAM3	0.770	0.380	-	-	-
IS	Blood	ILF3	-	-	-	-	-
IS	Blood	ILF3-DT	11.441	0.003	0.033	0.022	0.372
IS	Blood	KANK2	-	-	-	-	-
IS	Blood	KEAP1	3.934	0.047	-	-	-
IS	Blood	KLHDC3	-	-	-	-	-
IS	Blood	KRI1	0.082	0.774	-	-	-
IS	Blood	LDLR	-	-	-	-	-

IS	Blood	LY6G5C	0.183	0.913	-0.006	0.015	0.765
IS	Blood	MAD2L1BP	0.595	0.743	0.006	0.010	0.629
IS	Blood	MEA1	1.455	0.483	0.048	0.040	0.442
IS	Blood	MRPL2	1.482	0.223	-	-	-
IS	Blood	MRPL4	-	-	-	-	-
IS	Blood	MRPS18A	-	-	-	-	-
IS	Blood	MSH5	0.354	0.552	-	-	-
IS	Blood	PDE4A	1.152	0.283	-	-	-
IS	Blood	PEX6	21.165	0.001	-0.035	0.011	0.029
IS	Blood	PLPPR2	2.973	0.085	-	-	-
IS	Blood	POLR1C	-	-	-	-	-
IS	Blood	PPP2R5D	1.922	0.166	-	-	-
IS	Blood	PRKCSH	0.792	0.373	-	-	-
IS	Blood	PSMB9	3.658	0.301	0.057	0.033	0.222
IS	Blood	PTCRA	-	-	-	-	-
IS	Blood	RGL3	4.739	0.029	-	-	-
IS	Blood	RNF5	0.533	0.465	-	-	-
IS	Blood	RPL7L1	-	-	-	-	-
IS	Blood	RRP36	5.774	0.016	-	-	-
IS	Blood	S1PR5	1.665	0.197	-	-	-
IS	Blood	SLC44A2	10.212	0.069	0.006	0.036	0.878
IS	Blood	SMARCA4	-	-	-	-	-
IS	Blood	SPC24	-	-	-	-	-
IS	Blood	SWSAP1	-	-	-	-	-
IS	Blood	TAP2	3.639	0.162	0.014	0.010	0.407
IS	Blood	TIMM29	-	-	-	-	-
IS	Blood	TMED1	0.131	0.717	-	-	-
IS	Blood	TMEM205	0.023	0.880	-	-	-
IS	Blood	TNXB	0.011	0.917	-	-	-
IS	Blood	TSPAN16	-	-	-	-	-
IS	Blood	VPS52	-	-	-	-	-
IS	Blood	VWA7	5.964	0.113	-0.038	0.089	0.711
IS	Blood	ZNF20	-	-	-	-	-
IS	Blood	ZNF318	2.337	0.311	-0.074	0.068	0.474
IS	Blood	ZNF441	-	-	-	-	-
IS	Blood	ZNF491	-	-	-	-	-
IS	Blood	ZNF844	-	-	-	-	-

SE denotes standard error.

Table S15. Colocalization between circulating genes and CAD/IS.

Outcome	Tissue	Genes	SNP	Chr	BP	NSNPs	PP.H3	PP.H4
CAD	Blood	ADGRG3	rs727216	16	57718783	7748	0.9999	1.17E-07
CAD	Blood	AMIGO1	rs534135	1	110061625	6024	0.9999	1.72E-51
CAD	Blood	BRCA1	rs34766339	17	41394798	4387	0.9984	0.0011
CAD	Blood	CD320	rs2232767	19	8373533	8130	0.9999	2.04E-15
CAD	Blood	CYB561D1	rs17575205	1	110036998	5847	0.9999	8.17E-40
CAD	Blood	DCTN2	rs11172247	12	57946510	5139	0.9543	0.0019
CAD	Blood	GRN	rs114641762	17	42417164	5358	0.8952	0.0264
CAD	Blood	MAP3K6	rs11548322	1	27733916	4223	0.9764	3.85E-04
CAD	Blood	MARS1	rs3782125	12	57857000	4498	0.9579	1.50E-03
CAD	Blood	MYO1F	rs113576178	19	8596012	7853	0.9999	1.11E-11
CAD	Blood	NBR2	rs1799966	17	41223094	4488	0.9951	4.46E-03
CAD	Blood	NECTIN2	rs17561351	19	45372329	6798	0.9999	5.33E-49
CAD	Blood	NUDT21	rs4784669	16	56481282	7597	0.9999	2.67E-07
CAD	Blood	PPP2R3A	rs12695644	3	135844938	4385	0.9999	3.20E-05
CAD	Blood	PSRC1	rs7528419	1	109817192	5656	0.0065	0.9935
CAD	Blood	RAB11B	rs1136888	19	8536754	7681	0.9999	1.13E-09
CAD	Blood	RPS28	rs2972572	19	8390313	7349	0.9999	8.15E-15
CAD	Blood	STMN1	rs1257156	1	26297984	5035	0.9157	8.51E-03
CAD	Blood	TCTN2	rs11834492	12	124180011	6761	0.9995	0.0000
CAD	Blood	TMUB2	rs11079983	17	42198170	5319	0.9288	0.0054
CAD	Blood	TRIM34	rs10838482	11	5667082	9757	0.9997	3.01E-05
CAD	Blood	TSPAN31	rs56261123	12	58186594	5224	0.7482	0.2198
CAD	Blood	ZNF664	rs7958691	12	124440743	7163	0.9999	6.62E-12
IS	Blood	CDKN2D	rs8108051	19	10661775	5066	0.9936	1.36E-03
IS	Blood	CNPY3	rs4714634	6	42901120	4589	0.9959	1.19E-04
IS	Blood	ENSG00000231113	rs56106855	6	42909931	4523	0.9957	0.0003
IS	Blood	HLA-DOB	rs1044043	6	32793981	5950	0.1092	0.0072
IS	Blood	ILF3	rs12610411	19	10893074	4873	0.9353	0.0593
IS	Blood	KLHDC3	rs4987174	6	42931215	4461	0.9958	0.0002
IS	Blood	MAD2L1BP	rs111549887	6	43469519	4315	0.9924	0.0036
IS	Blood	MEA1	rs116750300	6	42981633	4687	0.9954	0.0006
IS	Blood	PRKCSH	rs160841	19	11559158	5026	0.9948	0.0002
IS	Blood	SMARCA4	rs73013167	19	11126199	5117	0.9855	0.0094
IS	Blood	TIMM29	rs36059375	19	11045531	5264	0.9774	0.0176

Chr denotes chromosome; BP, base pair position; PP.H3 denotes association with CAD or IS risk as well as (apo)lipoprotein measurements, but at distinct causal variants; PP.H4 denotes association with both traits, with a shared causal variant. A PP.H4 greater than 0.8 indicates significant colocalization.

Table S16. Causal associations of multi-tissue gene expression levels with CAD and IS using data from GTEx project.

Outcome	Tissue	Genes	Methods	NSNPs	OR (95%CI)	*pval*
CAD	Whole Blood	ADGRG3	Wald ratio	1	1.07 (0.98, 1.16)	0.111
CAD	Whole Blood	AMIGO1	Wald ratio	1	0.96 (0.92, 0.99)	0.041
CAD	Whole Blood	ARHGAP9	Wald ratio	1	1.02 (0.97, 1.09)	0.418
CAD	Whole Blood	ASB16-AS1	Wald ratio	1	1.05 (1.01, 1.09)	0.016
CAD	Whole Blood	ATP23	Wald ratio	1	1.01 (0.99, 1.02)	0.386
CAD	Whole Blood	ATP6V0A2	Wald ratio	1	1.03 (0.99, 1.07)	0.132
CAD	Whole Blood	BBS2	Wald ratio	1	1.01 (0.99, 1.02)	0.502
CAD	Whole Blood	CCDC92	Wald ratio	1	0.89 (0.85, 0.94)	3.46E-05
CAD	Whole Blood	CD52	Wald ratio	1	0.99 (0.96, 1.01)	0.304
CAD	Whole Blood	CEP85	Wald ratio	1	1.03 (0.98, 1.09)	0.270
CAD	Whole Blood	CERS4	Wald ratio	1	0.98 (0.95, 1.01)	0.273
CAD	Whole Blood	CPNE2	Wald ratio	1	0.98 (0.94, 1.03)	0.53
CAD	Whole Blood	CSGALNACT1	Wald ratio	1	0.97 (0.95, 0.99)	0.007
CAD	Whole Blood	DSTN	Wald ratio	1	0.98 (0.95, 1.02)	0.341
CAD	Whole Blood	EEF1AKMT3	Wald ratio	1	1.01 (0.99, 1.02)	0.274
CAD	Whole Blood	ELAPOR1	Wald ratio	1	1.02 (1.01, 1.03)	0.011
CAD	Whole Blood	FHIP1B	Wald ratio	1	1.04 (1.01, 1.07)	0.015
CAD	Whole Blood	GIHCG	Wald ratio	1	1.02 (0.97, 1.06)	0.451
CAD	Whole Blood	GRN	Wald ratio	1	0.89 (0.83, 0.96)	0.003
CAD	Whole Blood	GSTM1	Wald ratio	1	1.00 (0.98, 1.02)	0.760
CAD	Whole Blood	HBG2	Wald ratio	1	1.00 (0.98, 1.02)	0.726
CAD	Whole Blood	HMGN2	Wald ratio	1	1.07 (0.99, 1.16)	0.091
CAD	Whole Blood	IFI35	Wald ratio	1	0.97 (0.87, 1.08)	0.574
CAD	Whole Blood	INTS10	Wald ratio	1	0.95 (0.90, 1.01)	0.088
CAD	Whole Blood	IRS1	Wald ratio	1	1.01 (0.97, 1.06)	0.528
CAD	Whole Blood	KMT5A	Wald ratio	1	0.99 (0.94, 1.04)	0.673
CAD	Whole Blood	LINC01948	Wald ratio	1	0.96 (0.92, 1.01)	0.086
CAD	Whole Blood	LZTS1	Wald ratio	1	1.01 (0.99, 1.03)	0.355
CAD	Whole Blood	MAP3K6	Wald ratio	1	0.97 (0.93, 1.01)	0.152
CAD	Whole Blood	MARCHF2	Wald ratio	1	1.11 (1.06, 1.15)	1.58E-06
CAD	Whole Blood	MPP2	Wald ratio	1	0.97 (0.93, 1.01)	0.092
CAD	Whole Blood	MYO1F	Wald ratio	1	0.91 (0.80, 1.03)	0.149
CAD	Whole Blood	NECTIN2	Wald ratio	1	1.01 (1.00, 1.02)	0.211
CAD	Whole Blood	NEMP1	Wald ratio	1	1.05 (0.98, 1.11)	0.155
CAD	Whole Blood	NLRC5	Wald ratio	1	1.00 (0.97, 1.03)	0.865
CAD	Whole Blood	PCCB	Wald ratio	1	1.03 (0.97, 1.09)	0.359
CAD	Whole Blood	PEX6	Wald ratio	1	0.98 (0.97, 0.99)	0.009
CAD	Whole Blood	PIGV	Wald ratio	1	0.96 (0.92, 1.01)	0.121
CAD	Whole Blood	PSME3IP1	Wald ratio	1	0.88 (0.81, 0.96)	0.006
CAD	Whole Blood	PSRC1	Wald ratio	1	0.72 (0.69, 0.75)	8.11E-57

CAD	Whole Blood	RPS28	Wald ratio	1	1.14 (1.05, 1.24)	0.002
CAD	Whole Blood	RRBP1	Wald ratio	1	1.19 (1.10, 1.28)	6.67E-06
CAD	Whole Blood	SH3BGRL3	Wald ratio	1	0.96 (0.86, 1.07)	0.436
CAD	Whole Blood	TCTN2	Wald ratio	1	1.01 (0.96, 1.05)	0.791
CAD	Whole Blood	TMEM101	Wald ratio	1	1.00 (0.92, 1.10)	0.945
CAD	Whole Blood	TRIM5	Wald ratio	1	0.94 (0.89, 0.99)	0.023
CAD	Whole Blood	TSPAN31	Wald ratio	1	0.93 (0.89, 0.97)	3.06E-04
CAD	Whole Blood	UBQLNL	Wald ratio	1	1.01 (0.97, 1.05)	0.634
CAD	Whole Blood	ZNF683	Wald ratio	1	0.98 (0.93, 1.03)	0.387
IS	Whole Blood	ACP5	Wald ratio	1	1.00 (0.97, 1.03)	0.884
IS	Whole Blood	AP1M2	Wald ratio	1	1.05 (1.01, 1.10)	0.025
IS	Whole Blood	CCDC159	Wald ratio	1	1.01 (0.86, 1.18)	0.92
IS	Whole Blood	CDKN2D	Wald ratio	1	0.76 (0.62, 0.92)	0.004
IS	Whole Blood	CUL7	Wald ratio	1	1.01 (0.98, 1.05)	0.495
IS	Whole Blood	DNPH1	Wald ratio	1	0.99 (0.90, 1.08)	0.822
IS	Whole Blood	DOCK6-AS1	Wald ratio	1	1.01 (0.96, 1.06)	0.666
IS	Whole Blood	ENSG00000229391	IVW	2	0.99 (0.97, 1.01)	0.24
IS	Whole Blood	ENSG00000267174	Wald ratio	1	0.97 (0.95, 0.99)	0.019
IS	Whole Blood	EPOR	Wald ratio	1	1.00 (0.92, 1.09)	0.92
IS	Whole Blood	HLA-DOB	IVW	2	1.02 (0.99, 1.06)	0.147
IS	Whole Blood	HLA-DPB1	Wald ratio	1	0.93 (0.77, 1.13)	0.492
IS	Whole Blood	HLA-DQA2	IVW	2	1.00 (0.98, 1.02)	0.904
IS	Whole Blood	HLA-DQB2	Wald ratio	1	0.96 (0.90, 1.01)	0.118
IS	Whole Blood	HLA-DRB1	Wald ratio	1	1.00 (0.89, 1.12)	0.983
IS	Whole Blood	HLA-DRB5	Wald ratio	1	1.01 (0.93, 1.10)	0.806
IS	Whole Blood	ICAM3	Wald ratio	1	1.05 (0.93, 1.20)	0.43
IS	Whole Blood	ILF3-DT	Wald ratio	1	0.95 (0.88, 1.03)	0.199
IS	Whole Blood	KANK2	Wald ratio	1	0.99 (0.92, 1.08)	0.883
IS	Whole Blood	KRI1	Wald ratio	1	1.27 (1.09, 1.47)	0.002
IS	Whole Blood	LY6G5C	Wald ratio	1	1.02 (0.99, 1.05)	0.281
IS	Whole Blood	PEX6	Wald ratio	1	1.02 (1.00, 1.05)	0.071
IS	Whole Blood	PLPPR2	Wald ratio	1	1.01 (0.90, 1.13)	0.92
IS	Whole Blood	PPP2R5D	Wald ratio	1	0.93 (0.82, 1.04)	0.2
IS	Whole Blood	PRKCSH	Wald ratio	1	0.82 (0.69, 0.97)	0.023
IS	Whole Blood	PSMB9	Wald ratio	1	1.00 (0.94, 1.06)	0.958
IS	Whole Blood	RGL3	Wald ratio	1	1.06 (1.01, 1.11)	0.026
IS	Whole Blood	SLC44A2	Wald ratio	1	1.11 (1.01, 1.21)	0.025
IS	Whole Blood	SPC24	Wald ratio	1	0.97 (0.84, 1.13)	0.696
IS	Whole Blood	SWSAP1	Wald ratio	1	1.01 (0.88, 1.15)	0.92
IS	Whole Blood	TAP2	Wald ratio	1	0.96 (0.90, 1.02)	0.165
IS	Whole Blood	TMED1	Wald ratio	1	0.96 (0.89, 1.03)	0.265
IS	Whole Blood	TMEM205	Wald ratio	1	1.01 (0.88, 1.15)	0.92
IS	Whole Blood	TSPAN16	Wald ratio	1	1.00 (0.91, 1.09)	0.92
CAD	Artery_Aorta	CETP	Wald ratio	1	1.11 (1.07, 1.14)	2.14E-10

CAD	Artery_Aorta	ENSG00000270028	Wald ratio	1	0.95 (0.90, 0.99)	0.013
CAD	Artery_Aorta	PCCB	Wald ratio	1	1.01 (0.98, 1.05)	0.443
IS	Artery_Aorta	DOCK7	Wald ratio	1	0.94 (0.88, 1.01)	0.07
IS	Artery_Aorta	ENSG00000204338	IVW	2	1.00 (0.94, 1.05)	0.903
IS	Artery_Aorta	ENSG00000229391	Wald ratio	1	0.99 (0.94, 1.03)	0.512
IS	Artery_Aorta	GNMT	Wald ratio	1	0.98 (0.95, 1.00)	0.071
IS	Artery_Aorta	HLA-DQA1	IVW	2	1.00 (0.98, 1.02)	0.895
IS	Artery_Aorta	KANK2	Wald ratio	1	0.98 (0.89, 1.08)	0.713
IS	Artery_Aorta	PEX6	Wald ratio	1	1.03 (0.99, 1.06)	0.161
IS	Artery_Aorta	PPP2R5D	Wald ratio	1	0.96 (0.86, 1.07)	0.465
CAD	Artery_Coronary	CETP	Wald ratio	1	1.06 (1.04, 1.08)	2.91E-07
IS	Artery_Coronary	ENSG00000204338	Wald ratio	1	0.97 (0.94, 1.01)	0.098
IS	Artery_Coronary	ENSG00000229391	Wald ratio	1	1.01 (0.95, 1.06)	0.796
IS	Artery_Coronary	GNMT	Wald ratio	1	0.98 (0.96, 1.00)	0.084
IS	Artery_Coronary	HLA-DQA1	Wald ratio	1	1.00 (0.98, 1.02)	0.977
IS	Artery_Coronary	HLA-DQB2	Wald ratio	1	0.98 (0.95, 1.00)	0.058
IS	Artery_Coronary	KANK2	IVW	2	1.01 (0.94, 1.09)	0.742
IS	Artery_Coronary	PEX6	Wald ratio	1	1.02 (0.99, 1.05)	0.191
CAD	Artery_Tibial	ENSG00000250091	Wald ratio	1	0.94 (0.91, 0.96)	4.50E-08
CAD	Artery_Tibial	ENSG00000270028	Wald ratio	1	0.93 (0.91, 0.96)	3.88E-08
CAD	Artery_Tibial	PCCB	Wald ratio	1	1.02 (0.98, 1.07)	0.359
CAD	Artery_Tibial	PSRC1	Wald ratio	1	1.00 (0.97, 1.03)	0.931
IS	Artery_Tibial	DOCK7	Wald ratio	1	0.94 (0.87, 1.01)	0.07
IS	Artery_Tibial	ENSG00000204338	IVW	2	0.99 (0.92, 1.07)	0.806
IS	Artery_Tibial	ENSG00000229391	MR Egger	3	0.97 (0.87, 1.08)	0.653
IS	Artery_Tibial	ENSG00000229391	Weighted median	3	0.99 (0.97, 1.01)	0.33
IS	Artery_Tibial	ENSG00000229391	IVW	3	0.99 (0.97, 1.01)	0.259
IS	Artery_Tibial	ENSG00000229391	Simple mode	3	1.00 (0.97, 1.04)	0.884
IS	Artery_Tibial	ENSG00000229391	Weighted mode	3	0.98 (0.95, 1.00)	0.23
IS	Artery_Tibial	GNMT	Wald ratio	1	0.98 (0.96, 1.00)	0.08
IS	Artery_Tibial	HLA-DQA1	Wald ratio	1	1.04 (0.99, 1.09)	0.109
IS	Artery_Tibial	HLA-DQA1	IVW	2	1.00 (0.98, 1.02)	0.904
IS	Artery_Tibial	HLA-DQB1	Wald ratio	1	1.02 (0.94, 1.11)	0.665
IS	Artery_Tibial	HLA-DQB2	IVW	2	0.99 (0.96, 1.02)	0.417
IS	Artery_Tibial	HLA-DRB1	Wald ratio	1	1.07 (0.90, 1.27)	0.453
IS	Artery_Tibial	KANK2	Wald ratio	1	1.02 (0.94, 1.12)	0.611
CAD	Heart_Atrial_Appendage	CETP	Wald ratio	1	1.08 (1.05, 1.10)	7.60E-11
CAD	Heart_Atrial_Appendage	ENSG00000250091	Wald ratio	1	0.93 (0.91, 0.96)	4.41E-08
CAD	Heart_Atrial_Appendage	PCCB	Wald ratio	1	1.02 (0.97, 1.07)	0.389
CAD	Heart_Atrial_Appendage	PSRC1	Wald ratio	1	1.00 (0.97, 1.03)	0.931
IS	Heart_Atrial_Appendage	ENSG00000204338	IVW	2	0.99 (0.93, 1.06)	0.841
IS	Heart_Atrial_Appendage	ENSG00000229391	MR Egger	3	0.99 (0.91, 1.07)	0.811
IS	Heart_Atrial_Appendage	ENSG00000229391	Weighted median	3	1.00 (0.98, 1.01)	0.873
IS	Heart_Atrial_Appendage	ENSG00000229391	IVW	3	1.00 (0.98, 1.01)	0.937

IS	Heart_Atrial_Appendage	ENSG00000229391	Simple mode	3	1.00 (0.98, 1.02)	0.903
IS	Heart_Atrial_Appendage	ENSG00000229391	Weighted mode	3	1.00 (0.98, 1.02)	0.897
IS	Heart_Atrial_Appendage	GNMT	Wald ratio	1	0.98 (0.95, 1.00)	0.084
IS	Heart_Atrial_Appendage	HLA-DQA1	IVW	2	1.03 (1.00, 1.06)	0.037
IS	Heart_Atrial_Appendage	HLA-DQA1	MR Egger	3	0.99 (0.93, 1.06)	0.813
IS	Heart_Atrial_Appendage	HLA-DQA1	Weighted median	3	1.00 (0.98, 1.02)	0.922
IS	Heart_Atrial_Appendage	HLA-DQA1	IVW	3	1.00 (0.98, 1.02)	0.879
IS	Heart_Atrial_Appendage	HLA-DQA1	Simple mode	3	1.00 (0.98, 1.03)	0.887
IS	Heart_Atrial_Appendage	HLA-DQA1	Weighted mode	3	1.00 (0.98, 1.02)	0.994
IS	Heart_Atrial_Appendage	HLA-DRB1	Wald ratio	1	1.00 (0.97, 1.03)	0.931
IS	Heart_Atrial_Appendage	KANK2	Wald ratio	1	1.02 (0.98, 1.08)	0.329
IS	Heart_Atrial_Appendage	PEX6	Wald ratio	1	1.02 (1.00, 1.04)	0.097
IS	Heart_Atrial_Appendage	RPL7L1	Wald ratio	1	0.98 (0.88, 1.08)	0.628
CAD	Heart_Left_Ventricle	ENSG00000250091	Wald ratio	1	0.94 (0.92, 0.96)	6.25E-08
CAD	Heart_Left_Ventricle	ENSG00000270028	Wald ratio	1	0.91 (0.88, 0.94)	3.96E-09
CAD	Heart_Left_Ventricle	PSRC1	Wald ratio	1	0.71 (0.68, 0.74)	8.11E-57
IS	Heart_Left_Ventricle	ENSG00000204338	Wald ratio	1	1.03 (0.98, 1.08)	0.237
IS	Heart_Left_Ventricle	ENSG00000229391	MR Egger	3	0.96 (0.88, 1.05)	0.541
IS	Heart_Left_Ventricle	ENSG00000229391	Weighted median	3	0.99 (0.97, 1.01)	0.332
IS	Heart_Left_Ventricle	ENSG00000229391	IVW	3	0.99 (0.98, 1.01)	0.254
IS	Heart_Left_Ventricle	ENSG00000229391	Simple mode	3	1.00 (0.98, 1.03)	0.897
IS	Heart_Left_Ventricle	ENSG00000229391	Weighted mode	3	0.98 (0.96, 1.00)	0.224
IS	Heart_Left_Ventricle	GNMT	Wald ratio	1	0.98 (0.95, 1.00)	0.084
IS	Heart_Left_Ventricle	HLA-DQA1	IVW	2	1.00 (0.98, 1.02)	0.894
IS	Heart_Left_Ventricle	HLA-DQB1	Wald ratio	1	1.02 (1.00, 1.05)	0.058
IS	Heart_Left_Ventricle	HLA-DQB2	IVW	2	0.96 (0.93, 1.00)	0.068
IS	Heart_Left_Ventricle	HLA-DRB1	Wald ratio	1	0.97 (0.89, 1.06)	0.529
IS	Heart_Left_Ventricle	KANK2	IVW	2	1.05 (0.98, 1.12)	0.168
IS	Heart_Left_Ventricle	PEX6	Wald ratio	1	1.02 (1.00, 1.04)	0.084
CAD	Liver	CCDC92	Wald ratio	1	0.94 (0.92, 0.96)	3.60E-07
CAD	Liver	CELSR2	Wald ratio	1	0.91 (0.90, 0.92)	8.11E-57
CAD	Liver	PSRC1	Wald ratio	1	0.93 (0.92, 0.94)	8.11E-57
CAD	Liver	SORT1	Wald ratio	1	0.93 (0.92, 0.94)	8.11E-57
CAD	Liver	MSL2	Wald ratio	1	0.87 (0.84, 0.91)	1.78E-13
IS	Liver	PEX6	Wald ratio	1	1.02 (1.00, 1.04)	0.084
IS	Liver	ENSG00000229391	IVW	2	0.99 (0.97, 1.00)	0.159
IS	Liver	HLA-DQA1	Wald ratio	1	1.01 (0.98, 1.04)	0.422

IVW denotes inverse variance weighted method; *FDR* < 0.05 was considered as nominally significant.

Table S17. Colocalization between multi-tissue gene expression levels and CAD/IS.

Outcome	Tissue	Genes	SNP	Chr	BP	NSNPs	PP.H3	PP.H4
CAD	Blood	AMIGO1	rs2570972	1	109509517	1370	0.999	5.51E-52
CAD	Blood	ASB16-AS1	rs2631299	17	44142405	112	0.002	0.003
CAD	Blood	CCDC92	rs3768	12	124015292	2071	0.99	0.009
CAD	Blood	CSGALNACT1	rs7835354	8	19706638	817	0.999	2.96E-12
CAD	Blood	ELAPOR1	rs17014495	1	109171781	1271	0.999	5.51E-52
CAD	Blood	FHIP1B	rs11040803	11	6204885	1334	0.999	0.003
CAD	Blood	GRN	rs114641762	17	44339796	86	0.999	0.003
CAD	Blood	MARCHF2	rs17160489	19	8443507	1460	0.999	3.57E-04
CAD	Blood	PEX6	rs1768984201	6	42952678	1365	0.999	6.08E-06
CAD	Blood	PSME3IP1	rs370589327	16	57138652	1046	0.999	0.004
CAD	Blood	PSRC1	rs7528419	1	109274570	1341	0.999	5.51E-52
CAD	Blood	RPS28	rs2972574	19	8329208	1382	0.999	1.39E-04
CAD	Blood	RRBP1	rs1052965	20	17619919	940	0.999	0.001
CAD	Blood	TRIM5	rs4567498	11	5669775	1081	0.999	0.003
CAD	Blood	TSPAN31	rs61938185	12	57807992	1106	0.999	0.009
IS	Blood	AP1M2	rs150540855	19	10596297	2325	0.986	6.04E-05
IS	Blood	CDKN2D	rs11672361	19	10560702	2313	0.986	6.04E-05
IS	Blood	ENSG00000267174	rs420703	19	11301192	2170	0.986	5.78E-05
IS	Blood	KRI1	rs4310980	19	10558848	2311	0.986	6.04E-05
IS	Blood	PRKCSH	rs8101357	19	11449938	2277	0.986	5.78E-05
IS	Blood	RGL3	rs401564	19	11399390	2210	0.986	5.78E-05
IS	Blood	SLC44A2	rs150540855	19	10596297	2325	0.986	6.04E-05
CAD	Artery Aorta	ENSG00000270028	rs4765568	12	123994614	1799	0.999	2.42E-06
CAD	Artery Aorta	CETP	rs247617	16	56956804	1228	0.999	3.08E-08
CAD	Artery Coronary	CETP	rs1800775	16	56961324	676	0.736	0.0011
CAD	Artery Tibial	ENSG00000270028	rs12317176	12	123920171	2240	0.999	0.0009

CAD	Artery Tibial	ENSG00000250091	rs4930726	12	123943784	2235	0.999	0.0009
CAD	Heart Atrial Appendage	ENSG00000250091	rs10846580	12	123930906	1577	0.999	1.89E-06
CAD	Heart Atrial Appendage	CETP	rs56156922	16	56953457	685	0.999	4.01E-08
IS	Heart Atrial Appendage	HLA-DQA1	rs9272320	6	32636347	8950	0.323	0.0043
CAD	Heart Left Ventricle	ENSG00000270028	rs7133378	12	123924955	1466	0.999	2.39E-06
CAD	Heart Left Ventricle	ENSG00000250091	rs9971695	12	123928944	1463	0.999	2.39E-06
CAD	Heart Left Ventricle	PSRC1	rs7528419	1	109274570	566	0.999	1.11E-26
CAD	Liver	CCDC92	rs1054852	12	124011769	861	0.9992	1.51E-06
CAD	Liver	CELSR2	rs7528419	1	109274570	481	0.9999	1.83E-05
CAD	Liver	PSRC1	rs7528419	1	109274570	481	0.9999	1.83E-05
CAD	Liver	SORT1	rs7528419	1	109274570	481	0.9999	1.83E-05
CAD	Liver	MSL2	rs13081352	3	136222574	264	0.9984	0.0016

Chr denotes chromosome; BP, base pair position; PP.H3 denotes association with CAD or IS risk as well as (apo)lipoprotein measurements, but at distinct causal variants; PP.H4 denotes association with both traits, with a shared causal variant. A PP.H4 greater than 0.8 indicates significant colocalization.

Table S18. Causal associations of circulating *PSRC1* expression with traditional circulating lipids and C-reactive protein (CRP).

Outcome	Tissue	Gene	Methods	NSNPs	OR (95%CI)	*pval*
LDL-c	Whole Blood	PSRC1	IVW	2	0.74 (0.69, 0.79)	8.43E-22
TC	Whole Blood	PSRC1	IVW	2	0.78 (0.73, 0.82)	1.53E-19
TG	Whole Blood	PSRC1	IVW	2	0.98 (0.97, 0.99)	1.91E-09
CRP	Whole Blood	PSRC1	IVW	2	1.03 (0.99, 1.07)	0.075

IVW denotes inverse variance weighted.

Table S19. The nine *PSRC1*-associated circulating proteins.

Proteins	SNP	Chr	BP	Effect Allele	Alternate Allele	Beta	SE	*pval*
Apolipoprotein B	rs7528419	1	109817192	G	A	-0.1378	0.0302	5.13E-06
Carbonic anhydrase-related protein 10	rs7528419	1	109817192	G	A	-0.2967	0.0299	3.02E-23
Complement C1q tumor necrosis factor-related protein 1	rs7528419	1	109817192	G	A	-0.6366	0.0282	1.07E-112
Four-jointed box protein 1	rs7528419	1	109817192	G	A	-0.2628	0.03	1.82E-18
Granulins	rs7528419	1	109817192	G	A	-0.8234	0.0267	1.23E-208
Group XIIB secretory phospholipase A2-like protein	rs7528419	1	109817192	G	A	-0.4238	0.0294	4.17E-47
Hemojuvelin	rs7528419	1	109817192	G	A	-0.2292	0.03	2.40E-14
Neogenin	rs7528419	1	109817192	G	A	0.2287	0.0301	2.75E-14
Sodium-coupled monocarboxylate transporter 1	rs7528419	1	109817192	G	A	-0.1612	0.0302	9.33E-08

Chr denotes chromosome; BP, base pair position; SE, standard error.

Table S20. Genetic instruments for *PSRC1*-associated circulating proteins to CAD in two-sample MR analysis.

Exposure (Proteins)	SNP	Chr	Effect Allele	Alternate Allele	EAF	Beta	SE	*pval*	r^2	F statistic	Outcome
Apolipoprotein B	rs10001793	4	C	T	0.425	0.118	0.025	3.39E-06	0.0067	22.4189	CAD
Apolipoprotein B	rs10024435	4	A	C	0.225	-0.130	0.029	8.71E-06	0.0059	19.5911	CAD
Apolipoprotein B	rs1065853	19	T	G	0.078	-0.427	0.046	1.48E-20	0.0261	88.3968	CAD
Apolipoprotein B	rs114401436	5	T	C	0.017	-0.425	0.096	9.33E-06	0.0062	20.5063	CAD
Apolipoprotein B	rs11591147	1	T	G	0.018	-0.471	0.094	4.79E-07	0.0078	26.0451	CAD
Apolipoprotein B	rs117069514	15	C	T	0.028	-0.357	0.077	3.89E-06	0.0069	22.9995	CAD
Apolipoprotein B	rs1190618	14	C	G	0.773	0.139	0.030	4.90E-06	0.0068	22.4333	CAD
Apolipoprotein B	rs12331051	4	T	C	0.516	0.128	0.025	2.69E-07	0.0081	27.0061	CAD
Apolipoprotein B	rs12869811	13	G	A	0.073	0.231	0.047	9.77E-07	0.0072	23.8863	CAD
Apolipoprotein B	rs13437646	7	C	A	0.165	0.152	0.034	6.61E-06	0.0064	21.1536	CAD
Apolipoprotein B	rs144559255	11	T	C	0.026	0.360	0.078	3.55E-06	0.0066	21.8758	CAD
Apolipoprotein B	rs144879195	1	C	A	0.011	-0.594	0.129	4.27E-06	0.0077	25.6263	CAD
Apolipoprotein B	rs145012582	7	T	C	0.008	-0.609	0.137	9.12E-06	0.0061	20.4079	CAD
Apolipoprotein B	rs147711004	19	A	G	0.041	0.291	0.063	4.17E-06	0.0067	22.1840	CAD
Apolipoprotein B	rs216313	12	G	A	0.071	0.245	0.054	4.79E-06	0.0079	26.3692	CAD
Apolipoprotein B	rs2424455	20	G	A	0.849	-0.169	0.035	1.32E-06	0.0073	24.3610	CAD
Apolipoprotein B	rs2637886	1	C	T	0.526	0.114	0.026	8.91E-06	0.0065	21.5186	CAD
Apolipoprotein B	rs2731674	5	G	T	0.753	0.128	0.028	6.03E-06	0.0061	20.1017	CAD
Apolipoprotein B	rs3792773	5	A	G	0.037	0.312	0.066	2.24E-06	0.0070	23.1988	CAD
Apolipoprotein B	rs4144527	5	T	C	0.838	0.154	0.034	5.01E-06	0.0064	21.3409	CAD
Apolipoprotein B	rs4970836	1	A	G	0.771	0.140	0.030	2.69E-06	0.0069	22.9562	CAD
Apolipoprotein B	rs520354	2	G	A	0.467	0.127	0.025	2.19E-07	0.0081	26.7850	CAD
Apolipoprotein B	rs548403247	3	A	C	0.043	0.317	0.071	7.24E-06	0.0083	27.4839	CAD
Apolipoprotein B	rs56309410	1	G	T	0.239	-0.129	0.029	9.77E-06	0.0060	20.0051	CAD
Apolipoprotein B	rs6993770	8	T	A	0.282	0.123	0.027	7.59E-06	0.0061	20.2805	CAD
Apolipoprotein B	rs716410	6	G	A	0.486	-0.113	0.025	5.89E-06	0.0064	21.1806	CAD

Apolipoprotein B	rs72778456	16	T	C	0.084	0.210	0.046	4.68E-06	0.0068	22.4212	CAD
Apolipoprotein B	rs73035300	7	A	G	0.032	-0.358	0.079	6.17E-06	0.0079	26.3672	CAD
Apolipoprotein B	rs73113369	3	C	T	0.075	0.220	0.048	4.17E-06	0.0066	22.0707	CAD
Apolipoprotein B	rs73828908	4	A	G	0.080	-0.216	0.046	2.95E-06	0.0069	22.7879	CAD
Apolipoprotein B	rs74585152	3	C	T	0.027	-0.351	0.078	7.76E-06	0.0064	21.2118	CAD
Apolipoprotein B	rs7802161	7	A	T	0.048	0.269	0.060	6.92E-06	0.0066	21.8562	CAD
Apolipoprotein B	rs8067134	17	T	C	0.422	-0.111	0.025	8.32E-06	0.0060	20.0601	CAD
Apolipoprotein B	rs9358914	6	T	G	0.309	0.126	0.027	2.34E-06	0.0068	22.6617	CAD
Apolipoprotein B	rs9514807	13	C	G	0.360	-0.117	0.026	8.71E-06	0.0063	20.9421	CAD
Complement C1q tumor necrosis factor-related protein 1	rs10762481	10	C	T	0.383	-0.185	0.025	3.72E-13	0.0161	53.9880	CAD
Complement C1q tumor necrosis factor-related protein 1	rs111219454	12	C	T	0.101	0.204	0.046	8.51E-06	0.0075	25.0619	CAD
Complement C1q tumor necrosis factor-related protein 1	rs112305118	13	T	C	0.039	-0.311	0.068	5.62E-06	0.0072	23.8419	CAD
Complement C1q tumor necrosis factor-related protein 1	rs112453666	15	A	G	0.038	-0.324	0.070	3.72E-06	0.0077	25.4749	CAD
Complement C1q tumor necrosis factor-related protein 1	rs112635299	14	T	G	0.023	-0.584	0.084	3.16E-12	0.0151	50.4897	CAD
Complement C1q tumor necrosis factor-related protein 1	rs113634207	2	A	G	0.030	0.328	0.074	8.91E-06	0.0062	20.5990	CAD
Complement C1q tumor necrosis factor-related protein 1	rs116451478	1	G	A	0.065	-0.231	0.052	8.91E-06	0.0065	21.4843	CAD
Complement C1q tumor necrosis factor-related protein 1	rs138242461	6	T	C	0.019	0.505	0.102	7.08E-07	0.0097	32.1934	CAD
Complement C1q tumor necrosis factor-related protein 1	rs139201499	17	A	G	0.053	0.289	0.063	4.57E-06	0.0084	28.0530	CAD
Complement C1q tumor necrosis factor-related protein 1	rs139242303	14	G	T	0.015	0.486	0.102	2.04E-06	0.0072	23.8104	CAD
Complement C1q tumor necrosis factor-related protein 1	rs140128652	6	A	T	0.013	0.537	0.121	8.51E-06	0.0074	24.4826	CAD
Complement C1q tumor necrosis factor-related protein 1	rs17047444	3	C	T	0.216	-0.148	0.033	6.76E-06	0.0074	24.5824	CAD
Complement C1q tumor necrosis factor-related protein 1	rs2061596	11	T	C	0.125	0.169	0.037	5.25E-06	0.0062	20.6979	CAD
Complement C1q tumor necrosis factor-related protein 1	rs2642704	5	C	A	0.420	-0.113	0.025	7.94E-06	0.0063	20.8039	CAD
Complement C1q tumor necrosis factor-related protein 1	rs490748	1	T	G	0.422	-0.130	0.026	5.01E-07	0.0082	27.2151	CAD
Complement C1q tumor necrosis factor-related protein 1	rs5848	17	T	C	0.287	-0.248	0.028	1.26E-18	0.0253	85.4595	CAD
Complement C1q tumor necrosis factor-related protein 1	rs62653756	10	C	G	0.072	-0.217	0.049	7.59E-06	0.0063	21.0617	CAD
Complement C1q tumor necrosis factor-related protein 1	rs646776	1	T	C	0.777	0.640	0.028	1.82E-114	0.1421	546.4714	CAD
Complement C1q tumor necrosis factor-related protein 1	rs7259081	19	A	T	0.751	0.174	0.031	3.16E-08	0.0113	37.7124	CAD

Complement C1q tumor necrosis factor-related protein 1	rs72644662	1	T	C	0.028	-0.376	0.082	4.47E-06	0.0077	25.6142	CAD
Complement C1q tumor necrosis factor-related protein 1	rs72802395	16	A	G	0.086	-0.255	0.044	9.77E-09	0.0102	33.9659	CAD
Complement C1q tumor necrosis factor-related protein 1	rs7535434	1	T	G	0.279	-0.124	0.028	9.77E-06	0.0062	20.5311	CAD
Complement C1q tumor necrosis factor-related protein 1	rs78308677	20	G	C	0.029	-0.415	0.082	3.89E-07	0.0096	31.8628	CAD
Complement C1q tumor necrosis factor-related protein 1	rs79666815	2	G	A	0.096	-0.186	0.042	9.12E-06	0.0060	19.7847	CAD
Complement C1q tumor necrosis factor-related protein 1	rs8094242	18	C	T	0.876	0.175	0.038	2.88E-06	0.0067	22.1878	CAD
Complement C1q tumor necrosis factor-related protein 1	rs115134136	5	C	T	0.012	-0.523	0.116	6.61E-06	0.0067	22.3925	CAD
Complement C1q tumor necrosis factor-related protein 1	rs11658137	17	C	T	0.221	-0.145	0.032	7.94E-06	0.0072	23.9441	CAD
Carbonic anhydrase-related protein 10	rs117399000	17	A	G	0.037	-0.594	0.066	3.02E-19	0.0254	86.0570	CAD
Carbonic anhydrase-related protein 10	rs118127595	20	A	G	0.021	-0.407	0.088	4.07E-06	0.0067	22.3684	CAD
Carbonic anhydrase-related protein 10	rs12152880	5	C	T	0.043	-0.304	0.063	1.45E-06	0.0077	25.5164	CAD
Carbonic anhydrase-related protein 10	rs12740374	1	T	G	0.223	-0.298	0.030	2.09E-23	0.0307	104.5096	CAD
Carbonic anhydrase-related protein 10	rs138223889	5	A	T	0.017	-0.474	0.101	2.95E-06	0.0077	25.5594	CAD
Carbonic anhydrase-related protein 10	rs141660388	12	G	A	0.037	0.364	0.068	1.10E-07	0.0094	31.2764	CAD
Carbonic anhydrase-related protein 10	rs145369908	1	A	G	0.011	-0.626	0.137	5.01E-06	0.0084	27.9140	CAD
Carbonic anhydrase-related protein 10	rs147533560	3	C	T	0.070	0.237	0.050	2.45E-06	0.0072	24.0655	CAD
Carbonic anhydrase-related protein 10	rs148002873	18	T	C	0.103	-0.181	0.041	8.13E-06	0.0061	20.1716	CAD
Carbonic anhydrase-related protein 10	rs17007813	2	G	A	0.219	-0.154	0.032	1.10E-06	0.0081	26.9073	CAD
Carbonic anhydrase-related protein 10	rs182593503	21	A	T	0.163	0.188	0.037	3.24E-07	0.0097	32.2605	CAD
Carbonic anhydrase-related protein 10	rs188726095	12	G	A	0.036	-0.340	0.076	8.51E-06	0.0080	26.5947	CAD
Carbonic anhydrase-related protein 10	rs1950331	14	A	T	0.249	0.139	0.029	1.45E-06	0.0073	24.1130	CAD
Carbonic anhydrase-related protein 10	rs2938139	17	A	G	0.650	-0.148	0.026	1.15E-08	0.0100	33.4070	CAD
Carbonic anhydrase-related protein 10	rs62542335	8	C	T	0.220	0.147	0.030	1.26E-06	0.0074	24.4875	CAD
Carbonic anhydrase-related protein 10	rs75877009	3	T	A	0.025	0.355	0.079	6.92E-06	0.0063	20.7927	CAD
Carbonic anhydrase-related protein 10	rs77715861	15	T	A	0.022	-0.424	0.088	1.32E-06	0.0076	25.4209	CAD
Carbonic anhydrase-related protein 10	rs80348132	12	A	G	0.015	0.509	0.112	5.37E-06	0.0074	24.5704	CAD
Carbonic anhydrase-related protein 10	rs916471	6	A	T	0.396	0.120	0.025	1.86E-06	0.0069	22.8368	CAD
Carbonic anhydrase-related protein 10	rs10080	10	A	G	0.410	-0.128	0.025	3.47E-07	0.0079	26.3973	CAD

Carbonic anhydrase-related protein 10	rs10960792	9	A	G	0.872	0.169	0.037	5.13E-06	0.0064	21.1167	CAD
Four-jointed box protein 1	rs112791615	9	A	C	0.019	-0.445	0.092	1.32E-06	0.0075	24.9905	CAD
Four-jointed box protein 1	rs113148914	21	C	G	0.052	0.281	0.058	1.17E-06	0.0078	26.0780	CAD
Four-jointed box protein 1	rs114064849	1	A	G	0.027	-0.365	0.080	4.68E-06	0.0070	23.3101	CAD
Four-jointed box protein 1	rs11599255	10	A	C	0.160	0.179	0.036	7.59E-07	0.0086	28.5178	CAD
Four-jointed box protein 1	rs116029884	3	T	A	0.052	0.290	0.062	2.57E-06	0.0083	27.7428	CAD
Four-jointed box protein 1	rs117289402	8	G	T	0.023	-0.400	0.086	3.24E-06	0.0072	23.8123	CAD
Four-jointed box protein 1	rs117403438	13	T	C	0.025	-0.391	0.085	4.27E-06	0.0075	24.8200	CAD
Four-jointed box protein 1	rs117462427	8	G	A	0.033	0.329	0.072	4.37E-06	0.0070	23.2190	CAD
Four-jointed box protein 1	rs117531371	22	T	A	0.017	0.511	0.108	2.09E-06	0.0085	28.3102	CAD
Four-jointed box protein 1	rs12637279	3	G	A	0.391	-0.119	0.026	5.37E-06	0.0067	22.3172	CAD
Four-jointed box protein 1	rs12740374	1	T	G	0.223	-0.264	0.030	1.51E-18	0.0240	81.2069	CAD
Four-jointed box protein 1	rs12799443	11	T	C	0.411	0.173	0.027	7.08E-11	0.0144	48.2771	CAD
Four-jointed box protein 1	rs13281883	8	A	G	0.296	-0.121	0.027	8.13E-06	0.0061	20.4056	CAD
Four-jointed box protein 1	rs140928404	5	T	G	0.025	-0.433	0.086	5.37E-07	0.0091	30.3856	CAD
Four-jointed box protein 1	rs147956787	5	C	T	0.015	-0.482	0.108	7.59E-06	0.0069	22.9144	CAD
Four-jointed box protein 1	rs17172023	7	T	C	0.186	-0.151	0.034	8.32E-06	0.0069	22.9884	CAD
Four-jointed box protein 1	rs191967928	5	G	A	0.159	-0.156	0.034	4.79E-06	0.0065	21.6038	CAD
Four-jointed box protein 1	rs2172045	3	T	C	0.909	-0.197	0.043	5.13E-06	0.0064	21.3398	CAD
Four-jointed box protein 1	rs2820950	9	C	T	0.718	-0.130	0.027	2.00E-06	0.0069	22.8814	CAD
Four-jointed box protein 1	rs3184504	12	C	T	0.517	-0.119	0.025	1.26E-06	0.0070	23.3384	CAD
Four-jointed box protein 1	rs34551253	11	T	C	0.040	0.334	0.075	8.13E-06	0.0086	28.6084	CAD
Four-jointed box protein 1	rs6556224	5	G	T	0.714	0.155	0.028	4.27E-08	0.0098	32.5506	CAD
Four-jointed box protein 1	rs66477706	20	C	T	0.031	0.333	0.074	6.61E-06	0.0067	22.3842	CAD
Four-jointed box protein 1	rs73972703	18	T	C	0.041	0.307	0.064	1.51E-06	0.0075	24.8802	CAD
Four-jointed box protein 1	rs74609210	10	C	G	0.145	-0.161	0.035	4.90E-06	0.0064	21.3504	CAD
Four-jointed box protein 1	rs76486638	20	C	T	0.009	0.606	0.131	3.63E-06	0.0067	22.1726	CAD
Four-jointed box protein 1	rs7705197	5	G	A	0.242	-0.132	0.030	9.12E-06	0.0064	21.1894	CAD

Four-jointed box protein 1	rs7924470	11	A	C	0.561	0.131	0.026	3.02E-07	0.0084	27.9927	CAD
Four-jointed box protein 1	rs11074272	15	C	G	0.097	-0.194	0.042	3.63E-06	0.0066	22.0024	CAD
Four-jointed box protein 1	rs11162922	1	G	A	0.061	0.240	0.051	3.09E-06	0.0066	21.8113	CAD
Granulins	rs113269536	14	T	C	0.181	0.155	0.034	3.80E-06	0.0071	23.5053	CAD
Granulins	rs11893324	2	A	C	0.146	-0.161	0.035	3.24E-06	0.0064	21.3766	CAD
Granulins	rs12332763	5	G	A	0.058	0.251	0.055	5.01E-06	0.0068	22.6971	CAD
Granulins	rs13222454	7	A	C	0.026	-0.353	0.079	8.51E-06	0.0063	20.8423	CAD
Granulins	rs1343654	2	C	T	0.320	-0.117	0.026	9.77E-06	0.0059	19.7195	CAD
Granulins	rs139201499	17	A	G	0.053	0.281	0.063	8.51E-06	0.0080	26.4520	CAD
Granulins	rs146522329	3	G	A	0.013	-0.540	0.115	2.57E-06	0.0075	25.0629	CAD
Granulins	rs146750845	9	T	C	0.030	-0.353	0.076	3.02E-06	0.0073	24.3212	CAD
Granulins	rs2011494	2	A	G	0.714	0.132	0.028	2.63E-06	0.0071	23.6007	CAD
Granulins	rs2112256	5	T	G	0.036	-0.340	0.073	2.88E-06	0.0080	26.6048	CAD
Granulins	rs3208406	9	G	A	0.096	0.196	0.043	4.37E-06	0.0066	22.0742	CAD
Granulins	rs4259698	1	A	T	0.823	0.167	0.035	2.34E-06	0.0082	27.1312	CAD
Granulins	rs4747199	10	T	C	0.294	-0.237	0.027	4.27E-18	0.0232	78.4300	CAD
Granulins	rs490748	1	T	G	0.422	-0.119	0.026	3.72E-06	0.0069	23.0680	CAD
Granulins	rs56810541	4	T	A	0.391	0.127	0.026	6.61E-07	0.0077	25.6087	CAD
Granulins	rs5848	17	T	C	0.287	-0.276	0.028	8.13E-23	0.0313	106.4774	CAD
Granulins	rs59124683	2	G	T	0.428	0.116	0.025	4.90E-06	0.0066	21.7699	CAD
Granulins	rs60537218	2	T	C	0.218	-0.138	0.031	9.33E-06	0.0065	21.5399	CAD
Granulins	rs6936335	6	T	C	0.015	0.479	0.108	8.71E-06	0.0069	22.9032	CAD
Granulins	rs7155381	14	C	A	0.964	0.328	0.066	7.76E-07	0.0074	24.7144	CAD
Granulins	rs7259081	19	A	T	0.751	0.159	0.032	4.57E-07	0.0094	31.3480	CAD
Granulins	rs7478859	11	G	C	0.918	-0.209	0.045	4.37E-06	0.0065	21.6977	CAD
Granulins	rs7528419	1	G	A	0.223	-0.823	0.027	1.00E-200	0.2349	1012.8466	CAD
Granulins	rs7534498	1	A	G	0.189	-0.141	0.032	7.76E-06	0.0061	20.1908	CAD
Granulins	rs78308677	20	G	C	0.029	-0.432	0.082	1.17E-07	0.0104	34.7037	CAD

Granulins	rs78483586	7	A	G	0.035	-0.307	0.069	9.55E-06	0.0064	21.1180	CAD
Granulins	rs7907682	10	A	G	0.530	-0.113	0.025	4.27E-06	0.0064	21.1222	CAD
Granulins	rs8056814	16	A	G	0.090	-0.276	0.043	1.70E-10	0.0125	41.7290	CAD
Granulins	rs10759022	9	T	C	0.627	-0.114	0.026	7.94E-06	0.0061	20.3168	CAD
Granulins	rs10937185	3	A	G	0.142	0.171	0.037	3.72E-06	0.0071	23.6205	CAD
Hemojuvelin	rs10972581	9	T	C	0.401	-0.150	0.025	2.34E-09	0.0108	35.8941	CAD
Hemojuvelin	rs11123178	2	A	C	0.895	-0.222	0.043	3.16E-07	0.0092	30.7972	CAD
Hemojuvelin	rs112721997	20	A	G	0.044	-0.333	0.068	7.76E-07	0.0094	31.3660	CAD
Hemojuvelin	rs115486808	2	C	T	0.033	0.325	0.073	8.32E-06	0.0068	22.4620	CAD
Hemojuvelin	rs11794723	9	G	A	0.120	0.172	0.038	7.08E-06	0.0062	20.6889	CAD
Hemojuvelin	rs118106470	14	T	C	0.019	-0.426	0.092	3.98E-06	0.0067	22.2469	CAD
Hemojuvelin	rs11976611	7	A	G	0.210	-0.140	0.030	3.89E-06	0.0065	21.5356	CAD
Hemojuvelin	rs12285709	11	A	G	0.251	0.134	0.029	3.98E-06	0.0067	22.3823	CAD
Hemojuvelin	rs12529488	6	T	C	0.072	0.244	0.048	2.95E-07	0.0080	26.4946	CAD
Hemojuvelin	rs12740374	1	T	G	0.223	-0.231	0.030	1.41E-14	0.0185	62.2209	CAD
Hemojuvelin	rs151313431	14	G	A	0.016	0.512	0.104	8.51E-07	0.0084	27.8104	CAD
Hemojuvelin	rs16839039	1	G	T	0.015	-0.547	0.117	2.69E-06	0.0086	28.6220	CAD
Hemojuvelin	rs16851014	4	A	G	0.226	-0.132	0.030	8.71E-06	0.0060	20.0761	CAD
Hemojuvelin	rs17003597	21	G	A	0.108	-0.201	0.040	6.31E-07	0.0078	25.7972	CAD
Hemojuvelin	rs2435979	17	T	G	0.178	0.147	0.033	9.12E-06	0.0063	20.9493	CAD
Hemojuvelin	rs2663186	11	T	C	0.701	-0.126	0.027	4.68E-06	0.0066	21.9776	CAD
Hemojuvelin	rs34929745	10	T	C	0.045	0.276	0.060	4.47E-06	0.0066	21.7551	CAD
Hemojuvelin	rs4683441	3	G	A	0.493	0.119	0.025	1.95E-06	0.0071	23.6795	CAD
Hemojuvelin	rs4778090	15	G	A	0.556	-0.148	0.025	5.89E-09	0.0107	35.8265	CAD
Hemojuvelin	rs55728583	4	C	T	0.041	0.281	0.063	7.59E-06	0.0062	20.4286	CAD
Hemojuvelin	rs5752604	22	G	A	0.009	0.604	0.131	3.89E-06	0.0065	21.4869	CAD
Hemojuvelin	rs59319674	14	C	G	0.275	-0.126	0.028	4.90E-06	0.0063	20.8740	CAD
Hemojuvelin	rs75466623	1	A	G	0.016	-0.605	0.103	4.90E-09	0.0115	38.4160	CAD

Hemojuvelin	rs75485896	5	T	C	0.031	0.346	0.071	1.17E-06	0.0073	24.1632	CAD
Hemojuvelin	rs79098790	15	G	A	0.010	-0.570	0.126	5.75E-06	0.0066	21.8320	CAD
Hemojuvelin	rs9712146	2	T	C	0.019	0.470	0.105	7.76E-06	0.0080	26.7161	CAD
Hemojuvelin	rs10893498	11	A	G	0.138	-0.182	0.036	3.55E-07	0.0079	26.3028	CAD
Hemojuvelin	rs11711698	3	G	A	0.354	-0.116	0.026	7.76E-06	0.0062	20.5691	CAD
Hemojuvelin	rs11726050	4	G	C	0.048	0.257	0.058	8.71E-06	0.0060	20.0157	CAD
Neogenin	rs117323386	13	G	A	0.024	-0.417	0.086	1.17E-06	0.0081	26.9121	CAD
Neogenin	rs117902003	17	T	C	0.017	0.477	0.095	4.47E-07	0.0078	25.7684	CAD
Neogenin	rs12903656	15	C	G	0.101	0.286	0.042	5.50E-12	0.0148	49.6664	CAD
Neogenin	rs13038050	20	A	G	0.090	-0.212	0.044	1.58E-06	0.0073	24.2740	CAD
Neogenin	rs138835775	19	T	C	0.029	-0.378	0.082	3.89E-06	0.0082	27.1283	CAD
Neogenin	rs141158299	3	T	A	0.019	0.477	0.094	3.98E-07	0.0084	28.0389	CAD
Neogenin	rs146441533	11	T	C	0.014	-0.535	0.117	5.13E-06	0.0079	26.2295	CAD
Neogenin	rs17564137	5	C	T	0.062	-0.260	0.052	4.68E-07	0.0078	26.0212	CAD
Neogenin	rs34436714	19	A	C	0.213	-0.140	0.030	3.47E-06	0.0065	21.7230	CAD
Neogenin	rs34784126	4	T	C	0.329	-0.120	0.027	7.76E-06	0.0063	21.0302	CAD
Neogenin	rs3756555	5	A	G	0.329	-0.119	0.026	6.31E-06	0.0062	20.7478	CAD
Neogenin	rs4274662	21	A	C	0.190	-0.154	0.032	1.74E-06	0.0073	24.4238	CAD
Neogenin	rs56033109	16	C	T	0.093	-0.195	0.043	4.47E-06	0.0064	21.3737	CAD
Neogenin	rs629301	1	T	G	0.776	-0.233	0.030	7.08E-15	0.0189	63.5414	CAD
Neogenin	rs73148326	3	C	T	0.100	-0.199	0.042	1.86E-06	0.0071	23.6733	CAD
Neogenin	rs73355007	7	T	C	0.011	0.556	0.118	2.24E-06	0.0070	23.0963	CAD
Neogenin	rs73831204	4	C	A	0.012	0.507	0.113	7.08E-06	0.0062	20.5242	CAD
Neogenin	rs74580127	5	T	C	0.180	-0.152	0.033	5.13E-06	0.0068	22.6992	CAD
Neogenin	rs7462091	8	C	T	0.915	0.216	0.046	2.69E-06	0.0072	24.0787	CAD
Neogenin	rs7600467	2	T	A	0.938	-0.230	0.051	7.94E-06	0.0062	20.4650	CAD
Neogenin	rs78299458	10	G	A	0.018	0.448	0.100	7.41E-06	0.0069	23.0125	CAD
Neogenin	rs79671946	5	T	C	0.030	0.345	0.074	3.47E-06	0.0070	23.3219	CAD

Neogenin	rs9550431	13	A	C	0.483	-0.117	0.025	2.51E-06	0.0068	22.5147	CAD
Neogenin	rs9951183	18	A	G	0.398	0.119	0.025	1.86E-06	0.0068	22.4611	CAD
Neogenin	rs1044433	17	C	T	0.703	-0.130	0.028	3.02E-06	0.0070	23.3776	CAD
Neogenin	rs115829880	6	C	A	0.040	0.282	0.064	9.12E-06	0.0062	20.4920	CAD
Neogenin	rs12614673	2	A	T	0.076	-0.203	0.046	9.33E-06	0.0058	19.1951	CAD
Group XIIB secretory phospholipase A2-like protein	rs12637951	3	T	C	0.647	0.119	0.026	5.89E-06	0.0064	21.3340	CAD
Group XIIB secretory phospholipase A2-like protein	rs138488830	13	C	A	0.017	-0.446	0.099	6.92E-06	0.0066	22.0634	CAD
Group XIIB secretory phospholipase A2-like protein	rs147230016	1	C	T	0.024	-0.387	0.083	2.75E-06	0.0070	23.3287	CAD
Group XIIB secretory phospholipase A2-like protein	rs1799561	5	G	A	0.285	-0.129	0.028	5.62E-06	0.0067	22.3958	CAD
Group XIIB secretory phospholipase A2-like protein	rs182744657	8	T	C	0.052	0.288	0.060	1.38E-06	0.0082	27.4346	CAD
Group XIIB secretory phospholipase A2-like protein	rs36165532	2	G	A	0.364	0.118	0.027	9.55E-06	0.0064	21.4064	CAD
Group XIIB secretory phospholipase A2-like protein	rs4435708	4	G	A	0.351	-0.115	0.026	7.08E-06	0.0060	20.0258	CAD
Group XIIB secretory phospholipase A2-like protein	rs4632248	19	T	G	0.214	-0.141	0.030	2.57E-06	0.0067	22.1789	CAD
Group XIIB secretory phospholipase A2-like protein	rs4807487	19	T	C	0.061	0.232	0.053	9.77E-06	0.0062	20.5202	CAD
Group XIIB secretory phospholipase A2-like protein	rs4970836	1	A	G	0.771	0.422	0.029	3.80E-48	0.0631	222.1382	CAD
Group XIIB secretory phospholipase A2-like protein	rs544904792	16	A	C	0.011	0.591	0.132	7.59E-06	0.0076	25.2032	CAD
Group XIIB secretory phospholipase A2-like protein	rs61373967	6	C	T	0.010	-0.675	0.137	7.94E-07	0.0092	30.6116	CAD
Group XIIB secretory phospholipase A2-like protein	rs6545815	2	C	T	0.225	-0.157	0.033	1.55E-06	0.0086	28.5907	CAD
Group XIIB secretory phospholipase A2-like protein	rs6600376	1	C	A	0.301	-0.128	0.027	2.19E-06	0.0068	22.7415	CAD
Group XIIB secretory phospholipase A2-like protein	rs662	7	C	T	0.283	-0.155	0.027	1.74E-08	0.0097	32.3055	CAD
Group XIIB secretory phospholipase A2-like protein	rs7163501	15	C	G	0.019	0.458	0.093	8.32E-07	0.0079	26.1279	CAD
Group XIIB secretory phospholipase A2-like protein	rs73953232	18	C	A	0.044	-0.312	0.062	5.37E-07	0.0083	27.4897	CAD
Group XIIB secretory phospholipase A2-like protein	rs74063777	14	A	G	0.118	-0.190	0.039	7.76E-07	0.0075	25.0822	CAD
Group XIIB secretory phospholipase A2-like protein	rs74802900	1	T	C	0.045	-0.296	0.060	8.51E-07	0.0075	24.7951	CAD
Group XIIB secretory phospholipase A2-like protein	rs7779835	7	C	T	0.024	0.379	0.082	3.80E-06	0.0067	22.2928	CAD
Group XIIB secretory phospholipase A2-like protein	rs78057821	17	A	T	0.110	0.216	0.046	2.88E-06	0.0091	30.4623	CAD
Group XIIB secretory phospholipase A2-like protein	rs7808802	7	G	A	0.492	-0.115	0.025	3.39E-06	0.0066	22.0318	CAD
Group XIIB secretory phospholipase A2-like protein	rs78286207	2	C	G	0.053	-0.251	0.057	9.55E-06	0.0064	21.1428	CAD

Group XIIB secretory phospholipase A2-like protein	rs78800990	8	A	G	0.049	0.261	0.057	5.37E-06	0.0063	21.0745	CAD
Group XIIB secretory phospholipase A2-like protein	rs9512488	13	T	C	0.157	-0.150	0.034	8.71E-06	0.0060	19.8120	CAD
Group XIIB secretory phospholipase A2-like protein	rs977324	3	A	T	0.363	-0.123	0.026	2.34E-06	0.0070	23.1356	CAD
Group XIIB secretory phospholipase A2-like protein	rs10276485	7	A	G	0.050	0.296	0.058	2.75E-07	0.0083	27.7102	CAD
Group XIIB secretory phospholipase A2-like protein	rs10511312	3	C	G	0.035	0.335	0.069	1.23E-06	0.0076	25.1768	CAD
Group XIIB secretory phospholipase A2-like protein	rs1065853	19	T	G	0.078	-0.274	0.046	3.02E-09	0.0108	35.9716	CAD
Sodium-coupled monocarboxylate transporter 1	rs112837281	7	C	T	0.032	0.330	0.073	5.62E-06	0.0067	22.3125	CAD
Sodium-coupled monocarboxylate transporter 1	rs114121871	5	A	G	0.021	0.405	0.092	9.77E-06	0.0068	22.7160	CAD
Sodium-coupled monocarboxylate transporter 1	rs115696439	1	A	G	0.051	-0.266	0.060	8.91E-06	0.0068	22.6065	CAD
Sodium-coupled monocarboxylate transporter 1	rs116116251	4	G	A	0.018	-0.431	0.093	3.39E-06	0.0067	22.2673	CAD
Sodium-coupled monocarboxylate transporter 1	rs117336643	19	T	C	0.026	0.421	0.091	3.72E-06	0.0089	29.6798	CAD
Sodium-coupled monocarboxylate transporter 1	rs117484071	16	T	C	0.031	-0.342	0.076	6.92E-06	0.0069	23.0522	CAD
Sodium-coupled monocarboxylate transporter 1	rs12145173	1	T	A	0.384	0.121	0.027	8.91E-06	0.0069	22.8456	CAD
Sodium-coupled monocarboxylate transporter 1	rs12914819	15	C	A	0.057	-0.261	0.054	1.29E-06	0.0073	24.2792	CAD
Sodium-coupled monocarboxylate transporter 1	rs137880059	11	T	C	0.122	0.179	0.039	5.01E-06	0.0068	22.6154	CAD
Sodium-coupled monocarboxylate transporter 1	rs144225396	9	T	C	0.008	-0.736	0.158	3.02E-06	0.0081	26.8642	CAD
Sodium-coupled monocarboxylate transporter 1	rs149650692	8	A	T	0.011	0.526	0.118	7.94E-06	0.0062	20.4647	CAD
Sodium-coupled monocarboxylate transporter 1	rs150244229	8	C	G	0.009	0.679	0.150	5.50E-06	0.0086	28.7179	CAD
Sodium-coupled monocarboxylate transporter 1	rs17715292	18	C	A	0.108	0.178	0.040	8.91E-06	0.0061	20.2468	CAD
Sodium-coupled monocarboxylate transporter 1	rs1803274	3	T	C	0.203	0.191	0.031	6.61E-10	0.0118	39.4744	CAD
Sodium-coupled monocarboxylate transporter 1	rs189563861	6	A	C	0.020	-0.433	0.093	3.24E-06	0.0075	24.9567	CAD
Sodium-coupled monocarboxylate transporter 1	rs201994583	16	A	T	0.094	-0.201	0.045	6.31E-06	0.0069	23.0169	CAD
Sodium-coupled monocarboxylate transporter 1	rs2513973	8	T	C	0.072	0.226	0.048	2.82E-06	0.0068	22.7115	CAD
Sodium-coupled monocarboxylate transporter 1	rs2742981	6	T	A	0.732	0.128	0.028	4.47E-06	0.0064	21.2796	CAD
Sodium-coupled monocarboxylate transporter 1	rs2774043	14	T	C	0.870	-0.174	0.037	3.24E-06	0.0068	22.6828	CAD
Sodium-coupled monocarboxylate transporter 1	rs2802655	1	T	C	0.866	-0.165	0.036	5.75E-06	0.0063	21.0215	CAD
Sodium-coupled monocarboxylate transporter 1	rs3749107	2	A	G	0.077	-0.203	0.046	9.12E-06	0.0059	19.4449	CAD
Sodium-coupled monocarboxylate transporter 1	rs3972765	20	A	G	0.400	0.129	0.027	2.40E-06	0.0080	26.4484	CAD

Sodium-coupled monocarboxylate transporter 1	rs4665710	2	C	A	0.794	-0.279	0.030	2.04E-20	0.0256	86.5692	CAD
Sodium-coupled monocarboxylate transporter 1	rs4870498	6	T	C	0.083	-0.216	0.048	6.31E-06	0.0071	23.6935	CAD
Sodium-coupled monocarboxylate transporter 1	rs56120514	11	T	C	0.482	-0.115	0.025	2.88E-06	0.0066	22.0092	CAD
Sodium-coupled monocarboxylate transporter 1	rs602633	1	G	T	0.780	0.164	0.030	5.62E-08	0.0093	30.8525	CAD
Sodium-coupled monocarboxylate transporter 1	rs62276380	3	G	A	0.306	0.121	0.027	7.24E-06	0.0062	20.5232	CAD
Sodium-coupled monocarboxylate transporter 1	rs62382443	5	A	C	0.111	0.197	0.043	4.90E-06	0.0076	25.3909	CAD
Sodium-coupled monocarboxylate transporter 1	rs7209843	17	G	A	0.418	0.117	0.025	3.80E-06	0.0066	22.0104	CAD
Sodium-coupled monocarboxylate transporter 1	rs72632927	8	T	A	0.124	-0.182	0.039	3.63E-06	0.0072	23.7762	CAD
Sodium-coupled monocarboxylate transporter 1	rs7298155	12	C	G	0.041	-0.294	0.066	7.41E-06	0.0068	22.7357	CAD
Sodium-coupled monocarboxylate transporter 1	rs75224365	14	C	A	0.028	-0.366	0.079	4.07E-06	0.0072	24.0789	CAD
Sodium-coupled monocarboxylate transporter 1	rs76141277	15	G	A	0.112	0.197	0.043	5.25E-06	0.0077	25.5400	CAD
Sodium-coupled monocarboxylate transporter 1	rs7650596	3	A	C	0.780	-0.140	0.031	5.62E-06	0.0067	22.2235	CAD
Sodium-coupled monocarboxylate transporter 1	rs7980521	12	A	G	0.625	-0.123	0.027	6.03E-06	0.0071	23.5266	CAD

Chr denotes chromosome; EAF, effective allele frequency; SE, standard error; $R^2 = 2 \times MAF \times (1-MAF) \times beta^2$; $F = R^2 \times (N-2)/(1-R^2)$.

Table S21. Causal associations of *PSRC1*-associated circulating proteins with CAD.

Outcome	Proteins	Methods	NSNPs	OR (95%CI)	*pval*	*FDR*
CAD	Apolipoprotein B	MR Egger	35	1.15 (1.00, 1.33)	0.060	0.135
CAD	Apolipoprotein B	Weighted median	35	0.98 (0.96, 1.00)	0.091	0.273
CAD	Apolipoprotein B	IVW	35	1.04 (0.98, 1.10)	0.224	0.224
CAD	Carbonic anhydrase-related protein 10	MR Egger	21	1.09 (0.95, 1.25)	0.218	0.327
CAD	Carbonic anhydrase-related protein 10	Weighted median	21	0.99 (0.96, 1.02)	0.434	0.523
CAD	Carbonic anhydrase-related protein 10	IVW	21	1.05 (0.99, 1.12)	0.132	0.172
CAD	Complement C1q tumor necrosis factor-related protein 1	MR Egger	25	1.19 (1.13, 1.25)	2.21E-07	9.95E-07
CAD	Complement C1q tumor necrosis factor-related protein 1	Weighted median	25	1.04 (1.01, 1.07)	0.020	0.090
CAD	Complement C1q tumor necrosis factor-related protein 1	IVW	25	1.08 (1.04, 1.11)	3.41E-05	1.53E-04
CAD	Four-jointed box protein 1	MR Egger	30	1.05 (0.92, 1.20)	0.487	0.612
CAD	Four-jointed box protein 1	Weighted median	30	0.99 (0.97, 1.02)	0.483	0.523
CAD	Four-jointed box protein 1	IVW	30	1.05 (0.99, 1.10)	0.100	0.172
CAD	Granulins	MR Egger	30	1.12 (1.09, 1.16)	1.23E-07	9.95E-07
CAD	Granulins	Weighted median	30	1.06 (1.03, 1.10)	6.90E-05	0.0006
CAD	Granulins	IVW	30	1.06 (1.04, 1.09)	5.35E-07	4.82E-06
CAD	Group XIIB secretory phospholipase A2-like protein	MR Egger	29	1.16 (1.08, 1.24)	3.04E-04	0.0009
CAD	Group XIIB secretory phospholipase A2-like protein	Weighted median	29	1.01 (0.98, 1.03)	0.512	0.523
CAD	Group XIIB secretory phospholipase A2-like protein	IVW	29	1.05 (1.01, 1.09)	0.009	0.028
CAD	Hemojuvelin	MR Egger	28	1.08 (0.96, 1.21)	0.204	0.327
CAD	Hemojuvelin	Weighted median	28	1.01 (0.99, 1.04)	0.224	0.403
CAD	Hemojuvelin	IVW	28	1.03 (0.99, 1.09)	0.168	0.189
CAD	Neogenin	MR Egger	27	0.97 (0.86, 1.09)	0.643	0.643
CAD	Neogenin	Weighted median	27	0.98 (0.96, 1.01)	0.224	0.403
CAD	Neogenin	IVW	27	0.95 (0.90, 0.99)	0.029	0.064
CAD	Sodium-coupled monocarboxylate transporter 1	MR Egger	38	1.04 (0.92, 1.17)	0.544	0.612
CAD	Sodium-coupled monocarboxylate transporter 1	Weighted median	38	1.01 (0.98, 1.03)	0.523	0.523
CAD	Sodium-coupled monocarboxylate transporter 1	IVW	38	1.04 (0.99, 1.08)	0.134	0.172

IVW denotes inverse variance weighted method; *FDR* < 0.05 was considered as nominally significant.

Table S22. Colocalization between *PSRC1*-associated circulating proteins and CAD.

Outcome	Proteins	SNP	Chr	BP	NSNPs	PP.H3	PP.H4
CAD	Complement C1q tumor necrosis factor-related protein 1	rs646776	1	109818530	5651	0.0486	0.9514
CAD	Complement C1q tumor necrosis factor-related protein 1	rs5848	17	42430244	5286	0.9183	0.0006
CAD	Complement C1q tumor necrosis factor-related protein 1	rs10762481	10	73538164	6500	0.7315	0.0027
CAD	Complement C1q tumor necrosis factor-related protein 1	rs112635299	14	94838142	7923	0.001	0.999
CAD	Complement C1q tumor necrosis factor-related protein 1	rs72802395	16	75286484	8113	0.9985	0.0001
CAD	Complement C1q tumor necrosis factor-related protein 1	rs7259081	19	54339243	9979	0.1577	0.0088
CAD	Complement C1q tumor necrosis factor-related protein 1	rs78308677	20	56722048	7280	0.5985	0.0104
CAD	Complement C1q tumor necrosis factor-related protein 1	rs490748	1	201643520	6539	0.8573	0.0071
CAD	Complement C1q tumor necrosis factor-related protein 1	rs138242461	6	39090655	7241	0.3894	0.1335
CAD	Complement C1q tumor necrosis factor-related protein 1	rs139242303	14	94668249	7738	0.001	0.999
CAD	Complement C1q tumor necrosis factor-related protein 1	rs8094242	18	62370987	7365	0.0648	0.0048
CAD	Complement C1q tumor necrosis factor-related protein 1	rs112453666	15	96773014	6418	0.3893	0.0219
CAD	Complement C1q tumor necrosis factor-related protein 1	rs72644662	1	2468726	7102	0.4602	0.0188
CAD	Complement C1q tumor necrosis factor-related protein 1	rs139201499	17	49718126	6130	0.375	0.0129
CAD	Complement C1q tumor necrosis factor-related protein 1	rs2061596	11	37187913	7587	0.1174	0.0117
CAD	Complement C1q tumor necrosis factor-related protein 1	rs112305118	13	101198594	7030	0.0419	0.0056
CAD	Complement C1q tumor necrosis factor-related protein 1	rs17047444	3	68336960	7195	0.0536	0.0061
CAD	Complement C1q tumor necrosis factor-related protein 1	rs62653756	10	14176519	8787	0.0915	0.0058
CAD	Complement C1q tumor necrosis factor-related protein 1	rs2642704	5	132965342	6048	0.1121	0.0072
CAD	Complement C1q tumor necrosis factor-related protein 1	rs140128652	6	155112555	7664	0.0684	0.007
CAD	Complement C1q tumor necrosis factor-related protein 1	rs111219454	12	18250706	7877	0.5662	0.0189
CAD	Complement C1q tumor necrosis factor-related protein 1	rs116451478	1	226767251	6874	0.3145	0.0576
CAD	Complement C1q tumor necrosis factor-related protein 1	rs113634207	2	83746895	6332	0.1027	0.0045
CAD	Complement C1q tumor necrosis factor-related protein 1	rs79666815	2	228488279	7808	0.2288	0.006
CAD	Complement C1q tumor necrosis factor-related protein 1	rs7535434	1	41857828	6410	0.1248	0.0067
CAD	Granulins	rs7528419	1	109817192	5643	0.0103	0.9897
CAD	Granulins	rs5848	17	42430244	5286	0.9183	0.0006

CAD	Granulins	rs4747199	10	73569318	6343	0.7318	0.0029
CAD	Granulins	rs8056814	16	75252327	8074	0.9999	0.0001
CAD	Granulins	rs78308677	20	56722048	7280	0.753	0.0062
CAD	Granulins	rs7259081	19	54339243	9979	0.1425	0.0088
CAD	Granulins	rs56810541	4	187200550	8722	0.8852	0.0026
CAD	Granulins	rs7155381	14	61527867	5463	0.1072	0.0076
CAD	Granulins	rs4259698	1	248819871	4699	0.0557	0.0051
CAD	Granulins	rs146522329	3	183919412	6559	0.0517	0.0064
CAD	Granulins	rs2011494	2	43158208	7474	0.802	0.0052
CAD	Granulins	rs2112256	5	121587022	7622	0.5569	0.0125
CAD	Granulins	rs146750845	9	29396931	8398	0.0806	0.0081
CAD	Granulins	rs11162922	1	80572058	7748	0.1165	0.0061
CAD	Granulins	rs11893324	2	53225716	9039	0.3373	0.0049
CAD	Granulins	rs11074272	15	86024431	8085	0.8132	0.0162
CAD	Granulins	rs490748	1	201643520	6539	0.6208	0.0173
CAD	Granulins	rs113269536	14	34947987	7610	0.1711	0.006
CAD	Granulins	rs7907682	10	105371052	5520	0.7154	0.013
CAD	Granulins	rs3208406	9	37780831	6572	0.3449	0.0149
CAD	Granulins	rs7478859	11	37170885	7566	0.1093	0.0109
CAD	Granulins	rs59124683	2	240325579	8885	0.1134	0.0039
CAD	Granulins	rs12332763	5	125347288	8038	0.0552	0.0062
CAD	Granulins	rs7534498	1	109674087	5576	0.0103	0.9897
CAD	Granulins	rs13222454	7	67603787	10011	0.092	0.0082
CAD	Granulins	rs139201499	17	49718126	6130	0.3078	0.0459
CAD	Granulins	rs6936335	6	159309170	8245	0.4437	0.0744
CAD	Granulins	rs60537218	2	16189121	6496	0.0509	0.0052
CAD	Granulins	rs78483586	7	116402346	4816	0.3871	0.0282
CAD	Granulins	rs1343654	2	11005485	8523	0.1084	0.0095
CAD	Group XIIB secretory phospholipase A2-like protein	rs4970836	1	109821797	5676	0.0044	0.9956

CAD	Group XIIB secretory phospholipase A2-like protein	rs662	7	94937446	5438	0.1125	0.0069
CAD	Group XIIB secretory phospholipase A2-like protein	rs73953232	18	45025975	6928	0.1706	0.006
CAD	Group XIIB secretory phospholipase A2-like protein	rs74063777	14	78550328	7373	0.1812	0.0073
CAD	Group XIIB secretory phospholipase A2-like protein	rs61373967	6	138586141	6883	0.0453	0.0056
CAD	Group XIIB secretory phospholipase A2-like protein	rs7163501	15	90600851	6337	0.6534	0.007
CAD	Group XIIB secretory phospholipase A2-like protein	rs74802900	1	220892181	6835	0.1001	0.0056
CAD	Group XIIB secretory phospholipase A2-like protein	rs182744657	8	145703350	3573	0.1001	0.0078
CAD	Group XIIB secretory phospholipase A2-like protein	rs6545815	2	21579163	5829	0.6357	0.0072
CAD	Group XIIB secretory phospholipase A2-like protein	rs6600376	1	38750228	6133	0.7999	0.01
CAD	Group XIIB secretory phospholipase A2-like protein	rs977324	3	65291961	7526	0.4294	0.0078
CAD	Group XIIB secretory phospholipase A2-like protein	rs4632248	19	54324995	9990	0.138	0.006
CAD	Group XIIB secretory phospholipase A2-like protein	rs147230016	1	31390011	6774	0.058	0.0057
CAD	Group XIIB secretory phospholipase A2-like protein	rs78057821	17	7825671	6051	0.1644	0.021
CAD	Group XIIB secretory phospholipase A2-like protein	rs1044433	17	72947061	6729	0.6804	0.0094
CAD	Group XIIB secretory phospholipase A2-like protein	rs7808802	7	49471741	7724	0.1414	0.0054
CAD	Group XIIB secretory phospholipase A2-like protein	rs7779835	7	79686983	6225	0.3766	0.0062
CAD	Group XIIB secretory phospholipase A2-like protein	rs78800990	8	25551709	7690	0.4518	0.0122
CAD	Group XIIB secretory phospholipase A2-like protein	rs1799561	5	165858382	6113	0.0493	0.0057
CAD	Group XIIB secretory phospholipase A2-like protein	rs12637951	3	126272914	8537	0.4889	0.0107
CAD	Group XIIB secretory phospholipase A2-like protein	rs138488830	13	54651042	6509	0.07	0.0083
CAD	Group XIIB secretory phospholipase A2-like protein	rs4435708	4	88199059	6683	0.0768	0.0061
CAD	Group XIIB secretory phospholipase A2-like protein	rs544904792	16	89325238	8934	0.1474	0.0147
CAD	Group XIIB secretory phospholipase A2-like protein	rs9512488	13	27583770	8313	0.3723	0.009
CAD	Group XIIB secretory phospholipase A2-like protein	rs115829880	6	121713976	7499	0.1071	0.0126
CAD	Group XIIB secretory phospholipase A2-like protein	rs12614673	2	195959105	5627	0.0543	0.0052
CAD	Group XIIB secretory phospholipase A2-like protein	rs78286207	2	192620263	4533	0.0631	0.007
CAD	Group XIIB secretory phospholipase A2-like protein	rs36165532	2	239782337	8378	0.0701	0.0047
CAD	Group XIIB secretory phospholipase A2-like protein	rs4807487	19	3554702	7871	0.0802	0.0078
CAD	Neogenin	rs629301	1	109818306	5648	0.0056	0.9944

CAD	Neogenin		rs12903656	15	73326961	5037	0.8843	0.0242
CAD	Neogenin		rs10893498	11	126241696	7478	0.9669	0.0089
CAD	Neogenin		rs141158299	3	12426878	7275	0.4353	0.0189
CAD	Neogenin		rs117902003	17	59044509	3318	0.7545	0.0079
CAD	Neogenin		rs17564137	5	88378600	4040	0.7549	0.0066
CAD	Neogenin		rs117323386	13	111288887	8922	0.6007	0.0115
CAD	Neogenin		rs13038050	20	42133747	6830	0.1978	0.0067
CAD	Neogenin		rs4274662	21	37827694	7482	0.1092	0.0041
CAD	Neogenin		rs73148326	3	24309095	7745	0.1082	0.0049
CAD	Neogenin		rs9951183	18	25231495	6032	0.1344	0.0143
CAD	Neogenin		rs73355007	7	57259939	6153	0.042	0.0074
CAD	Neogenin		rs9550431	13	29569052	7076	0.8569	0.0028
CAD	Neogenin		rs7462091	8	140141910	7515	0.0635	0.011
CAD	Neogenin		rs79671946	5	2624166	10725	0.107	0.0108
CAD	Neogenin		rs34436714	19	54327313	9996	0.1322	0.0061
CAD	Neogenin		rs138835775	19	56907156	8740	0.0782	0.0076
CAD	Neogenin		rs56033109	16	59207631	7698	0.1019	0.0055
CAD	Neogenin		rs74580127	5	73220830	6538	0.074	0.0043
CAD	Neogenin		rs146441533	11	92485563	6555	0.0617	0.0099
CAD	Neogenin		rs3756555	5	115243936	8724	0.0983	0.0044
CAD	Neogenin		rs73831204	4	81847776	6702	0.4507	0.0095
CAD	Neogenin		rs78299458	10	100050676	5965	0.2947	0.0117
CAD	Neogenin		rs11711698	3	121917387	6383	0.2646	0.1325
CAD	Neogenin		rs34784126	4	59973705	8418	0.0965	0.0034
CAD	Neogenin		rs7600467	2	106543154	8105	0.0835	0.0045
CAD	Neogenin		rs11726050	4	127055132	7619	0.2675	0.028

Chr denotes chromosome; BP, base pair position; PP.H3 denotes association with CAD or IS risk as well as (apo)lipoprotein measurements, but at distinct causal variants; PP.H4 denotes association with both traits, with a shared causal variant. A PP.H4 greater than 0.8 indicates significant colocalization.

Table S23. The 131 (apo)lipoprotein measurements examined in this study.

(apo)lipoprotein profile	Abbreviation	Cluster	Units	Group	Sub.Group	UKB. Field.ID	QC.Flag. Field.ID
Total Concentration of Lipoprotein Particles	Total_P	A	mmol/L	Lipoprotein particle concentrations		23427	23727
Total Concentration of VLDL Particles	VLDL_P	A	mmol/L	Lipoprotein particle concentrations		23428	23728
Total Concentration of Very Small VLDL Particles	XS_VLDL_P	A	mmol/L	Lipoprotein subclasses	Very small VLDL (average diameter 31.3 nm)	23516	23816
Total Concentration of Small VLDL Particles	S_VLDL_P	A	mmol/L	Lipoprotein subclasses	Small VLDL (average diameter 36.8 nm)	23509	23809
Total Concentration of Medium VLDL Particles	M_VLDL_P	A	mmol/L	Lipoprotein subclasses	Medium VLDL (average diameter 44.5 nm)	23502	23802
Total Concentration of Large VLDL Particles	L_VLDL_P	A	mmol/L	Lipoprotein subclasses	Large VLDL (average diameter 53.6 nm)	23495	23795
Total Concentration of Very Large VLDL Particles	XL_VLDL_P	A	mmol/L	Lipoprotein subclasses	Very large VLDL (average diameter 64 nm)	23488	23788
Total Concentration of Chylomicrons and Extremely Large VLDL Particles	XXL_VLDL_P	A	mmol/L	Lipoprotein subclasses	Chylomicrons and extremely large VLDL (particle diameters from 75 nm upwards)	23481	23781
Total Concentration of IDL Particles	IDL_P	A	mmol/L	Lipoprotein subclasses	IDL (average diameter 28.6 nm)	23523	23823
Total Concentration of LDL Particles	LDL_P	A	mmol/L	Lipoprotein particle concentrations		23429	23729
Total Concentration of Small LDL Particles	S_LDL_P	A	mmol/L	Lipoprotein subclasses	Small LDL (average diameter 18.7 nm)	23544	23844
Total Concentration of Medium	M_LDL_P	A	mmol/L	Lipoprotein subclasses	Medium LDL (average diameter 23	23537	23837

LDL Particles					nm)		
Total Concentration of Large LDL Particles	L_LDL_P	A	mmol/L	Lipoprotein subclasses	Large LDL (average diameter 25.5 nm)	23530	23830
Total Concentration of HDL Particles	HDL_P	A	mmol/L	Lipoprotein particle concentrations		23430	23730
Total Concentration of Small HDL Particles	S_HDL_P	A	mmol/L	Lipoprotein subclasses	Small HDL (average diameter 8.7 nm)	23572	23872
Total Concentration of Medium HDL Particles	M_HDL_P	A	mmol/L	Lipoprotein subclasses	Medium HDL (average diameter 10.9 nm)	23565	23865
Total Concentration of Large HDL Particles	L_HDL_P	A	mmol/L	Lipoprotein subclasses	Large HDL (average diameter 12.1 nm)	23558	23858
Total Concentration of Very Large HDL Particles	XL_HDL_P	A	mmol/L	Lipoprotein subclasses	Very large HDL (average diameter 14.3 nm)	23551	23851
Total Lipids in Lipoprotein Particles	Total_L	B	mmol/L	Total lipids		23423	23723
Total Lipids in VLDL	VLDL_L	B	mmol/L	Total lipids		23424	23724
Total Lipids in Very Small VLDL	XS_VLDL_L	B	mmol/L	Lipoprotein subclasses	Very small VLDL (average diameter 31.3 nm)	23517	23817
Total Lipids in Small VLDL	S_VLDL_L	B	mmol/L	Lipoprotein subclasses	Small VLDL (average diameter 36.8 nm)	23510	23810
Total Lipids in Medium VLDL	M_VLDL_L	B	mmol/L	Lipoprotein subclasses	Medium VLDL (average diameter 44.5 nm)	23503	23803
Total Lipids in Large VLDL	L_VLDL_L	B	mmol/L	Lipoprotein subclasses	Large VLDL (average diameter 53.6 nm)	23496	23796
Total Lipids in Very Large VLDL	XL_VLDL_L	B	mmol/L	Lipoprotein subclasses	Very large VLDL (average diameter 64 nm)	23489	23789
Total Lipids in Chylomicrons and	XXL_VLDL_L	B	mmol/L	Lipoprotein subclasses	Chylomicrons and extremely large	23482	23782

Extremely Large VLDL					VLDL (particle diameters from 75 nm upwards)		
Total Lipids in IDL	IDL_L	B	mmol/L	Lipoprotein subclasses	IDL (average diameter 28.6 nm)	23524	23824
Total Lipids in LDL	LDL_L	B	mmol/L	Total lipids		23425	23725
Total Lipids in Small LDL	S_LDL_L	B	mmol/L	Lipoprotein subclasses	Small LDL (average diameter 18.7 nm)	23545	23845
Total Lipids in Medium LDL	M_LDL_L	B	mmol/L	Lipoprotein subclasses	Medium LDL (average diameter 23 nm)	23538	23838
Total Lipids in Large LDL	L_LDL_L	B	mmol/L	Lipoprotein subclasses	Large LDL (average diameter 25.5 nm)	23531	23831
Total Lipids in HDL	HDL_L	B	mmol/L	Total lipids		23426	23726
Total Lipids in Small HDL	S_HDL_L	B	mmol/L	Lipoprotein subclasses	Small HDL (average diameter 8.7 nm)	23573	23873
Total Lipids in Medium HDL	M_HDL_L	B	mmol/L	Lipoprotein subclasses	Medium HDL (average diameter 10.9 nm)	23566	23866
Total Lipids in Large HDL	L_HDL_L	B	mmol/L	Lipoprotein subclasses	Large HDL (average diameter 12.1 nm)	23559	23859
Total Lipids in Very Large HDL	XL_HDL_L	B	mmol/L	Lipoprotein subclasses	Very large HDL (average diameter 14.3 nm)	23552	23852
Total Cholesterol	Total_C	C	mmol/L	Cholesterol		23400	23700
VLDL Cholesterol	VLDL_C	C	mmol/L	Cholesterol		23403	23703
Total Cholesterol in Very Small VLDL	XS_VLDL_C	C	mmol/L	Lipoprotein subclasses	Very small VLDL (average diameter 31.3 nm)	23519	23819
Total Cholesterol in Small VLDL	S_VLDL_C	C	mmol/L	Lipoprotein subclasses	Small VLDL (average diameter 36.8 nm)	23512	23812
Total Cholesterol in Medium VLDL	M_VLDL_C	C	mmol/L	Lipoprotein subclasses	Medium VLDL (average diameter 44.5 nm)	23505	23805

Total Cholesterol in Large VLDL	L_VLDL_C	C	mmol/L	Lipoprotein subclasses	Large VLDL (average diameter 53.6 nm)	23498	23798
Total Cholesterol in Very Large VLDL	XL_VLDL_C	C	mmol/L	Lipoprotein subclasses	Very large VLDL (average diameter 64 nm)	23491	23791
Total Cholesterol in Chylomicrons and Extremely Large VLDL	XXL_VLDL_C	C	mmol/L	Lipoprotein subclasses	Chylomicrons and extremely large VLDL (particle diameters from 75 nm upwards)	23484	23784
Total Cholesterol in IDL	IDL_C	C	mmol/L	Lipoprotein subclasses	IDL (average diameter 28.6 nm)	23526	23826
LDL Cholesterol	LDL_C	C	mmol/L	Cholesterol		23405	23705
Total Cholesterol in Small LDL	S_LDL_C	C	mmol/L	Lipoprotein subclasses	Small LDL (average diameter 18.7 nm)	23547	23847
Total Cholesterol in Medium LDL	M_LDL_C	C	mmol/L	Lipoprotein subclasses	Medium LDL (average diameter 23 nm)	23540	23840
Total Cholesterol in Large LDL	L_LDL_C	C	mmol/L	Lipoprotein subclasses	Large LDL (average diameter 25.5 nm)	23533	23833
HDL Cholesterol	HDL_C	C	mmol/L	Cholesterol		23406	23706
Total Cholesterol in Small HDL	S_HDL_C	C	mmol/L	Lipoprotein subclasses	Small HDL (average diameter 8.7 nm)	23575	23875
Total Cholesterol in Medium HDL	M_HDL_C	C	mmol/L	Lipoprotein subclasses	Medium HDL (average diameter 10.9 nm)	23568	23868
Total Cholesterol in Large HDL	L_HDL_C	C	mmol/L	Lipoprotein subclasses	Large HDL (average diameter 12.1 nm)	23561	23861
Total Cholesterol in Very Large HDL	XL_HDL_C	C	mmol/L	Lipoprotein subclasses	Very large HDL (average diameter 14.3 nm)	23554	23854
Total Free Cholesterol	Total_FC	D	mmol/L	Free cholesterol		23419	23719
Free Cholesterol in VLDL	VLDL_FC	D	mmol/L	Free cholesterol		23420	23720
Free Cholesterol in Very Small	XS_VLDL_FC	D	mmol/L	Lipoprotein subclasses	Very small VLDL (average diameter	23521	23821

VLDL					31.3 nm)		
Free Cholesterol in Small VLDL	S_VLDL_FC	D	mmol/L	Lipoprotein subclasses	Small VLDL (average diameter 36.8 nm)	23514	23814
Free Cholesterol in Medium VLDL	M_VLDL_FC	D	mmol/L	Lipoprotein subclasses	Medium VLDL (average diameter 44.5 nm)	23507	23807
Free Cholesterol in Large VLDL	L_VLDL_FC	D	mmol/L	Lipoprotein subclasses	Large VLDL (average diameter 53.6 nm)	23500	23800
Free Cholesterol in Very Large VLDL	XL_VLDL_FC	D	mmol/L	Lipoprotein subclasses	Very large VLDL (average diameter 64 nm)	23493	23793
Free Cholesterol in Chylomicrons and Extremely Large VLDL	XXL_VLDL_FC	D	mmol/L	Lipoprotein subclasses	Chylomicrons and extremely large VLDL (particle diameters from 75 nm upwards)	23486	23786
Free Cholesterol in IDL	IDL_FC	D	mmol/L	Lipoprotein subclasses	IDL (average diameter 28.6 nm)	23528	23828
Free Cholesterol in LDL	LDL_FC	D	mmol/L	Free cholesterol		23421	23721
Free Cholesterol in Small LDL	S_LDL_FC	D	mmol/L	Lipoprotein subclasses	Small LDL (average diameter 18.7 nm)	23549	23849
Free Cholesterol in Medium LDL	M_LDL_FC	D	mmol/L	Lipoprotein subclasses	Medium LDL (average diameter 23 nm)	23542	23842
Free Cholesterol in Large LDL	L_LDL_FC	D	mmol/L	Lipoprotein subclasses	Large LDL (average diameter 25.5 nm)	23535	23835
Free Cholesterol in HDL	HDL_FC	D	mmol/L	Free cholesterol		23422	23722
Free Cholesterol in Small HDL	S_HDL_FC	D	mmol/L	Lipoprotein subclasses	Small HDL (average diameter 8.7 nm)	23577	23877
Free Cholesterol in Medium HDL	M_HDL_FC	D	mmol/L	Lipoprotein subclasses	Medium HDL (average diameter 10.9 nm)	23570	23870
Free Cholesterol in Large HDL	L_HDL_FC	D	mmol/L	Lipoprotein subclasses	Large HDL (average diameter 12.1 nm)	23563	23863

Free Cholesterol in Very Large HDL	XL_HDL_FC	D	mmol/L	Lipoprotein subclasses	Very large HDL (average diameter 14.3 nm)	23556	23856
Total Esterified Cholesterol	Total_CE	E	mmol/L	Cholesteryl esters		23415	23715
Cholesteryl Esters in VLDL	VLDL_CE	E	mmol/L	Cholesteryl esters		23416	23716
Cholesteryl Esters in Very Small VLDL	XS_VLDL_CE	E	mmol/L	Lipoprotein subclasses	Very small VLDL (average diameter 31.3 nm)	23520	23820
Cholesteryl Esters in Small VLDL	S_VLDL_CE	E	mmol/L	Lipoprotein subclasses	Small VLDL (average diameter 36.8 nm)	23513	23813
Cholesteryl Esters in Medium VLDL	M_VLDL_CE	E	mmol/L	Lipoprotein subclasses	Medium VLDL (average diameter 44.5 nm)	23506	23806
Cholesteryl Esters in Large VLDL	L_VLDL_CE	E	mmol/L	Lipoprotein subclasses	Large VLDL (average diameter 53.6 nm)	23499	23799
Cholesteryl Esters in Very Large VLDL	XL_VLDL_CE	E	mmol/L	Lipoprotein subclasses	Very large VLDL (average diameter 64 nm)	23492	23792
Cholesteryl Esters in Chylomicrons and Extremely Large VLDL	XXL_VLDL_CE	E	mmol/L	Lipoprotein subclasses	Chylomicrons and extremely large VLDL (particle diameters from 75 nm upwards)	23485	23785
Cholesteryl Esters in IDL	IDL_CE	E	mmol/L	Lipoprotein subclasses	IDL (average diameter 28.6 nm)	23527	23827
Cholesteryl Esters in LDL	LDL_CE	E	mmol/L	Cholesteryl esters		23417	23717
Cholesteryl Esters in Small LDL	S_LDL_CE	E	mmol/L	Lipoprotein subclasses	Small LDL (average diameter 18.7 nm)	23548	23848
Cholesteryl Esters in Medium LDL	M_LDL_CE	E	mmol/L	Lipoprotein subclasses	Medium LDL (average diameter 23 nm)	23541	23841
Cholesteryl Esters in Large LDL	L_LDL_CE	E	mmol/L	Lipoprotein subclasses	Large LDL (average diameter 25.5 nm)	23534	23834
Cholesteryl Esters in HDL	HDL_CE	E	mmol/L	Cholesteryl esters		23418	23718
Cholesteryl Esters in Small HDL	S_HDL_CE	E	mmol/L	Lipoprotein subclasses	Small HDL (average diameter 8.7	23576	23876

					nm)		
Cholesteryl Esters in Medium HDL	M_HDL_CE	E	mmol/L	Lipoprotein subclasses	Medium HDL (average diameter 10.9 nm)	23569	23869
Cholesteryl Esters in Large HDL	L_HDL_CE	E	mmol/L	Lipoprotein subclasses	Large HDL (average diameter 12.1 nm)	23562	23862
Cholesteryl Esters in Very Large HDL	XL_HDL_CE	E	mmol/L	Lipoprotein subclasses	Very large HDL (average diameter 14.3 nm)	23555	23855
Total Triglycerides	Total_TG	F	mmol/L	Triglycerides		23407	23707
Triglycerides in VLDL	VLDL_TG	F	mmol/L	Triglycerides		23408	23708
Triglycerides in Very Small VLDL	XS_VLDL_TG	F	mmol/L	Lipoprotein subclasses	Very small VLDL (average diameter 31.3 nm)	23522	23822
Triglycerides in Small VLDL	S_VLDL_TG	F	mmol/L	Lipoprotein subclasses	Small VLDL (average diameter 36.8 nm)	23515	23815
Triglycerides in Medium VLDL	M_VLDL_TG	F	mmol/L	Lipoprotein subclasses	Medium VLDL (average diameter 44.5 nm)	23508	23808
Triglycerides in Large VLDL	L_VLDL_TG	F	mmol/L	Lipoprotein subclasses	Large VLDL (average diameter 53.6 nm)	23501	23801
Triglycerides in Very Large VLDL	XL_VLDL_TG	F	mmol/L	Lipoprotein subclasses	Very large VLDL (average diameter 64 nm)	23494	23794
Triglycerides in Chylomicrons and Extremely Large VLDL	XXL_VLDL_TG	F	mmol/L	Lipoprotein subclasses	Chylomicrons and extremely large VLDL (particle diameters from 75 nm upwards)	23487	23787
Triglycerides in IDL	IDL_TG	F	mmol/L	Lipoprotein subclasses	IDL (average diameter 28.6 nm)	23529	23829
Triglycerides in LDL	LDL_TG	F	mmol/L	Triglycerides		23409	23709
Triglycerides in Small LDL	S_LDL_TG	F	mmol/L	Lipoprotein subclasses	Small LDL (average diameter 18.7 nm)	23550	23850
Triglycerides in Medium LDL	M_LDL_TG	F	mmol/L	Lipoprotein subclasses	Medium LDL (average diameter 23	23543	23843

					nm)		
Triglycerides in Large LDL	L_LDL_TG	F	mmol/L	Lipoprotein subclasses	Large LDL (average diameter 25.5 nm)	23536	23836
Triglycerides in HDL	HDL_TG	F	mmol/L	Triglycerides		23410	23710
Triglycerides in Small HDL	S_HDL_TG	F	mmol/L	Lipoprotein subclasses	Small HDL (average diameter 8.7 nm)	23578	23878
Triglycerides in Medium HDL	M_HDL_TG	F	mmol/L	Lipoprotein subclasses	Medium HDL (average diameter 10.9 nm)	23571	23871
Triglycerides in Large HDL	L_HDL_TG	F	mmol/L	Lipoprotein subclasses	Large HDL (average diameter 12.1 nm)	23564	23864
Triglycerides in Very Large HDL	XL_HDL_TG	F	mmol/L	Lipoprotein subclasses	Very large HDL (average diameter 14.3 nm)	23557	23857
Total Phospholipids in Lipoprotein Particles	Total_PL	G	mmol/L	Phospholipids		23411	23711
Phospholipids in VLDL	VLDL_PL	G	mmol/L	Phospholipids		23412	23712
Phospholipids in Very Small VLDL	XS_VLDL_PL	G	mmol/L	Lipoprotein subclasses	Very small VLDL (average diameter 31.3 nm)	23518	23818
Phospholipids in Small VLDL	S_VLDL_PL	G	mmol/L	Lipoprotein subclasses	Small VLDL (average diameter 36.8 nm)	23511	23811
Phospholipids in Medium VLDL	M_VLDL_PL	G	mmol/L	Lipoprotein subclasses	Medium VLDL (average diameter 44.5 nm)	23504	23804
Phospholipids in Large VLDL	L_VLDL_PL	G	mmol/L	Lipoprotein subclasses	Large VLDL (average diameter 53.6 nm)	23497	23797
Phospholipids in Very Large VLDL	XL_VLDL_PL	G	mmol/L	Lipoprotein subclasses	Very large VLDL (average diameter 64 nm)	23490	23790
Phospholipids in Chylomicrons and Extremely Large VLDL	XXL_VLDL_PL	G	mmol/L	Lipoprotein subclasses	Chylomicrons and extremely large VLDL (particle diameters from 75	23483	23783

					nm upwards)		
Phospholipids in IDL	IDL_PL	G	mmol/L	Lipoprotein subclasses	IDL (average diameter 28.6 nm)	23525	23825
Phospholipids in LDL	LDL_PL	G	mmol/L	Phospholipids		23413	23713
Phospholipids in Small LDL	S_LDL_PL	G	mmol/L	Lipoprotein subclasses	Small LDL (average diameter 18.7 nm)	23546	23846
Phospholipids in Medium LDL	M_LDL_PL	G	mmol/L	Lipoprotein subclasses	Medium LDL (average diameter 23 nm)	23539	23839
Phospholipids in Large LDL	L_LDL_PL	G	mmol/L	Lipoprotein subclasses	Large LDL (average diameter 25.5 nm)	23532	23832
Phospholipids in HDL	HDL_PL	G	mmol/L	Phospholipids		23414	23714
Phospholipids in Small HDL	S_HDL_PL	G	mmol/L	Lipoprotein subclasses	Small HDL (average diameter 8.7 nm)	23574	23874
Phospholipids in Medium HDL	M_HDL_PL	G	mmol/L	Lipoprotein subclasses	Medium HDL (average diameter 10.9 nm)	23567	23867
Phospholipids in Large HDL	L_HDL_PL	G	mmol/L	Lipoprotein subclasses	Large HDL (average diameter 12.1 nm)	23560	23860
Phospholipids in Very Large HDL	XL_HDL_PL	G	mmol/L	Lipoprotein subclasses	Very large HDL (average diameter 14.3 nm)	23553	23853
Mean VLDL Particle Size	VLDL_size	H	nm	Lipoprotein particle sizes		23431	23731
Mean LDL Particle Size	LDL_size	H	nm	Lipoprotein particle sizes		23432	23732
Mean HDL Particle Size	HDL_size	H	nm	Lipoprotein particle sizes		23433	23733
Apolipoprotein A1	ApoA1	I	g/l	Apolipoproteins		23440	23740
Apolipoprotein B	ApoB	I	g/l	Apolipoproteins		23439	23739

(apo)lipoprotein profile: full name or description of the (apo)lipoprotein profile. Abbreviation: abbreviation name of the (apo)lipoprotein profile. Cluster: classification of (apo)lipoprotein

profile in this study from cluster A to I. Units: units of absolute concentration of the (apo)lipoprotein profile. Group: (apo)lipoprotein profile group as listed by Nightingale. Sub.Group: (apo)lipoprotein profile sub-group as listed by Nightingale. UKB.Field.ID: field ID for the (apo)lipoprotein profile in UK Biobank. QC.Flag.Field.ID: field ID for the corresponding (apo)lipoprotein profile measurement QC flags in UK Biobank.

Table S24. Food loadings(≤ -0.3 or ≥ 0.3) for principal components 1 to 4 (PC1 to PC4).

Food Variables	PC1	PC2	PC3	PC4
Salt	0.82			
Poultry	0.80			
Processed meat	0.78			
Beef	0.76			
Lamb/mutton	0.72			
Pork	0.72			
Non-oily fish	0.70			
Oily fish	0.67			
Cheese	0.35			
Cooked vegetables		0.76		
Raw vegetables		0.76		
Fresh fruit		0.44		0.46
water		0.34		
Tea			0.88	
Coffee			0.89	
Cereal				0.8
Dried fruit				0.6

Table S25. STROBE-MR checklist table.[1,2]

Item No.	Section	Checklist item	Page No.
1	**TITLE and ABSTRACT**	Indicate Mendelian randomization (MR) as the study's design in the title and/or the abstract if that is a main purpose of the study	1-2
	**INTRODUCTION**		
2	**Background**	Explain the scientific background and rationale for the reported study. What is the exposure? Is a potential causal relationship between exposure and outcome plausible? Justify why MR is a helpful method to address the study question	3-5
3	**Objectives**	State specific objectives clearly, including pre-specified causal hypotheses (if any). State that MR is a method that, under specific assumptions, intends to estimate causal effects	3-5
	**METHODS**		
4	**Study design and data sources**	Present key elements of the study design early in the article. Consider including a table listing sources of data for all phases of the study. For each data source contributing to the analysis, describe the following:	
	a)	Setting: Describe the study design and the underlying population, if possible. Describe the setting, locations, and relevant dates, including periods of recruitment, exposure, follow-up, and data collection, when available.	22-26
	b)	Participants: Give the eligibility criteria, and the sources and methods of selection of participants. Report the sample size, and whether any power or sample size calculations were carried out prior to the main analysis	22-26
	c)	Describe measurement, quality control and selection of genetic variants	22-26
	d)	For each exposure, outcome, and other relevant variables, describe methods of assessment and diagnostic criteria for diseases	22-26
	e)	Provide details of ethics committee approval and participant informed consent, if relevant	22-26
5	**Assumptions**	Explicitly state the three core IV assumptions for the main analysis (relevance, independence and exclusion restriction) as well assumptions for any additional or sensitivity analysis	22
6	**Statistical methods: main analysis**	Describe statistical methods and statistics used	

	a)	Describe how quantitative variables were handled in the analyses (i.e., scale, units, model)	22-26
	b)	Describe how genetic variants were handled in the analyses and, if applicable, how their weights were selected	22-26
	c)	Describe the MR estimator (e.g. two-stage least squares, Wald ratio) and related statistics. Detail the included covariates and, in case of two-sample MR, whether the same covariate set was used for adjustment in the two samples	22-26
	d)	Explain how missing data were addressed	-
	e)	If applicable, indicate how multiple testing was addressed	-
7	**Assessment of assumptions**	Describe any methods or prior knowledge used to assess the assumptions or justify their validity	22
8	**Sensitivity analyses and additional analyses**	Describe any sensitivity analyses or additional analyses performed (e.g. comparison of effect estimates from different approaches, independent replication, bias analytic techniques, validation of instruments, simulations)	22
9	**Software and pre- registration**		
	a)	Name statistical software and package(s), including version and settings used	22
	b)	State whether the study protocol and details were pre-registered (as well as when and where)	-
	**RESULTS**		
10	**Descriptive data**		
	a)	Report the numbers of individuals at each stage of included studies and reasons for exclusion. Consider use of a flow diagram	22, 24-26
	b)	Report summary statistics for phenotypic exposure(s), outcome(s), and other relevant variables (e.g. means, SDs, proportions)	-
	c)	If the data sources include meta-analyses of previous studies, provide the assessments of heterogeneity across these studies	-
	d)	For two-sample MR:<br>i. Provide justification of the similarity of the genetic variant-exposure associations between the exposure and outcome samples<br>ii. Provide information on the number of individuals who overlap between the exposure and outcome studies	22<br>-<br>-

11	**Main results**		
	a)	Report the associations between genetic variant and exposure, and between genetic variant and outcome, preferably on an interpretable scale	-
	b)	Report MR estimates of the relationship between exposure and outcome, and the measures of uncertainty from the MR analysis, on an interpretable scale, such as odds ratio or relative risk per SD difference	6-8, 10-12
	c)	If relevant, consider translating estimates of relative risk into absolute risk for a meaningful time period	-
	d)	Consider plots to visualize results (e.g. forest plot, scatterplot of associations between genetic variants and outcome versus between genetic variants and exposure)	41, 44, 47
12	**Assessment of assumptions**		
	a)	Report the assessment of the validity of the assumptions	6-7,10
	b)	Report any additional statistics (e.g., assessments of heterogeneity across genetic variants, such as I2, Q statistic or E-value)	6-7,10
13	**Sensitivity analyses and additional analyses**		
	a)	Report any sensitivity analyses to assess the robustness of the main results to violations of the assumptions	7, 8, 10
	b)	Report results from other sensitivity analyses or additional analyses	8,11
	c)	Report any assessment of direction of causal relationship (e.g., bidirectional MR)	-
	d)	When relevant, report and compare with estimates from non-MR analyses	7
	e)	Consider additional plots to visualize results (e.g., leave-one-out analyses)	-
	**DISCUSSION**		
14	**Key results**	Summarize key results with reference to study objectives	13-14
15	**Limitations**	Discuss limitations of the study, taking into account the validity of the IV assumptions, other sources of potential bias, and imprecision. Discuss both direction and magnitude of any potential bias and any efforts to address them	18
16	**Interpretation**		

	a)	Meaning: Give a cautious overall interpretation of results in the context of their limitations and in comparison with other studies	14-17
	b)	Mechanism: Discuss underlying biological mechanisms that could drive a potential causal relationship between the investigated exposure and the outcome, and whether the gene-environment equivalence assumption is reasonable. Use causal language carefully, clarifying that IV estimates may provide causal effects only under certain assumptions	14-17
	c)	Clinical relevance: Discuss whether the results have clinical or public policy relevance, and to what extent they inform effect sizes of possible interventions	14-17
17	**Generalizability**	Discuss the generalizability of the study results (a) to other populations, (b) across other exposure periods/timings, and (c) across other levels of exposure	18
	**OTHER INFORMATION**		
18	**Funding**	Describe sources of funding and the role of funders in the present study and, if applicable, sources of funding for the databases and original study or studies on which the present study is based	27
19	**Data and data sharing**	Provide the data used to perform all analyses or report where and how the data can be accessed, and reference these sources in the article. Provide the statistical code needed to reproduce the results in the article, or report whether the code is publicly accessible and if so, where	27
20	**Conflicts of Interest**	All authors should declare all potential conflicts of interest	27

This checklist is copyrighted by the Equator Network under the Creative Commons Attribution 3.0 Unported (CC BY 3.0) license.

1. Skrivankova VW, Richmond RC, Woolf BAR, Yarmolinsky J, Davies NM, Swanson SA, et al. Strengthening the Reporting of Observational Studies in Epidemiology using Mendelian Randomization (STROBE-MR) Statement. *JAMA*. 2021;under review.

2. Skrivankova VW, Richmond RC, Woolf BAR, Davies NM, Swanson SA, VanderWeele TJ, et al. Strengthening the Reporting of Observational Studies in Epidemiology using Mendelian Randomisation (STROBE-MR): Explanation and Elaboration. *BMJ*. 2021;375:n2233.

Using specified value of concentration parameter			Using conservative value of concentration parameter		
Overlap proportion	Bias	Type 1 error rate	Overlap proportion	Bias	Type 1 error rate
0.0	0.000	0.05	0.0	0.000	0.05
0.1	0.000	0.05	0.1	0.000	0.05
0.2	0.000	0.05	0.2	0.000	0.05
0.3	0.000	0.05	0.3	0.000	0.05
0.4	0.000	0.05	0.4	0.000	0.05
0.5	0.000	0.05	0.5	0.000	0.05
0.6	0.000	0.05	0.6	0.000	0.05
0.7	0.000	0.05	0.7	0.000	0.05
0.8	0.000	0.05	0.8	0.000	0.05
0.9	0.000	0.05	0.9	0.000	0.05
1.0	0.000	0.05	1.0	0.000	0.05

Concentration parameter (expected value of F statistic) = 108.02
Conservative value of concentration parameter (lower limit of one-sided 95% confidence interval) = 108.02

Figure S1. Bias and type 1 error rate for MR analysis of Total_P with mean cIMT.

Using specified value of concentration parameter			Using conservative value of concentration parameter		
Overlap proportion	Bias	Type 1 error rate	Overlap proportion	Bias	Type 1 error rate
0.0	0.000	0.05	0.0	0.000	0.05
0.1	0.000	0.05	0.1	0.000	0.05
0.2	0.000	0.05	0.2	0.000	0.05
0.3	0.000	0.05	0.3	0.000	0.05
0.4	0.000	0.05	0.4	0.000	0.05
0.5	0.000	0.05	0.5	0.000	0.05
0.6	0.000	0.05	0.6	0.000	0.05
0.7	0.000	0.05	0.7	0.000	0.05
0.8	0.000	0.05	0.8	0.000	0.05
0.9	0.000	0.05	0.9	0.000	0.05
1.0	0.000	0.05	1.0	0.000	0.05

Concentration parameter (expected value of F statistic) = 115.38
Conservative value of concentration parameter (lower limit of one-sided 95% confidence interval) = 115.37

Figure S2. Bias and type 1 error rate for MR analysis of HDL_P with mean cIMT.

Using specified value of concentration parameter			Using conservative value of concentration parameter		
Overlap proportion	Bias	Type 1 error rate	Overlap proportion	Bias	Type 1 error rate
0.0	0.000	0.05	0.0	0.000	0.05
0.1	0.000	0.05	0.1	0.000	0.05
0.2	0.000	0.05	0.2	0.000	0.05
0.3	0.000	0.05	0.3	0.000	0.05
0.4	0.000	0.05	0.4	0.000	0.05
0.5	0.000	0.05	0.5	0.000	0.05
0.6	0.000	0.05	0.6	0.000	0.05
0.7	0.000	0.05	0.7	0.000	0.05
0.8	0.000	0.05	0.8	0.000	0.05
0.9	0.000	0.05	0.9	0.000	0.05
1.0	0.000	0.05	1.0	0.000	0.05

Concentration parameter (expected value of F statistic) = 137.91
Conservative value of concentration parameter (lower limit of one-sided 95% confidence interval) = 137.91

Figure S3. Bias and type 1 error rate for MR analysis of M_HDL_P with mean cIMT.

Using specified value of concentration parameter			Using conservative value of concentration parameter		
Overlap proportion	Bias	Type 1 error rate	Overlap proportion	Bias	Type 1 error rate
0.0	0.000	0.05	0.0	0.000	0.05
0.1	0.000	0.05	0.1	0.000	0.05
0.2	0.000	0.05	0.2	0.000	0.05
0.3	0.000	0.05	0.3	0.000	0.05
0.4	0.000	0.05	0.4	0.000	0.05
0.5	0.000	0.05	0.5	0.000	0.05
0.6	0.000	0.05	0.6	0.000	0.05
0.7	0.000	0.05	0.7	0.000	0.05
0.8	0.000	0.05	0.8	0.000	0.05
0.9	0.000	0.05	0.9	0.000	0.05
1.0	0.000	0.05	1.0	0.000	0.05

Concentration parameter (expected value of F statistic) = 146.19
Conservative value of concentration parameter (lower limit of one-sided 95% confidence interval) = 146.19

Figure S4. Bias and type 1 error rate for MR analysis of HDL_L with mean cIMT.

Using specified value of concentration parameter			Using conservative value of concentration parameter		
Overlap proportion	Bias	Type 1 error rate	Overlap proportion	Bias	Type 1 error rate
0.0	0.000	0.05	0.0	0.000	0.05
0.1	0.000	0.05	0.1	0.000	0.05
0.2	0.000	0.05	0.2	0.000	0.05
0.3	0.000	0.05	0.3	0.000	0.05
0.4	0.000	0.05	0.4	0.000	0.05
0.5	0.000	0.05	0.5	0.000	0.05
0.6	0.000	0.05	0.6	0.000	0.05
0.7	0.000	0.05	0.7	0.000	0.05
0.8	0.000	0.05	0.8	0.000	0.05
0.9	0.000	0.05	0.9	0.000	0.05
1.0	0.000	0.05	1.0	0.000	0.05

Concentration parameter (expected value of F statistic) = 138.54
Conservative value of concentration parameter (lower limit of one-sided 95% confidence interval) = 138.53

Figure S5. Bias and type 1 error rate for MR analysis of M_HDL_L with mean cIMT.

Using specified value of concentration parameter			Using conservative value of concentration parameter		
Overlap proportion	Bias	Type 1 error rate	Overlap proportion	Bias	Type 1 error rate
0.0	0.000	0.05	0.0	0.000	0.05
0.1	0.000	0.05	0.1	0.000	0.05
0.2	0.000	0.05	0.2	0.000	0.05
0.3	0.000	0.05	0.3	0.000	0.05
0.4	0.000	0.05	0.4	0.000	0.05
0.5	0.000	0.05	0.5	0.000	0.05
0.6	0.000	0.05	0.6	0.000	0.05
0.7	0.000	0.05	0.7	0.000	0.05
0.8	0.000	0.05	0.8	0.000	0.05
0.9	0.000	0.05	0.9	0.000	0.05
1.0	0.000	0.05	1.0	0.000	0.05

Concentration parameter (expected value of F statistic) = 158.10
Conservative value of concentration parameter (lower limit of one-sided 95% confidence interval) = 158.09

Figure S6. Bias and type 1 error rate for MR analysis of L_HDL_L with mean cIMT.

Using specified value of concentration parameter			Using conservative value of concentration parameter		
Overlap proportion	Bias	Type 1 error rate	Overlap proportion	Bias	Type 1 error rate
0.0	0.000	0.05	0.0	0.000	0.05
0.1	0.000	0.05	0.1	0.000	0.05
0.2	0.000	0.05	0.2	0.000	0.05
0.3	0.000	0.05	0.3	0.000	0.05
0.4	0.000	0.05	0.4	0.000	0.05
0.5	0.000	0.05	0.5	0.000	0.05
0.6	0.000	0.05	0.6	0.000	0.05
0.7	0.000	0.05	0.7	0.000	0.05
0.8	0.000	0.05	0.8	0.000	0.05
0.9	0.000	0.05	0.9	0.000	0.05
1.0	0.000	0.05	1.0	0.000	0.05

Concentration parameter (expected value of F statistic) = 131.55
Conservative value of concentration parameter (lower limit of one-sided 95% confidence interval) = 131.54

Figure S7. Bias and type 1 error rate for MR analysis of HDL_C with mean cIMT.

Using specified value of concentration parameter			Using conservative value of concentration parameter		
Overlap proportion	Bias	Type 1 error rate	Overlap proportion	Bias	Type 1 error rate
0.0	0.000	0.05	0.0	0.000	0.05
0.1	0.000	0.05	0.1	0.000	0.05
0.2	0.000	0.05	0.2	0.000	0.05
0.3	0.000	0.05	0.3	0.000	0.05
0.4	0.000	0.05	0.4	0.000	0.05
0.5	0.000	0.05	0.5	0.000	0.05
0.6	0.000	0.05	0.6	0.000	0.05
0.7	0.000	0.05	0.7	0.000	0.05
0.8	0.000	0.05	0.8	0.000	0.05
0.9	0.000	0.05	0.9	0.000	0.05
1.0	0.000	0.05	1.0	0.000	0.05

Concentration parameter (expected value of F statistic) = 123.53
Conservative value of concentration parameter (lower limit of one-sided 95% confidence interval) = 123.53

Figure S8. Bias and type 1 error rate for MR analysis of M_HDL_C on mean cIMT.

Using specified value of concentration parameter			Using conservative value of concentration parameter		
Overlap proportion	Bias	Type 1 error rate	Overlap proportion	Bias	Type 1 error rate
0.0	0.000	0.05	0.0	0.000	0.05
0.1	0.000	0.05	0.1	0.000	0.05
0.2	0.000	0.05	0.2	0.000	0.05
0.3	0.000	0.05	0.3	0.000	0.05
0.4	0.000	0.05	0.4	0.000	0.05
0.5	0.000	0.05	0.5	0.000	0.05
0.6	0.000	0.05	0.6	0.000	0.05
0.7	0.000	0.05	0.7	0.000	0.05
0.8	0.000	0.05	0.8	0.000	0.05
0.9	0.000	0.05	0.9	0.000	0.05
1.0	0.000	0.05	1.0	0.000	0.05

Concentration parameter (expected value of F statistic) = 136.86
Conservative value of concentration parameter (lower limit of one-sided 95% confidence interval) = 136.85

Figure S9. Bias and type 1 error rate for MR analysis of M_HDL_FC with mean cIMT.

Using specified value of concentration parameter			Using conservative value of concentration parameter		
Overlap proportion	Bias	Type 1 error rate	Overlap proportion	Bias	Type 1 error rate
0.0	0.000	0.05	0.0	0.000	0.05
0.1	0.000	0.05	0.1	0.000	0.05
0.2	0.000	0.05	0.2	0.000	0.05
0.3	0.000	0.05	0.3	0.000	0.05
0.4	0.000	0.05	0.4	0.000	0.05
0.5	0.000	0.05	0.5	0.000	0.05
0.6	0.000	0.05	0.6	0.000	0.05
0.7	0.000	0.05	0.7	0.000	0.05
0.8	0.000	0.05	0.8	0.000	0.05
0.9	0.000	0.05	0.9	0.000	0.05
1.0	0.000	0.05	1.0	0.000	0.05

Concentration parameter (expected value of F statistic) = 127.99
Conservative value of concentration parameter (lower limit of one-sided 95% confidence interval) = 127.99

Figure S10. Bias and type 1 error rate for MR analysis of HDL_CE with mean cIMT.

Using specified value of concentration parameter			Using conservative value of concentration parameter		
Overlap proportion	Bias	Type 1 error rate	Overlap proportion	Bias	Type 1 error rate
0.0	0.000	0.05	0.0	0.000	0.05
0.1	0.000	0.05	0.1	0.000	0.05
0.2	0.000	0.05	0.2	0.000	0.05
0.3	0.000	0.05	0.3	0.000	0.05
0.4	0.000	0.05	0.4	0.000	0.05
0.5	0.000	0.05	0.5	0.000	0.05
0.6	0.000	0.05	0.6	0.000	0.05
0.7	0.000	0.05	0.7	0.000	0.05
0.8	0.000	0.05	0.8	0.000	0.05
0.9	0.000	0.05	0.9	0.000	0.05
1.0	0.000	0.05	1.0	0.000	0.05

Concentration parameter (expected value of F statistic) = 92.55
Conservative value of concentration parameter (lower limit of one-sided 95% confidence interval) = 92.54

Figure S11. Bias and type 1 error rate for MR analysis of M_HDL_CE with mean cIMT.

Using specified value of concentration parameter			Using conservative value of concentration parameter		
Overlap proportion	Bias	Type 1 error rate	Overlap proportion	Bias	Type 1 error rate
0.0	0.000	0.05	0.0	0.000	0.05
0.1	0.000	0.05	0.1	0.000	0.05
0.2	0.000	0.05	0.2	0.000	0.05
0.3	0.000	0.05	0.3	0.000	0.05
0.4	0.000	0.05	0.4	0.000	0.05
0.5	0.000	0.05	0.5	0.000	0.05
0.6	0.000	0.05	0.6	0.000	0.05
0.7	0.000	0.05	0.7	0.000	0.05
0.8	0.000	0.05	0.8	0.000	0.05
0.9	0.000	0.05	0.9	0.000	0.05
1.0	0.000	0.05	1.0	0.000	0.05

Concentration parameter (expected value of F statistic) = 154.47
Conservative value of concentration parameter (lower limit of one-sided 95% confidence interval) = 154.46

Figure S12. Bias and type 1 error rate for MR analysis of L_HDL_CE with mean cIMT.

Using specified value of concentration parameter			Using conservative value of concentration parameter		
Overlap proportion	Bias	Type 1 error rate	Overlap proportion	Bias	Type 1 error rate
0.0	0.000	0.05	0.0	0.000	0.05
0.1	0.000	0.05	0.1	0.000	0.05
0.2	0.000	0.05	0.2	0.000	0.05
0.3	0.000	0.05	0.3	0.000	0.05
0.4	0.000	0.05	0.4	0.000	0.05
0.5	0.000	0.05	0.5	0.000	0.05
0.6	0.000	0.05	0.6	0.000	0.05
0.7	0.000	0.05	0.7	0.000	0.05
0.8	0.000	0.05	0.8	0.000	0.05
0.9	0.000	0.05	0.9	0.000	0.05
1.0	0.000	0.05	1.0	0.000	0.05

Concentration parameter (expected value of F statistic) = 156.77
Conservative value of concentration parameter (lower limit of one-sided 95% confidence interval) = 156.76

Figure S13. Bias and type 1 error rate for MR analysis of HDL_PL with mean cIMT.

Using specified value of concentration parameter			Using conservative value of concentration parameter		
Overlap proportion	Bias	Type 1 error rate	Overlap proportion	Bias	Type 1 error rate
0.0	0.000	0.05	0.0	0.000	0.05
0.1	0.000	0.05	0.1	0.000	0.05
0.2	0.000	0.05	0.2	0.000	0.05
0.3	0.000	0.05	0.3	0.000	0.05
0.4	0.000	0.05	0.4	0.000	0.05
0.5	0.000	0.05	0.5	0.000	0.05
0.6	0.000	0.05	0.6	0.000	0.05
0.7	0.000	0.05	0.7	0.000	0.05
0.8	0.000	0.05	0.8	0.000	0.05
0.9	0.000	0.05	0.9	0.000	0.05
1.0	0.000	0.05	1.0	0.000	0.05

Concentration parameter (expected value of F statistic) = 169.75
Conservative value of concentration parameter (lower limit of one-sided 95% confidence interval) = 169.75

Figure S14. Bias and type 1 error rate for MR analysis of L_HDL_PL with mean cIMT.

Using specified value of concentration parameter			Using conservative value of concentration parameter		
Overlap proportion	Bias	Type 1 error rate	Overlap proportion	Bias	Type 1 error rate
0.0	0.000	0.05	0.0	0.000	0.05
0.1	0.000	0.05	0.1	0.000	0.05
0.2	0.000	0.05	0.2	0.000	0.05
0.3	0.000	0.05	0.3	0.000	0.05
0.4	0.000	0.05	0.4	0.000	0.05
0.5	0.000	0.05	0.5	0.000	0.05
0.6	0.000	0.05	0.6	0.000	0.05
0.7	0.000	0.05	0.7	0.000	0.05
0.8	0.000	0.05	0.8	0.000	0.05
0.9	0.000	0.05	0.9	0.000	0.05
1.0	0.000	0.05	1.0	0.000	0.05

Concentration parameter (expected value of F statistic) = 161.77
Conservative value of concentration parameter (lower limit of one-sided 95% confidence interval) = 161.76

Figure S15. Bias and type 1 error rate for MR analysis of HDL_size with mean cIMT.

```
Using specified value of concentration parameter        Using conservative value of concentration parameter
Overlap proportion       Bias     Type 1 error rate       Overlap proportion       Bias     Type 1 error rate
------------------------------------------------        ------------------------------------------------
0.0                      0.000    0.05                    0.0                      0.000    0.05
0.1                      0.000    0.05                    0.1                      0.000    0.05
0.2                      0.000    0.05                    0.2                      0.000    0.05
0.3                      0.000    0.05                    0.3                      0.000    0.05
0.4                      0.000    0.05                    0.4                      0.000    0.05
0.5                      0.000    0.05                    0.5                      0.000    0.05
0.6                      0.000    0.05                    0.6                      0.000    0.05
0.7                      0.000    0.05                    0.7                      0.000    0.05
0.8                      0.000    0.05                    0.8                      0.000    0.05
0.9                      0.000    0.05                    0.9                      0.000    0.05
1.0                      0.000    0.05                    1.0                      0.000    0.05


Concentration parameter (expected value of F statistic) = 139.58
Conservative value of concentration parameter (lower limit of one-sided 95% confidence interval) = 139.58
```

Figure S16. Bias and type 1 error rate for MR analysis of ApoA1 with mean cIMT.

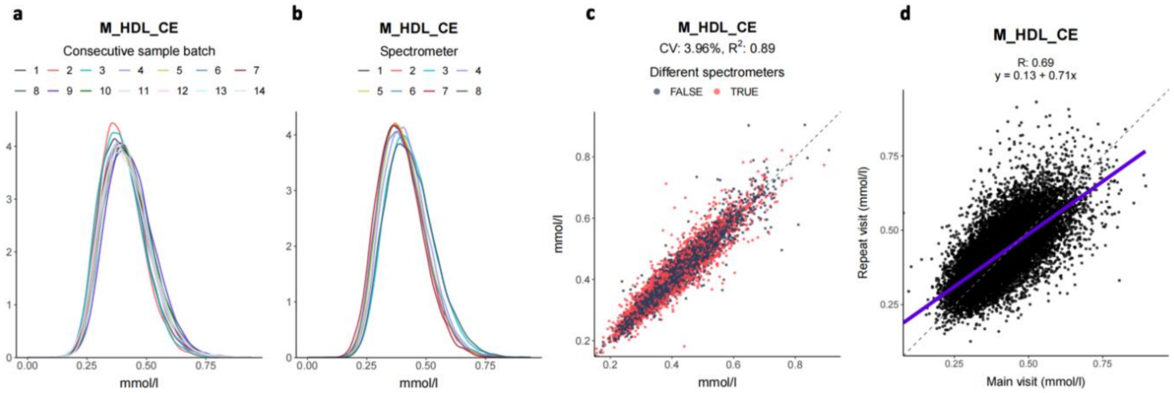

Figure S17. Technical and biological repeatability for cholesteryl esters in medium HDL (M_HDL_CE). Technical consistency in terms of a) distributions of consecutive batches of sample shipments, b) distributions in different spectrometers, c) consistency of ~650 blind duplicates samples (giving rise to a between-instrument CV of 3.96%). Panel d) shows the biological repeatability for measurements from blood samples from the same individuals drawn ~4 years apart for approximately 1500 samples. The corresponding plots for each biomarker is given in the UK Biobank data resource (https://biobank.ndph.ox.ac.uk/showcase/label.cgi?id=220).
